# High ectomycorrhizal relative abundance during winter at the treeline

**DOI:** 10.1093/ismeco/ycaf010

**Published:** 2025-01-25

**Authors:** Luis A Saona, Christian I Oporto, Pablo Villarreal, Kamila Urbina, Cristian Correa, Julian F Quintero-Galvis, Paulo Moreno-Meynard, Frida I Piper, Juliana A Vianna, Roberto F Nespolo, Francisco A Cubillos

**Affiliations:** Facultad de Química y Biología, Departamento de Biología, Universidad de Santiago de Chile, Santiago 9170124, Chile; Millennium Nucleus of Patagonian Limit of Life, Valdivia 5110566, Chile; Facultad de Química y Biología, Departamento de Biología, Universidad de Santiago de Chile, Santiago 9170124, Chile; Millennium Nucleus of Patagonian Limit of Life, Valdivia 5110566, Chile; Facultad de Química y Biología, Departamento de Biología, Universidad de Santiago de Chile, Santiago 9170124, Chile; Centro Científico y Tecnológico de Excelencia Ciencia & Vida, Fundación Ciencia & Vida, Huechuraba 8580702, Santiago, Chile; Millennium Institute for Integrative Biology, Santiago 8331150, Chile; Facultad de Química y Biología, Departamento de Biología, Universidad de Santiago de Chile, Santiago 9170124, Chile; Millennium Nucleus of Patagonian Limit of Life, Valdivia 5110566, Chile; Millennium Institute for Integrative Biology, Santiago 8331150, Chile; Instituto de Conservación, Biodiversidad y Territorio, Universidad Austral de Chile, Valdivia 5110566, Chile; Millennium Nucleus of Patagonian Limit of Life, Valdivia 5110566, Chile; Facultad de Ciencias, Instituto de Ciencias Ambientales y Evolutivas, Universidad Austral de Chile, Valdivia 5110566, Chile; Millennium Nucleus of Patagonian Limit of Life, Valdivia 5110566, Chile; Centro de Investigación en Ecosistemas de la Patagonia, Coyhaique 5951369, Chile; Millennium Nucleus of Patagonian Limit of Life, Valdivia 5110566, Chile; Instituto de Cs. Biológicas, Universidad de Talca, Campus Lircay, Talca 3460000, Chile; Instituto de Ecología y Biodiversidad, Santiago 7800003, Chile; Millennium Nucleus of Patagonian Limit of Life, Valdivia 5110566, Chile; Laboratorio de Biodiversidad Molecular, Facultad de Ciencias Biológicas, Instituto para el Desarrollo Sustentable, Pontificia Universidad Católica de Chile, Santiago 6904411, Chile; Millennium Institute Center for Genome Regulation, Santiago 8580745, Chile; Millennium Institute Biodiversity of Antarctic and Subantarctic Ecosystems, Santiago 6904411, Chile; Millennium Nucleus of Patagonian Limit of Life, Valdivia 5110566, Chile; Millennium Institute for Integrative Biology, Santiago 8331150, Chile; Facultad de Ciencias, Instituto de Ciencias Ambientales y Evolutivas, Universidad Austral de Chile, Valdivia 5110566, Chile; Center of Applied Ecology and Sustainability, Santiago, 6904411, Chile; Facultad de Química y Biología, Departamento de Biología, Universidad de Santiago de Chile, Santiago 9170124, Chile; Millennium Nucleus of Patagonian Limit of Life, Valdivia 5110566, Chile; Millennium Institute for Integrative Biology, Santiago 8331150, Chile

**Keywords:** treeline, ectomycorrhiza, saprotroph, rhizosphere, temperate forests, *Nothofagus*, *Cortinarius*

## Abstract

The rhizosphere is the soil region around plant roots hosting a diverse microbial community, influencing nutrient availability and how plants react to extreme conditions. However, our understanding of the fungi biodiversity and the impact of environmental variations on this biodiversity is still in its infancy. Our study investigates fungal communities’ diversity and functional traits in the rhizosphere of *Nothofagus pumilio,* one of the few winters deciduous treeline species in the world, forming the treeline in southern South America. At four distinct locations covering 10° latitude, we collected soil samples at treeline and 200 m below over four seasons during a single year. We employed ITS metabarcoding to elucidate fungal community structures. Our results reveal that fungal diversity was mainly determined by latitudinal variation, with higher levels during warmer seasons and lower altitudes. Interestingly, we found a marked dominance of ectomycorrhizal fungi at the treeline, particularly during the winter. In contrast, saprotrophic fungi were more abundant at lower altitudes, particularly during the warmer spring and summer seasons. These findings highlight the temporal and spatial dynamics of rhizospheric fungal communities and their potential roles in ecological processes, emphasizing the value of these communities as indicators of environmental change in high-elevation forests.

## Introduction

The rhizosphere is the soil region surrounding plant roots hosting a diverse microbial community. This microbiome aids nutrient cycling, enhances plant growth through nutrient solubilization and hormone production, and influences plant defense mechanisms and plants’ response to extreme conditions [1]. Within this interface, fungi establish a variety of symbiotic relationships with trees, facilitating the uptake of nutrients through mycorrhizal associations [2, 3], improving stress tolerance [2, 4, 5], and contributing to the overall stability and productivity of the forest ecosystem [6]. Among the key fungal guilds to this ecosystem are ectomycorrhizal (EcM) and saprotrophs, whose ecological roles are crucial in the forest [7–10]. EcM forms symbiotic associations with plant roots, which are fundamental to the tree’s nutrition and water stress response [11–13]. Saprotrophs significantly contribute to the decomposition of organic matter and nutrient cycling, which are essential for forest sustainability [14–16]. Nonetheless, our understanding of the factors influencing the composition of the rhizosphere and the diversity of associated organisms across different forest ecosystems, especially in the context of climate change, is still in its early stages of development [6]. In this context, seasonal variations and altitudinal gradients emerge as critical factors in examining the effects of climate change on rhizosphere composition and associated biotic communities [17–20].

The ‘treeline’ represents the ecotone where trees cease to grow at higher altitudes, marking the transition from forested to treeless elevations [21]. Notably, the treeline is influenced by the mean annual temperature and decreasing nitrogen levels with increasing altitude [22]. Around 70% of mountain treelines are currently experiencing shifts to higher elevations at an ~1.2 m per year rate, a value that increases to 3.1 m per year in tropical regions [23]. Thus, under the climate change scenario, treeline dynamics are intrinsically immersed in biodiversity conservation and mountain ecosystem management. Although the treeline’s extreme conditions and ecological importance have been recognised, the diversity and functionality of the rhizosphere fungal community in the treeline have not been systematically and comprehensively characterized [17, 20, 23].

The treeline of Southern South America *Nothofagus* Forests (SSAN forests, hereafter) is mostly dominated by *Nothofagus pumilio,* a deciduous tree species that can reach up to 30-m heights with an average lifespan of 200–300 years. *N. pumilio* can survive in low-temperature environments and thrive under reduced nutrient availability soil conditions [22]. This adaptation is reflected in its role as a dominant species in sub-Antarctic forests [24, 25]. These interactions, particularly the EcM associations detected in the *N. pumilio* rhizosphere [26, 27], indicate a symbiotic role between EcM and *N. pumilio*, facilitating survival and normal growth under unfavourable conditions [27]. In this sense, ~73%–79% of *N. pumilio* trees in the Argentinian Patagonia exhibit some EcM colonization [27, 28]. Similar studies in Chilean Patagonia indicate that EcM-*N. pumilio* abundance increases with altitude [29]. However, this altitudinal pattern has only been reported in Tierra del Fuego during the summer season [29]. Consequently, it remains unclear whether these findings apply to other latitudes or seasons, such as the treeline, thereby limiting a comprehensive understanding of the role of EcM in the adaptation of *N. pumilio* to extremely cold environments. This limitation emphasizes the need to determine how complex fungal interactions contribute to the SSAN forest ecosystem resilience and health under climate change conditions.

This study describes the dynamics of fungal communities in the *N. pumilio* rhizosphere at the treeline ecotone. We evaluated the effects of the altitude, latitude, and seasonal changes in the fungal biodiversity. We obtained rhizosphere samples from *N. pumilio* collected at four sampling points in Chilean Patagonia, considering two distinct altitudes (treeline and below-treeline) across the four yearly seasons. We examined the fungal community’s diversity through metabarcoding and provided evidence of the dynamic relationships between EcM and saprotrophic fungi across different environmental conditions. We hypothesize that EcM fungi dominate under extremely cold conditions at the treeline due to their potential ecological advantages under unfavourable conditions [27]. Consequently, we predict a higher relative abundance of EcM fungi at the treeline during winter compared to other seasons and altitudes, where saprotrophs are expected to be more prevalent. This research underscores the importance of monitoring fungal community dynamics as bioindicators for assessing the impacts of climate change on forest ecosystem resilience and function.

## Materials and methods

### Sampling sites

**Figure 1:**
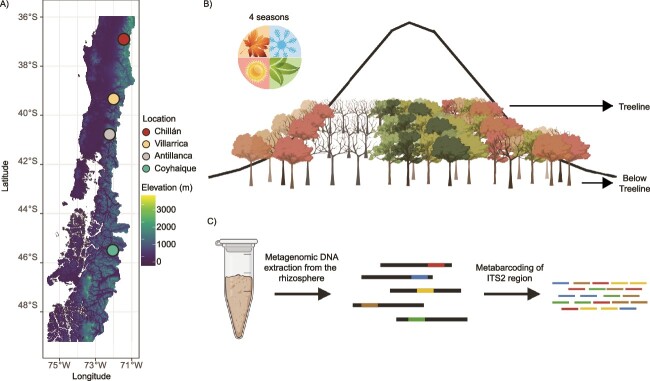
**Study design and sample processing in Patagonian *Nothofagus* forests**. (A) Sampling site maps created using elevation raster data depicting the specific collection locations within the study area. (B) Sampling strategy highlighting sample collection at two different forest elevations (at the treeline and 200 m below the treeline) across all four seasons to capture seasonal variability in microbial community composition. (C) Sample processing workflow from field collection and DNA extraction to amplicon library preparation for metabarcoding analysis, detailing the key steps in soil microbial diversity analysis. Created with BioRender.com

Rhizosphere samples were collected from four distinct geographical locations in central Chilean Patagonia (Fig. 1A**,**  Table S1). The selected locations include Nevados de Chillan (36.91°S), Villarrica National Park (39.28°S), Puyehue National Park (Antillanca) (40.78°S), and Coyhaique National Reserve (45.51°S) (Fig. 1A). The criteria for site selection were: (i) minimal human disturbance and absence of landslide or avalanche impacts; (ii) presence of a clearly defined treeline; (iii) driving accessibility throughout the year; and (iv) predominance of *N. pumilio* trees, offering a uniform context for rhizosphere sampling [30]. Soil sample collection required intense effort, particularly during winter when snow depth exceeds 3 m, sometimes covering trees entirely. Despite these challenges, the sampling design (two elevations, four localities, see below) successfully addresses the logistical complexities of deep snow and harsh winter conditions.

In addition to the latitudinal gradient, we incorporated an altitudinal component at each site. Two elevation points were designated: the treeline (T) at the upper limit of *N. pumilio* forest growth, and below treeline (BT), a point ~200 vertical m below the treeline as previously reported [31] (Fig. 1B). Based on environmental data obtained from the WorldClim database, mean annual air temperatures across the sites ranged from 7.4°C to 11.5°C, with minimum daily averages during winter reaching −8.1°C at Coyhaique and maximum daily averages up to 23.5°C during summer at Chillán (Table S1). These data provide a general representation of atmospheric conditions, as we did not directly measure soil or rhizosphere temperatures. However, during winter sampling, we observed snow accumulation ranging from 1 to 3 m above ground level at most sites. This factor likely influences soil thermal dynamics by insulating it from extreme air temperatures. While site-specific snow depth or duration measurements were not recorded, these field observations, combined with prior studies, frame the ‘extreme’ conditions described in the manuscript as a combination of prolonged snow cover, subzero air temperatures, and limited resource availability during winter.

Our sampling campaigns spanned 2022 and 2023, encompassing all four seasons: April 2022 (fall), June 2022 (winter), October 2022 (spring), and January 2023 (summer). These dates, marking the onset of each season, align with critical phenological stages in *N. pumilio* forests, such as leaf colour change, shedding, or new leaf production.

### Rhizosphere sample collection

Soil samples from *N. pumilio*’s rhizosphere were dug with a clean garden hand shovel at 10–20 cm depth and packed in labelled 50 ml sterile centrifuge tubes. The sampling process was initiated in the fall of 2022 and involved collecting three samples per location, altitude, and season. This approach evolved in the subsequent seasons—winter, spring, and summer—during which the number of samples was increased to five per site. To ensure a comprehensive sampling representation, each sample was gathered from an area encompassing at least five trees, aiming to cover the rhizosphere of over 15 trees during fall and extending to 25 trees in the subsequent seasons (winter, spring, and summer) at each site and altitude. The same trees identified in the first sampling campaign were consistently sampled in later collections, provided that environmental conditions allowed the corresponding sampling campaign. For example, heavy snowfall occasionally hindered whole tree access. All samples were stored at 4°C within 1 day until DNA extraction was performed within 10 days. This sampling protocol was successfully completed, except for Coyhaique at the treeline in the fall 2022, which could not be reached due to bad weather conditions and heavy snowfall. Therefore, a total of 141 samples were collected in four locations, two altitudes, and four seasons (Table S1).

### DNA extraction and sequencing

DNA extraction was carried out using the Geneaid Presto™ Soil DNA Extraction Kit (cat. No. SLD100). For this, ~0.5 g of soil was used separately from each sample (141 extractions), following the manufacturer’s instructions. The DNA obtained was then used as a template to amplify the internal nuclear ribosomal transcribed spacer 2 (ITS2), using the ITS3 forward primer (5′-GCATCGATGAAGAACGCAGC- 3′) and the ITS4 reverse primer (5′-TCCTCCGCTTATTGATATATATGC-3′) [32]. The primers were designed by adding Illumina adapters, using the forward sequence (5´-TCGTCGGCAGCGTCAGATGTGTATAAGAGACAG-3′) and reverse sequence (5´-GTCTCGTGGGCTCGGAGATGTGTATAAGAGACAG–3′) as described in the Illumina metagenomic ITS sequencing library preparation protocol (Illumina Inc., San Diego, CA) (Fig. 1C). Library preparation was performed as described in the Illumina ITS metagenomics guide (Illumina Inc., San Diego, CA), with the difference that equimolar pools of the samples with different barcodes were initially generated, which were amplified using the Illumina DNA/RNA UD Indexes Set B, Tagmentation (96 Indexes, 96 Samples). Libraries were quantified using the QubitTM 4 Fluorometer (cat. no. Q33226) and fragment analysis was performed on the TapeStation 2200. Subsequently, the libraries were sequenced using the Illumina MiSeq equipment with 300 bp paired-end reads at the sequencing facilities of the Faculty of Medicine at the Universidad de Chile.

### Processing of ITS2 sequence data

We used Qiime2 [33] for demultiplexing the samples depending on their corresponding barcode, using the commands *‘qiime tools import –type MultiplexedPairedEndBarcodeInSequence’* and *‘qiime cutadapt demux-paired’*, resulting in the isolation of 141 individual samples (Table S2). Raw samples were processed in R using the DADA2 pipeline with default settings [34]. Both forward and reverse sequences’ quality profiles were scrutinized and filtered using the *‘filter and trim’* function (Table S2). The taxonomic assignment for each Amplicon Sequence Variant (ASV) was performed using the UNITE database [35].

The phyloseq package was then used to create comprehensive taxonomic information for each ASV (*tax_table* function), an abundance object for each ASV per sample (*otu_table* function), and the associated metadata (*sample_data* function, Table S3). These individual objects were merged using the *merge_phyloseq* function into a unified phyloseq object, which was subsequently utilized for further analysis [36]. This unified phyloseq object facilitated the exportation of ASV abundance data for integration with Funguild [37]. Taxonomic assignments and data visualization were performed using the ggplot2 package [38]. Within the Funguild analysis, an abundance threshold greater than 0.5% was applied to focus on guilds of ecological significance. Furthermore, any ASVs identified as associated with more than one distinct guild were classified as ‘unidentified-ecology’ to preclude the misclassification of ASVs potentially associated with multiple guilds as strictly EcM, saprotrophic, endophytic, etc [39]. The final list of all ASVs identified in all samples, along with their taxonomic assignment down to species level and their ecological role as determined by FunGuild.

### Physicochemical analysis

Physicochemical analysis was performed on soil samples obtained in the summer campaign. For this purpose, ~300 g of soil associated with the rhizosphere was taken from the first five trees marked by altitude and mixed to complete 1.5 kg per altitude. Samples were stored in sterile bags at 4°C until processing. Due to the number of samples needed for the analysis and the extreme climatic conditions, samples from the fall and winter seasons were not obtained, so the analysis was performed only with summer samples.

Analyses of pH, organic matter (OM) and nutrients were performed by Laboratorio Agroanálisis UC, Facultad de Agronomía y Sistemas Naturales, Pontificia Universidad Católica de Chile, according to the methods established by the Standardization and Accreditation Commission (CNA) of the Chilean Society of Soil Science [40]. The nutrients measured were N, P, K, Zn, S, B, Ca, and Mg.

### Statistical analysis

Data filtering and statistical analysis were conducted using R (version 4.3.1) for the alpha diversity analysis of rhizospheric fungal communities. We utilized the *phyloseq* package for handling and analysing sequencing data and *dplyr* for data manipulations. Alpha diversity (including species richness, Shannon entropy, and Simpson-Giani concentration) was assessed using Hill numbers, which provide effective numbers of species and offer a coherent statistical framework for comparing biodiversity across multiple scales [41, 42]. Specifically, Shannon entropy corresponds to q = 1, and the Simpson-Giani concentration corresponds to q = 2 within the Hill number framework. All calculations were performed using standard implementations of Hill numbers, ensuring that diversity metrics are directly comparable and biologically interpretable. Visualization of results was performed with *ggplot2*. Statistical significance was assessed using generalized linear models (GLM), specifically fitting negative binomial models to account for overdispersion observed in the count data. The richness observed was modelled as a function of location and season, with interactions considered where appropriate. Model comparisons were made using the *drop1* function to evaluate the significance of each term by sequentially dropping them from the full model. The step-by-step analyses are shown in the Supplementary material.

For beta diversity analysis, the *phyloseq* package was employed to prepare and manipulate the data objects. Sample pseudo-replication was handled by merging samples from the same sites and seasons using the *merge_samples* method to ensure consistent sampling units at a landscape scale. Principal Coordinates Analysis (PCoA) based on Bray–Curtis dissimilarity (tb-PCoA) and Redundancy Analysis (tb-RDA) based on Hellinger-transformed data were conducted to explore the relationships between community composition and environmental variables. Minimum (T_min), mean (T_mean),, and maximum (T_max) temperatures obtained from the WorldClim database were added as variables. Variance Inflation Factor analyses were performed to assess multicollinearity among predictors. When high covariance was detected, the affected variables were excluded to optimize model performance. The significance of modelled gradients and effects was tested by permutation tests, ensuring robust inference of environmental impacts on microbial community structure. These analytical procedures are outlined in the Supplementary Material section.

For the statistical analysis of soil physicochemical properties, data manipulation, and tb-RDA were applied, as detailed in the supplementary material, beta diversity. The soil samples were aggregated and statistically analysed using R. The primary statistical methodology involved tb-RDA to explore the relationships between soil physicochemical properties and microbial community structures. Initially, Box-Cox transformations transformed soil data to normalize distributions and reduce skewness. This pre-processing step ensured more reliable statistical inference by stabilizing variance across the dataset. The tb-RDA model incorporated variables such as organic matter, nitrogen, phosphorus, and sulfur content, selected through a stepwise model selection process. This selection was based on their statistical significance and contribution to explaining the variance observed in microbial community compositions. The model’s effectiveness was assessed via permutation tests, confirming the significance of the selected model terms (*P* < .05).

To predict the impact of environmental factors on fungal guild relative abundance within the rhizosphere of *N. pumilio* we employed a Boosted Regression Tree (BRT) analysis [43]. Using the *gbm.step* function allowed us to fine-tune the models in relation to tree complexity, learning rate, and bag fraction. Given the relatively small number of sites, we opted for a tc of 2 [44]. To compensate for reduced tree length, a slower learning rate (0.001) was implemented to reach a minimum of 1000 trees. A bag fraction of 0.75 utilized 75% of the data for fitting and 25% for prediction [45]. Employing a Gaussian family of relationships and 10-fold cross-validation, we determined interactions between predictors following Elith et al. (2008).

To measure the relative impact of environmental variables encompassing season, altitude, and geographic location, we aggregated the relative influence of each chosen predictor, focusing on two predominant fungal guilds: EcM and saprotrophs [46].

The fitted functions of the BRT model were visualized using partial dependence plot [46]. Lastly, model efficiency was assessed through the percentage of explained deviance [(mean total deviance – mean residual deviance)/mean total deviance] and cross-validation correlation coefficients [45, 47]. This comprehensive analysis gave insights into the patterns of variation in fungal guilds with respect to environmental factors in the rhizosphere of *N. pumilio*.

## Results

### Fungal biodiversity is mainly determined by latitudinal variation in the *N. Pumilio* rhizosphere

Samples were analysed using DADA2 to identify the ASVs present in *N. pumilio* rhizosphere soils. After filtering and rarefaction to confirm that all samples achieved adequate sequencing depth (Fig. S1), we identified 5739 different ASVs across the 141 samples (Table S3). Four samples were discarded because they needed to meet the minimum threshold of 5000 reads per sample.

**Figure 2:**
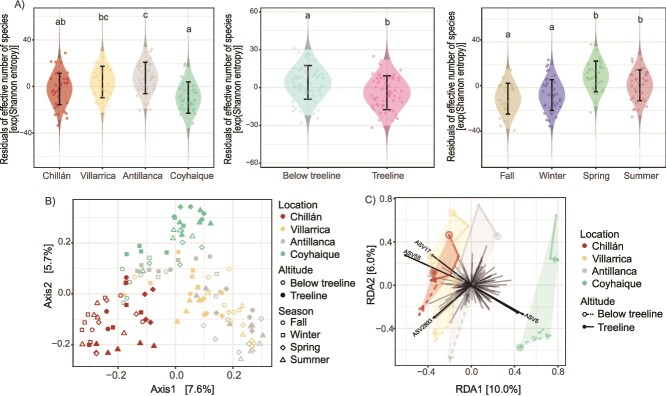
**Fungal alpha diversity, and unconstrained ordination of rhizosphere fungal community composition.** (A) Residual diversity plots of diversity, exp (Shannon index), illustrating the marginal contribution of location (left panel), altitude (center), and season (right) to the overall diversity variation. In the background, normal homoscedastic violin plots represent the density function of model predictions. (B) Beta diversity is expressed as PCoA based on bray-Curtis pairwise distances. (C) Visualization of the tbRDA results. Samples from the same location were connected with edges beginning from a start circle in winter and ending with arrowheads in summer. Altitude is also distinguished with different edge type and start-circles.

We conducted an alpha diversity analysis estimating observed richness, Shannon entropy, and Simpson-Gini concentration to determine the fungal biodiversity in the *N. pumilio* rhizosphere and how it varies by season, altitude, and location. Richness analysis through observed ASVs revealed differences among variables (Fig. S2). While the effect of season (negative binomial GLM, χ^2^ = 25.26; df = 3) and location (χ^2^ = 20.09; df = 3; *P* < .001) was highly significant (*P* < .001), the effect of altitude was not statistically significant (χ^2^ = 0.98, df = 1, *P* = .323), nor the interaction terms (Supplementary material: alpha diversity). On the other hand, the linear model of Shannon’s entropy index (Fig. 2A) revealed a significant effect of locality, altitude, and season (*P* < .001), which together accounted for 33.4% of the variance (Adj. R^2^). We found the highest diversities in spring, specifically below the treeline in Antillanca. In contrast, the lowest diversities were estimated during fall and winter, particularly at the treeline in the coldest southernmost location, Coyhaique (Fig. 2A).

PCoA was applied to explore the composition of the microbial communities, revealing distinct patterns of sample clustering (Fig. 2B). Notably, the geographical locations of Antillanca and Villarrica exhibited considerable overlap in the ordination space across all seasons, suggesting a similar rhizospheric fungal composition between these two geographically close areas (Fig. 1A). On the contrary, Coyhaique and Chillán were distributed more distantly, reflecting marked differences in their fungal communities (Fig. 2B).

Transformation-based redundancy analysis (tb-RDA) was conducted to evaluate the influence of environmental variables on microbial community structure. Following bidirectional stepwise model selection, we found that the best model explaining the fungal community composition included season, altitude, maximum temperature (T_max), and season:altitude (see Supplementary material: beta diversity). All constraining variables, in the best tb-RDA model, explain 32.8% of the variation in the fungi community data, and the first two constraining axes explain 16.0% (Fig. 2C). The interpretation of the tb-RDA plot could relate the first axis (RDA1) to latitudinal variation, and the second (RDA2) to seasonal and altitudinal variation. Notably, ASV5, ASV17, and ASV55 emerged as influential taxa (vectors) in the RDA plot, reflecting significant differences in their relative abundances among sites. For example, ASV5 exhibited a higher average relative abundance in Coyhaique (3.58%) compared to Chillán (0.438%), Villarrica (0.003%), and was absent in Antillanca. Similarly, ASV17 was more abundant in Chillán (3.16%) and Villarrica (1.07%) than in Coyhaique (0.340%) and Antillanca (0.139%). ASV55 showed higher relative abundances in Chillán (3.25%) and Villarrica (2.46%) compared to Antillanca (0.465%) and Coyhaique (0.032%). PERMANOVA of the marginal effect of each model term while controlling for the effects of all others indicated that all model terms of the best model were statistically significant (*P* < .05).

### Cortinariaceae (Basidiomycota) is the most abundant family in the winter treeline

**Figure 3:**
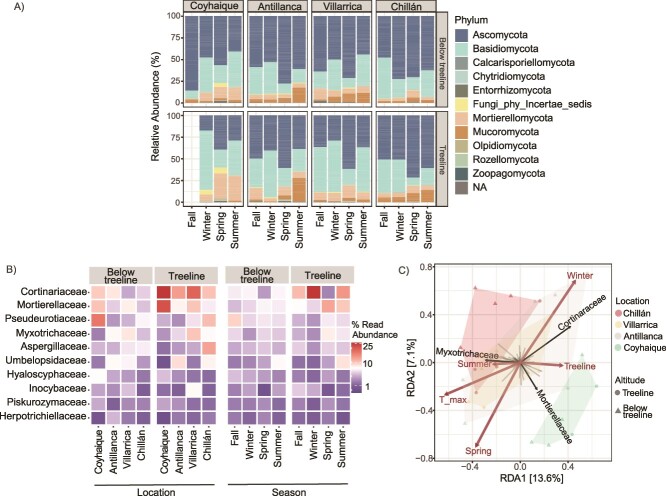
**Taxonomic distribution of fungal communities in the rhizosphere of *N. Pumilio*.** (A) Spatio-temporal patterns of community composition of fungal phyla in the rhizosphere of *N. Pumilio*. (B) Spatial distribution of the top-10 most abundant fungal families. (C) tb-RDA plot of the family-level aggregated data set. Family scores as well as constraining variables of the same ordination were plotted separated for clarity.

Examination of rhizosphere-associated fungal communities in *N. pumilio* forests at varying locations, altitudes, and seasons revealed pronounced changes within the fungal kingdom, where Ascomycota and Basidiomycota were the most abundant phyla across all samples (Fig. 3A, Table S4 and S5). On one hand, Ascomycota notably increases relative abundance during the spring and in areas below the treeline. In contrast, Basidiomycota ASVs were significantly enriched in colder winter conditions, particularly at the treeline (Fig. 3A, Table S5). This trend was consistently observed at all study locations, indicating a strong altitudinal and seasonal trend specificity in the distribution of this phylum. Beyond these dominant phyla, we observed fluctuations in the relative abundance of other phyla, including Mortierellomycota, Mucoromycota and ‘Fungi incertae sedis’. Although less pronounced, these variations suggest a diverse and responsive fungal community within the rhizosphere. Statistical analyses confirmed that location, season, and altitude significantly influenced the relative abundance of fungal ASVs (GLM, *P* < .05). Notably, the interaction between winter and the treeline was significant (*P* = .018), indicating distinct shifts in the relative abundance of fungal ASVs under these conditions. Additionally, interactions between location and season were significant, particularly in Antillanca and Chillán during spring (*P* < .05), underscoring site-specific seasonal dynamics. The triple interaction (Location:Season:Altitude), however, did not improve model fit and was excluded for parsimony. The analysis was extended to the level of fungal families, revealing distinct patterns (Fig. 3B). Dominant families varied mostly by site at lower elevations. Interestingly, the Cortinariaceae family was predominantly abundant at the treeline during winter at most sites.

To further understand this pattern, we analysed the composition of the Cortinariaceae. We identified 233 ASVs at the species level within this family across all samples (Table S4). In the Coyhaique treeline during winter, the high abundance of Cortinariaceae was predominantly due to certain ASVs. Notably, ASV1790 (*Cortinarius fluorescens*) was the most dominant ASV across sites, seasons and altitudes, accounting for 45.1% of the total relative abundance of Cortinariaceae. This ASV was exclusively found in the Coyhaique treeline during winter and was absent in other locations and seasons. Similarly, ASV1794 (19.2%) and ASV1792 (18.6%) were unique to these specific conditions. While some ASVs like ASV1793 and ASV498 were present in other locations, their relative abundances were significantly lower than Coyhaique.

To determine common and unique ASVs among different variables considered in this study, a comparative analysis of the distribution and specificity of the ASVs was conducted (Fig. S3). We identified 323 ASVs common to all seasons, 100 ASVs shared across various locations, and 972 ASVs throughout the different altitudes. Notably, although the Cortinariaceae family is the most abundant in the overall composition (Fig. 3B), the Mortierellaceae family dominates among the ASVs shared across different seasons, locations, and altitudinal gradients (Fig. S3). To statistically validate the higher relative abundance of the Cortinariaceae family during winter at the treeline, a tb-RDA was performed. This time, the model focused on family-level classifications rather than the total ASV abundance. The axes explained 13.6% (RDA1) and 7.1% (RDA2) of the variance in the data, both of which were statistically significant (*P* < .05, Fig. 3C). Next, the variance explained by each individual variable in the best tb-RDA model was assessed. The variable T_max accounted for 14.7% of the total constrained variance, while the interaction between season and altitude accounted for 5.4%. These results highlight the significant influence of maximum temperature and the combined effect of season and altitude on the fungal community structure (Fig. 3C), with Cortinariaceae being linked to extreme conditions associated with winter and the treeline.

**Figure 4:**
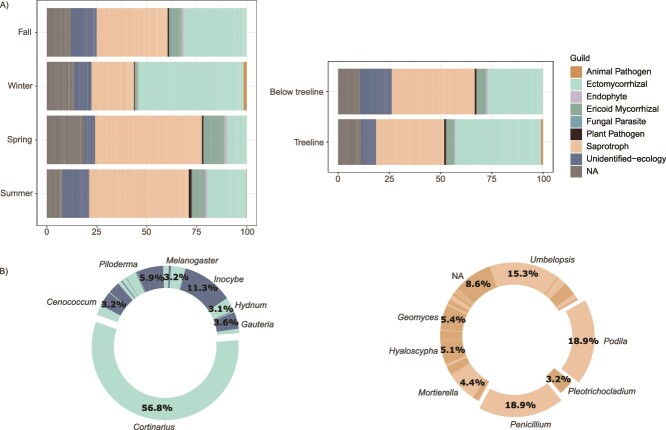
**Fungal guild abundance variation in the rhizosphere of *N. Pumilio*.** (A) Bar charts indicate the abundance of fungal guilds in the rhizosphere of *N. Pumilio*, categorized by season (fall, winter, spring, summer) and altitude (treeline and below treeline). (B) Donut charts displaying the genera representing ASVs identified as ectomycorrhizal and saprotroph, complementing the information in (A) by detailing the genera constituting these dominant functional guilds. The most abundant genera are highlighted: *Cortinarius* representing 56.8% of the ectomycorrhizal guild, while *Podila* and *Penicillium* each constitute 18.9% of the saprotrophic guild.

To elucidate the ecological roles of the fungal communities across different altitudes, locations, and seasons, we assessed the taxonomic distribution of the sequenced samples using the Funguild database. Our results revealed a marked spatial and temporal variation in the fungal guild’s composition (Fig. 4A). Using Fisher’s Exact Test, we further validated these patterns, showing that the relative proportion of EcM fungi was significantly higher during winter compared to summer across all altitudes (Odds Ratio = 3.25, *P* < .001). In comparison, the relative proportion of saprotrophic fungi was significantly higher in summer compared to winter (Odds Ratio = 0.49, *P* = .002). Regarding altitudinal patterns, the relative proportion of EcM fungi was higher at the treeline than below (Odds Ratio = 1.64, *P* = .056). The relative proportion of saprotrophic fungi did not show significant differences between altitudes (Odds Ratio = 0.71, *P* = .15).

The functional guild composition across all samples is predominantly characterized by an assemblage of EcM and saprotrophic fungi (Fig. 4A). Within the EcM guild, the genus *Cortinarius* is particularly prominent, accounting for 56.8% of the relative abundance (Fig. 4B), which is consistent with the observed dominance in the most abundant families (Fig. 3). In the saprotrophic category, the genera *Podila* and *Penicillium* are the most abundant, representing 18.9% of the abundance within this guild on each case (Fig. 4C).

### Seasonal changes determine guild abundance in the SSAN forest

**Figure 5:**
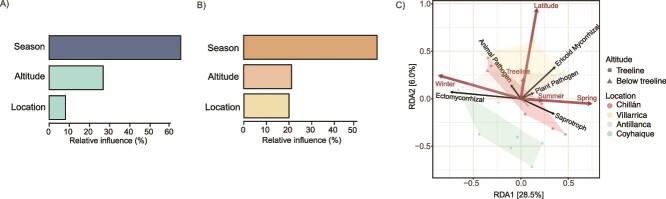
**Variables influencing fungal guilds in the rhizosphere of *N. Pumilio*.** (A) Bar charts from a BRT analysis showing the relative influence of seasonal, altitudinal, and geographic variables on EcM fungi abundance. (B) Similar BRT analysis detailing the influence of the same variables on saprotrophic fungi abundance. Both a and B demonstrate the season as a significant factor. (C) tb-RDA plot of the data set, separated by guild, to visualize the relationship between guild scores and constraining variables.

Through BRT analysis, we quantified the association of EcM (Fig. 5A) and saprotrophic fungi (Fig. 5B) with environmental factors, such as season, altitude, and location. The seasonal variable emerged as the primary factor influencing the abundance of both fungal guilds, contributing 64.2% to the explanatory power of the EcM model, followed by altitude and location with contributions of 25.7% and 10.2%, respectively (Fig. 5A). For saprotrophic fungi, the contributions were 59.0% for the season, 21.1% for altitude, and 19.9% for location (Fig. 5B). The tb-RDA plot clarified that EcM guilds are directly associated to winter conditions, while saprotrophs are associated with spring (Fig. 5C). This visual representation supports the BRT analysis findings, providing an integrative view of how fungal guilds are influenced by seasonality and altitude. To simplify the full model into a more parsimonious and less redundant model, the tb-RDA model was refined through bidirectional stepwise selection. This model included latitude, season, altitude, and the season:altitude interaction, explaining 53.1% of the variance (R^2^), with a penalized adjusted R^2^ of 36.6%. This indicates that while our model accounts for over half of the observed variation, the remaining variation might be attributed to other environmental or stochastic processes not captured within our study’s scope. The significance of the tb-RDA’s first constraining axis was validated statistically, and PERMANOVA confirmed the statistical significance of all model terms (*P* < .05, Supplementary material: beta diversity). Thus, these results prove that the latitude, season, altitude, and the season:altitude interaction significantly influence the fungal community structure in the rhizosphere of *N. pumilio*.

To evaluate the influence of the summer soil physicochemical properties on fungal community composition at the ASV level (Table S6), a tb-RDA was performed. Our model incorporated log-transformed nutrient content variables, including organic matter, nitrogen, phosphorus, and sulphur. The model elucidated that these variables collectively explained 69% of the variance in fungal community data, with the first two axes accounting for 48.2%. Specifically, log_10_ (Phosphorus) emerged as the sole nutrient significantly correlating with community composition, as evidenced by PERMANOVA (*P* < .05) (Supplementary material, beta diversity). For the analysis focusing on fungal guilds, a refined model highlighted log_10_ (Sulphur) as a significant predictor, explaining 30.3% of community variance, with adjusted R^2^ indicating an 18.7% explanation after penalizing for the number of predictors. The axes explained 38.8% (RDA1) and 15.2% (RDA2) of the variance in the data, both of which were statistically significant (*P* < .05, Fig. S4). PERMANOVA results affirmed the statistical significance of log_10_ (Sulphur) in shaping guild composition (*P* < .05), indicating a correlation between the abundance of EcM and Sulphur (Fig. S4).

## Discussion

Our study deepens the current understanding of the fungal ecology associated with the rhizosphere of *N. pumilio* at the treeline in Patagonian temperate forests. Employing a robust sampling design encompassing four latitudes, four seasons, and two altitudes, we observed a fungal community sensitive to each analyzed factor, revealing a dynamic spatiotemporal distribution of diversity and functions. Notably, our results suggest a relatively higher tolerance of EcM fungi to cold conditions than other guilds, as evidenced by their increased relative proportion during winter and treeline. More than 90% of forest trees in temperate and boreal zones form EcM associations [48, 49], while most ground vegetation associates with arbuscular mycorrhizal or ericoid mycorrhizal fungi [48, 50]. The structure of EcM communities varies with host species, temperature, geographic location, and soil properties, among other factors. However, the effects of season and altitude associated with temperature variations on the abundance and distribution of EcM have yet to be explored. Interestingly, we observed a higher relative abundance of EcM fungi under substantial snow cover in cold conditions [51, 52]. Most research on extreme environments, such as the treeline, has not examined the influence of seasonal and altitudinal variation on the structure of the rhizospheric fungal community, thus providing only a partial picture [26, 29]. The treeline represents a critical ecotone that is highly sensitive to climate change and environmental variations because it is controlled by low temperature [51]. Understanding the microbial dynamics at the treeline can provide insights into how ecosystem functions and biodiversity may respond to global climate change, offering essential information for conservation and management strategies [52–54].

Surprisingly, despite variations in alpha and beta diversity and taxonomic profiles among the four surveyed forests, the ecological roles represented by fungal guilds remained consistent between forests. This reflects the concept of functional redundancy, where diverse taxa can perform similar ecological functions, ensuring their persistence even under microbial biodiversity loss. In macroecological terms, this phenomenon has been explained as the maintenance of ecosystem functions by diverse species, reducing the likelihood of functional loss despite changes in community composition [55, 56]. Examples of this phenomenon are seen in litter-decomposing fungal communities, where species maintain consistent nutrient cycling functions despite taxonomic shifts [57, 58]. Similar redundancy has been observed in EcM fungal communities, where diverse taxa contribute equivalently to soil enzymatic activities, particularly in carbon and nutrient cycling, ensuring ecosystem functionality despite changes in fungal composition [59]. Furthermore, the consistent directional trends in ASV composition vectors across seasons (Fig. 2C) suggest that despite the geographic and altitudinal differences, fungal communities at each site undergo seasonal shifts similarly. This shared seasonal modulation implies that common environmental or biotic factors likely influence the variation in the rhizosphere fungal communities, indicating a degree of ecosystem stability in how fungal communities respond to seasonal changes, as similar patterns of variation are observed consistently across all sampling sites. Building on this, while site-specific differences in fungal diversity and composition were evident, soil conditions such as sulphur concentrations emerge as crucial environmental factors influencing fungal guilds (Fig. S4). Our study highlights the interaction between sulphur concentrations and EcM abundance during the summer. This seasonal observation suggests that sulphur dynamics and availability, crucial for plant growth and ecosystem health [60], are strongly related to higher relative EcM abundance. Although these observations expand our understanding of EcM interactions with macronutrients in soil, the limitation to summer samples makes further seasonal studies necessary to fully capture the dynamics of these essential microbial processes throughout the year.

The ubiquitous presence of Ascomycota and Basidiomycota across diverse geographies and environmental conditions underscores their ecological robustness, likely backed by a broad functional repertoire that enables their persistence in various environmental niches [61–63]. Notably, the seasonal and altitudinal patterns observed in this work, especially with Basidiomycota thriving at the treeline during winter, highlight their relative abundance under cold stress or perhaps their dominance due to the decrease in other fungal groups under such harsh conditions. This ability to endure extreme conditions may reflect a broader ecological strategy, where organisms capable of surviving in challenging environments take advantage of reduced competition in such niches [64].

Although tree metabolism is low during winter, a higher relative abundance of EcM may play a role in the nitrogen supply required by *N. pumilio* to endure the harsh conditions at the treeline in winter [65]. Remarkably, our analysis detected a significantly higher relative abundance of EcM at the treeline than just 200 m below treeline. This conspicuous change in EcM dominance occurring along such a narrow altitudinal range may respond to the fact that soil nitrogen availability steeply decreases with elevation and is significantly lower at the treeline than below it [66–68]. Indeed, previous studies demonstrated that a positive correlation between a higher relative abundance of EcM fungi and enzymatic capacity for organic N mobilization [65]. Thus, winter-active EcM, if confirmed, may benefit host trees during the harsh Patagonian winter and eventually explain the dominance of a winter deciduous species in the treeline, which is exceptional compared to most other treelines [22, 69]. Other studies have shown that EcM not only survived in cold environments but was metabolically active and the most abundant guild in low temperatures conditions, even freezing [70, 71]. A coherent pattern has also been described along an altitudinal gradient [72], Nevertheless, our study is the first one demonstrating the increasing relative abundance of EcM at the treeline, which is a worldwide limit of the tree growth form controlled by low temperatures. This result adds to the mounting evidence showing distinct altitudinal and seasonal patterns in fungal community composition under cold conditions. On the contrary, Koizumi et al. (2020) observed that summer temperatures in *Pinus pumilia* forests in Japanese archipelagos are the main parameter that forms the EcM community, particularly during the hottest summer months. However, they did not conduct winter samplings. Interestingly, they identified that EcM species such as *Russula emetica*, *Suillus spraguei*, *Suillus sp.*, and *Tomentella sp*. showed higher relative frequency as temperatures increases; while species like *Cortinarius acutus*, *Cortinarius aurantiobasis*, and *Cortinarius junghuhnii* showed higher relative frequency at lower temperatures [73]. Similarly, deep-snow treatments revealed that snow significantly reduced the number of EcM, although dominant genera like *Cortinarius*, *Inocybe*, and *Tomentella* were unaffected [74]. This revealed a correlation between low temperatures and EcM of the genus *Cortinarius*, which agrees with our findings. In our study, the Cortinariaceae family is the most abundant in winter and treeline, with the *Cortinarius* genus being the most abundant EcM in our samples. This finding agrees with a previous study that identified Cortinariaceae as the most abundant EcM in a plot at the treeline of *N. pumilio* across three years, using morpho-anatomic identification [26]. The Cortinariaceae family has been previously linked to trees of the Nothofagaceae family. Cortinariaceae was the most abundant family in *N. pumilio* seedlings in Argentinian Patagonia [75]. Similarly, in the southernmost tip of continental Chile, Cortinariaceae was among the most abundant in the rhizosphere of *N. pumilio* at different elevations [29]. In New Zealand, *Cortinarius* was the most abundant genus in the soil associated with *N. menziesii* [76]. This body of evidence suggests a key role for the Cortinariaceae family in the rhizosphere of *N. pumilio* in Patagonian temperate forests, especially under extremely low-temperature conditions.

In summary, this study offers a compelling picture of spatiotemporal patterns of rhizospheric fungal communities in SSAN forests. Our findings demonstrate a significant prevalence of ectomycorrhizas during winter and in high-altitude regions at the treeline. The increased relative abundance of these symbiotic relationships during the harshest season and at this ecological edge suggests their potential importance in shaping rhizosphere dynamics under cold conditions. These results raise new hypotheses about the ecological role of EcM fungi in supporting plant life at the edge of Patagonian forests, which future studies can further explore.

## Supplementary Material

Sup_Figures_ycaf010

Table_S1_ycaf010

Table_S2_ycaf010

Table_S3_ycaf010

241213_Tabla_S4_ycaf010

Table_S5_ycaf010

Table_S6_ycaf010

Supplementary_Material_ycaf010

250122_Supplementary_Legends_ycaf010

## Data Availability

All raw data from the metabarcoding sequencing have been deposited in the Sequence Read Archive of the National Center for Biotechnology Information under BioProject accession number PRJNA1133747.
