# Supplementary figures and images for: High ectomycorrhizal relative abundance during winter at the treeline
Source: ISME Commun. 2025 Jan 25;5(1):ycaf010. doi: 10.1093/ismeco/ycaf010 (PMC11815889; doi:10.1093/ismeco/ycaf010)

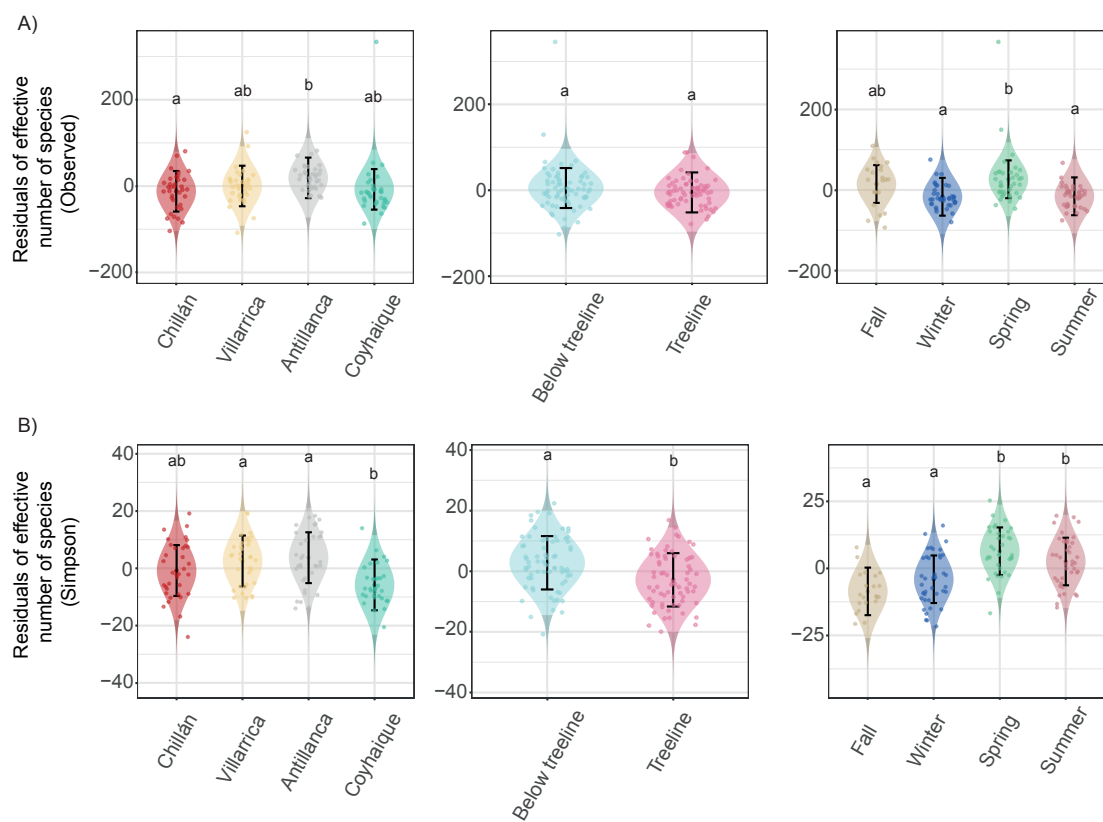

Fig. S2

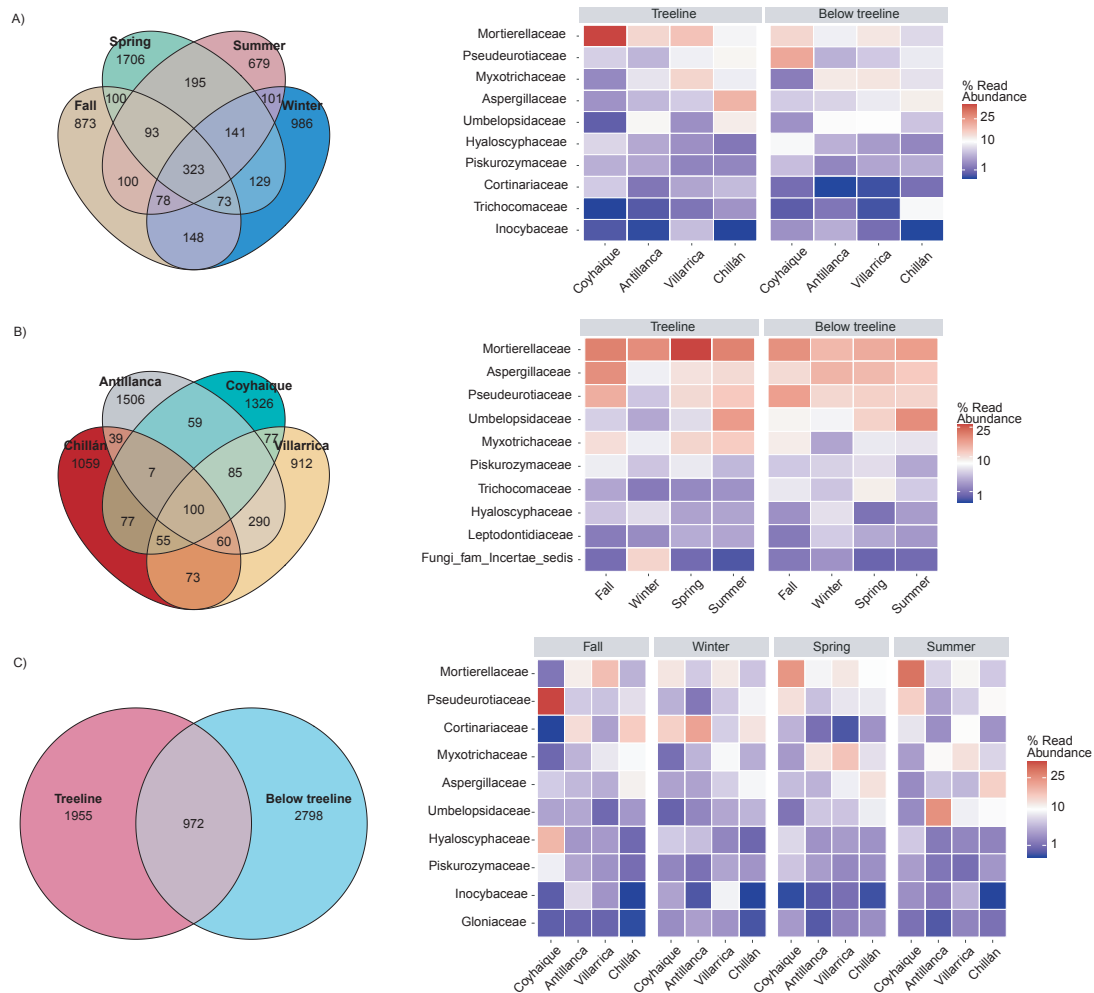

Fig. S3

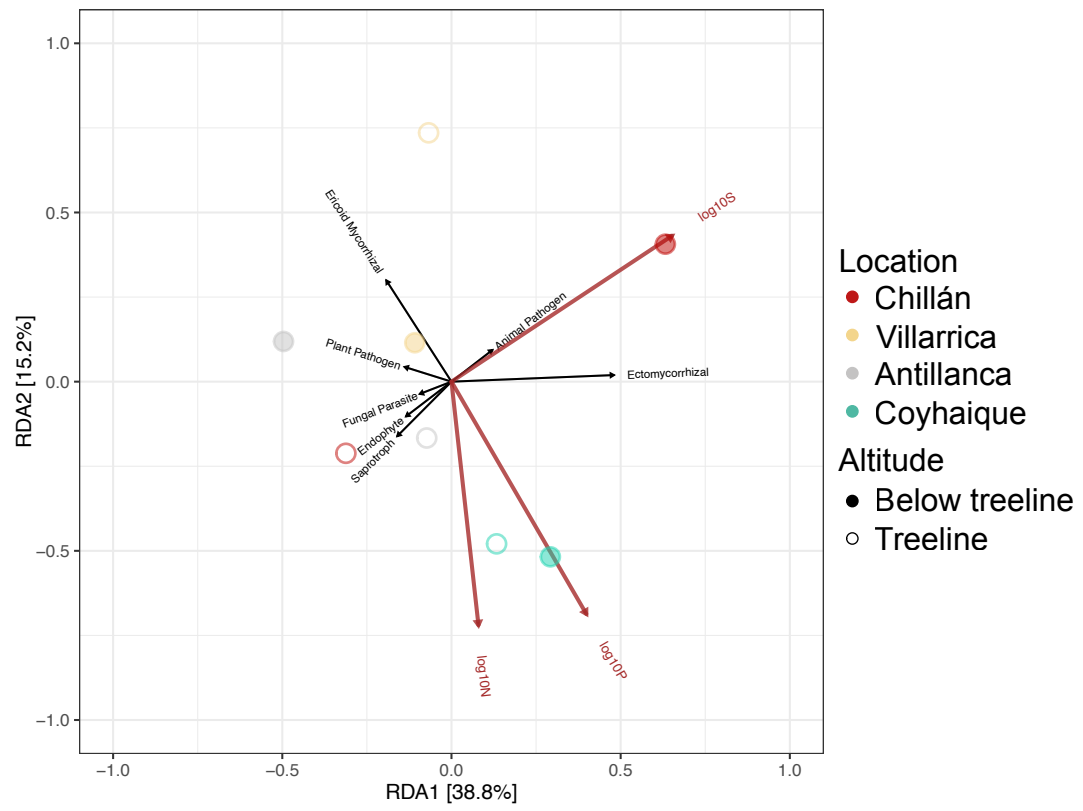

Fig. S4

Supplement: Sup_Figures_ycaf010 [file sup_figures_ycaf010.pdf]
