# Supplementary material for: High ectomycorrhizal relative abundance during winter at the treeline
Source: ISME Commun. 2025 Jan 25;5(1):ycaf010. doi: 10.1093/ismeco/ycaf010 (PMC11815889; doi:10.1093/ismeco/ycaf010)
Supplement: Supplementary_Material_ycaf010 [file supplementary_material_ycaf010.pdf]

SUPPLEMENTARY METERIAL: **High ectomycorrhizal abundance during ecosystem hibernation at the treeline**

Chapter 1: Alpha diversity

1. System

This document was produced in R Markdown, including a narrative written in LATEX and data handling and analyses conducted in R (R Core Team, 2021).

1.1. System and R version

R version 4.3.1 (2023-06-16)
Platform: x86_64-pc-linux-gnu (64-bit)
Running under: Ubuntu 20.04.6 LTS

1.2. R Libraries

The following libraries were used.

```
rm(list = ls())
# install.packages('bookdown') devtools::install_github('joey711/phyloseq')
library(phyloseq)  # http://joey711.github.io/phyloseq/
library("tidyr")  # pivot_longer
library("stringr") library("ggplot2")
library("tibble")
# library('recipes') library('plyr') # join functions
library("dplyr")
# # library('Cairo') install.packages('kableExtra', dependencies = T)
# remove.packages('kableExtra') devtools::install_github('kupietz/kableExtra')
library("kableExtra")  # great pretty tables, pipeline flow
library("ggmap")
# library(rgdal) library(rgeos) # need to migrate to package sf!  library(sp)
```

```r
# library('grid') # arrange ggplots together
library("gridExtra") # arrange ggplots together
# library(rgbif) library(lubridate) library(dbscan) # find point clusters
```

2. Preamble

During the last decade altitude gradients have gained attention as a model to understand the effects of global warming. Much reseach was focused on vegetation composition, morphology and physiology, and a common phenomenon is the existence of a functional treeline described as an altitude above which forest cover and vigor quickly decline. Hence, a steep ecological cline develos at about the height of the tree line, and it is hypothetized that that the compositipn and function of the microbiome also shift from below to above the tree line. In this study we describe rizospheric fungal community patterns at the tree line and 200 vertical meters below it. We also study rizospheric fungal community dynamics through the four seasons of one year. Methods involved ADN extraction from *c.* 160 soil samples and the rizospheric fungal community determination through metagenomics. The objective of this document is to describe and analyze in detail the resulting dataset weaving narrative, data handling, statistical analyses and visualizatuions in support to the man scientific article in prepartion.

3. Experimental design

Soil samples (5 composed samples per Location) were collected at two altitudes in four decidious *Nothofagus* forests along the Andes mountain range in Chile (36 to 45°S). Each sample combined soil collected at the base of five trees to enhance overall representation and reduce the variance due to fine-scale variation. The region/forests sampled were Antillanca, Chillán, Villarrica and Coyhaique. This sampling was repeated in spring, summer, fall and winter. Therefore the (expected) number of samples analyzed were 2 (altitudes) x 5 (replicas) x 4 (seasons) x 4 (regions) = 160. The DNA extraction and metabarcoding pipeline were described elsewhere, but essentially the internal transcribed spacer (ITS2), the most commonly sequenced genetic marker in mycology, was sequenced and each amplicon sequence variant (ASV) was subsequently matched to an existing database to assign a taxonomic hypothesis at Phylum, Order, and Family levels. Additionally, the fungi community was also analysed from a functional perspective by assigning fungal functional traits to ASVs.

4. Data importation

This document and analysis builds upon previous bioinformatic pipeline that resulted in a Phyloseq object containing all sample ASVs. This object, along with additional metadata were the basis of subsequent analyses.

4.1. Phyloseq object

```r
pseq2_filtered <- readRDS(file = "../Received_files/pseq2_filtered.rds") pseq2_filtered
```

```
## phyloseq-class experiment-level object
## otu_table()        OTU Table:          [ 5739 taxa and 137 samples ]
## sample_data() Sample Data:             [ 137 samples by 6 sample variables ] ##
tax_table()           Taxonomy Table:     [ 5739 taxa by 7 taxonomic ranks ]
```

```
# Reorder some factors
sample_data(pseq2_filtered)$Name <- factor(sample_data(pseq2_filtered)$Name, levels = c("CT", "CBT", "AT", "ABT",
    "VT", "VBT", "CHT", "CHBT"))
sample_data(pseq2_filtered)$Season <- factor(sample_data(pseq2_filtered)$Season, levels = c("Fall",
    "Winter", "Spring", "Summer"))
sample_data(pseq2_filtered)$Location <- factor(sample_data(pseq2_filtered)$Location, levels = c("Chillán",
    "Villarrica", "Antillanca", "Coyhaique"))
sample_data(pseq2_filtered)$Altitude <- factor(sample_data(pseq2_filtered)$Altitude, levels = c("Treeline",
    "Below treeline"))



new_names <- c(ABT = "Antillanca BT", AT = "Antillanca T", CBT = "Coyhaique BT",
    CT = "Coyhaique T", CHBT = "Chillán BT", CHT = "Chillán T", VBT = "Villarrica BT", VT = "Villarrica T")
```

The data comprise 137 successfully sequenced samples, collected in four zones both at the treeline and 200 m below the treeline, in each of the four seasons (Table 1).

Table 1: Breakdown of sample size indicating number of soil samples at Treeline/Below treeline.

	Fall	Winter	Spring	Summer	Subtotal	Total
Chillán	3/3	5/5	5/4	5/5	**18/17**	**35**
Villarrica	3/3	4/5	4/5	5/5	**16/18**	**34**
Antillanca	3/3	5/5	4/5	5/5	**17/18**	**35**
Coyhaique	3/0	5/5	5/5	5/5	**18/15**	**33**
**Subtotal**	**12/9**	**19/20**	**18/19**	**20/20**	**69/68**	**137**
**Total**	**21**	**39**	**37**	**40**	**137**	**137**

```
# Fix inconsistencies in SampleID that would prevent table joins
X <- data.frame(sample_data(pseq2_filtered))

# Modify the Sample_ID column
X <- X %>%
    mutate(Sample_ID = str_replace(Sample_ID, "_(.*)", ~tolower(.x))) rownames(X) <- X$Sample_ID
sample_data(pseq2_filtered) <- sample_data(X)
```

4.2. Sampling location coordinates

The coordinates in decimal degrees of sampling sites (Table 2) were incorporated to the Phyloseq object. Sampling took place in four Andean zones along a long latitudinal transect (Figure 1).

A closer inspection of satellite imagery, revealed various exposure and geographies that may have a role in the microbiome, beyond latitude. Unfortunately, replication was not sufficiently spread out in each sampling location to capture landscape-scale variability (Figure 2).

5. Number of reads per sample

The number of ASV reads per sample (sequencing depth) was variable, from 82 up to 200 thousand. After rerefying sample ASV richness as a function of sequencing depth, we decided to filter out 4 samples with <5000 reads. The resulting distribution of reads per sample profile is shown in Figure 3.

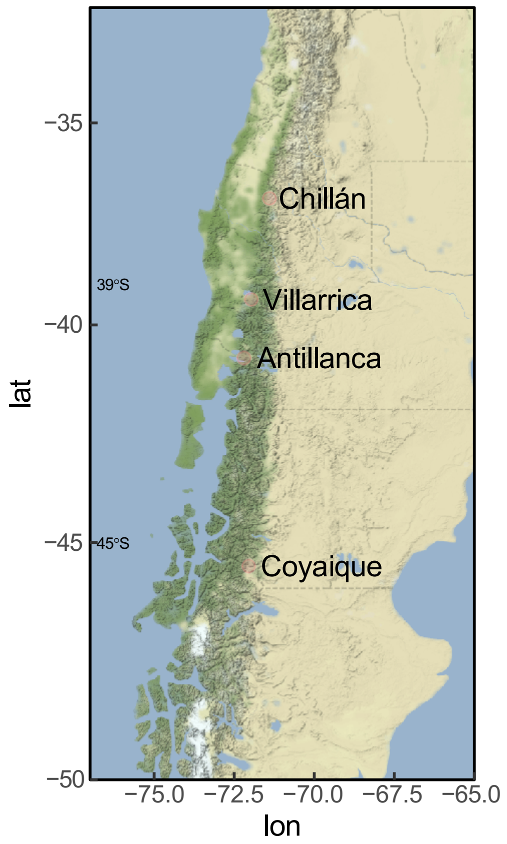

Figure 1: Distribution of sampling zones along the Andes mountain range. In each of these zones, replicated sampling was conducted at the treeline and 200 m bolow the treeline.

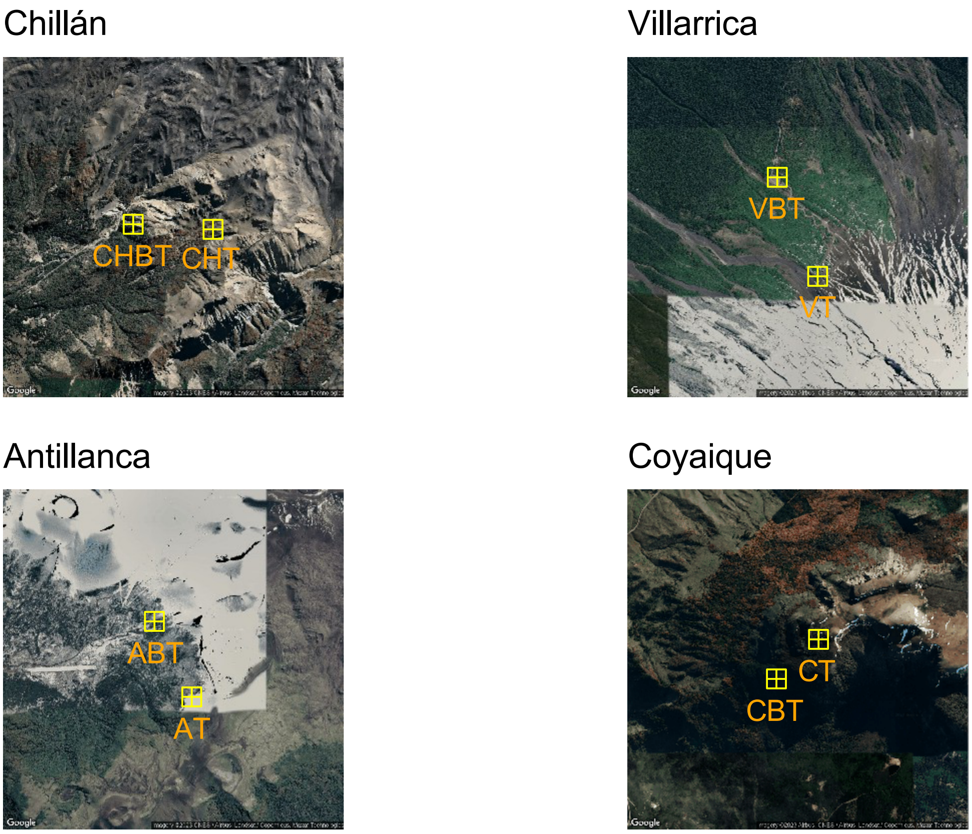

Figure 2: Sampling site locations at the threeline (codes ending in T) and 200 m below the treeline (ending in BT) in each zone. Three to five replicas were taken at each location and season.

Table 2: Geographic coordinates of sampling sites (Decial Degrees, WGS84).

Site	Name	Latitude	Longitude
Chillán	CHT	-36.90613	-71.39394
Chillán	CHBT	-36.90548	-71.40683
Villarrica	VT	-39.39359	-71.96410
Villarrica	VBT	-39.38123	-71.97063
Antillanca	AT	-40.78633	-72.19268
Antillanca	ABT	-40.77709	-72.19871
Coyaique	CT	-45.51626	-72.04409
Coyaique	CBT	-45.52073	-72.05089

```
otu_table <- phyloseq::otu_table(pseq2_filtered) otu_df <-
as.data.frame(as.matrix(otu_table))

read_abundance <- rowSums(otu_df)
read_abundance <- sort(read_abundance, decreasing = TRUE)

# -> plot
```

6. Number of reads per ASV

The abundance of the ASVs was highly variable as well, in the range 0, 3.7597×10^5 (Figure 4).

```
ASV_abundance <- colSums(otu_df)
ASV_abundance <- sort(ASV_abundance, decreasing = TRUE)
```

Warning: Transformation introduced infinite values in continuous y-axis ## Warning: Removed

14 rows containing non-finite values (`stat_align()`).

7. Alpha diversity

7.1. *Diversity profiling as a function of* q *(Hill's numbers)*

Hill numbers, expressed as *effective number* of species or types, provide a coherent statistical framework to assess biodiversity across multiple scales. Originally introduced by John H. Hill (1973), these numbers unify a wide array of biodiversity indices under a single mathematical formulation. Hill numbers are expressed in terms of order q, where q determines the sensitivity of the measure to (un)evenness in abundances (Jost, 2006). A $q = 0$ gives species richness irrespective of their relative abundances, $q = 1$ corresponds to the exponential of Shannon entropy, and $q = 2$ yields the inverse Simpson index that weighs heavily dominant species. Hence, by varying the value of q, one can weigh the importance of rare or abundant species in their analyses. The interpretation of *effective number* is straightforward: the number of equally abundant species/tapes that would be needed to give the same value of a diversity measure (Jost, 2006; Chao et al., 2014).

Next, we examine the alpha diversity of all samples, expressed as effective number of ASVs along a diversity-index continuum, from $q = 0$ (richness), through $q = 1$ (Shannon entropy) to $q = 2$ (Simpson-Gini concentration) hilldiv, an R package for the integral analysis of diversity based on Hill numbers (Alberdi 2019).

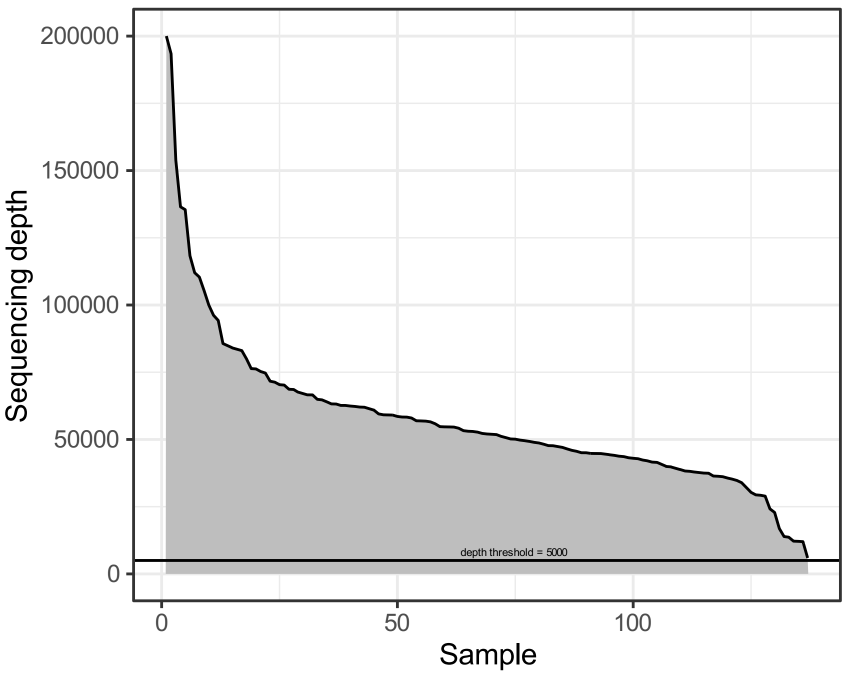

Figure 3: Number of reads obtained per sample (sequencing depth). Samples were sorted by decreasing sequencing depth, and those with <5000 reads were excluded from the analysis.

```
library(hilldiv)
```

```
## Registered S3 methods overwritten by 'FSA':
##      method          from
##      confint.boot car ##
         hist.boot       car

##
## Attaching package: 'hilldiv'

## The following object is masked from 'package:phyloseq':
##
##          merge_samples
```

```
# Extract and transpose ASV table
asv <- t(otu_table(pseq2_filtered))

# Calculate diversity profiles for each sample
div <- div_profile(count = asv, qvalues = seq(from = 0, to = 2, by = (0.1)))

# Calculate a global alpha diversity profile
div.global <- div_profile(count = asv, qvalues = seq(from = 0, to = 2, by = (0.1)), level = "alpha")
div.global <- data.frame(Div.order = as.numeric(names(div.global)), Effective.number = div.global)

# extra data wrangling -> plot
```

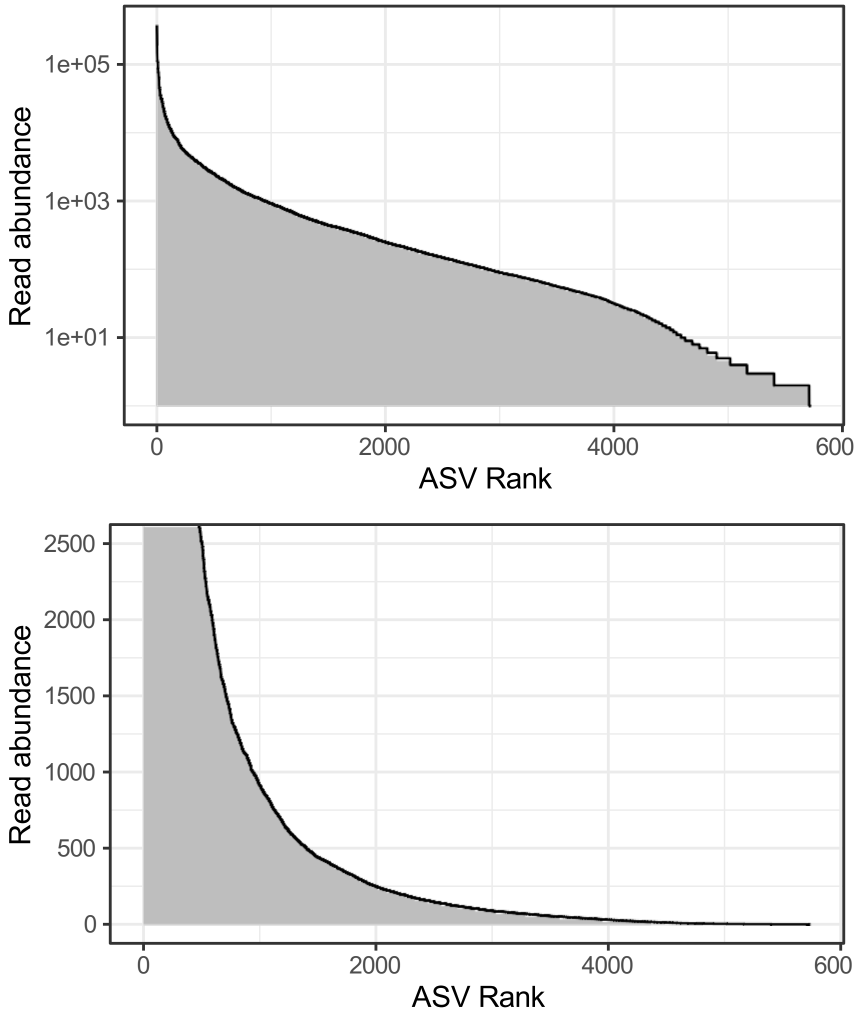

Figure 4: Total number of reads per ASV. Note the logarithmic scale in the y-axis of the top panel that shows the overall pattern, and the raw scale in the botom panel that zooms in ASVs with <2500 reads.

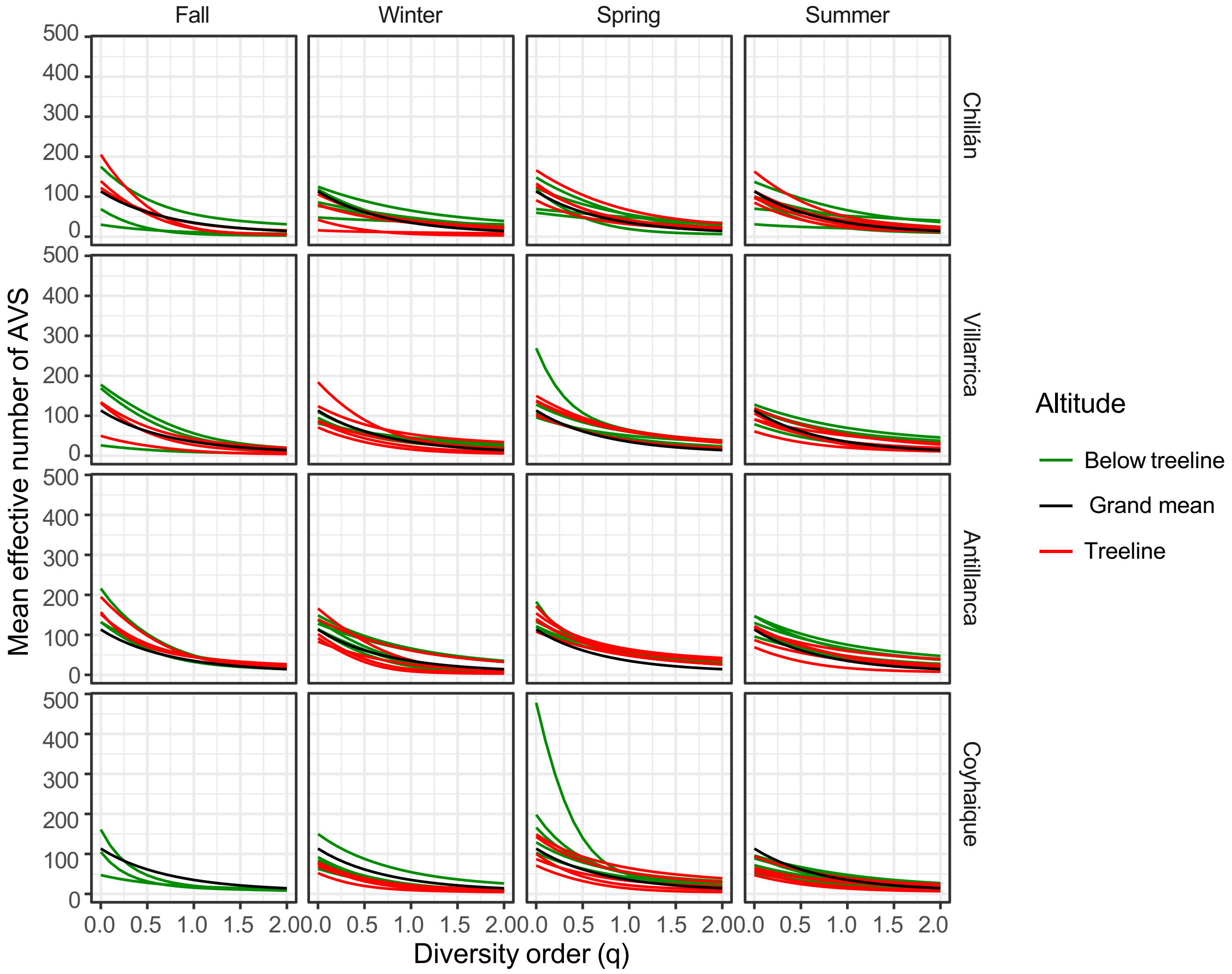

Figure 5: Alpha diversity profiles showing the effective number of ASVs (Hill's numbers) as a function of diversity order, q.

All diversity profiles showed negative slopes indicating heterogeneity in ASVs abundance within samples with relatively small numbers of dominating ASVs (Figure 5). This effect is quite extreme in samples with 200 or more ASVs (see richness at $q = 0$). As the diversity order increases, so does the weight of dominant species in the diversity formula thus causing a drop in the effective number of ASV values towards the right-hand side of all curves. It is hard to conclude from this figure if there is a statistically significant effect of *Location*, *Season* and *Altitude*, though by using the mean profile across all samples (black line) as a benchmark, it becomes apparent that the collection of profiles from some Locations and Seasons are offset (e.g., Antillanca in Spring, or Coyhaique in the Summer).

7.2. Observed richness (R)

Species richness (Hill number at $q = 0$ in the framework addressed above) can be studied from the observed number of taxa or estimated (extrapolated) number of taxa adding up both observed and unobserved (due to sampling limitations) number of taxa. For the latter, it is necessary to use untrimmed, non-normalized count data, as many of these estimates rely on the number of taxa present in only one (singletons) and only two sites (boubletons), or on rare taxa present at very low abundances. Of course, any surrogate for taxa can be used, including amplicon sequence variants (ASV).

Below, the observed number of amplicon sequence variants (ASV) is studied, R_{ASV}. The raw data can be seen in Figure 6.

The observed ASV richness, R_{ASV}, was calculated with the wrapper function estimate_richness{phyloseq} (underlying functions are from the vegan package), and subsequently the sample_data(pseq2_filtered) was updated with R.

```
R.obs <- estimate_richness(pseq2_filtered, measures = c("Observed")) %>% rownames_to_column(var =
     "Sample_ID")

R.obs <- full_join(data.frame(sample_data(pseq2_filtered)), R.obs, by = "Sample_ID") %>%
     column_to_rownames(var = "Sample_ID")

sample_data(pseq2_filtered) <- R.obs
```

7.2.1. Poisson regression on $R_{ASV.obs}$

Richness is count data taking only positive integers and hwnce a general linear model can be used. We begin by fitting a múltimple poisson regression (Eq. 1).

$$
\begin{aligned}
R_i &\sim \text{Poisson}(\mu_i),\\
E(R|\text{Treatment}) &= \mu,\\
\mu_i &= \exp(\eta_i),\\
\eta_i &= \beta_0 + \beta_1\text{Season}_i + \beta_2\text{Site}_i + \beta_3\text{Altitude}_i
\end{aligned}
\tag{1}
$$

```
m1 <- glm(Observed ~ Season + Altitude + Location + Season:Altitude + Altitude:Location, data = R.obs, family =
     poisson)

summary(m1)
```

```
##
## Call:
## glm(formula = Observed ~ Season + Altitude + Location + Season:Altitude + ##
       Altitude:Location, family = poisson, data = R.obs)
##
## Coefficients:
##                          Estimate Std. Error z value Pr(>|z|)
##  (Intercept)              4.91566    0.03371 145.816  < 2e-16
##  SeasonWinter            -0.38847    0.03701 -10.497  < 2e-16
##  SeasonSpring            -0.05237    0.03520  -1.488    0.137
##  SeasonSummer            -0.36285    0.03682  -9.855  < 2e-16
##  AltitudeBelow   treeline -0.37275   0.04812  -7.746 9.48e-15
##  LocationVillarrica      -0.01488    0.03236  -0.460    0.646
```

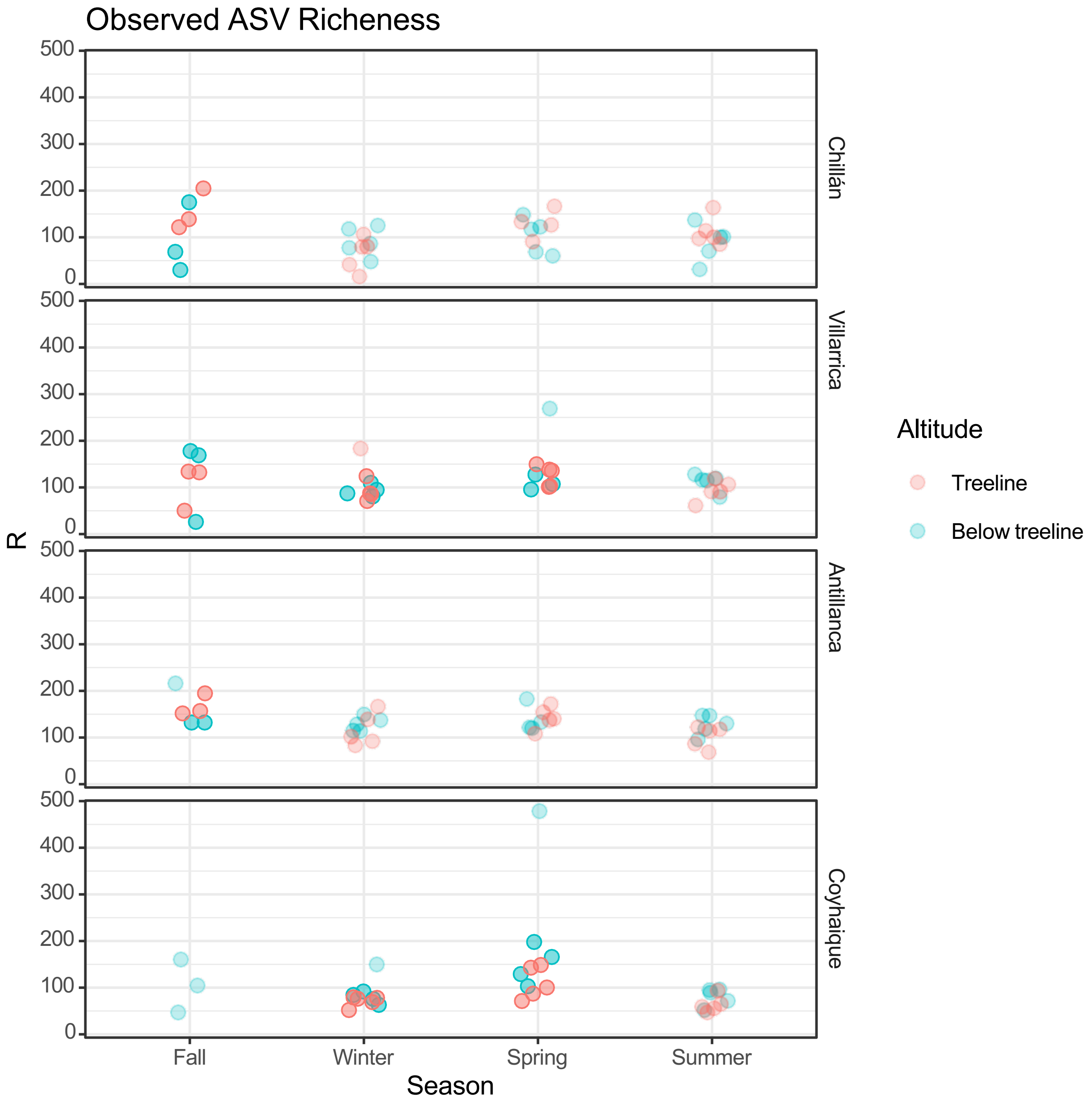

Figure 6: Observed ASV richness.

```
##  LocationAntillanca LocationCoyhaique                  0.14608    0.03117    4.687  2.77e-06
##  SeasonWinter:AltitudeBelowtreeline                   -0.25715    0.03744   -6.869  6.48e-12
##                                                        0.22467    0.05081    4.422  9.80e-06
##  SeasonSpring:AltitudeBelow  treeline                  0.30248    0.04795    6.309  2.81e-10
##  SeasonSummer:AltitudeBelow  treeline                  0.19935    0.05041    3.955  7.66e-05
##  AltitudeBelow treeline:LocationVillarrica             0.26454    0.04656    5.681  1.34e-08
##  AltitudeBelow treeline:LocationAntillanca             0.24887    0.04470    5.568  2.58e-08
##  AltitudeBelow treeline:LocationCoyhaique              0.55017    0.04939   11.140   < 2e-16
##
## (Intercept)                                ***
## SeasonWinter                               ***
## SeasonSpring
## SeasonSummer                               ***
## AltitudeBelow treeline                     ***
## LocationVillarrica
## LocationAntillanca                         ***
## LocationCoyhaique                          ***
##  SeasonWinter:AltitudeBelow   treeline          ***    ##
SeasonSpring:AltitudeBelow     treeline            ***     ##
SeasonSummer:AltitudeBelow   treeline              ***     ##
AltitudeBelow treeline:LocationVillarrica *** ## AltitudeBelow
treeline:LocationAntillanca       ***       ##       AltitudeBelow
treeline:LocationCoyhaique  *** ## ---
## Signif. codes:  0 '***' 0.001 '**' 0.01 '*' 0.05 '.' 0.1 ' ' 1 ##
## (Dispersion parameter for poisson family taken to be 1) ##
##       Null deviance: 2816.8  on 136  degrees of freedom ## Residual
deviance: 1980.1  on 123  degrees of freedom ## AIC: 2895.3
##
## Number of Fisher Scoring iterations: 4
```

```
(dispersion <- summary(m1)$deviance/summary(m1)$df.residual)
```

```
## [1] 16.09862
```

Before getting too excited about the high significance of most parameters, note that this model fit suggests substantial overdispersion as inferred from calculating the Dispersion statistic = 16.0986232 (Zuur *et al.* 2009, P224). Overdispersion can greatly inflate the rate of *Type I error*. Some of this overdispersion might be due to outliers, particularly CBT4_spring (Figure 6). Model misspecification may contribute as well. Next, we explored the effects of refitting the model after filtering out one outlier, and displayed the deviance residuals against all explanatory variables.

```
# Exclude offending outlier

R.obs.outlier <- R.obs %>% filter(rownames(.) !=
      "CBT4_spring")

# Refit
m1 <- glm(Observed ~ Season + Altitude + Location + Season:Altitude + Altitude:Location,
```

```r
      data = R.obs.outlier, family = poisson)

summary(m1)
```

```
##
## Call:
## glm(formula = Observed ~ Season + Altitude + Location + Season:Altitude + ##
            Altitude:Location, family = poisson, data = R.obs.outlier)
##
## Coefficients:
##                                               Estimate Std. Error z value Pr(>|z|)
##  (Intercept)                                   4.91566    0.03371  145.816   < 2e-16
##  SeasonWinter                                 -0.38847    0.03701  -10.497   < 2e-16
##  SeasonSpring                                 -0.05237    0.03520   -1.488   0.13677
##  SeasonSummer                                 -0.36285    0.03682   -9.855   < 2e-16
##  AltitudeBelow     treeline                   -0.32776    0.04809   -6.815  9.40e-12
##  LocationVillarrica                           -0.01488    0.03236   -0.460   0.64556
##  LocationAntillanca                            0.14608    0.03117    4.687  2.77e-06
##  LocationCoyhaique                            -0.25715    0.03744   -6.869  6.48e-12
##  SeasonWinter:AltitudeBelow  treeline          0.22687    0.05081    4.465  8.01e-06
##  SeasonSpring:AltitudeBelow  treeline          0.16990    0.04873    3.486   0.00049
##  SeasonSummer:AltitudeBelow  treeline          0.19935    0.05041    3.955  7.66e-05
##  AltitudeBelow treeline:LocationVillarrica     0.26032    0.04656    5.591  2.26e-08
##  AltitudeBelow treeline:LocationAntillanca     0.24242    0.04470    5.423  5.86e-08
##  AltitudeBelow treeline:LocationCoyhaique      0.38000    0.05059    7.511  5.86e-14
##
## (Intercept)                                   ***
## SeasonWinter                                  ***
## SeasonSpring
## SeasonSummer                                  ***
## AltitudeBelow treeline                        ***
## LocationVillarrica
## LocationAntillanca                            ***
## LocationCoyhaique                             ***
##  SeasonWinter:AltitudeBelow   treeline              ***   ##
SeasonSpring:AltitudeBelow    treeline              ***    ##
SeasonSummer:AltitudeBelow   treeline               ***    ##
AltitudeBelow treeline:LocationVillarrica *** ## AltitudeBelow
treeline:LocationAntillanca       ***       ##      AltitudeBelow
treeline:LocationCoyhaique  *** ## ---
## Signif. codes:  0 '***' 0.001 '**' 0.01 '*' 0.05 '.' 0.1 ' ' 1 ##
## (Dispersion parameter for poisson family taken to be 1) ##
##       Null deviance: 2161.2  on 135  degrees of freedom ## Residual
deviance: 1497.5  on 122  degrees of freedom ## AIC: 2404.6
##
## Number of Fisher Scoring iterations: 4
```

```
(dispersion <- summary(m1)$deviance/summary(m1)$df.residual)
```

```
## [1] 12.27431
```

The Dispersion statistic = 12.27 indeed decreased, but it is still substantially overdispersed. Further- more, standard diagnostic plots including Pearson's residuals and residuls vs. leverage plots also suggested overdispersion and a handful of particularly influential datapoints beyond the outlier already excluded (not shown).

An intuitive way for GLM model validation is through inspection of simulation-based residuals (Gelman and Hill 2006, P. 155). We performed predictive simulations using the fitted model to generate 1000 data sets, which provided a probabilistic benchmark to compare with observed values in the original data set. Hence, simulation-based residuals in the range [0,1] were calculated, 0.5 meaning half of the simulated values lie above and half below the observed value (excellent fit). The residuals were visualized and variously tested using the DHARMa R package (Hartig 2022).

```
library(DHARMa)
```

##

```
This is DHARMa 0.4.6. For overview type '?DHARMa'. For recent changes, type news(package = 'DHARMa
```

```
# citation('DHARMa')

simulationOutput <- simulateResiduals(fittedModel = m1, plot = F, n = 1000)
```

```
# plot(simulationOutput)
par(mfrow = c(2, 1))
plotQQunif(simulationOutput) # left plot in plot.DHARMa()
plotResiduals(simulationOutput) # right plot in plot.DHARMa()
```

The results show a significant departure from the expected distribution of residuals (see Kolmogrov- Smirnov test, KS), as well as heavy overdispersion with many observed values lying beyond the simulation envelope, and an observed interquartil range wider than the expected interquartil range over the whole range of model predictions (Figure 7). Since any biological conclusion was compromised, we moved on to incorporate overdispersion into our GLM model though quasi-Poisson (you can skip this subsection) and negative binomial GLM (preferred).

7.2.2. Quasi-Piosson regression on $R_{ASV.obs}$

Next we attempted to address overdispersion by using a Poisson GLM model with an additional scale factor such that the variance is given by $\theta \times \mu$, where μ is the mean and θ the dispersion parameter. This so called quasi-Poisson GLM allows the variance to scale faster than the mean. Again, we removed the same outlier as above.

```
# Exclude offending outlier

R.obs.outlier <- R.obs %>% filter(rownames(.) !=
    "CBT4_spring")

m2 <- glm(Observed ~ Season + Altitude + Location + Season:Altitude + Altitude:Location, data = R.obs.outlier,
    family = quasipoisson)

summary(m2)
```

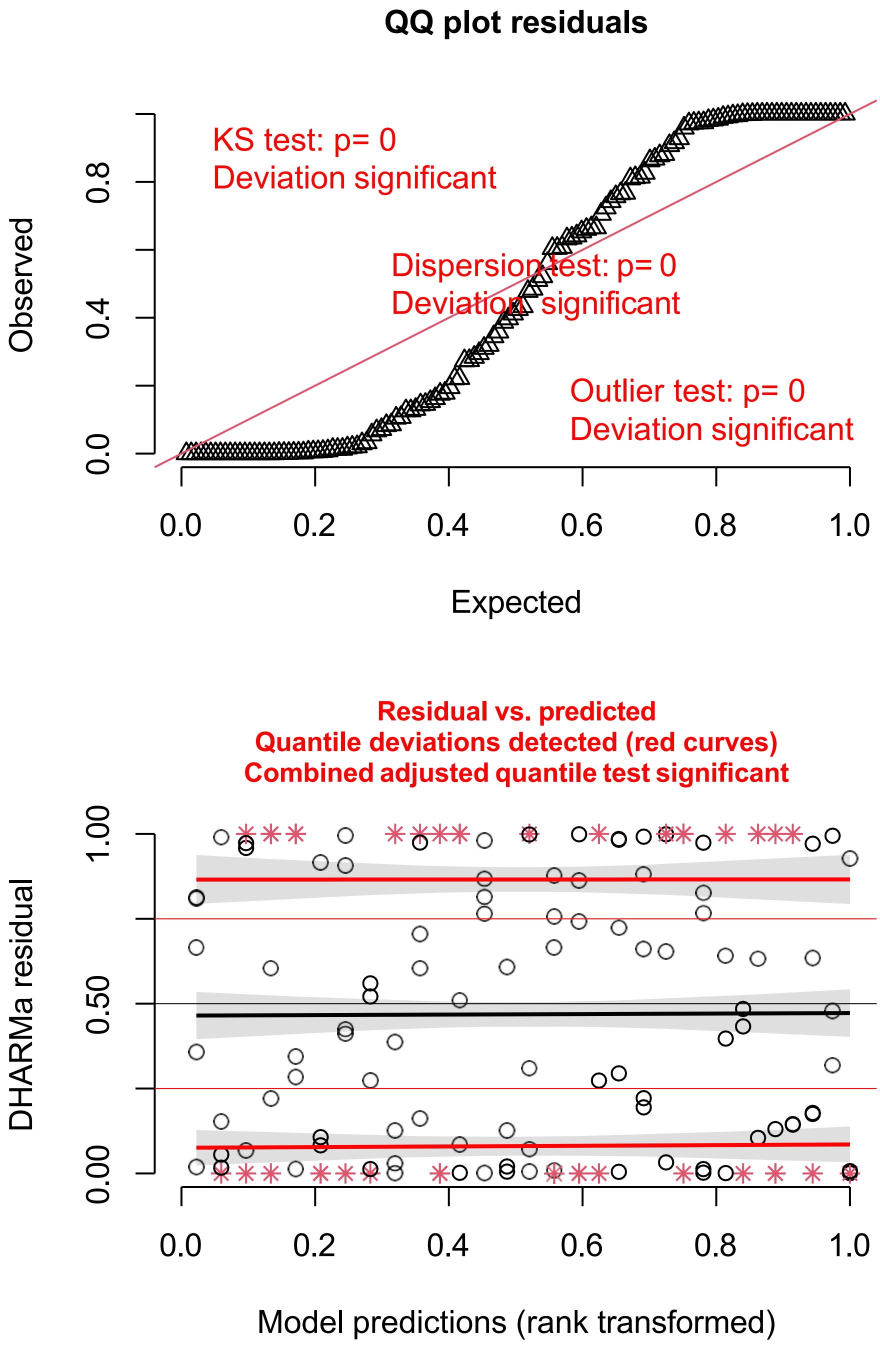

Figure 7: Simulation-based residual plots and diagnostic tests por the Poisson GLM on observed richness.

```
##
## Call:
## glm(formula = Observed ~ Season + Altitude + Location + Season:Altitude + ##
            Altitude:Location, family = quasipoisson, data = R.obs.outlier)
##
## Coefficients:
##                                          Estimate Std. Error t value Pr(>|t|)
## (Intercept)                               4.91566    0.11619  42.308  < 2e-16
## SeasonWinter                             -0.38847    0.12755  -3.046  0.00285
## SeasonSpring                             -0.05237    0.12131  -0.432  0.66671
## SeasonSummer                             -0.36285    0.12690  -2.859  0.00500
## AltitudeBelow      treeline              -0.32776    0.16575  -1.977  0.05025
## LocationVillarrica                       -0.01488    0.11152  -0.133  0.89406
## LocationAntillanca                        0.14608    0.10741   1.360  0.17634
## LocationCoyhaique                        -0.25715    0.12903  -1.993  0.04850
## SeasonWinter:AltitudeBelow  treeline      0.22687    0.17513   1.295  0.19761
## SeasonSpring:AltitudeBelow  treeline      0.16990    0.16797   1.012  0.31376
## SeasonSummer:AltitudeBelow  treeline      0.19935    0.17373   1.147  0.25343
## AltitudeBelow treeline:LocationVillarrica 0.26032    0.16049   1.622  0.10737
## AltitudeBelow treeline:LocationAntillanca 0.24242    0.15406   1.574  0.11819
## AltitudeBelow treeline:LocationCoyhaique  0.38000    0.17437   2.179  0.03123
##
## (Intercept)                              ***
## SeasonWinter                             **
## SeasonSpring
## SeasonSummer                             **
## AltitudeBelow treeline                   .
## LocationVillarrica
## LocationAntillanca
## LocationCoyhaique                        *
## SeasonWinter:AltitudeBelow  treeline
## SeasonSpring:AltitudeBelow  treeline
## SeasonSummer:AltitudeBelow  treeline
## AltitudeBelow treeline:LocationVillarrica
## AltitudeBelow treeline:LocationAntillanca
##  AltitudeBelow treeline:LocationCoyhaique *
## ---
## Signif. codes:  0 '***' 0.001 '**' 0.01 '*' 0.05 '.' 0.1 ' ' 1
##
## (Dispersion parameter for quasipoisson family taken to be 11.8786)
##
##     Null deviance: 2161.2  on 135  degrees of freedom
## Residual deviance: 1497.5  on 122  degrees of freedom
## AIC: NA
##
## Number of Fisher Scoring iterations: 4
```

```
(dispersion <- summary(m2)$deviance/summary(m2)$df.residual)
```

```
## [1] 12.27431
```

As expected, by incorporating the dispersion parameter θ in the model, less parameters were significant

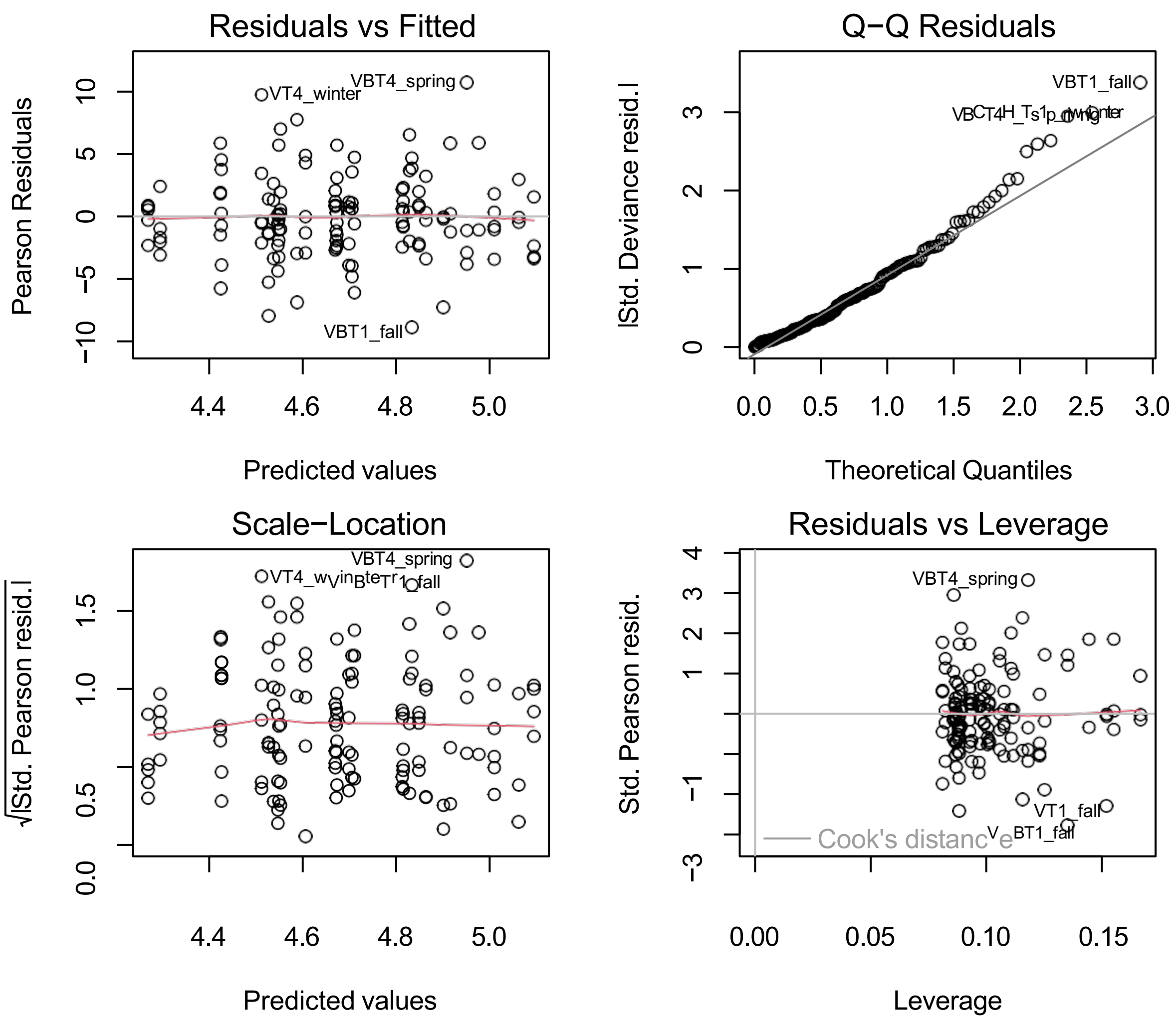

Figure 8: Standard diagnostic plots of quasi-Poisson múltiple regression after removal of one outlier.

(due to wider confidence intervals). Estimated θ = 11.9, similar to our calculated value of 12.3. Scaled Pearson's residuals, residuals vs. leverage, and other residual plots behaved much better, with less signs of overdispersion and no overly influential points (Figures 8, 9 and 10). Simulation-based residuals diagnostics, likelihood-based methods were not available for *quasi*-GLMs, which is a drawback.

7.2.3. *Negative binomial GLM*

An alternative GLM potentially suitable uses the negative binomial distribution.

```
# Negative binomial fit
m3 <- MASS::glm.nb(Observed ~ Altitude + Location + Season + Season:Altitude + Altitude:Location, link = log, data =
     R.obs.outlier)

summary(m3, correlation = F) ##
## Call:
## MASS::glm.nb(formula = Observed ~ Altitude + Location + Season + ##
       Season:Altitude + Altitude:Location, data = R.obs.outlier,
```

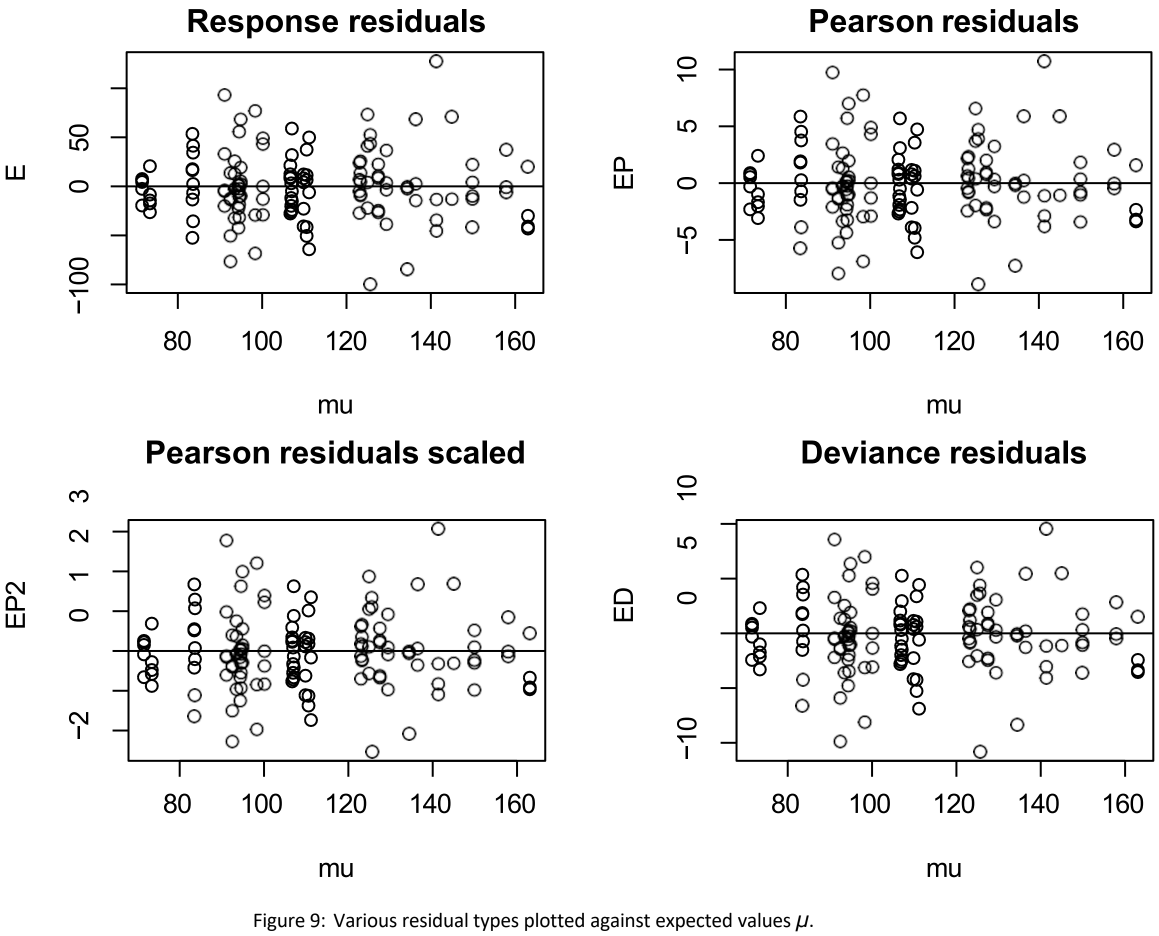

Figure 9: Various residual types plotted against expected values μ.

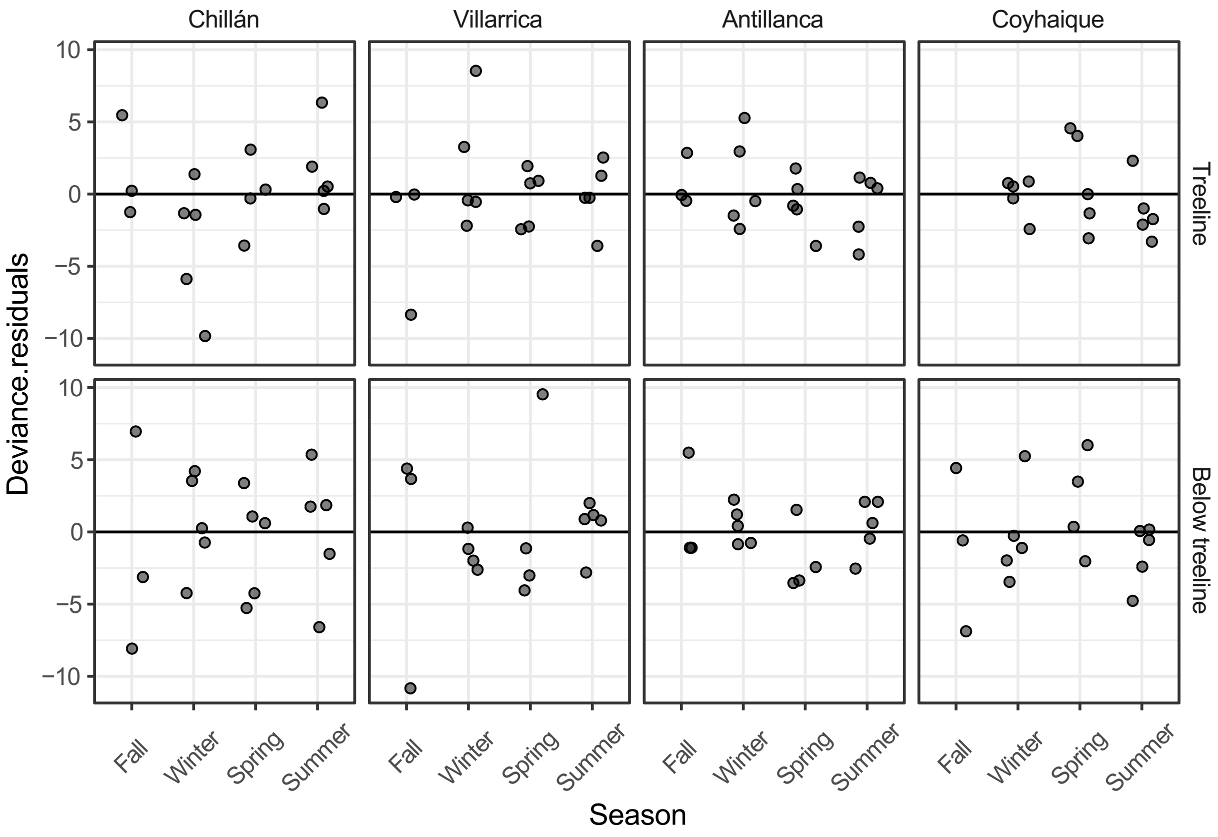

Figure 10: Deviance residuals plotted against predictor variables.

```
##          link = log, init.theta = 9.886254763) ##
## Coefficients:
##                                                   Estimate Std. Error z value Pr(>|z|)
## (Intercept)                                        4.89959    0.12764  38.386  < 2e-16
## AltitudeBelow treeline                            -0.31351    0.17385  -1.803  0.07133
## LocationVillarrica                                 0.01349    0.11250   0.120  0.90458
## LocationAntillanca                                 0.15845    0.11219   1.412  0.15786
## LocationCoyhaique                                 -0.25827    0.12075  -2.139  0.03245
## SeasonWinter                                      -0.38692    0.13508  -2.864  0.00418
## SeasonSpring                                      -0.03909    0.13585  -0.288  0.77356
## SeasonSummer                                      -0.36026    0.13502  -2.668  0.00763
## AltitudeBelow treeline:SeasonWinter                0.23631    0.18235   1.296  0.19502
## AltitudeBelow treeline:SeasonSpring                0.17219    0.18443   0.934  0.35050
## AltitudeBelow treeline:SeasonSummer                0.20290    0.18148   1.118  0.26355
## AltitudeBelow treeline:LocationVillarrica          0.22092    0.16047   1.377  0.16861
## AltitudeBelow treeline:LocationAntillanca          0.23078    0.15876   1.454  0.14604
## AltitudeBelow treeline:LocationCoyhaique           0.36199    0.16535   2.189  0.02858
##
## (Intercept)                                       ***
## AltitudeBelow treeline                            .
## LocationVillarrica
## LocationAntillanca
## LocationCoyhaique                                 *
## SeasonWinter                                      **
## SeasonSpring
## SeasonSummer                                      **
## AltitudeBelow treeline:SeasonWinter
## AltitudeBelow treeline:SeasonSpring
## AltitudeBelow treeline:SeasonSummer
## AltitudeBelow treeline:LocationVillarrica
## AltitudeBelow treeline:LocationAntillanca
## AltitudeBelow treeline:LocationCoyhaique   *
## ---
## Signif. codes:  0 '***' 0.001 '**' 0.01 '*' 0.05 '.' 0.1 ' ' 1
##
## (Dispersion parameter for Negative Binomial(9.8863) family taken to be                1)
##
##        Null deviance: 196.03  on 135  degrees of freedom
## Residual deviance: 140.34  on 122  degrees of freedom
## AIC: 1382.7
##
## Number of Fisher Scoring iterations: 1
##
##
##                      Theta:  9.89
##                  Std. Err.:  1.32
##
##   2 x log-likelihood:  -1352.737
```

```
(dispersion <- summary(m3)$deviance/summary(m3)$df.residual)
```

```
## [1] 1.150369
```

Interestingly, this model shows a *Dispersion statistic* = 1.2, which solves the overdispersion problem.

Furthermore the simulation-based diagnostics shows a satisfying fit, with observations conforming to expected simulated values and residual quartiles uniformly distributed and corresponding to the simulated quartiles (Figure 8).

```
simulationOutput <- simulateResiduals(fittedModel = m3, plot = F, n = 1000)

# plot(simulationOutput)
par(mfrow = c(2, 1))
plotQQunif(simulationOutput)  # left plot in plot.DHARMa()
plotResiduals(simulationOutput)  # right plot in plot.DHARMa()
```

Also, as expected for a good model fit, simulation-based residuals were centered in 0.5 and were homogeneously distributed among sampling groups (Figure 9). Overall, we considered the negative binomial GLM model adequate for the observed richness data, and we moved on to examine

```
par(mfrow = c(3, 1), cex.main = 0.5) plotResiduals(simulationOutput, form =
R.obs.outlier$Altitude) plotResiduals(simulationOutput, form =
R.obs.outlier$Location) plotResiduals(simulationOutput, form = R.obs.outlier$Season)
```

Next, the significance of each predictor variable (and interaction terms) was evaluated by excluding one term of the global model at a time and comparing the reduced model to the global model trough likelihood ratio test (drop1{stats} function).

```
LRT <- drop1(m3, test = "Chi", scope = . ~ .)
```

Table 3: Likelihood ratio tests (LRT) for individual terms of the negative binomial model of observed richness. The first row represents the full model.

	Df	Deviance	AIC	LRT	Pr(>Chi)
<none>	NA	140.3450	1380.737	NA	NA
Altitude	1	143.6500	1382.042	3.305028	0.0690682
Location	3	152.5198	1386.911	12.174845	0.0068076
Season	3	158.6220	1393.014	18.277046	0.0003856
Altitude:Season	3	142.1513	1376.543	1.806348	0.6135545
Altitude:Location	3	145.3364	1379.728	4.991450	0.1724243

Based on the results of likelihood ratio tests excluding one term at a time from the global negative binomial GLM model (Table 3), we decided to drop the two interaction terms and refit the model.

7.2.4. Negative binomial GLM, only main effects

```
# Negative binomial fit
m4 <- MASS::glm.nb(Observed ~ Altitude + Location + Season, link = log, data = R.obs.outlier)

summary(m4, correlation = F)
```

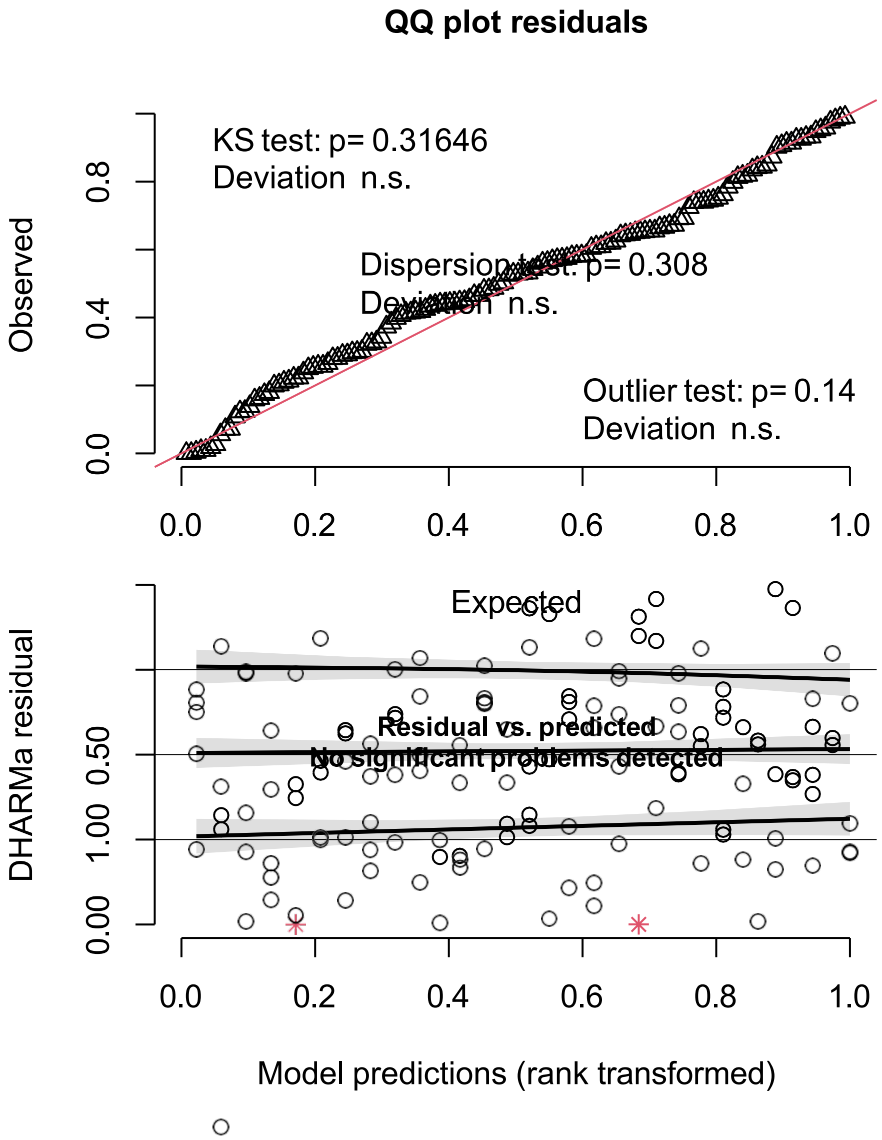

Figure 11: (#fig:sim.residuals2)Simulation-based residual plots and diagnostic tests por the negative binomial GLM on observed richness.

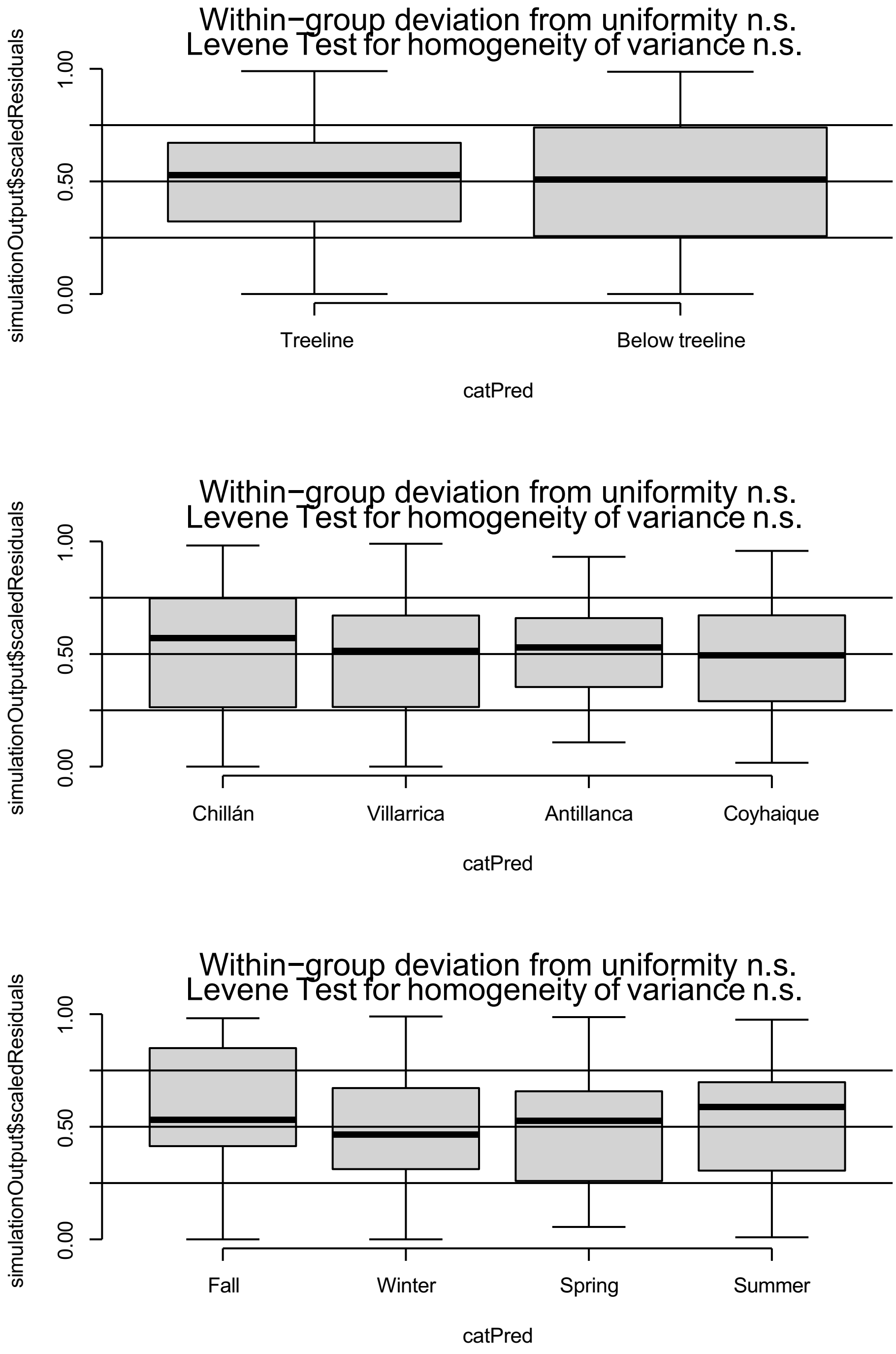

Figure 12: Simulation-based residual plots of negative binomial GLM in relation to predictive variables.

```
##
## Call:
## MASS::glm.nb(formula = Observed ~ Altitude + Location + Season, ##          data =
R.obs.outlier, link = log, init.theta = 9.327200853) ##
## Coefficients:
##                              Estimate Std. Error z value Pr(>|z|)
##  (Intercept)                  4.72662    0.09499  49.760  < 2e-16 ***
##  AltitudeBelow treeline       0.05814    0.05876   0.989 0.322434
##  LocationVillarrica           0.11512    0.08231   1.399 0.161929
##  LocationAntillanca           0.26858    0.08146   3.297 0.000977 ***
##  LocationCoyhaique           -0.08006    0.08418  -0.951 0.341580
##  SeasonWinter                -0.28050    0.09268  -3.027 0.002473 **
##  SeasonSpring                 0.03640    0.09361   0.389 0.697386
##  SeasonSummer                -0.26354    0.09215  -2.860 0.004240 **
##  ---
##  Signif. codes:  0 '***' 0.001 '**' 0.01 '*' 0.05 '.' 0.1 ' ' 1
##
##  (Dispersion parameter for Negative Binomial(9.3272) family taken          to be 1)
##
##      Null deviance: 186.01  on 135  degrees of freedom
##  Residual deviance: 140.23  on 128  degrees of freedom
##  AIC: 1377.9
##
##  Number of Fisher Scoring iterations: 1
##
##
##                Theta:  9.33
##            Std. Err.:  1.23
##
##   2 x log-likelihood:  -1359.944
```

```
(dispersion <- summary(m4)$deviance/summary(m4)$df.residual)
```

```
## [1] 1.095576
```

Dispersion statistic = 1.1, which suggests no problem. The simulation-based diagnostics also reveal no problem (Figure 10).

```
simulationOutput <- simulateResiduals(fittedModel = m4, plot = F, n = 1000)

# plot(simulationOutput)
par(mfrow = c(2, 1))
plotQQunif(simulationOutput)  # left plot in plot.DHARMa()
plotResiduals(simulationOutput)  # right plot in plot.DHARMa()
```

Also, as expected for a good model fit, simulation-based residuals were centered in 0.5 and were homogeneously distributed among sampling groups (Figure 11).

```
par(mfrow = c(3, 1), cex.main = 0.5) plotResiduals(simulationOutput, form =
R.obs.outlier$Altitude) plotResiduals(simulationOutput, form =
R.obs.outlier$Location) plotResiduals(simulationOutput, form = R.obs.outlier$Season)
```

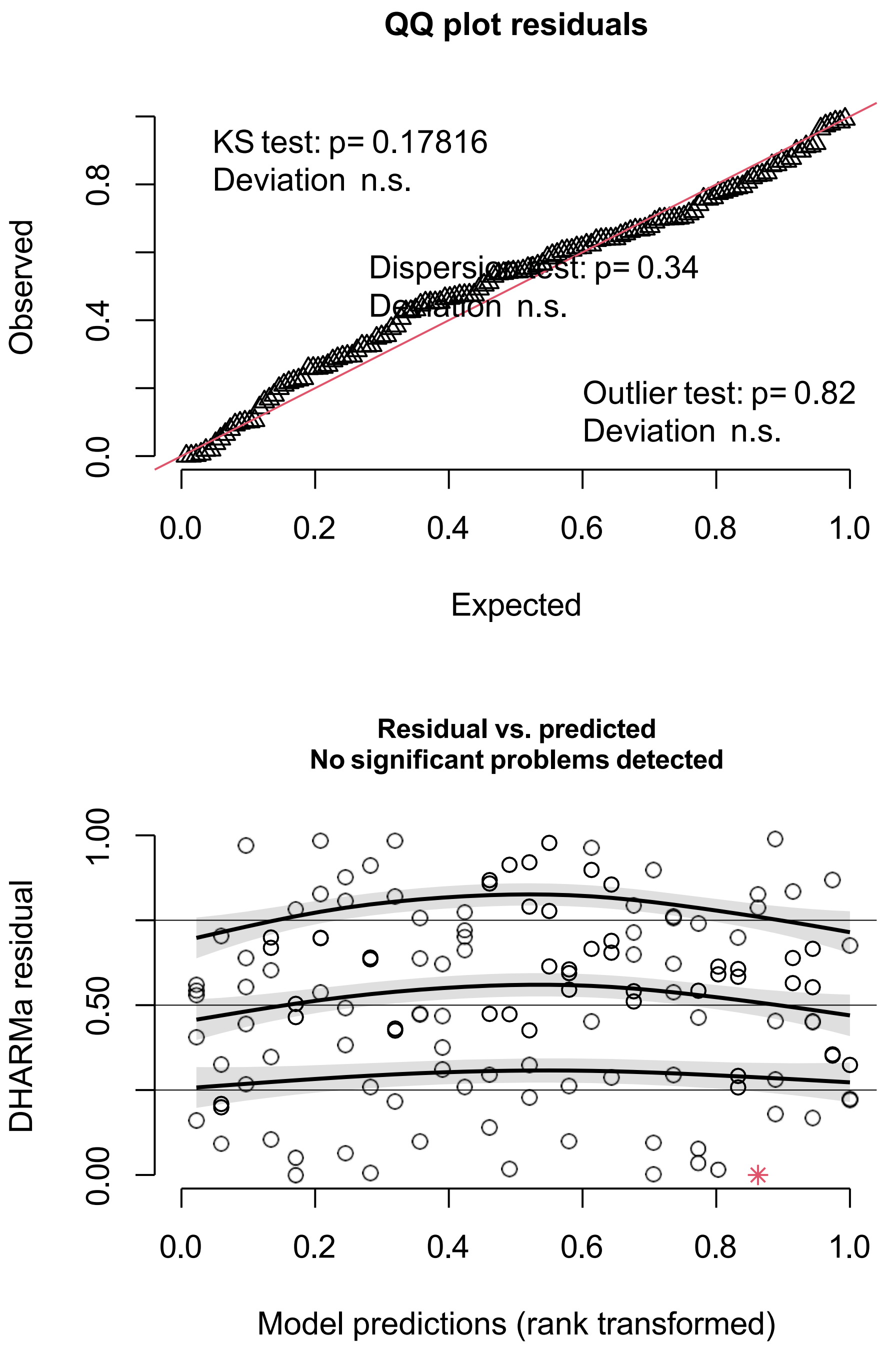

Figure 13: (#fig:sim.residuals3)Simulation-based residual plots and diagnostic tests por the negative binomial GLM on observed richness, excluding interactions.

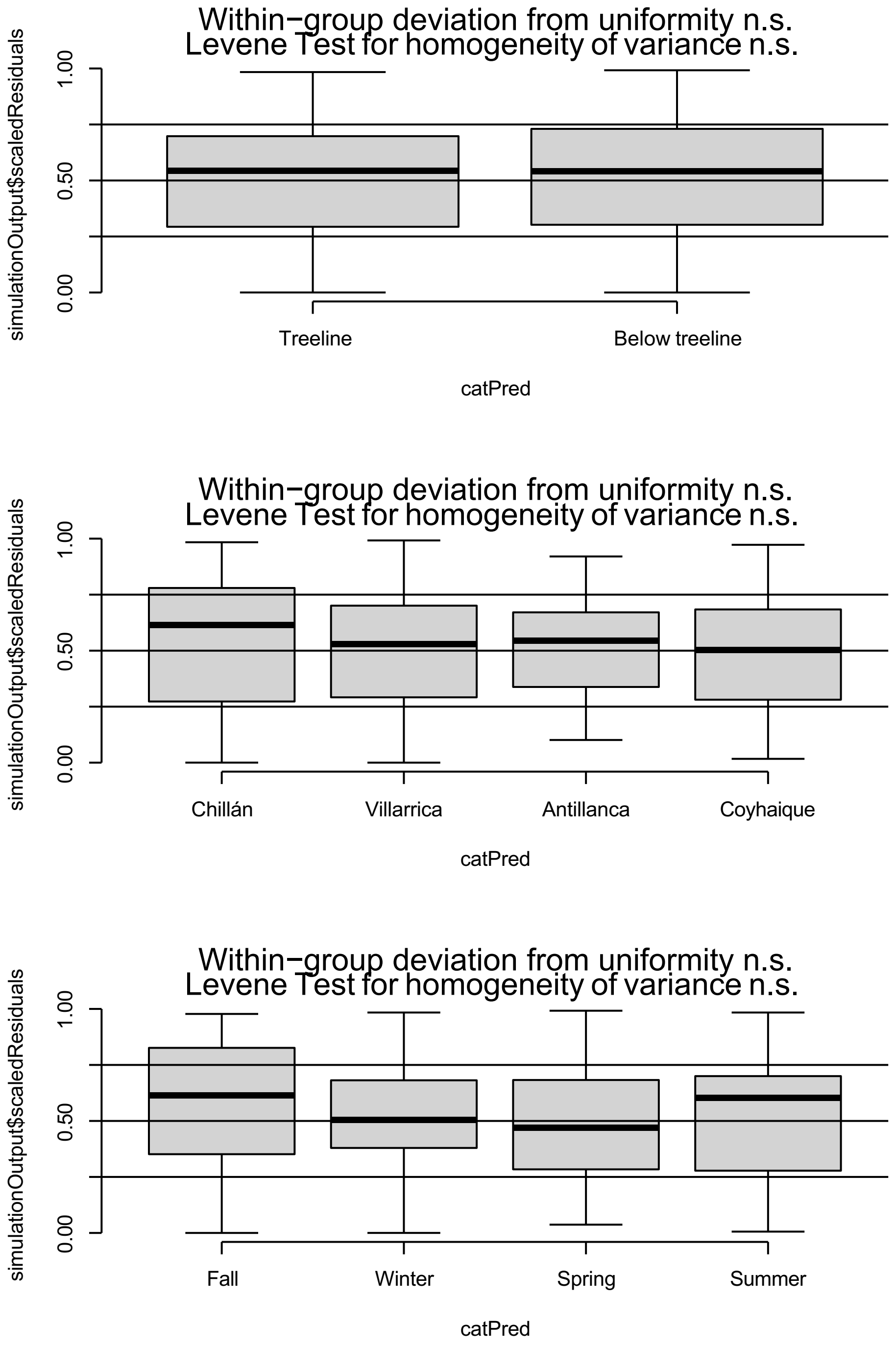

Figure 14: Simulation-based residual plots of negative binomial GLM in relation to predictive variables.

The statistical significance of remaining model terms was again evaluated with the drop1{stats}function.

```
LRT <- drop1(m4, test = "Chi", scope = . ~ .)
```

```
LRT <- as.data.frame(LRT)

kable(LRT, caption = "Likelihood ratio tests (LRT) for the main effects of the negative binomial GLM
    kable_styling(bootstrap_options = c("striped", "hover"), full_width = F, latex_options = c("hold_
```

Table 4: Likelihood ratio tests (LRT) for the main effects of the negative binomial GLM of observed richness. The first row represents the full model, which now did not include interactions.

	Df	Deviance	AIC	LRT	Pr(>Chi)
<none>	NA	140.2337	1375.944	NA	NA
Altitude	1	141.2114	1374.922	0.9776757	0.3227733
Location	3	160.3214	1390.032	20.0876649	0.0001628
Season	3	165.4984	1395.209	25.2646547	0.0000136

```
# %>% column_spec(6:7, background = 'lightgray', bold = T) %>% column_spec(2:7, # width = '1.5cm')
%>% row_spec(5:6, background = 'lightgray', bold = T)
```

While the effect of *Season* and *Location* were highly significant (*P* < 0.001), the effect of *Altitude* was not statistically significant (P = 0.323) (Table 4). The same result is confirmed with backward selection based on AIC ctireria, as implemented in function step{stats}.

Therefore, the best model can be written as follows:

MASS::glm.nb(formula = Observed ~ Location + Season, data = R.obs.outlier, init.theta = 9.256615304, link = log)

7.2.5. Negtive binomial GLM, final model

```
# Negative binomial fit
m5 <- MASS::glm.nb(formula = Observed ~ Location + Season, data = R.obs.outlier, init.theta = 9, link = log)

summary(m5, correlation = F)
```

```
##
## Call:
## MASS::glm.nb(formula = Observed ~ Location + Season, data = R.obs.outlier, ##       init.theta =
9.256614718, link = log)
##
## Coefficients:
##                     Estimate Std. Error z value Pr(>|z|)
## (Intercept)          4.75614    0.08877  53.578  < 2e-16 ***
## LocationVillarrica   0.11567    0.08256   1.401 0.161202
## LocationAntillanca   0.26997    0.08173   3.303 0.000956 ***
## LocationCoyhaique   -0.07374    0.08446  -0.873 0.382611
## SeasonWinter        -0.28245    0.09286  -3.042 0.002352 **
## SeasonSpring         0.03266    0.09375   0.348 0.727545
```

```
## SeasonSummer       -0.26510     0.09238  -2.870 0.004108 **
## ---
## Signif. codes:  0 '***' 0.001 '**' 0.01 '*' 0.05 '.' 0.1 ' ' 1
##
## (Dispersion parameter for Negative Binomial(9.2566) family taken to be 1)
##
##     Null deviance: 184.74  on 135  degrees of freedom
## Residual deviance: 140.25  on 129  degrees of freedom
## AIC: 1376.9
##
## Number of Fisher Scoring iterations: 1
##
##
##               Theta:  9.26
##           Std. Err.:  1.22
##
##  2 x log-likelihood:  -1360.919
```

```
(dispersion <- summary(m5)$deviance/summary(m5)$df.residual)
```

```
## [1] 1.087206
```

Dispersion statistic = 1.1, which suggests no problem. The simulation-based diagnostics also reveal no problem (not shown).

Also, as expected for a good model fit, simulation-based residuals were centered in 0.5 and were homogeneously distributed among sampling groups (Figure 11).

The statistical significance of remaining model terms was again evaluated with the drop1{stats} function, and, as expected, the remaining two terms (*Location* and *Season*) were highly signifficant (Table 5). The fitted model passed all the above tests for model diagnostics and validation (not shown).

```
LRT <- drop1(m5, test = "Chi", scope = . ~ .)
```

Table 5: Likelihood ratio tests (LRT) for the main effects of the negative binomial GLM of observed richness. The first row represents the full model, which now did not include interactions.

	Df	Deviance	AIC	LRT	Pr(>Chi)
<none>	NA	140.2496	1374.919	NA	NA
Location	3	159.8843	1388.554	19.63469	0.0002021
Season	3	165.2094	1393.879	24.95984	0.0000157

7.2.6. *Illustration of results*

There are a number of options to illustrate the results. One option is to simply plot parameter estimates, and whether they're significantly different from zero. This can be a bit frustrating because for factors there is a reference factor level which in this case was arbitrarily set to Chillán and Spring. The reference level is set to zero in the model and is represented by the intercept (Figure 15). This method is better suited for experiments with control groups.

A better option is to show the model fit in the scale of the response, i.e., number of ASVs. We illustrated the model fit and 95% predictive confidence intervals for the full experimental design, including the original data in the graph.

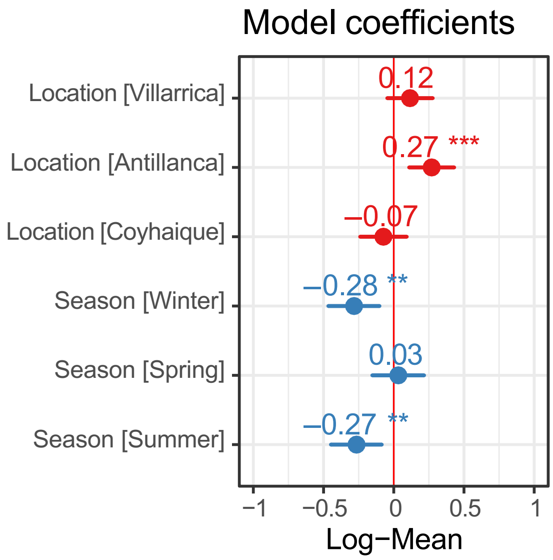

Figure 15: Model coefficients of reduced negative binomial GLM. Note these are in the link scale.

```
newdata <- expand.grid(Location = unique(R.obs.outlier$Location), Season = unique(R.obs.outlier$Seaso preds <- predict(m5,

newdata, se.fit = TRUE, dispersion = m5$theta)
newdata$fit <- exp(preds$fit)
newdata$upper <- exp(preds$fit + 1.96 * preds$se.fit) newdata$lower <-
exp(preds$fit - 1.96 * preds$se.fit)
```

The results showed a very high within-sample variability which was exacerbated at high expected ASV richness. Nevertheless, some temporal and spatial patterns did emerge: ASV richness was higher in spring and fall than in winter and summer, and Antillanca exhibited the highest diversity (Figure 16). As mentioned earlier, we found no significant effect of *Altitude* (treeline or below treeline) nor of the interaction between *Season* and *Location*. If there was an effect, overdispersion probably hindered it.

7.3. Shannon entropy and its effective numbers

Other alpha diversity assessments should incorporate ASVs's relative abundances.
Here we analyzed the *effective number of ASVs* at q = 1, related to the Shannon entropy index, and q = 4, related to the Simpso-Gin concentration (see above *Diversity profiling as a function of q*). We begin by effective numbers at *q* = 1. It is interesting to note, however, the difference between the frequency distribution of the Shannon index itself and the corresponding Hill numbers (Figure 17). Effective species numbers are easier to interpret and show desirable statistical properties.

We calculated the Shannon entropy index and associated effective numbers (i.e., Hill numbers with q = 2, see above *Diversity profiling as a function of q*).

```
# Calculate Shannon index
H.obs <- estimate_richness(pseq2_filtered, measures = c("Shannon")) %>% rownames_to_column(var =
    "Sample_ID")

# Calculate effective numbers (Hill numbers, q = 1)
Hill.1 <- data.frame(Hill.1 = hilldiv::hill_div(t(otu_df), qvalue = 1)) %>% rownames_to_column(var =
    "Sample_ID")
```

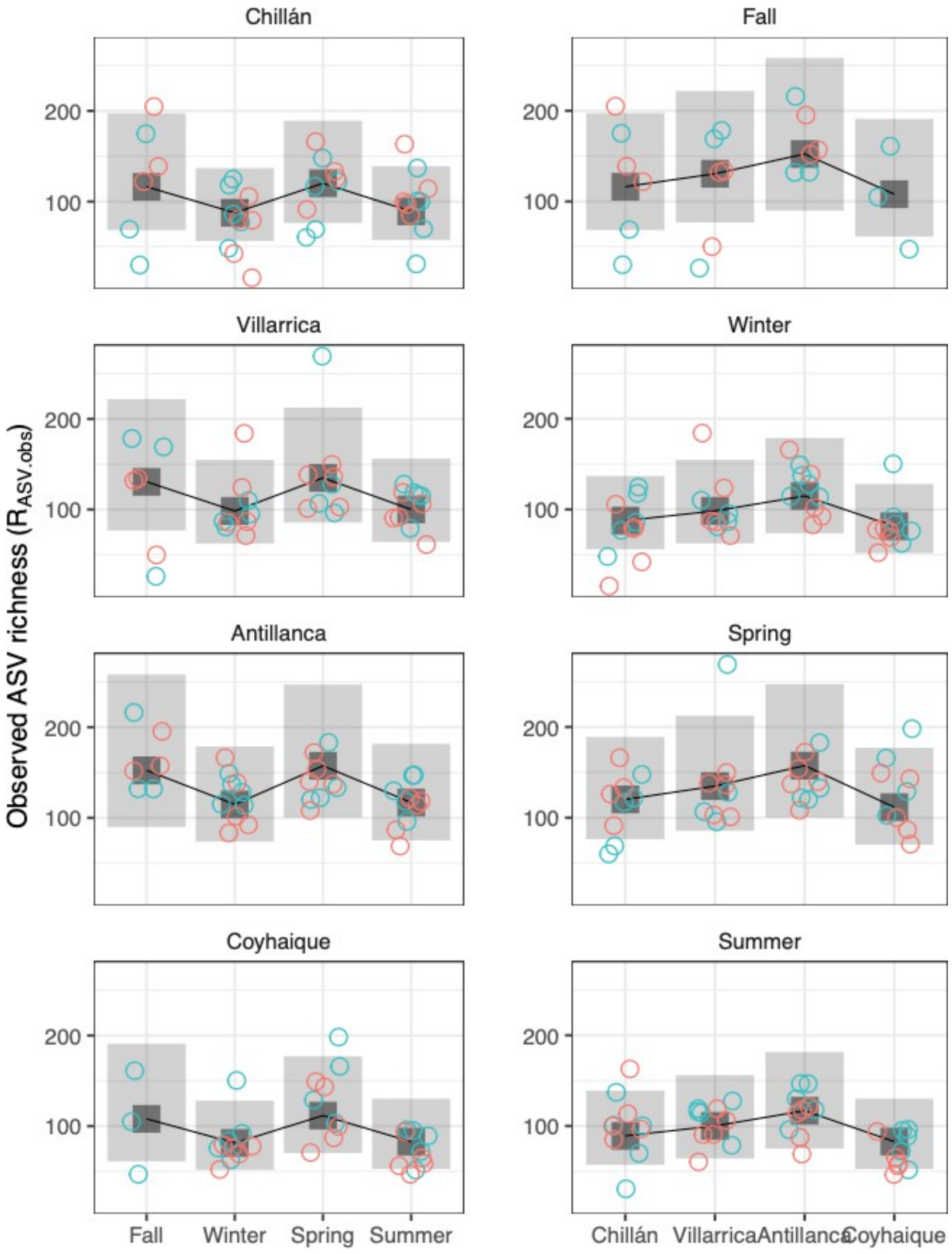

Figure 16: Results of negative binomial GLM on observed ASV richness. Model mean fit (dark squares) and predictive 95% confidence intervals (boxes) alongh with observed ASV richness (red and blue circles, for treeline and below treeline, respectively). Though somehow redundant, each column emphasizes either temporal or spatial patterns. This final model did not include the effect of altitude.

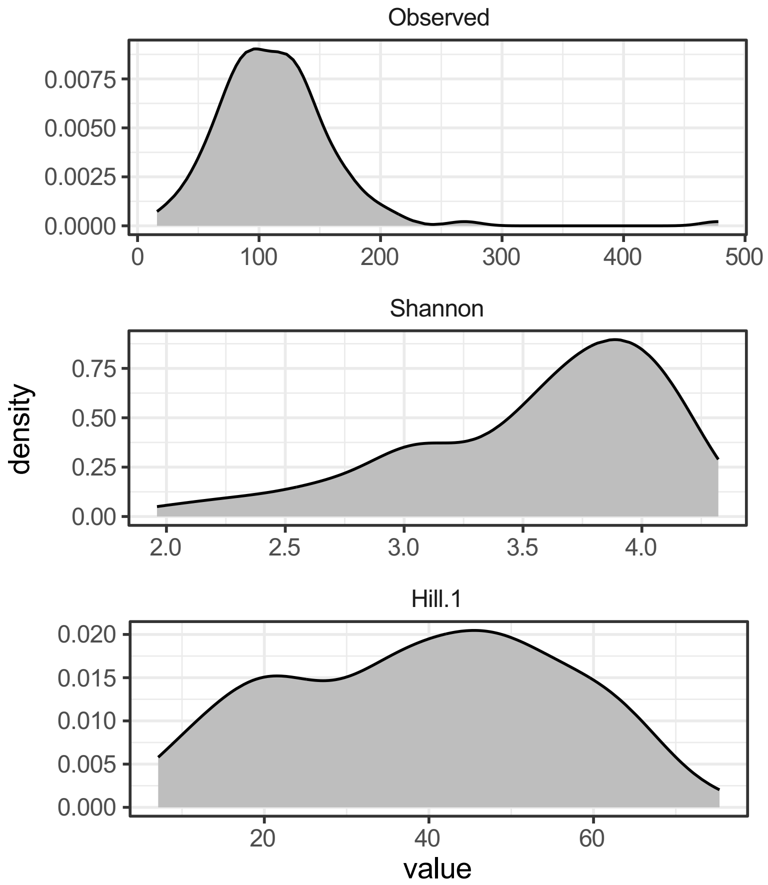

Figure 17: Density distibutions of three different diversity indices: observed richness (studied in depth in the previos section), Shannon entropy in its original form, and Hill numbers (*q* = 1) of the latter, expressing effective number of ASVs.

```
# Incorporate to sample_data
tmp <- full_join(H.obs, Hill.1, by = "Sample_ID")

H.obs <- data.frame(sample_data(pseq2_filtered)) %>%
    rownames_to_column(var = "Sample_ID") %>% full_join(.,
    tmp, by = "Sample_ID") %>% column_to_rownames(var =
    "Sample_ID")

sample_data(pseq2_filtered) <- H.obs
```

```
diversity %>%
    ggplot(aes(x = value, group = "Div.index")) + facet_wrap(~Div.index, scales = "free",
    ncol = 1) + geom_density(fill = "gray") + theme_bw() + theme(strip.background = element_blank())
```

7.3.1. Linear model on Hill numbers q = 1

The following linear model was fitted to Hill numbers, *q* = 1 (i.e., exp(Shannon index)).

$$\text{Hill1}_i \sim N(\mu_i, \sigma)$$
$$E(Y \mid X) = \mu$$
$$\mu_i = \beta_0 + \beta_1 \text{Season}_i + \beta_2 \text{Location}_i + \beta_3 \text{Altitude}_i + \beta_4 \text{Location}_i \times \text{Altitude}_i + \beta_5 \text{Season}_i \times \text{Altitude}_i \quad (3)$$

Its distribution of residuals was not significantly different from a normal distribution (see Shapiro-Wilk normality test; Figure **??**), and diagnostic plots of residuals were adequate (Figure 18).

```
div.data <- data.frame(unclass(sample_data(pseq2_filtered))) %>% mutate(Sample_ID =
        paste0(Name, Replicate)) %>%
        dplyr::select(Sample_ID, Location, Season, Altitude, Observed, Shannon, Hill.1)

m1 <- lm(Hill.1 ~ Location + Season + Altitude + Location:Altitude + Season:Altitude, data = div.data)

# Shapiro-Wilk normality test
shapiro.test(residuals(m1))
```

```
##
##  Shapiro-Wilk normality test ##
## data:  residuals(m1)
## W = 0.98611, p-value = 0.1818
```

```
## Warning: Using `size` aesthetic for lines was deprecated in ggplot2 3.4.0. ## i Please use
`linewidth` instead.
## This warning is displayed once every 8 hours.
## Call `lifecycle::last_lifecycle_warnings()` to see where this warning was ## generated.
```

```
## Warning: The dot-dot notation (`..density..`) was deprecated in ggplot2 3.4.0. ## i Please use
`after_stat(density)` instead.
## This warning is displayed once every 8 hours.
## Call `lifecycle::last_lifecycle_warnings()` to see where this warning was ## generated.
```

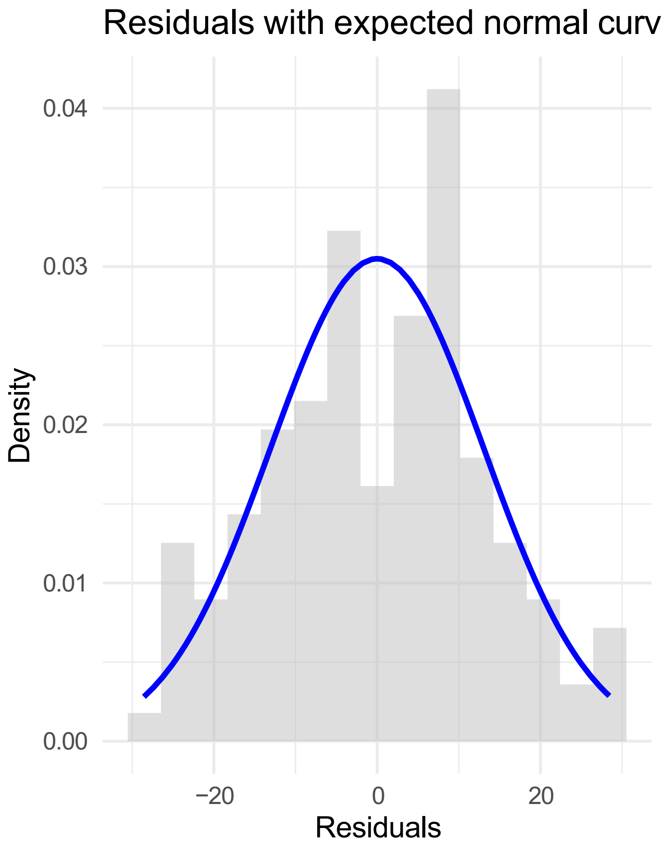

7.3.2. *Model selection*

We used likelihood ratio tests to sequentially drop non-significant terms. Each term was tested at a time by dropping the term, refitting the model, and and comparing it with the full model.

```
LRT <- drop1(m1, test = "Chisq")
```

Table 6: Likelihood ratio tests (LRT) for interaction terms of the linear model of Hill.1 [exp(Shannon entropy)]. The first row represents the full model.

	Df	Sum of Sq	RSS	AIC	Pr(>Chi)
<none>	NA	NA	23272.78	731.5031	NA
Location:Altitude	3	30.18788	23302.97	725.6807	0.9811230
Season:Altitude	3	890.78411	24163.57	730.6490	0.1614189

The LRTs for the interaction terms were not significant (Table 6). Therefore both interactions were dropped, and a new set of LRTs were conducted to evaluate the main effects.

```
m2 <- update(m1, formula. = . ~ . - Location:Altitude - Season:Altitude)

LRT2 <- drop1(m2, test = "Chisq")
```

The main effects were highly significant (Table 7). The diagnostic plots suggest the fit is adequate (Figure 19).

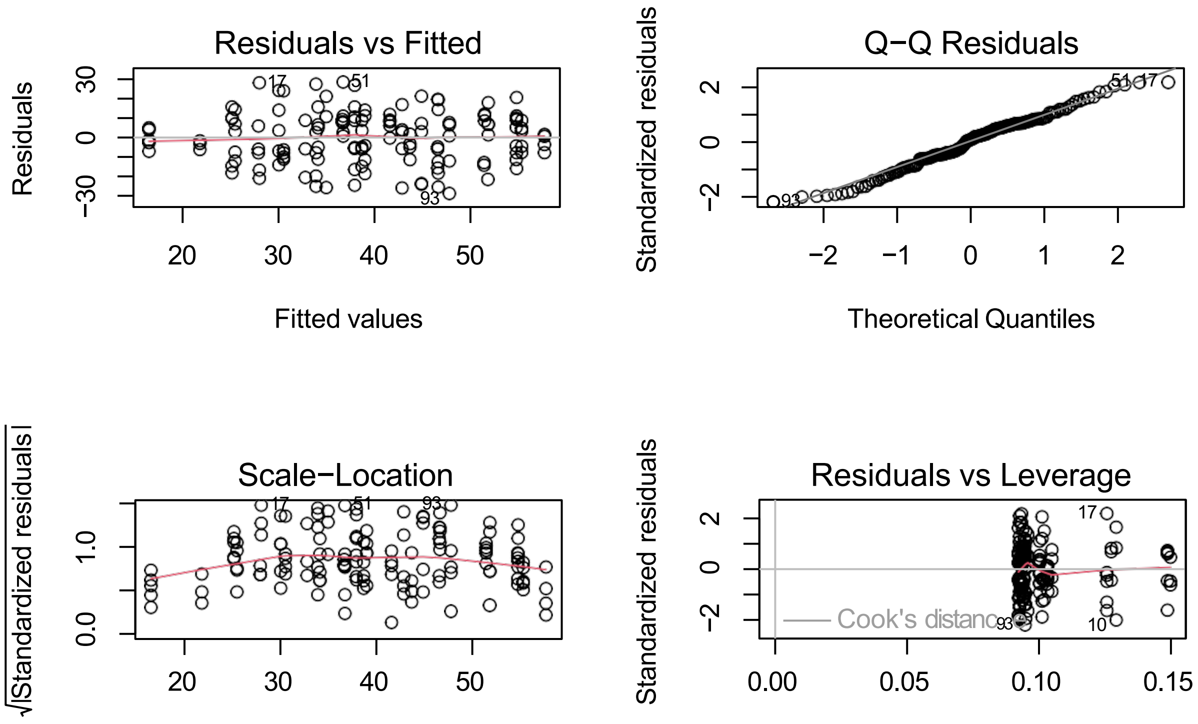

Figure 18: Standard diagnostic plots for LM.

Table 7: Likelihood ratio tests (LRT) for individual terms of the linear model of Hill.1. The first row represents the full model.

	Df	Sum of Sq	RSS	AIC	Pr(>Chi)
<none>	NA	NA	24204.96	724.8835	NA
Location	3	5644.085	29849.05	747.5980	0.0000026
Season	3	7415.478	31620.44	755.4962	0.0000001
Altitude	1	2291.289	26496.25	735.2745	0.0004314

Therefore, the chosen model was (Eqn. 4):

$$\begin{aligned} \text{Hill1}_i &\sim N(\mu_i, \sigma) \\ \text{E}(Y\,|X) &= \mu \\ \mu_i &= \beta_0 + \beta_1\text{Season}_i + \beta_2\text{Site}_i + \beta_3\text{Altitude}_i \end{aligned} \tag{4}$$

The overall model was highly significant, with an adjusted R^2 = 33.4%.

```
summary(m2)
```

```
##
## Call:
## lm(formula = Hill.1 ~ Location + Season + Altitude, data = div.data) ##
## Residuals:
##         Min          1Q  Median          3Q         Max
```

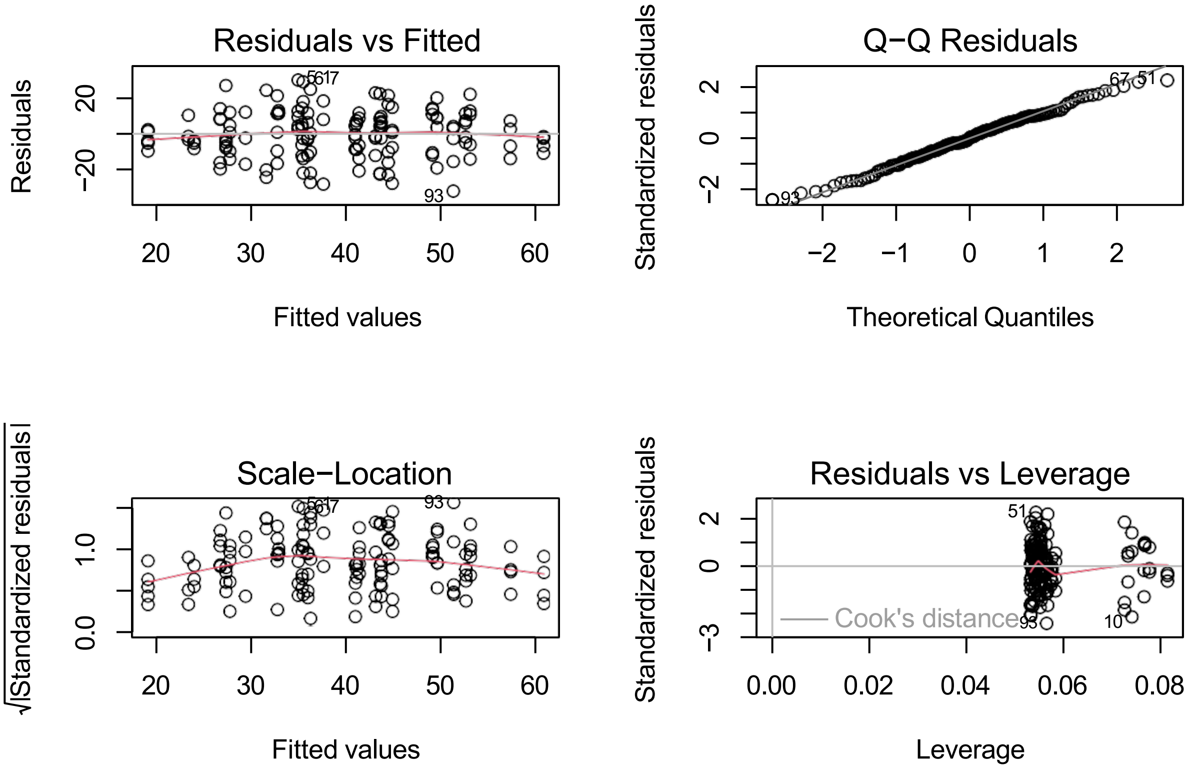

Figure 19: Diagnostic plots of final LM for effective number of ASVs.

```
## -32.108  -9.556        0.326     9.478  30.220 ##
## Coefficients:
##                          Estimate Std. Error t value Pr(>|t|)
## (Intercept)                23.401      3.818   6.129 9.97e-09 ***
## LocationVillarrica          6.008      3.301   1.820 0.071031 .
## LocationAntillanca          9.502      3.275   2.901 0.004371 **
## LocationCoyhaique          -7.611      3.336  -2.282 0.024140 *
## SeasonWinter                3.350      3.727   0.899 0.370437
## SeasonSpring               19.703      3.765   5.233 6.57e-07 ***
## SeasonSummer               11.971      3.708   3.229 0.001577 **
## AltitudeBelow treeline      8.209      2.349   3.494 0.000652 *** ##
---
## Signif. codes:  0 '***' 0.001 '**' 0.01 '*' 0.05 '.' 0.1 ' ' 1 ##
## Residual standard error: 13.7 on 129 degrees of freedom ## Multiple R-
squared:  0.3684, Adjusted R-squared:  0.3341 ## F-statistic: 10.75 on 7 and
129 DF,  p-value: 1.274e-10
```

A coefficient plot can be seen in Figure 20.

Warning: Length of `group.terms` does not equal number of model coefficients. ## Ignoring this argument.

The model fit and 95% predictive confidence intervals for the full experimental design was also plotted, along with the original data (Figure 21).

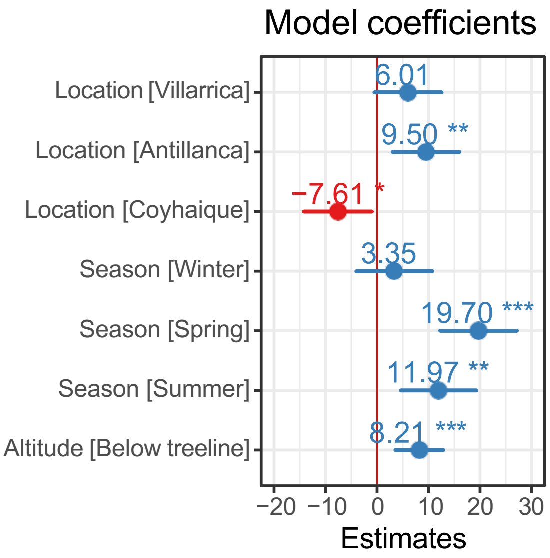

Figure 20: Model coefficients of reduced negative binomial GLM. Note these are in the link scale.

```
newdata <- expand.grid(Location = unique(div.data$Location), Season = unique(div.data$Season), Altitude =
        unique(div.data$Altitude))

preds <- predict(m2, newdata, se.fit = TRUE, interval = "prediction") preds <-
as.data.frame(preds$fit)
newdata$fit <- preds$fit
newdata$upper <- preds$upr  #$fit + 1.96 * preds$se.fit
newdata$lower <- preds$lwr  #$fit - 1.96 * preds$se.fit
```

To illustrate the effect size of the marginal contribution of *Altitude* to the effective number of ASV (Hill.1) after adjusting for *Location* and *Season*, we plotted the residuals of a reduced model lacking *Altitude* against *Altitude*. This provided a good appreciation of the marginal contribution of *Altitude* to the *Hill.1* in light of additional unmodeled variation (residual error; Figure 22).

```
# Fit reduced model
m3.alt <- update(m2, . ~ . - Altitude) div.data$residuals.Altitude <-
residuals(m3.alt)

# Create a data frame
means.sd <- div.data %>%
        group_by(Altitude) %>%
        summarise(mu = mean(residuals.Altitude, na.rm = T), sigma = sd(residuals.Altitude, na.rm = T))
# Fill in with means and (single) sigma value from lm()
means.sd$mu <- coef(lm(residuals.Altitude ~ 0 + Altitude, data = div.data)) means.sd$sigma <-
sigma(lm(residuals.Altitude ~ 0 + Altitude, data = div.data))
```

```
# devtools::install_github('wjschne/ggnormalviolin')
library("ggnormalviolin")

div.data %>%
```

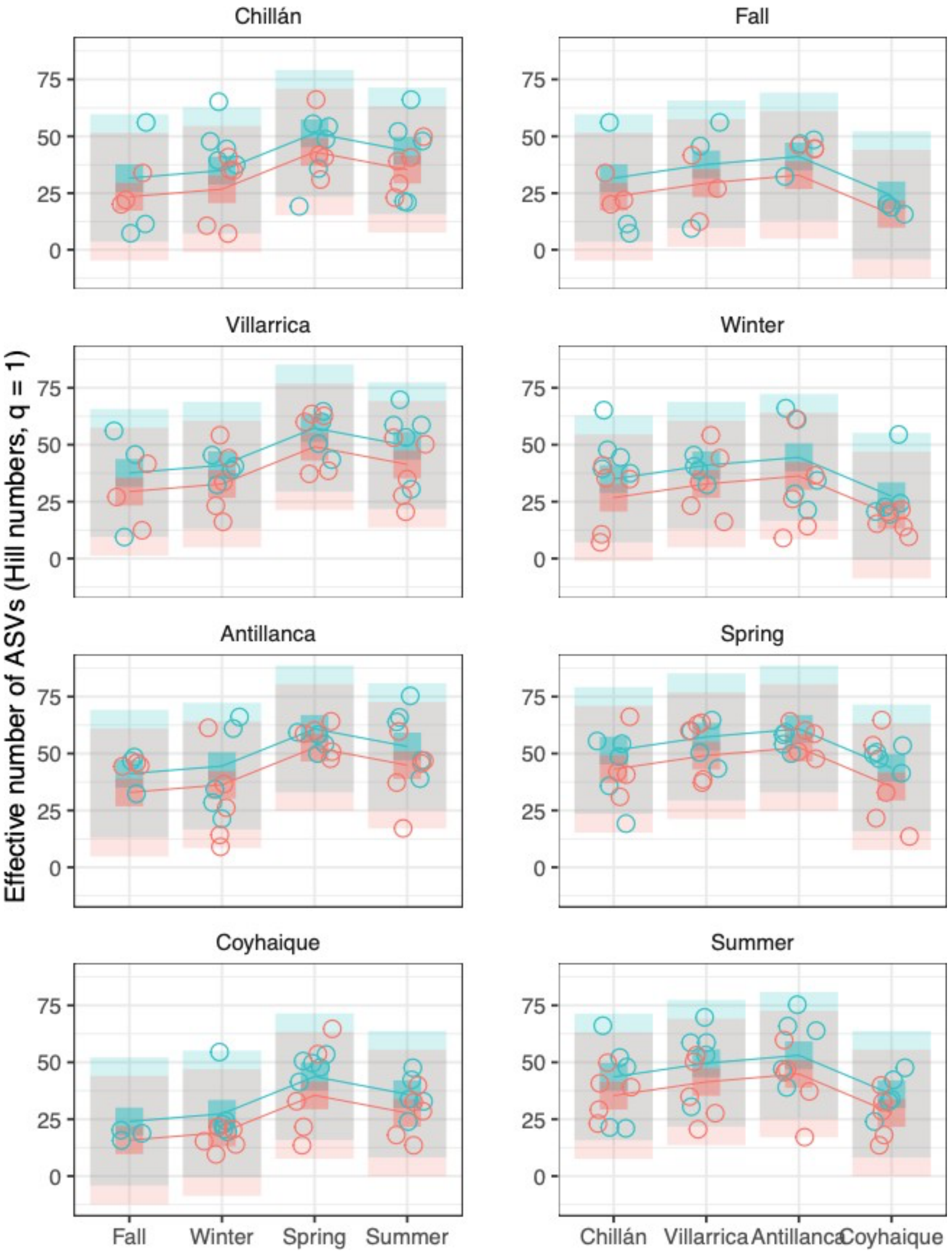

Figure 21: Results of linear model on effective number of ASVs at q = 1 [= exp(Shannon index)]. Model mean fit (squares) and predictive 95% confidence intervals (boxes) alongh with observed ASV richness (red and blue circles, for treeline and below treeline, respectively). Though somehow redundant, each column emphasizes either temporal or spatial patterns.

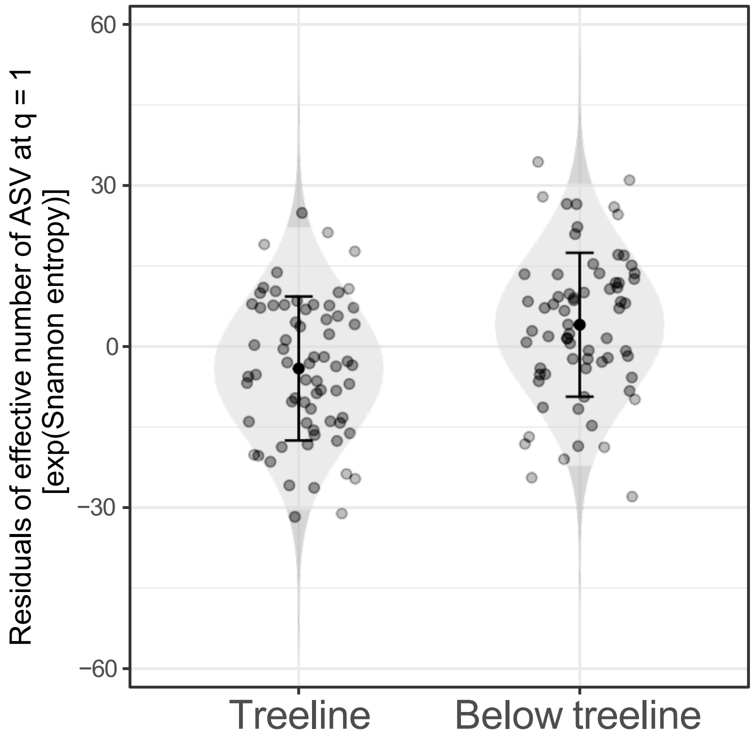

Figure 22: Plot of residuals illustrating the effect size of *Altitude* after accounting for *Location* and *Season* (see text). In the background, normal homoscedastic violin plots represent the density function of model predictions. Black dots and errorbars show the means +- SDs.

```
    ggplot() + # geom_violin()+ ggplot() + # geom_violin()+
geom_normalviolin(data = means.sd, aes(x = Altitude, mu = mu, sigma = sigma), alpha = 0.5, tail_alpha = 0.15,
    show.legend = T, p_tail = 0.05) + geom_point(data = means.sd,
    aes(x = Altitude, y = mu)) + geom_errorbar(data = means.sd, aes(x = Altitude,
    ymin = mu - sigma, ymax = mu + sigma), width = 0.1) + geom_jitter(data = div.data,
    aes(Altitude, residuals.Altitude), width = 0.2, alpha = 0.5) + labs(y = "Residuals of effective n
    theme_bw() + theme(axis.title.x = element_text(size = 0), axis.text.x = element_text(size = 15))
```

The results showed a statistically significant (see above) yet modest effect size of *Altitude*, though in the expected direction. After adjusting for the effects of *Location* and *Season*, fungi in the rhizosphere of soil 200 m below the treeline had on average only 8.15 ASVs more than that at the treeline (Figure 22).

Similarly, we studied the marginal contribution of *Location* and *Season* (Figures 23 and 24).

```
m3.loc <- update(m2, . ~ . - Location)
div.data$residuals.Location <- residuals(m3.loc) # Create a
data frame
means.sd <- div.data %>%
    group_by(Location) %>%
    summarise(mu = mean(residuals.Location, na.rm = T), sigma = sd(residuals.Location, na.rm = T))
# Fill in with means and (single) sigma value from lm()
means.sd$mu <- coef(lm(residuals.Location ~ 0 + Location, data = div.data)) means.sd$sigma <-
sigma(lm(residuals.Location ~ 0 + Location, data = div.data))
```

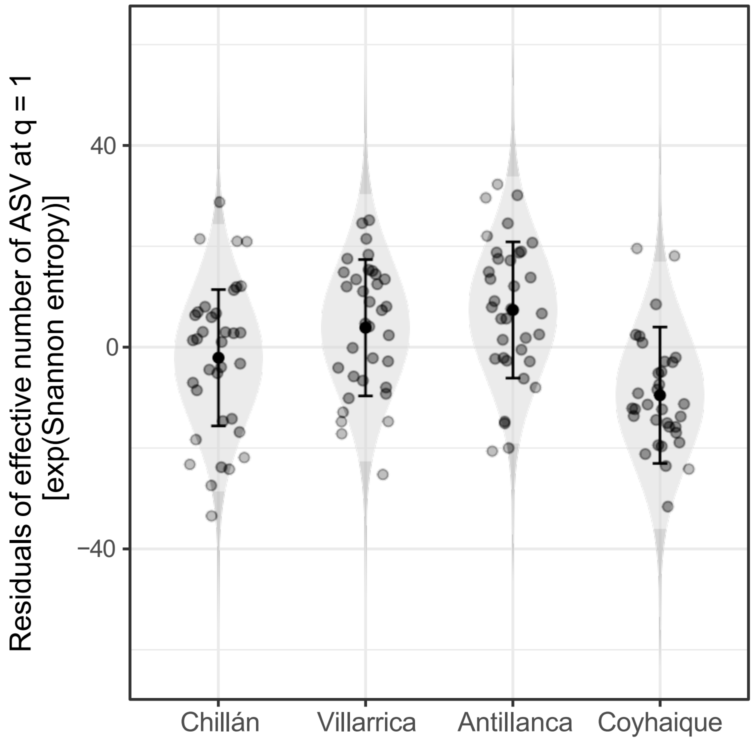

Figure 23: Plot of residuals illustrating the effect size of *Location* after accounting for *Altitude* and *Season*. Other conventions as in Figure 22

```
# devtools::install_github('wjschne/ggnormalviolin')
library("ggnormalviolin")

div.data %>%
    ggplot() + # geom_violin()+ ggplot() + # geom_violin()+
geom_normalviolin(data = means.sd, aes(x = Location, mu = mu, sigma = sigma), alpha = 0.5, tail_alpha = 0.15,
    show.legend = T, p_tail = 0.05) + geom_point(data = means.sd,  aes(x = Location, y = mu)) +
    geom_errorbar(data = means.sd, aes(x = Location,
    ymin = mu - sigma, ymax = mu + sigma), width = 0.1) + geom_jitter(data = div.data,
    aes(Location, residuals.Location), width = 0.2, alpha = 0.5) + labs(y = "Residuals of effective n
    theme_bw() + theme(axis.title.x = element_text(size = 0), axis.text.x = element_text(size = 10))
```

```
# Fit reduced model
m3.sea <- update(m2, . ~ . - Season) div.data$residuals.Season <-
residuals(m3.sea)

# Create a data frame
means.sd <- div.data %>%
    group_by(Season) %>%
    summarise(mu = mean(residuals.Season, na.rm = T), sigma = sd(residuals.Season, na.rm = T))
# Fill in with means and (single) sigma value from lm()
means.sd$mu <- coef(lm(residuals.Season ~ 0 + Season, data = div.data))
```

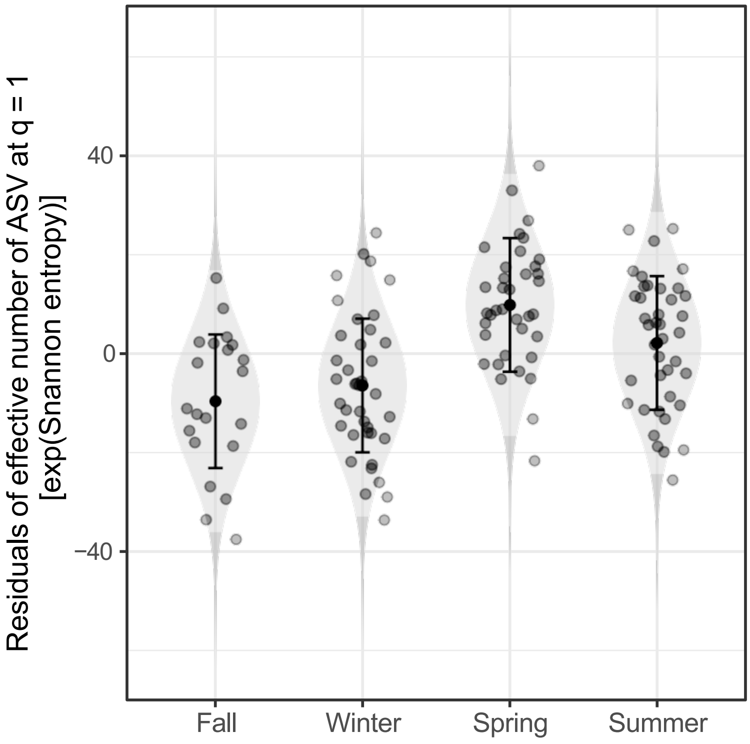

Figure 24: Plot of residuals illustrating the effect size of *Season* after accounting for Altitude and Location. Other conventions as in Figure ef{fig:resalt}

```
means.sd$sigma <- sigma(lm(residuals.Season ~ 0 + Season, data = div.data))
```

```
# devtools::install_github('wjschne/ggnormalviolin')
library("ggnormalviolin")

div.data %>%
    ggplot() + # geom_violin()+ ggplot() + # geom_violin()+
geom_normalviolin(data = means.sd, aes(x = Season, mu = mu, sigma = sigma), alpha = 0.5, tail_alpha = 0.15,
    show.legend = T, p_tail = 0.05) + geom_point(data = means.sd, aes(x = Season, y = mu)) +
    geom_errorbar(data = means.sd, aes(x = Season, ymin = mu - sigma, ymax = mu + sigma), width = 0.1) +
    geom_jitter(data = div.data, aes(Season,
    residuals.Season), width = 0.2, alpha = 0.5) + labs(y = "Residuals of effective number of ASV at
    theme_bw() + theme(axis.title.x = element_text(size = 0), axis.text.x = element_text(size = 10))
```

7.3.3. *Partial coefficients of determiaation, partial-R^2*

The partial coefficient of determination (partil R^2) for each explanatory variable was 18.9, 23.5, 8.6% for Location, Season, Altitude, respectively. The overall coefficient of determination was (R^2) = 33.4% (adjusted R^2 = 33.4%).

```r
# library(rsq)

rsq::rsq.partial(objF = m2, type = "v", adj = T)
```

```
##$adjustment ##
[1] TRUE
##
## $variable
## [1] "Location" "Season"          "Altitude" ##
## $partial.rsq
## [1] 0.17022919 0.21671336 0.07939439
```

```r
# rsq::rsq.partial(objF = m1, objR = m2, type='v', adj = F)

# Manual approximation for the records
(sigma(m3.loc)^2 - sigma(m2)^2)/sigma(m3.loc)^2
```

```
## [1] 0.1702292
```

```r
(sigma(m3.sea)^2 - sigma(m2)^2)/sigma(m3.sea)^2
```

```
## [1] 0.2167134
```

```r
(sigma(m3.alt)^2 - sigma(m2)^2)/sigma(m3.alt)^2
```

```
## [1] 0.07939439
```

SUPPLEMENTARY METERIAL: **Chapter 2: Beta diversity**

1. System

This document was produced in R Markdown, including a narrative written in LATEX and data handling and analyses conducted in R (R Core Team, 2021).

1.1. System and R version

R version 4.3.2 (2023-10-31)
Platform: x86_64-pc-linux-gnu (64-bit) ## Running under: Ubuntu 20.04.6 LTS

A complete list of session information is included at the end of this document.

1.2. R Libraries

The following libraries were used.

```
rm(list = ls())
#install.packages('microviz') devtools::install_github('joey711/phyloseq') library("phyloseq") #
http://joey711.github.io/phyloseq/ library("metagMisc") #
devtools::install_github('vmikk/metagMisc')
# library('philr') # Load later to avoid annoying messages library('microViz') # # sourced later
https://david-barnett.github.io/microViz/index.html
# library('Biostrings') # sourced later library("tidyr") #
pivot_longer library("stringr")
library("ggplot2") library("tibble")
# library('recipes') library('plyr') # join functions
library("dplyr")
# # library('Cairo') install.packages('kableExtra', dependencies = T)
# remove.packages('kableExtra') devtools::install_github('kupietz/kableExtra')
library("kableExtra") # great pretty tables, pipeline flow
library("ggmap")
# library(rgdal) library(rgeos) # need to migrate to package sf!   library(sp) # library('grid') # arrange
ggplots together
library("gridExtra") # arrange ggplots together
# library(rgbif) library(lubridate) library(dbscan) # find point clusters
```

2. Phyloseq object preparation

```
# Import core phyloseq object
pseq2_filtered <- readRDS(file = "../Received files_2024_01/pseq2_2024_02_01.rds") pseq2_filtered
```

```
## phyloseq-class experiment-level object
## otu_table()          OTU Table:          [ 5739 taxa and 137 samples ]
## sample_data() Sample Data:               [ 137 samples by 11 sample variables ] ##
tax_table()             Taxonomy Table:     [ 5739 taxa by 8 taxonomic ranks ]
## phy_tree()           Phylogenetic Tree: [ 5739 tips and 5737 internal nodes ] ## refseq()
                        DNAStringSet:       [ 5739 reference sequences ]
```

```
# tmp <- get_variable(pseq2_filtered) dput(colnames(tmp)) # Import

additional phyloseq with soil data (only summer)
```

```
soil_vars <- readRDS(file = "../Received files_2024_01/phy_summer_soil_variables_2024_01_23_LS.rds")

# Merge the sample data of both
tmp1 <- get_variable(pseq2_filtered) tmp2 <-
get_variable(soil_vars)
sample_data(pseq2_filtered) <- left_join(tmp1, tmp2, by = join_by(Sample_ID, Name, Location, Season, Altitude,
        Replicate, Latitude, Longitude)) %>% column_to_rownames("Sample_ID")

# get_variable(pseq2_filtered) %>% filter(!is.na(Organic_matter)) %>%
# ggplot(aes(Location, Organic_matter, col=Altitude))+ geom_jitter(width =
# 0.3)+ theme_bw()

rm(tmp1, tmp2, soil_vars)
```

2.0.1. ASV labelling and sequence storage

For easy display purposes, shorten the column names (ASV) by using nicknames instead, and keeping the original sequences in the *refseq* slot of the phyloseq object. Later, the original names can be accessed via refseq(my_pyloseq_object).

(ignore this step if it was already done)

```
dna <- Biostrings::DNAStringSet(taxa_names(pseq2_filtered))

names(dna) <- taxa_names(pseq2_filtered) pseq2_filtered <-
merge_phyloseq(pseq2_filtered, dna)
taxa_names(pseq2_filtered) <- paste0("ASV", seq(ntaxa(pseq2_filtered)))

# head(taxa_names(pseq2_filtered)) head(refseq(pseq2_filtered))
```

2.0.2. Fix and ehance sample data

```
# Reorder some factors
sample_data(pseq2_filtered)$Name <- factor(sample_data(pseq2_filtered)$Name, levels = c("CT", "CBT", "AT", "ABT",
        "VT", "VBT", "CHT", "CHBT"))

sample_data(pseq2_filtered)$Season <- factor(sample_data(pseq2_filtered)$Season, levels = c("Fall",
        "Winter", "Spring", "Summer"))

sample_data(pseq2_filtered)$Location <- factor(sample_data(pseq2_filtered)$Location, levels = c("Chillán",
        "Villarrica", "Antillanca", "Coyhaique"))

sample_data(pseq2_filtered)$Altitude <- factor(sample_data(pseq2_filtered)$Altitude, levels = c("Treeline",
        "Below treeline"))

# Create a new factor within which all samples are just replicas/pseudoreplicas
tmp <- data.frame(sample_data(pseq2_filtered))
tmp$spsample <- interaction(tmp$Name, tmp$Season)  # split-plot sample
sample_data(pseq2_filtered) <- sample_data(tmp)

# For the sake of tidiness, reorder samples
pseq2_filtered <- pseq2_filtered %>%
        microViz::ps_arrange(Location, Altitude, Season, Replicate)
```

2.0.3. Geographic coordinates

Geographic coordinates were assigned to each site and altitude.

3. Additional ancillary data

3.1. Soil samples

Soil samples were collected during the summer sampling campaign. These were analyzed at *Agroanalysis services* from the *Facultad de Agronomía e Ingeniería Forestal* of *Pontificia Universidad Catótilica de Chile*.

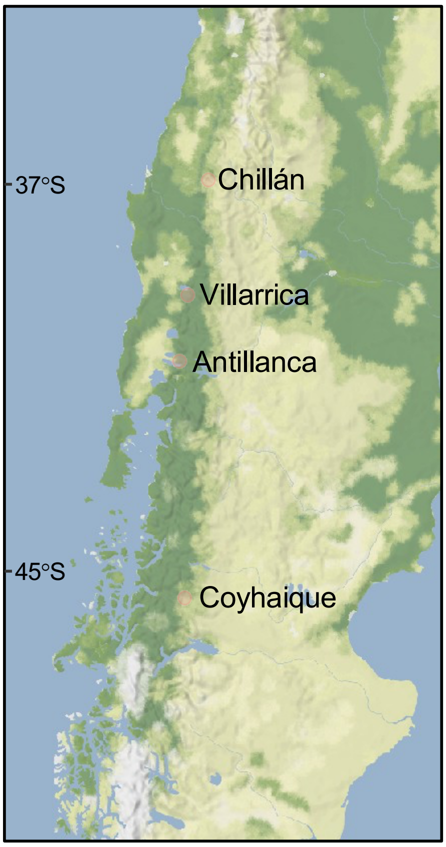

Figure 1: Distribution of sampling zones along the Andes mountain range. In each of these zones, replicated sampling was conducted at the treeline and 200 m bolow the treeline.

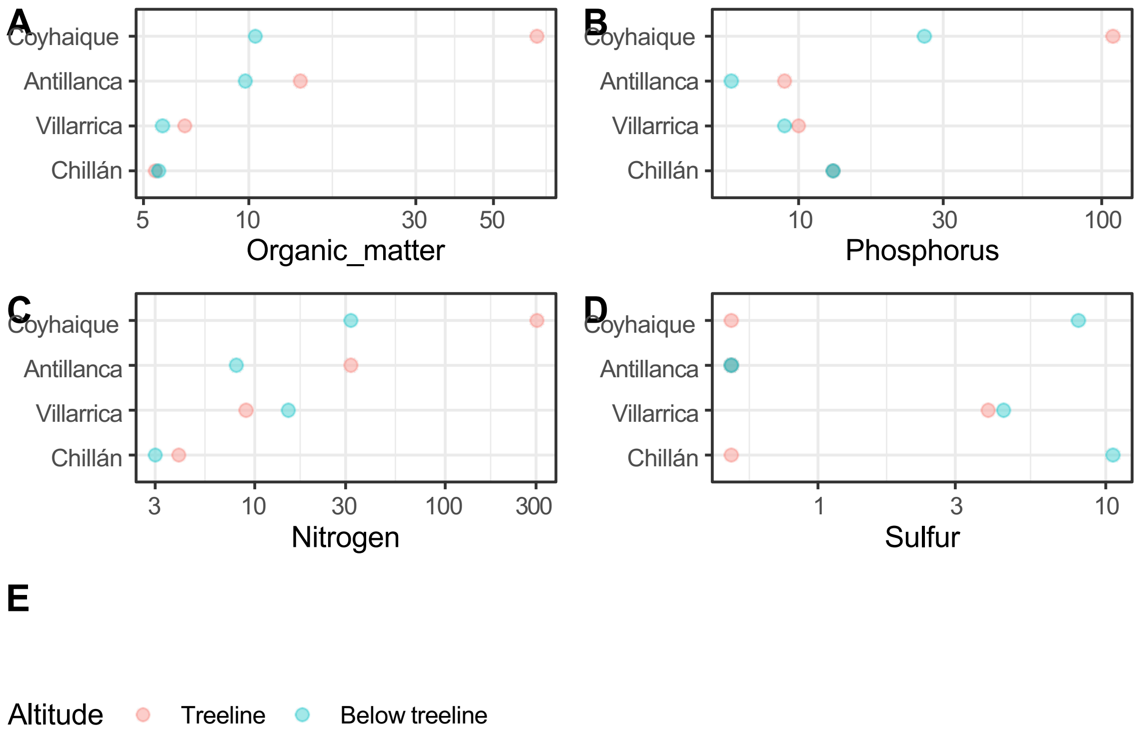

Figure 2: Soil variables (log10-transformed).

We incorporated the concentrations (mg/kg) of Phosphorus, Nitrogen, Sulfur, and the relative concentration of Organic matter (%) as environmental covariates of the fungi community data, but first, univariate and multivariate distributions of these variables were transformed and visualized. Transformation was necessary to stabilize the variances and mitigate the effect of a sample (Coyhaique, treeline) which produced very high values in several variables.

See univariate values per sample in Figures 2 and 4; pairwise bivariate plots and correlations in Figures 3 and 5), and PCAs in Figure 6.

Box-cox transformation

The Box-Cox transformation is a family of power transformations designed to stabilize variance and make data (particularly residuals) more normally distributed. It also mitigates the effect of outliers by compressing the scale. Below the code to calculate λ (the key transformation parameter) and apply the transformation to each soil variable, though the outputs were suppressed for the sake of saving space.

```
# Organic matter
tmp <- MASS::boxcox(lm(soil_data$Organic_matter ~ 1), seq(-5, 2, 1/10))
```

```
lambda <- tmp$x[which.max(tmp$y)]  # However, taken to be -1.
lambda <- -1

soil_data$OM_boxcox <- (soil_data$Organic_matter^lambda - 1)/lambda

par(mfrow = c(1, 2)) hist(soil_data$Organic_matter, breaks =
5) hist(soil_data$OM_boxcox, breaks = 5)
```

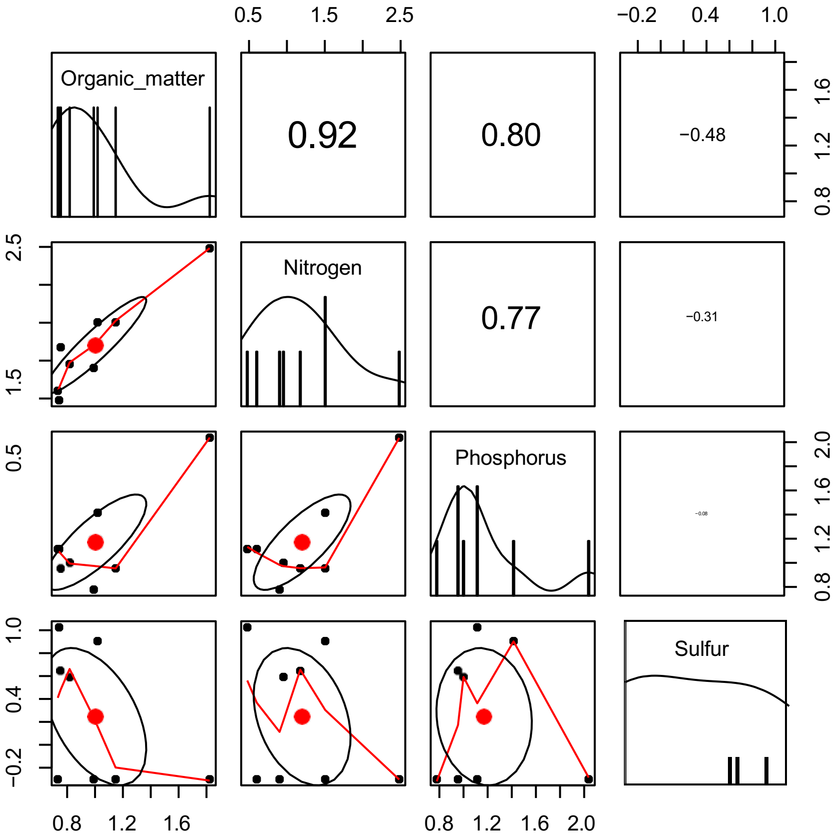

Figure 3: Pairwise soil variables correlations plot (log10-transformed).

```
# Phosphorus
tmp <- MASS::boxcox(lm(soil_data$Phosphorus ~ 1), seq(-5, 2, 1/10)) lambda <-
tmp$x[which.max(tmp$y)]   # However, taken to be -1. lambda <- -1

soil_data$Phosphorus_boxcox <- (soil_data$Phosphorus^lambda - 1)/lambda

par(mfrow = c(1, 2))
```

```
hist(soil_data$Phosphorus, breaks = 5)
hist(soil_data$Phosphorus_boxcox, breaks = 5)
```

```
# Nitrogen
tmp <- MASS::boxcox(lm(soil_data$Nitrogen ~ 1), seq(-5, 2, 1/10)) lambda <-
tmp$x[which.max(tmp$y)]   # However, taken to be -0.5. lambda <- -0.5

soil_data$Nitrogen_boxcox <- (soil_data$Nitrogen^lambda - 1)/lambda

par(mfrow = c(1, 2))
```

```
hist(soil_data$Nitrogen, breaks = 5)
hist(soil_data$Nitrogen_boxcox, breaks = 5)
```

```
# Sulfur
tmp <- MASS::boxcox(lm(soil_data$Sulfur ~ 1), seq(-5, 2, 1/10)) lambda <-
tmp$x[which.max(tmp$y)]   # However, taken to be 0. lambda <- 0

soil_data$Sulfur_boxcox <- log(soil_data$Sulfur)  par(mfrow =

c(1, 2))
```

```
hist(soil_data$Sulfur, breaks = 5)
hist(soil_data$Sulfur_boxcox, breaks = 5)
```

```
library("ggfortify")

pca1 <- princomp(~log10(Organic_matter) + log10(Nitrogen) + log10(Phosphorus) + log10(Sulfur), cor = T, data = soil_data)

pca1plot <- autoplot(pca1, data = soil_data, colour = "Location", shape = "Altitude", size = 3, loadings = TRUE,
        loadings.label = TRUE, loadings.colour = "black",
        loadings.label.colour = "black", loadings.label.repel = T) + labs(title = "PCA on log10-transform
        theme_bw() + theme(legend.position = "none")


pca2 <- princomp(~OM_boxcox + Nitrogen_boxcox + Phosphorus_boxcox + Sulfur_boxcox, cor = T, data =
        soil_data)
```

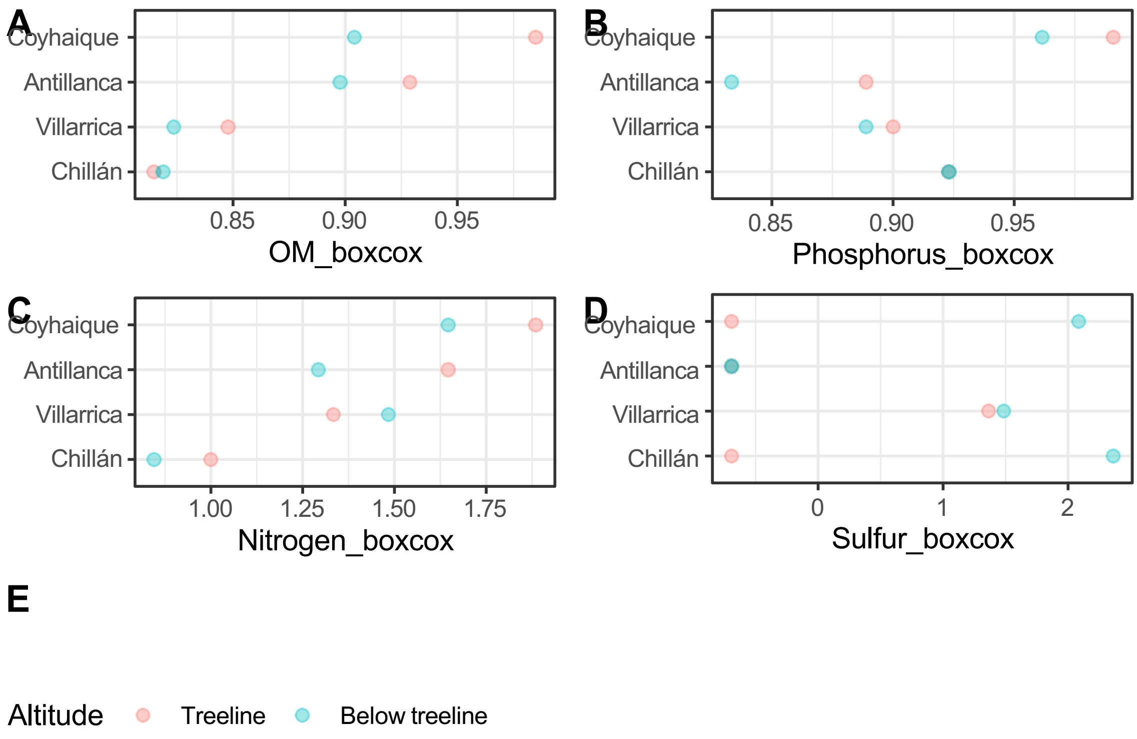

Figure 4: Soil variables (Box-Cox-transformed). Lambda was set to -1, -1, -0.5, and 0 (i.e., log(y)), for panels A to D, respectively.

```
pca2plot <- autoplot(pca2, data = soil_data, colour = "Location", shape = "Altitude", size = 3, loadings = TRUE,
        loadings.label = TRUE, loadings.colour = "black",
        loadings.label.colour = "black", loadings.label.repel = T) + labs(title = "PCA on Box-Cox transfo
        theme_bw() + theme(legend.position = "bottom", legend.justification = c(0, 0.5), legend.spacing.x = unit(-
        0.15, "cm"))

legend <- cowplot::get_legend(pca2plot)

pca2plot <- pca2plot + theme(legend.position = "none")  #labs(x=NULL)

cowplot::plot_grid(pca1plot, pca2plot, legend, nrow = 3, rel_heights = c(4, 4, 1))
```

3.2. Temperature data

A 30-year average (1970-2000) of minimum, maximum and mean temperature for each sampled month and site was downloaded from WorldClim (Figure 7).

Fick, S.E. and R.J. Hijmans, 2017. WorldClim 2: new 1km spatial resolution climate surfaces for global land areas. International Journal of Climatology 37 (12): 4302-4315.

3.3. Guilds

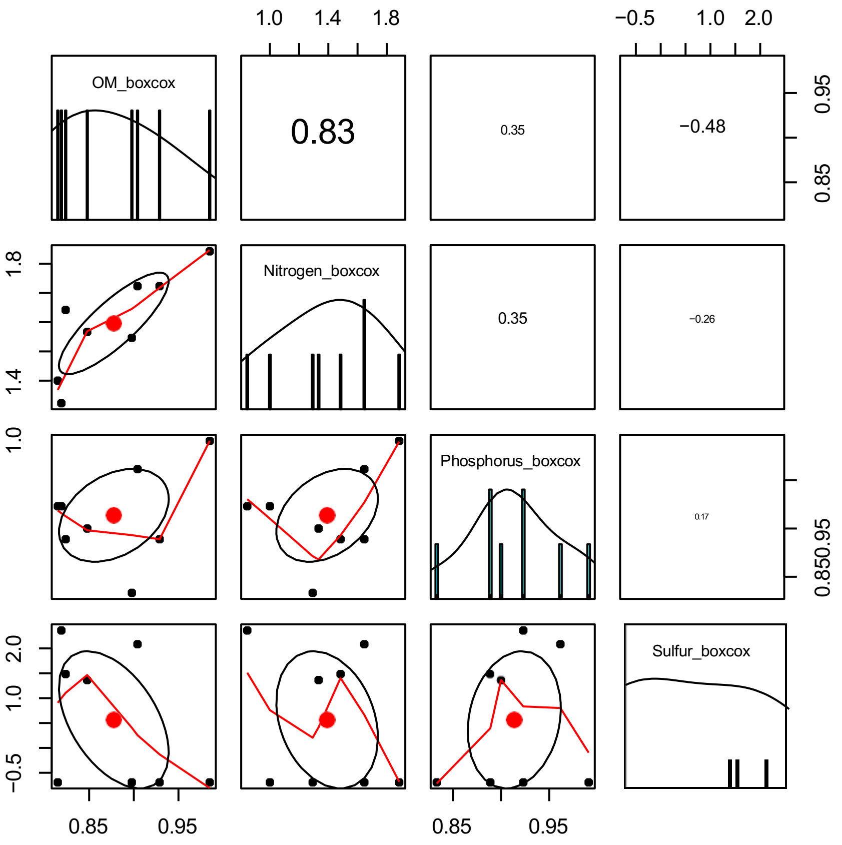

Figure 5: Pairwise soil variables correlations plot (Box-Cox-transformed). Lambda was set to -1, -1, -0.5, and 0 (i.e., log(y)), for panels A to D, respectively.

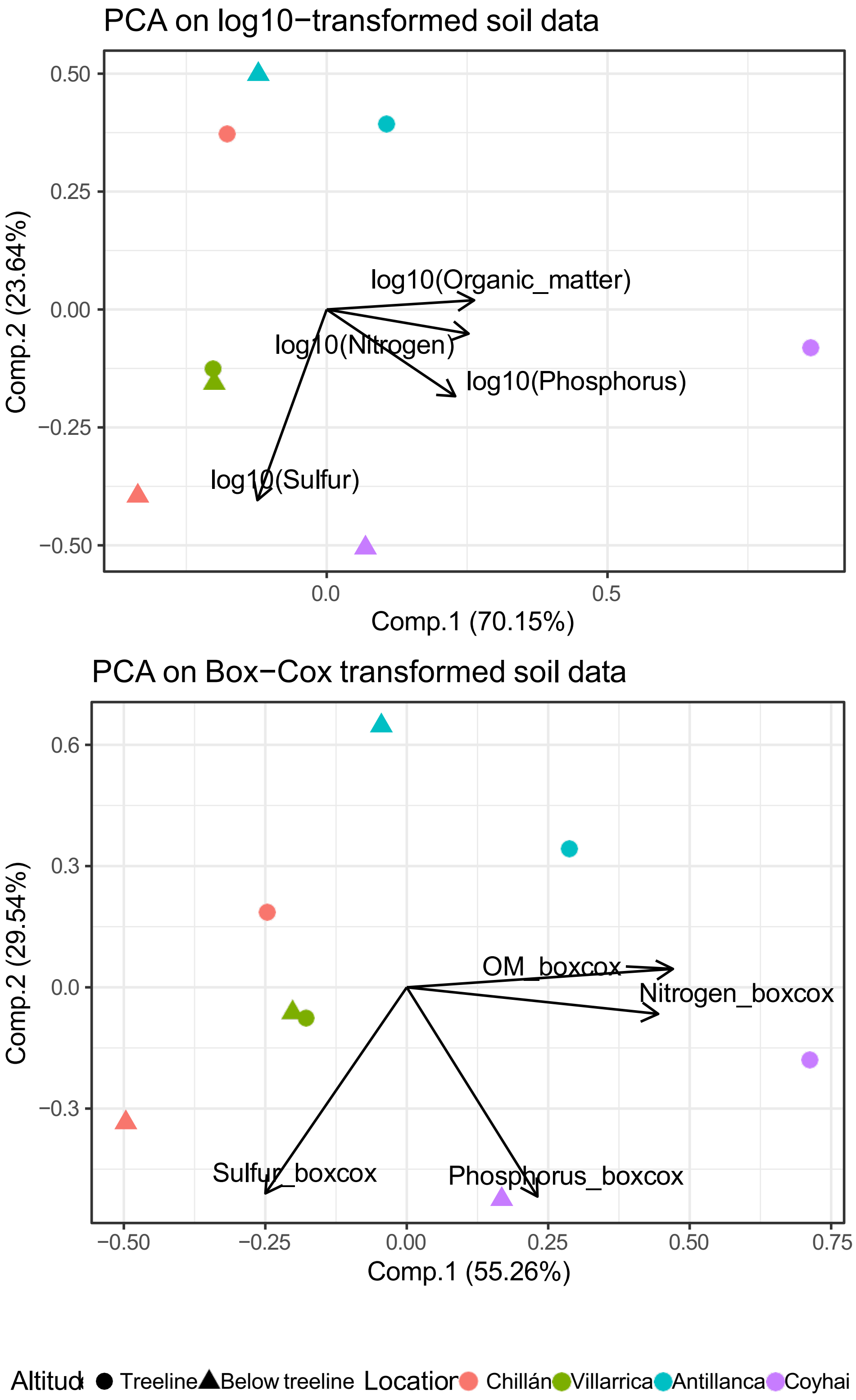

Figure 6: Principal component analysis on transformed soil variables. PCAs were carried out on correlations.

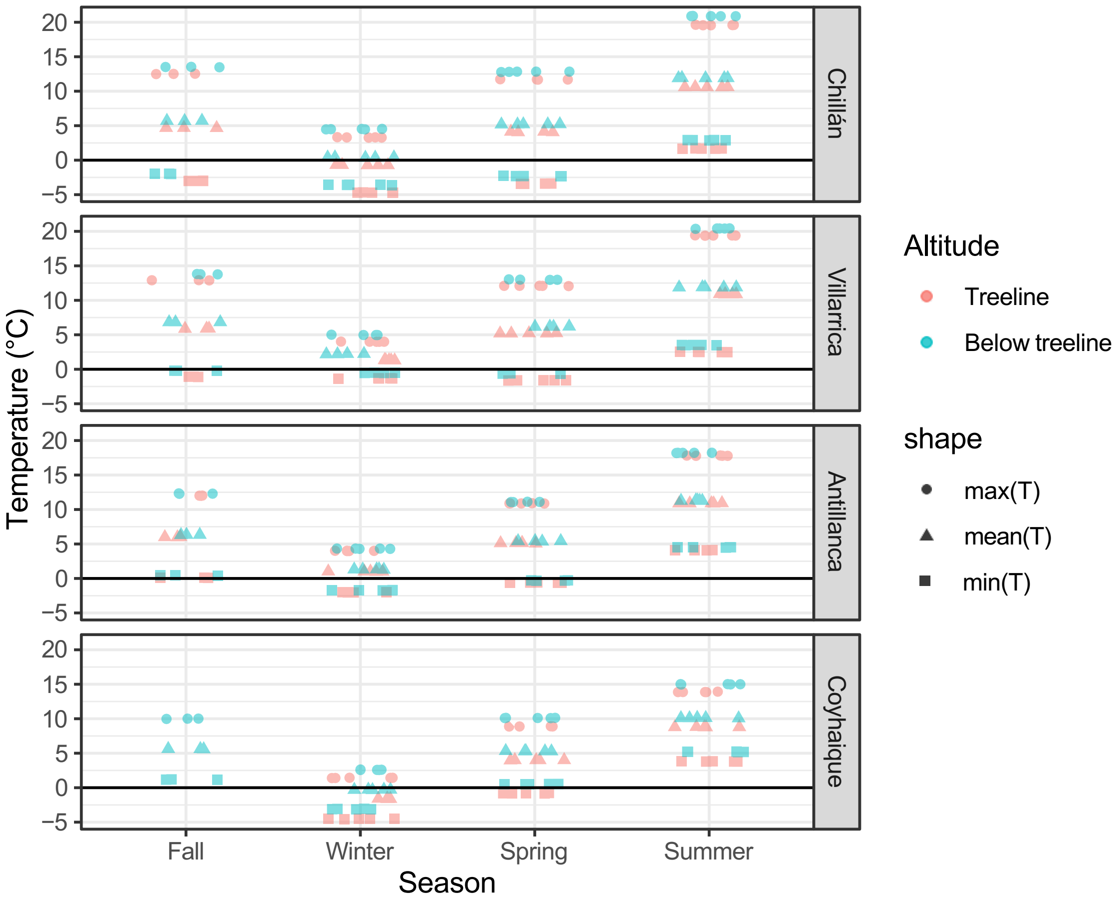

Figure 7: Thirty-year (1970-2000) average temperature ancillary data (min, mean, and max).

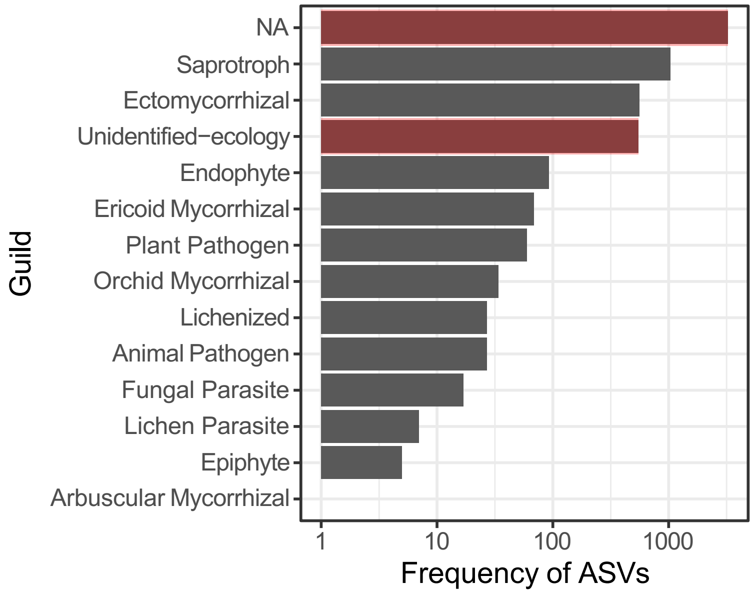

Figure 8: List of guilds and frequency with which they were attributed to ASVs. Note log10 scale.

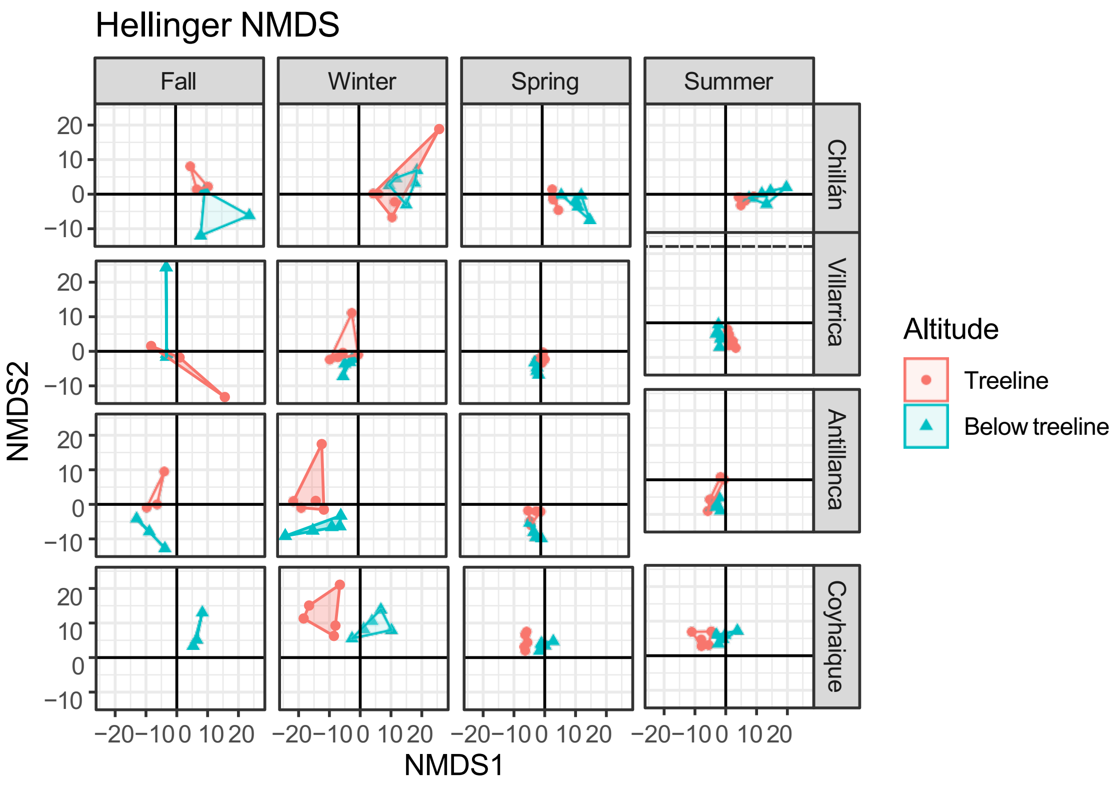

Figure 9: NMDS of phylo3 on Hellinger distances.

Thirteen guilds were attributed to 1930 ASVs; 33.7 % of ASVs were labelled with a guild. However, 3795 (66.3 %) ASVs could not be classified (sum of Undefined-ecology and NA; Figure 8).

4. Pseudoreplicates-aggregation

While some spatio-temporal patterns emerged in preliminary non-metric multidimensional scaling (NMDS) plots on Hellinger-transformed community composition data, it was striking the high variability observed between samples collected in the same site at the same time (i.e., hereafter pseudoreplicates) in comparison to the variability among seasons, localities and altitude (Figure 9; see transformations section below). This result suggests either high sampling error or high variability of the fungi community at a very fine scale. Regardless, the analyses presented below work with a data set in which speudoreplicates were combined into one composite sample.

Hence, we created a second version of the data set by combining (summing) psudoreplicated samples, that is, groups of three (fall 2022) or five (all the rest) samples collected from tree patches at the same location, altitude and season. These groups of samples were not considered true replicas because they represent a single sampling unit at the landscape scale (i.e., same forest or hill slope). This feature of the experimental design has to be considered for statistical inference, and for core analyses and visualizations the sample-aggregated data set was preferable.

```
# We begin by modifying the sample data manually because
# merge_samples{phyloseq} messes up the labels factor levels (bug).

tmp <- get_variable(pseq2_filtered) %>% group_by(Location,
       Season, Altitude, spsample) %>%
```

```
    summarise(Latitude = mean(Latitude), Longitude = mean(Longitude), T_min = mean(T_min), T_mean =
        mean(T_mean), T_max = mean(T_max), Organic_matter = mean(Organic_matter), Nitrogen =
        mean(Nitrogen), Phosphorus = mean(Phosphorus), Sulfur = mean(Sulfur),
        .groups = "keep") %>%
    column_to_rownames("spsample") %>%
    mutate(spsample = rownames(.), Altitude2 = case_match(Altitude, "Treeline" ~
        "AT", "Below treeline" ~ "BT"))

tmp$plot <- interaction(tmp$Location, tmp$Altitude) tmp$plot <-
factor(tmp$plo, levels = unique(tmp$plot))

# Group samples by factor variable using merge_samples{phyloseq}
pseq2_aggregated <- merge_samples(pseq2_filtered, group = "spsample", sum)

# Replace the sample data to recover factor labels
sample_data(pseq2_aggregated) <- sample_data(tmp)

# For the sake of tidiness, reorder samples
pseq2_aggregated <- pseq2_aggregated %>%
    microViz::ps_arrange(Location, Altitude, Season)

## Taxonomic fix Perform taxonomic fix
pseq2_aggregated <- pseq2_aggregated %>%
    microViz::tax_fix()
```

5. Preliminary filtering

5.1. Number of reads per sample

[see previous script]

5.2. Number of reads per ASV

```
otu_df <- otu_table(pseq2_aggregated)
```

The abundance of the ASVs was highly variable, in the range 0, 3.7597×10^5 (Figure 10).

5.3. Filter out rare ASVs (sample-wise)

```
ASV_abundance <- colSums(otu_df)
ASV_abundance <- sort(ASV_abundance, decreasing = TRUE)

# Set sample-wise abundance threshold, below which ASVs were filtered out.
threshold = 10
```

Warning: Transformation introduced infinite values in continuous y-axis ## Warning:

Removed 14 rows containing non-finite values (`stat_align()`).

Filtering was done using the following function:

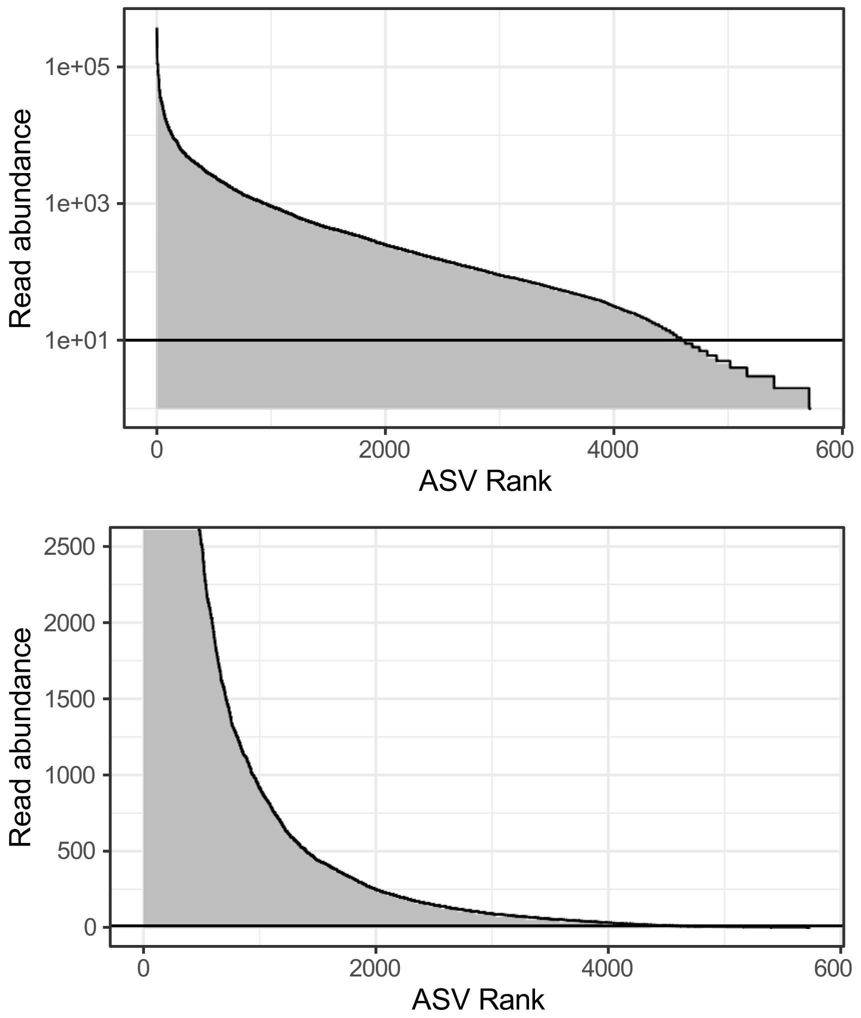

Figure 10: Total number of reads per ASV. Note the logarithmic scale in the y-axis of the top panel that shows the overall pattern, and the raw scale in the botom panel that zooms in ASVs with <2500 reads. ASVs with an absolute abundance lower than 10 were filtered out (i.e., below broken line).

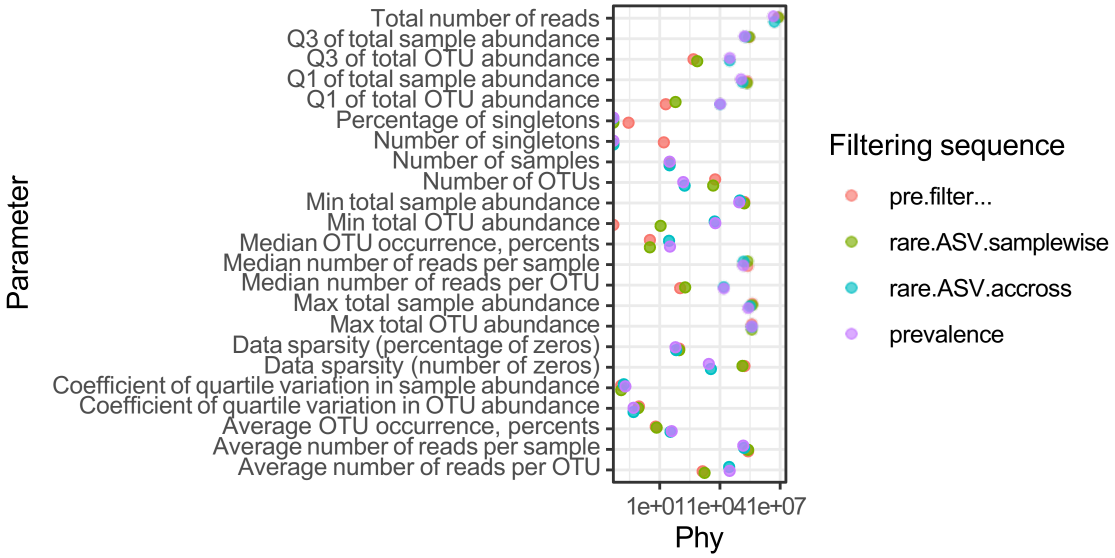

Figure 11: Comparison of phyloseq datasets after sequential filtering to remove rare ASV per sample (phylo2) and taxa with small mean relative abundance (phylo3).

```
phylo <- phyloseq_filter_sample_wise_abund_trim(pseq2_aggregated, minabund = 10, relabund = FALSE,
    rm_zero_OTUs = TRUE)
```

5.4. Filter out rare ASVs (across samples)

We further filtered out taxa with small mean relative abundance across samples (<0.1%). The frac argument of phyloseq_filter_taxa_rel_abund() has a very large impact on the number of taxa retained after filtering, because there are may rare taxa in the sample pool (note long tail in Figure 10). If frac = 0.01 (1%), then the total number of species retained was 10. If frac = 0.001 (0.1%), then the total number of species retained was 60 (Figure 13).

```
phylo2 <- phyloseq_filter_taxa_rel_abund(phylo, frac = 0.001)
```

5.5. Filter by prevalence

Because we were interested in general temporal and spacial patterns at the scale of our experimental design, we further applied a prevalence filter to exclude ASVs that only appeared one or twice among all samples.

```
# phylo3 <- phyloseq_filter_prevalence(physeq = phylo3, prev.trh = 3/137)

# function above failed, run custom filter instead:
phylo3 <- filter_taxa(phylo2, function(x) { (sum(x > 0) >= 3)
}, prune = T)
```

A complete summary comparing the different phyloseqobjects before and after filtering can be viewed in Figure 11.

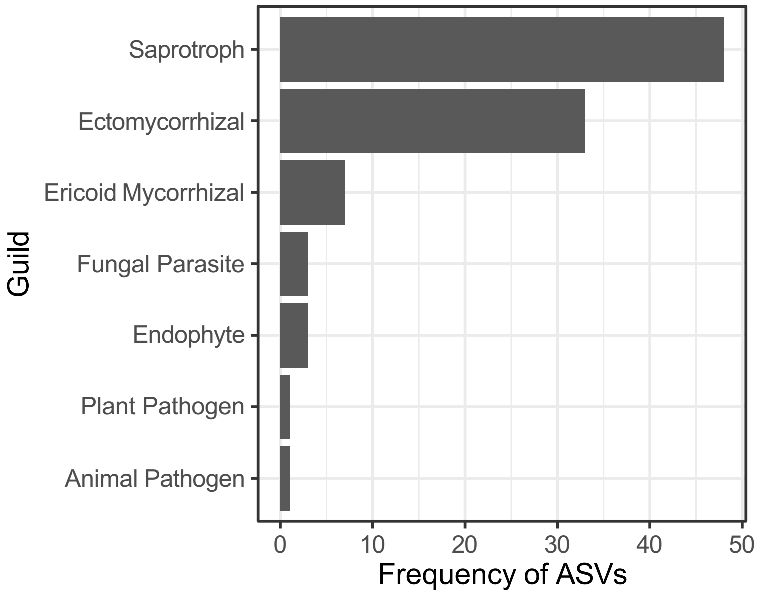

Figure 12: List of guilds included in the filtered Guilds data set, and frequency with which they were attributed to ASVs.

5.6. Filter assigned guilds into separate dataset

Because so many ASVs could not be associated to a guild, guild ordinations or direct gradient analyses were conducted using a filtered data set named *Guilds*, where ASVs with no associated guild were excluded (*cf*, Figures 8 and 12). A list of taxa that were kept in the *Guilds* data set and their associated guilds is presented in Table 1.

```
guildtable <- as.data.frame(tax_table(phylo3)) %>%
      select(Species, Guild) %>% rownames_to_column() %>%
      mutate(hasguild = !(grepl("^._", Guild) | grepl("Unidentified-ecology", Guild) | grepl("NA", Guild)))

phylo3_guilded <- prune_taxa(taxa = guildtable$hasguild, x = phylo3) phylo3_guilded
```

```
## phyloseq-class experiment-level object
## otu_table()          OTU Table:          [ 96 taxa and 31 samples ]
## sample_data() Sample Data:               [ 31 samples by 15 sample variables ] ##
tax_table()          Taxonomy Table:     [ 96 taxa by 8 taxonomic ranks ]
## phy_tree()           Phylogenetic Tree: [ 96 tips and 94 internal nodes ]
```

Table 1: List of remaining taxa in the Guilds data set, and their assigned guilds.

Family	Genus	Species	Guild
f____Trichosporonaceae	g____Cutaneotrichospor	osn___debeurmannianum	Animal Pathogen
f____Cortinariaceae	g____Cortinarius	g____Cortinarius Genus	Ectomycorrhizal
f____Cortinariaceae	g____Cortinarius	g____Cortinarius Genus	Ectomycorrhizal
f____Cortinariaceae	g____Cortinarius	g____Cortinarius Genus	Ectomycorrhizal
f____Cortinariaceae	g____Cortinarius	g____Cortinarius Genus	Ectomycorrhizal
f____Cortinariaceae	g____Cortinarius	g____Cortinarius Genus	Ectomycorrhizal
f____Cortinariaceae	g____Cortinarius	g____Cortinarius Genus	Ectomycorrhizal
f____Cortinariaceae	g____Cortinarius	g____Cortinarius Genus	Ectomycorrhizal
f____Cortinariaceae	g____Cortinarius	g____Cortinarius Genus	Ectomycorrhizal
f____Cortinariaceae	g____Cortinarius	g____Cortinarius Genus	Ectomycorrhizal
f____Cortinariaceae	g____Cortinarius	s____carneoroseus	Ectomycorrhizal
f____Cortinariaceae	g____Cortinarius	s____fuligineoviolaceus	Ectomycorrhizal
f____Cortinariaceae	g____Cortinarius	s____imbecillis	Ectomycorrhizal
f____Cortinariaceae	g____Cortinarius	s____riopancensis	Ectomycorrhizal
f____Cortinariaceae	g____Cortinarius	s____sulphureomyceliat	usEctomycorrhizal
f____Cortinariaceae	g____Phlegmacium	s____punctatisporum	Ectomycorrhizal
f____Gloniaceae	g____Cenococcum	g____Cenococcum Genus	Ectomycorrhizal
f____Gloniaceae	g____Cenococcum	g____Cenococcum Genus	Ectomycorrhizal
f____Gloniaceae	g____Cenococcum	g____Cenococcum Genus	Ectomycorrhizal
f____Hydnaceae	g____Clavulina	g____Clavulina Genus	Ectomycorrhizal
f____Hydnaceae	g____Hydnum	g____Hydnum Genus	Ectomycorrhizal
f____Inocybaceae	g____Inocybe	g____Inocybe Genus	Ectomycorrhizal
f____Inocybaceae	g____Inocybe	g____Inocybe Genus	Ectomycorrhizal
f____Inocybaceae	g____Inocybe	g____Inocybe Genus	Ectomycorrhizal
f____Inocybaceae	g____Inocybe	g____Inocybe Genus	Ectomycorrhizal
f____Melanogastraceae	g____Melanogaster	s____macrocarpus	Ectomycorrhizal
f____Pilodermataceae	g____Piloderma	g____Piloderma Genus	Ectomycorrhizal
f____Pilodermataceae	g____Piloderma	g____Piloderma Genus	Ectomycorrhizal
f____Pseudeurotiaceae	g____Pseudogymnoascu	ss____roseus	Ectomycorrhizal
f____Pyronemataceae	g____Wilcoxina	g____Wilcoxina Genus	Ectomycorrhizal
f____Sebacinaceae	g____Sebacina	g____Sebacina Genus	Ectomycorrhizal
f____Sebacinaceae	g____Sebacina	g____Sebacina Genus	Ectomycorrhizal
f____Thelephoraceae	g____Tomentella	g____Tomentella Genus	Ectomycorrhizal
o____Thelebolales Order	o____Thelebolales Order	o____Thelebolales Order	Ectomycorrhizal
f____Leptodontidiaceae	g____Leptodontidium	g____Leptodontidium Genus	Endophyte
f____Leptodontidiaceae	g____Leptodontidium	s____trabinellum	Endophyte
f____Ploettnerulaceae	g____Cadophora	g____Cadophora Genus	Endophyte
f____Myxotrichaceae	g____Oidiodendron	g____Oidiodendron Genus	Ericoid Mycorrhizal
f____Myxotrichaceae	g____Oidiodendron	g____Oidiodendron Genus	Ericoid Mycorrhizal
f____Myxotrichaceae	g____Oidiodendron	g____Oidiodendron Genus	Ericoid Mycorrhizal

Table 1: List of remaining taxa in the Guilds data set, and their assigned guilds.
(continued)

Family	Genus	Species	Guild
f____Myxotrichaceae	g____Oidiodendron	g____Oidiodendron Genus	Ericoid Mycorrhizal
f____Myxotrichaceae	g____Oidiodendron	g____Oidiodendron Genus	Ericoid Mycorrhizal
f____Myxotrichaceae	g____Oidiodendron	s____chlamydosporicum	Ericoid Mycorrhizal
f____Myxotrichaceae	g____Oidiodendron	s____pilicola	Ericoid Mycorrhizal
f____Hyaloscyphaceae	g____Hyphodiscus	g____Hyphodiscus Genus	Fungal Parasite
f____Hyaloscyphaceae	g____Hyphodiscus	g____Hyphodiscus Genus	Fungal Parasite
f____Hyaloscyphaceae	g____Hyphodiscus	g____Hyphodiscus Genus	Fungal Parasite
f____Rickenellaceae	g____Rickenella	s____minuta	Plant Pathogen
f____Aspergillaceae	g____Penicillium	g____Penicillium Genus	Saprotroph
f____Aspergillaceae	g____Penicillium	s____canescens	Saprotroph
f____Aspergillaceae	g____Penicillium	s____jamesonlandense	Saprotroph
f____Aspergillaceae	g____Penicillium	s____jamesonlandense	Saprotroph
f____Aspergillaceae	g____Penicillium	s____longicatenatum	Saprotroph
f____Aspergillaceae	g____Penicillium	s____thomii	Saprotroph
f____Chaetosphaeriacea	ef____Chaetosphaeriacea Family	ef____Chaetosphaeriacea Family	eSaprotroph
f____Geminibasidiaceae	g____Geminibasidium	g____Geminibasidium Genus	Saprotroph
f____Helotiaceae	g____Scytalidium	s____vaccinii	Saprotroph
f____Helotiales_fam_In	cgertaeL_esoehduismicola	s____minima	Saprotroph
f____Hyaloscyphaceae	f____Hyaloscyphaceae Family	f____Hyaloscyphaceae Family	Saprotroph
f____Hyaloscyphaceae	g____Hyaloscypha	g____Hyaloscypha Genus	Saprotroph
f____Hyaloscyphaceae	g____Hyaloscypha	s____finlandica	Saprotroph
f____Hyaloscyphaceae	g____Hyaloscypha	s____finlandica	Saprotroph
f____Hyaloscyphaceae	g____Hyaloscypha	s____fuckelii	Saprotroph
f____Hyaloscyphaceae	g____Lachnellula	g____Lachnellula Genus	Saprotroph
f____Hyaloscyphaceae	g____Lachnellula	g____Lachnellula Genus	Saprotroph
f____Leucosporidiaceae	g____Leucosporidium	g____Leucosporidium Genus	Saprotroph
f____Melanommataceae	g____Herpotrichia	g____Herpotrichia Genus	Saprotroph
f____Melanommataceae	g____Pleotrichocladiun	s____opacum	Saprotroph
f____Mortierellaceae	g____Gryganskiella	s____cystojenkinii	Saprotroph
f____Mortierellaceae	g____Linnemannia	s____amoeboidea	Saprotroph
f____Mortierellaceae	g____Linnemannia	s____gamsii	Saprotroph
f____Mortierellaceae	g____Linnemannia	s____gamsii	Saprotroph
f____Mortierellaceae	g____Mortierella	s____basiparvispora	Saprotroph
f____Mortierellaceae	g____Mortierella	s____basiparvispora	Saprotroph
f____Mortierellaceae	g____Mortierella	s____basiparvispora	Saprotroph
f____Mortierellaceae	g____Mortierella	s____basiparvispora	Saprotroph
f____Mortierellaceae	g____Mortierella	s____basiparvispora	Saprotroph
f____Mortierellaceae	g____Mortierella	s____basiparvispora	Saprotroph
f____Mortierellaceae	g____Mortierella	s____pseudozygospora	Saprotroph
f____Mortierellaceae	g____Podila	s____humilis	Saprotroph
f____Pseudeurotiaceae	f____Pseudeurotiaceae Family	f____Pseudeurotiaceae Family	Saprotroph
f____Pseudeurotiaceae	f____Pseudeurotiaceae Family	f____Pseudeurotiaceae Family	Saprotroph
f____Pseudeurotiaceae	g____Geomyces	g____Geomyces Genus	Saprotroph
f____Pseudeurotiaceae	g____Geomyces	s____auratus	Saprotroph
f____Pseudeurotiaceae	g____Gymnostellatosp	as____japonica	Saprotroph

Table 1: List of remaining taxa in the Guilds data set, and their assigned guilds.
(continued)

Family	Genus	Species	Guild
f____Pseudeurotiaceae	g____Pseudeurotium	g____Pseudeurotium Genus	Saprotroph
f____Saccharomycetacea	eg____Saccharomyces	s____bayanus	Saprotroph
f____Schizoporaceae	g____Schizoporaceae_ge	ng_hSechizeo_poserdaciseae_ge Genus	nS_alpnrcoetroapeh_sedis
f____Trichocomaceae	g____Sagenomella	s____diversispora	Saprotroph
f____Trichosporonaceae	g____Apiotrichum	s____xylopini	Saprotroph
f____Umbelopsidaceae	g____Umbelopsis	g____Umbelopsis Genus	Saprotroph
f____Umbelopsidaceae	g____Umbelopsis	s____changbaiensis	Saprotroph
f____Umbelopsidaceae	g____Umbelopsis	s____changbaiensis	Saprotroph
f____Umbelopsidaceae	g____Umbelopsis	s____changbaiensis	Saprotroph
f____Umbelopsidaceae	g____Umbelopsis	s____dimorpha	Saprotroph
f____Umbelopsidaceae	g____Umbelopsis	s____isabellina	Saprotroph

6. Taxonomic overview

The filtered phylo3 object contained the following number of taxa and ranks. It must be noted that at almost every taxonomic rank, there were NA values as well as taxa with *Incertae sedis* status. Depending on the specific application, it might be advisable to filter out these instances.

Next, taxonomic lists for each taxonomic rank were extracted for display.

6.1. Overall taxonomic lists

```
tax.lists.unique <- list(Phylum = get_taxa_unique(phylo3, taxonomic.rank = "Phylum"),
        Class = get_taxa_unique(phylo3, taxonomic.rank = "Class"), Order = get_taxa_unique(phylo3, taxonomic.rank = "Order"),
                Family = get_taxa_unique(phylo3, taxonomic.rank = "Family"),
        Genus = get_taxa_unique(phylo3, taxonomic.rank = "Genus"), Species = get_taxa_unique(phylo3, taxonomic.rank =
                "Species"))
```

(Optionally) remove NAvalues, but note this has no effect on the phyloseqobject carried down the pipe. Additional code is needed to filter the phyloseqobject too.

```
tax.lists.unique <- sapply(tax.lists.unique, function(x) { x[!is.na(x)]
})
```

See a list of taxa by rank found in all samples, and a summary of richness by taxonomic rank in Table 2 and Figure 13.

Table 2: Lists of taxa found by taxonomic rank, all samples combined (read each column as an independent table, rows don't necessarily match between columns). * = Incertae sedis status.

Phylum	Class	Order	Family	Genus	Species
Ascomycota	Leotiomycetes	Thelebolales	Pseudeurotiaceae	Pseudogymnoascus	roseus
Mucoromycota	Dothideomycetes	Helotiales	Hyaloscyphaceae	Hyaloscypha	finlandica
Basidiomycota	Eurotiomycetes	Pleosporales	Melanommataceae	Pleotrichocladium	opacum
Mortierellomycota	Umbelopsidomycetes	Eurotiales	Aspergillaceae	Pseudeurotiaceae Family	Pseudeurotiaceae Family
Fungi_phy_*	Tremellomycetes	Umbelopsidales	Umbelopsidaceae	Penicillium	longicatenatum
	Agaricomycetes	Filobasidiales	Piskurozymaceae	Umbelopsis	appendiculatus
	Mortierellomycetes	Chaetothyriales	Helotiales_fam_*	Solicoccozyma	Umbelopsis Genus
	Fungi_cls_*	Agaricales	Trichocomaceae	Humicolopsis	terricola
	Sordariomycetes	Mortierellales	Herpotrichiellaceae	Gymnostellatospora	jamesonlandense
	Pezizomycetes	Leotiomycetes Class	Inocybaceae	Sagenomella	Humicolopsis Genus
	Saccharomycetes	Mytilinidales	Mortierellaceae	Exophiala	japonica
	Ascomycota Phylum	Fungi_ord_*	Leotiomycetes Class	Inocybe	Solicoccozyma Genus
	Microbotryomycetes	Sordariales	Helotiales Order	Podila	diversispora
	Geminibasidiomycetes	Pezizales	Tricholomataceae	Leotiomycetes Class	isabellina
		Saccharomycetales	Myxotrichaceae	Helotiales Order	tremulae
		Hypocreales	Gloniaceae	Tricholoma	Inocybe Genus
		Cantharellales	Leptodontidiaceae	Mortierella	humilis
		Tremellales	Fungi_fam_*	Linnemannia	Leotiomycetes Class
		Chaetosphaeriales	Chaetomiaceae	Oidiodendron	Helotiales Order
		Trichosporonales	Pyronemataceae	Cenococcum	Tricholoma Genus
		Coniochaetales	Saccharomycetaceae	Leptodontidium	Hyaloscypha Genus
		Ascomycota Phylum	Hypocreaceae	Fungi_gen_*	basiparvispora
		Venturiales	Cordycipitaceae	Pseudeurotium	gamsii
		Leucosporidiales	Cortinariaceae	Humicola	Oidiodendron Genus
		Atheliales	Helotiaceae	Hyphodiscus	fuckelii
		Thelephorales	Hydnaceae	Herpotrichia	Cenococcum Genus
		Boletales	Trimorphomycetaceae	Wilcoxina	Leptodontidium Genus
		Tremellodendropsidales	Chaetosphaeriaceae	Saccharomyces	Fungi_gen_* Genus
		Hymenochaetales	Trichosporonaceae	Trichoderma	Pseudeurotium Genus
		Geminibasidiales	Coniochaetaceae	Cordyceps	fuscoatra
		Eurotiomycetes Class	Ascomycota Phylum	Cortinarius	Hyphodiscus Genus
		Sebacinales	Venturiaceae	Helotiaceae Family	Herpotrichia Genus
			Leucosporidiaceae	Clavulina	Wilcoxina Genus
			Chlorociboriaceae	Geomyces	bayanus
			Cephalothecaceae	Hyaloscyphaceae Family	Trichoderma Genus
			Ploettnerulaceae	Saitozyma	amoeboidea
			Eurotiales_fam_*	Chaetosphaeriaceae Family	pseudozygospora
			Pilodermataceae	Apiotrichum	javanica
			Thelephoraceae	Gryganskiella	fuligineoviolaceus
			Sordariales_fam_*	Coniochaeta	sulphureomyceliatus
			Podosporaceae	Ascomycota Phylum	Helotiaceae Family
			Melanogastraceae	Venturia	Clavulina Genus
			Tremellodendropsidales_fam_*	Stamnidium	carneoroseus
			Chaetothyriales_fam_*	Leucosporidium	chlamydosporicum
			Schizoporaceae	Lachnellula	changbaiensis
			Geminibasidiaceae	Herpotrichiellaceae Family	Geomyces Genus
			Lipomycetaceae	Brahmaculus	auratus
			Eurotiomycetes Class	Cephalothecaceae_gen_*	Hyaloscyphaceae Family

Table 2: Lists of taxa found by taxonomic rank, all samples combined (read each column as an independent table, rows don't necessarily match between columns). * = Incertae sedis status. *(continued)*

Phylum	Class	Order	Family	Genus	Species
			Chaetothyriales Order	Cadophora	thomii
			Ophiocordycipitaceae	Eurotiales_gen_*	podzolica
			Amanitaceae	Myxotrichum	Chaetosphaeriaceae Family
			Rickenellaceae	Piloderma	xylopini
			Sebacinaceae	Thelephoraceae Family	cystojenkinii
			Thelebolales Order	Tomentella	dimorpha
				Sordariales_gen_*	Coniochaeta Genus
				Podosporaceae Family	Ascomycota Phylum
				Melanogaster	Venturia Genus
				Tremellodendropsidale	s_vagcecni_ni*i
				Chaetothyriales_gen_*	Leucosporidium Genus
				Schizoporaceae_gen_*	Lachnellula Genus
				Geminibasidium	Herpotrichiellaceae Family
				Lipomyces	magellanicus
				Eurotiomycetes Class	Cephalothecaceae_gen Genus
				Chaetothyriales Order	Cadophora Genus
				Chaetothyriales_fam_ Family	*Cortinarius Genus
				Tolypocladium	Eurotiales_gen_* Genus
				Amanita	cancellatum
				Rickenella	Piloderma Genus
				Hydnum	Thelephoraceae Family
				Cutaneotrichosporon	Tomentella Genus
				Sebacina	Sordariales_gen_* Genus
				Leohumicola	Podosporaceae Family
				Phlegmacium	trabinellum
				Thelebolales Order	macrocarpus
					Tremellodendropsidale Genus
					imbecillis
					Chaetothyriales_gen_ Genus
					Penicillium Genus
					Schizoporaceae_gen_* Genus
					Geminibasidium Genus
					canescens
					lipofer
					Eurotiomycetes Class
					vineae
					Chaetothyriales Order
					Chaetothyriales_fam_ Family
					pilicola
					cylindrosporum
					diemii
					minuta

_*
s_gen_*
*
*

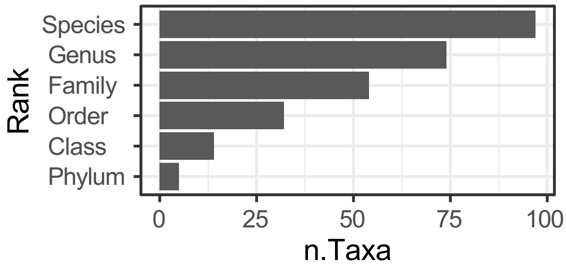

Figure 13: Number of taxa by taxonomic rank.

Table 2: Lists of taxa found by taxonomic rank, all samples combined (read each column as an independent table, rows don't necessarily match between columns). * = Incertae sedis status. *(continued)*

Phylum	Class	Order	Family	Genus	Species
					Hydnum Genus
					debeurmannianum
					Sebacina Genus
					minima
					riopancensis
					punctatisporum
					Thelebolales Order

6.2. *Top taxa only*

Now we can filter the phyloseq object to retain the most abundant taxa (e.g., top 10%).

```
# Filter phyloseq object for the most abundant taxa
perc <- 10

P <- tax_glom(phylo3, "Phylum") C <-
tax_glom(phylo3, "Class") O <-
tax_glom(phylo3, "Order")
F. <- tax_glom(phylo3, "Family") G <-
tax_glom(phylo3, "Genus")
S <- tax_glom(phylo3, "Species")

P.top <- phyloseq_filter_top_taxa(P, perc = perc, n = NULL)
```

Warning in prune_taxa(taxa, phy_tree(x)): prune_taxa attempted to reduce tree to 1 or fewer tips. ## tree replaced with NULL.

```
C.top <- phyloseq_filter_top_taxa(C, perc = perc, n = NULL)
```

Warning in prune_taxa(taxa, phy_tree(x)): prune_taxa attempted to reduce tree to 1 or fewer tips. ## tree replaced with NULL.

```
O.top <- phyloseq_filter_top_taxa(O, perc = perc, n = NULL)
F.top <- phyloseq_filter_top_taxa(F., perc = perc, n = NULL)
G.top <- phyloseq_filter_top_taxa(G, perc = perc, n = NULL) S.top <-
phyloseq_filter_top_taxa(S, perc = perc, n = NULL)
```

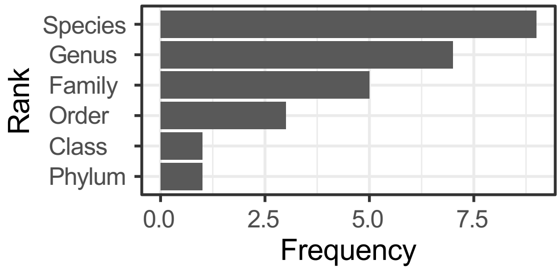

Figure 14: Number of top 10% taxa by taxonomic rank.

```
tax.lists.unique.top <- list(Phylum = get_taxa_unique(P.top, taxonomic.rank = "Phylum"), Class =
    get_taxa_unique(C.top, taxonomic.rank = "Class"), Order = get_taxa_unique(O.top,
        taxonomic.rank = "Order"), Family = get_taxa_unique(F.top, taxonomic.rank = "Family"), Genus =
    get_taxa_unique(G.top, taxonomic.rank = "Genus"), Species = get_taxa_unique(S.top,
        taxonomic.rank = "Species"))
```

See a list of top taxa by rank found in all samples, and a summary of richness by taxonomic rank in Table 3 and Figure 14.

```
tax.lists.unique.top <- lapply(tax.lists.unique.top, function(x) sapply(x, function(y) substring(y, 4)))

max_length <- max(sapply(tax.lists.unique.top, length)) padded_list <-

lapply(tax.lists.unique.top, function(x) {
    length(x) <- max_length
    x[is.na(x)] <- ""  # Replace NA with blank
    x <- gsub("_Incertae_sedis", "_*", x) x
})

df <- as.data.frame(padded_list, stringsAsFactors = FALSE)
```

Table 3: Lists of top 10 % taxa found by taxonomic rank, all samples combined (read each column as an independent table).

Phylum	Class	Order	Family	Genus	Species
Ascomycota	Leotiomycetes	Thelebolales	Pseudeurotiaceae	Pseudogymnoascus	roseus
		Mortierellales	Aspergillaceae	Penicillium	longicatenatum
		Helotiales	Umbelopsidaceae	Umbelopsis	terricola
			Mortierellaceae	Solicoccozyma	jamesonlandense
			Myxotrichaceae	Podila	humilis
				Oidiodendron	Leotiomycetes Class
				Cortinarius	Oidiodendron Genus
					changbaiensis
					Cortinarius Genus

6.3. Plots of composition by taxa

An initial set of visualizations of the microbiome composition by taxa was done by combining (summing) psudo-replicated samples (see the *Pseudoreplicates-aggregated data set* section, above), and then plotting the

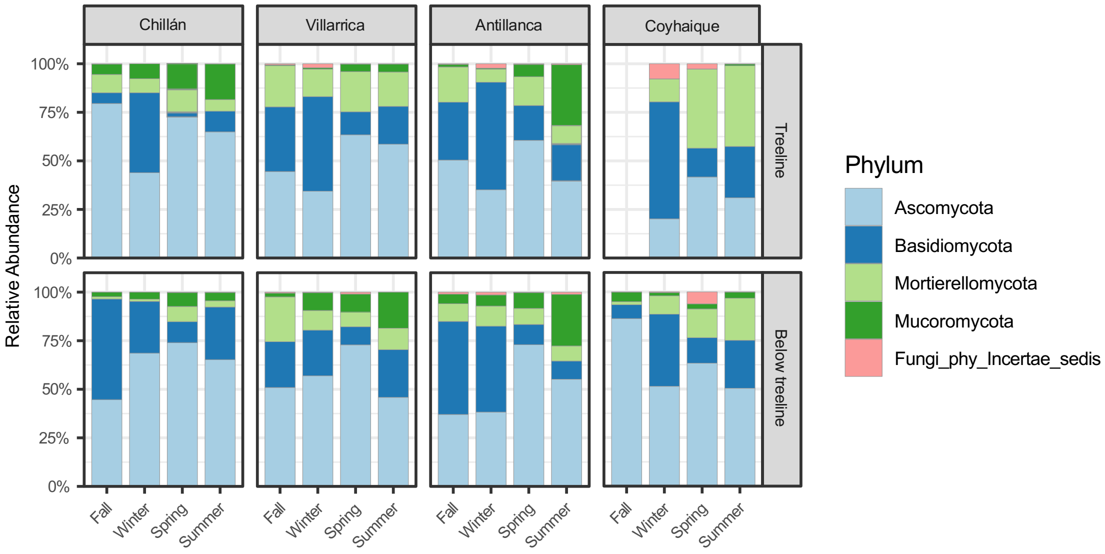

Figure 15: Taxonomic composition the Phylum level.

proportion of each taxon in each of the cells of our experimental design layout. This was repeated for each taxonomic rank from Phylum to Genus, and the 12 most abundant taxa were labelled (Figures 15 and 16.

```
## Registered S3 method overwritten by 'seriation': ##
     method          from
##   reorder.hclust vegan
```

Table 4: Lists of taxa with *Incertae sedis* status (read each column as an independent table).

Phylum	Class	Order	Family	Genus	Species	_* caceae_gen_*
Fungi_phy_*	Fungi_cls_*	Fungi_ord_*	Helotiales_fam_*	Fungi_gen_*	Fungi_gen Genus	gen_*
			Fungi_fam_*	Cephalothecaceae_gen_*	Cephalothe Genus	_gen_*
			Eurotiales_fam_*	Eurotiales_gen_*	Eurotiales_ Genus	ndropsidales_gen_*
			Sordariales_fam_*	Sordariales_gen_*	Sordariales Genus	iales_gen_* ceae_gen_*
			Tremellodendropsidales_fam_*	Tremellodendropsidales_gen_*	Tremellode Genus	iales_fam_*
			Chaetothyriales_fam_*	Chaetothyriales_gen_*	Chaetothyr Genus	
				Schizoporaceae_gen_*	Schizopora Genus	
				Chaetothyriales_fam_* Family	Chaetothyr Family	

6.4. *Inceta sedis*

There are a number of taxa flagged with *incertae sedis* status. Table 4 shows all the *incertae sedis* found in the dataset (read each column as an independent table, rows don't necessarily match between columns). Taxa with *incertae sedis* status were retained in the dataset.

```
# List all cases involving incertae sedis
incerta.cedis <- sapply(tax.lists.unique, function(x) { x[grep("Incertae_sedis", x, ignore.case = T,
      invert = F)]
})
```

7. Data transformations and dissimilarity metrics (for CoDa)

High throughput sequencing data is *compositional data* (CoDa), not abundance data, and requires special handling. There are a number of valid approaches that can render valid transformations and distance metrics necessary for clustering and ordinations. Below, a short overview of a few recommended options (Gloor *et al.* 2017; Quinn *et al.* 2018):

7.1. *Bray-Curtis distance on proportional data*

One simple option is to first transform data to proportions, and then calculate pairwise Bray-Curtis distances. This metric makes sense as it is an asymmetric index that gives double weight to shared taxa's presence and less weight to similarities based on shared taxa's absence (i.e., deals with the double zeroes issue). Ranges between 0 and 1 (Figure 17).

7.2. *Sorensen distance*

Like Bray-Curtis distance but computed on taxa's presence-absence data. Hence, it ignores differences in relative abundance. (Beware of possible discrepancy in the similarity/distance definition of different implementations; might need to get the reciprocal). Ranges between 0 and 1.

7.3. *Hellinger transformation and distance*

The Hellinger distance is the Euclidean distance calculated from the square root of the proportion- transformed data (Hellinger transformation). It conferes very good mathematical properties an allows the application of linear models such as PCoA and db-RDA (Figure 18).

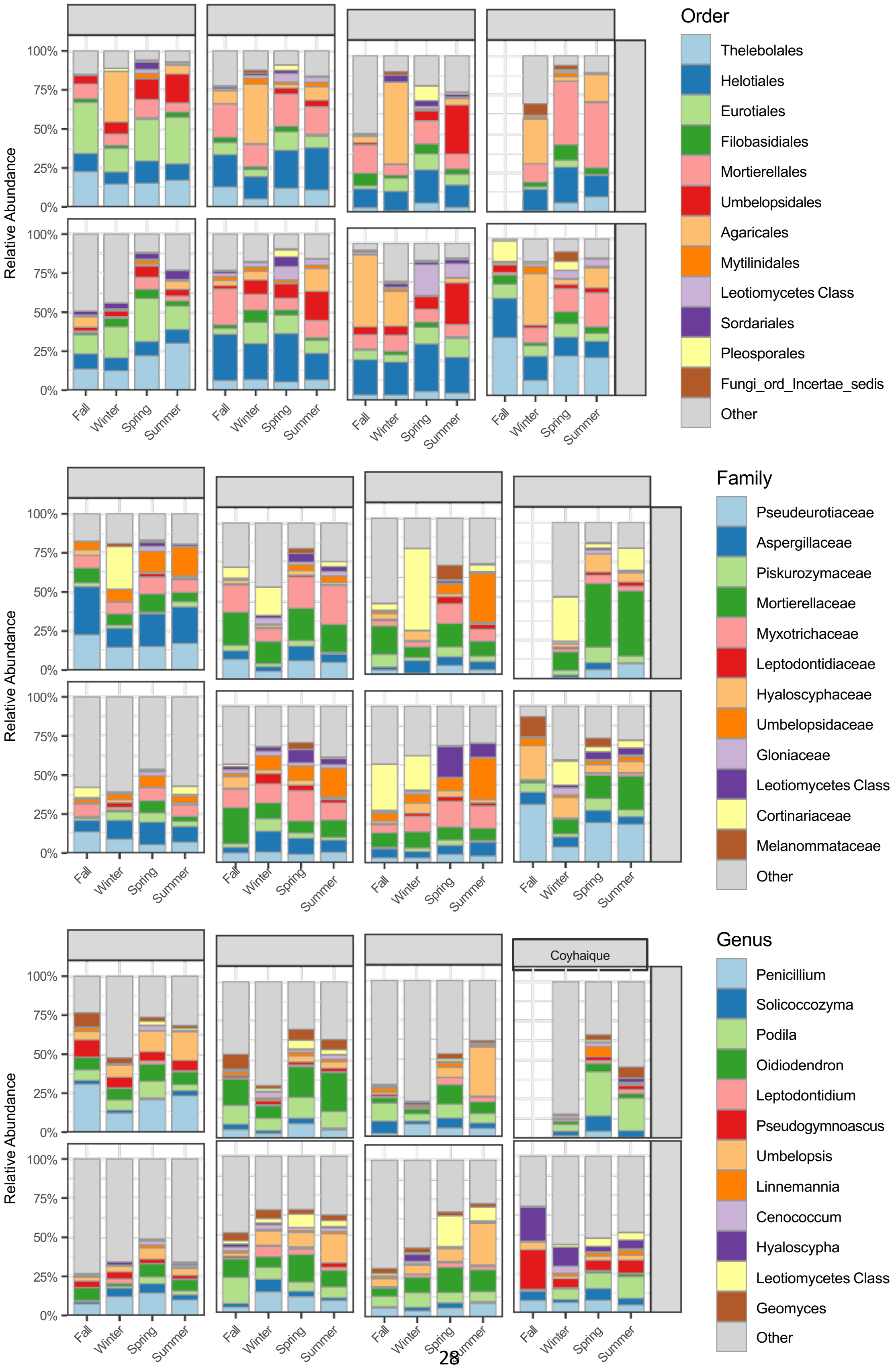

Figure 16: Taxonomic composition of top 12 taxa at the order, family and genus level.

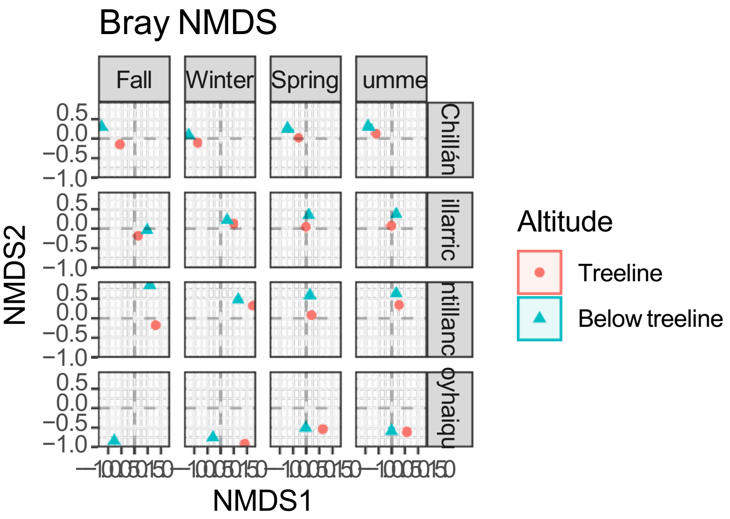

Figure 17: NMDS of phylo3 on Bray-Curtis distances.

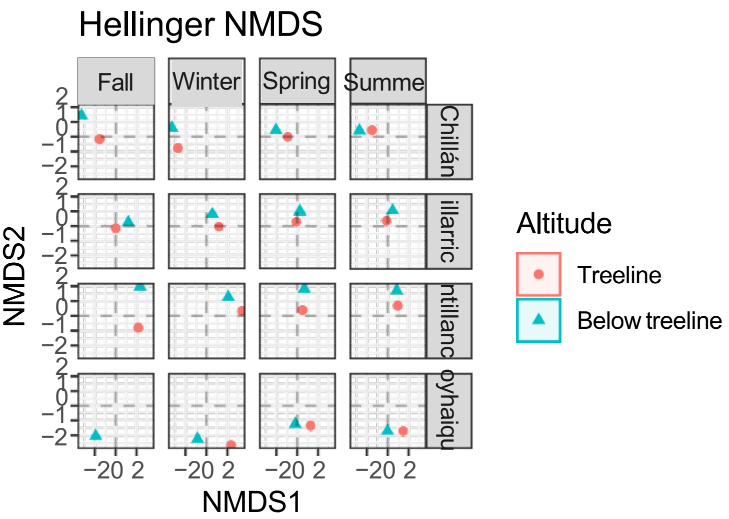

Figure 18: NMDS of phylo3 on Hellinger distances.

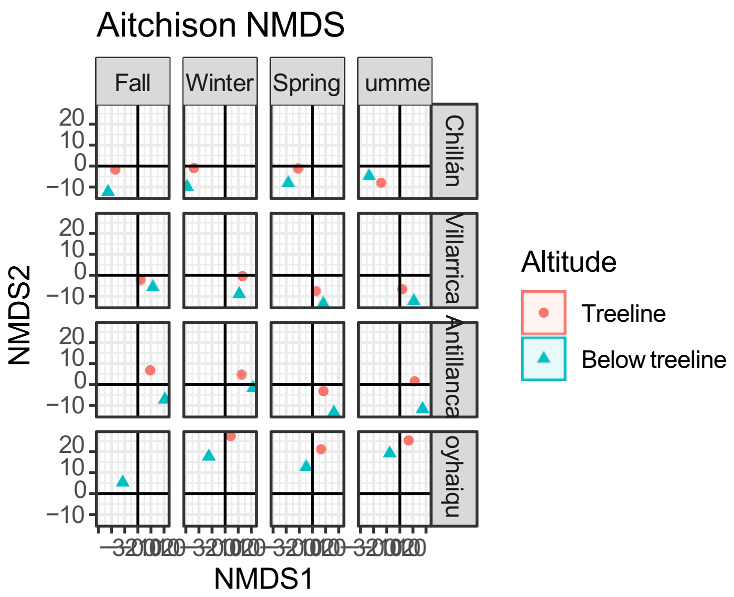

Figure 19: NMDS of phylo3 on Aitchison distances.

7.4. Centered log-ratio (clr) transformation

"Ratio transformations capture the relationships between the features in the dataset and these ratios are the same whether the data are counts or proportions. Taking the logarithm of these ratios, thus log-ratios, makes the data symmetric and linearly related, and places the data in a log-ratio coordinate space" [. . .] and "their sample space is real numbers, and this represents a major advantage for the application of standard statistical methods that have been developed for real random variables" (Gloor *et al.* 2017).

The centered log-ratio (clr) transformation is defined as the natural log of the ratio between the counts of one taxon divided by G(x), the geometric mean of the counts of all taxa. The sum of the components of clr(x) is 0 by definition.

Deal with zeroes before clr-transformation. The G(x) cannot be determined for sparse data without deleting, replacing or estimating the 0 count values. Function microbiome::transform(phylo3, 'clr') adopted here applies a pseudocount of min(relative abundance)/2 to exact zero relative abundance entries in OTU table before taking logs. Other methods for dealing with 0 count values exist (Gloor *et al.* 2017).

```
# Example (not run), outputs count transformation within phyoseq object
phylo3.clr <- microbiome::transform(phylo3, "clr") # Note that small pseudo count is added if data c

# Check zero-sum property.
all(round(apply(otu_table(phylo3.clr), 1, sum), 2) == 0)
```

```
## [1] TRUE
```

7.5. Aitchison distance

Euclidean distance between samples after centered log-ratio (clr) transformation. The Aitchison distance is superior to both the widely used Jensen-Shannon divergence and the Bray-Curtis dissimilarity metrics, being more stable to subsetting (Figure 19).

The above indices ignore any phylogenetic information in the data. Different taxa are treated the same regardless of their phylogenetic relatedness. Below an alternative that includes phylogenetic information in the community distance metric.

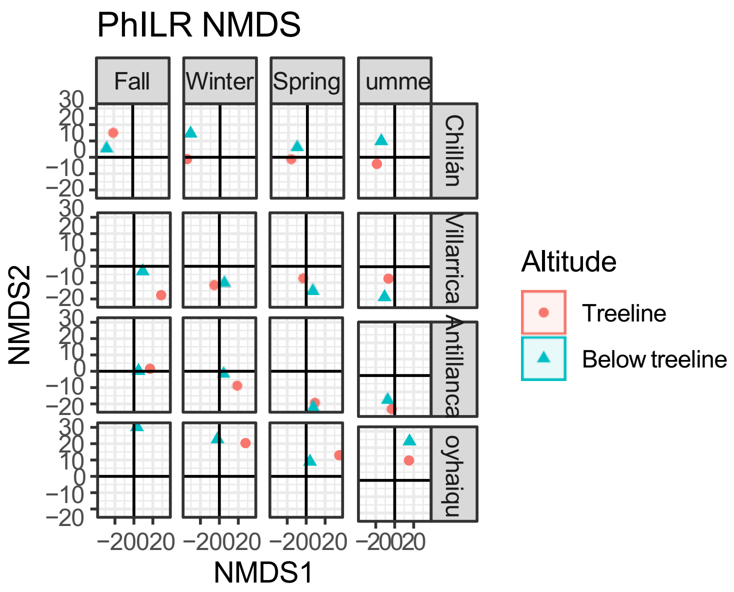

Figure 20: NMDS of phylo3 on Phylogenetic Isometric Log-Ratio (PhILR) Transform based Euclidea distance

7.6. PhILR transformation

Phylogenetic Isometric Log-Ratio Transform (PhILR transform and R packege; Silverman et al. 2017).This transformation is based on balances (binary partitions) along an evolutionary tree (akin and can replace UniFrac distance metric). Distances determined by phylogenetic transforms have the advantage that the binary partitions chosen have a simple interpretation and the correlation structure of the data is fully accounted for (20). Zero values must be removed either through use of pseudocount, multiplicative replacement or (https://www.bioconductor.org/packages/devel/bioc/vignettes/philr/inst/doc/philr-intro.html)

The function philr::philr() implements a user friendly wrapper for the key steps in the philr transform.

1. Convert the phylogenetic tree to its sequential binary partition (SBP) representation using the function philr::phylo2sbp() 2. Calculate the weighting of the taxa (aka parts) or use the user specified weights 3. Built the contrast matrix from the SBP and taxa weights using the function philr::buildilrBasep() 4. Convert OTU table to relative abundance (using philr::miniclo()) and 'shift' dataset using the weightings (Egozcue and Pawlowsky-Glahn 2016) using the function philr::shiftp(). 5. Transform the data to PhILR space using the function philr::ilrp() 6. (Optional) Weight the resulting PhILR space using phylogenetic distance. These weights are either provided by the user or can be calculated by the function philr::calculate.blw().

Building Sequential Binary Partition from Tree... ## Building

Contrast Matrix...

Transforming the Data...

Calculating ILR Weights...

8. Unconstrained ordination

8.1. Transformation-based PCA (tb-PCA) of taxonomic groups

Same as principal coordinates analysis (PCoA) which is a PCA on a distance metric different from Euclidean. Here we used method PCA to accomplish PCoA because methods PCA calculated Euclidean distance on our Hellinger-transformed data effectively rendering a PCoA on Hellinger distances.

Unconstrained ordination through tb-PCA showed an apparent structure in the data related to location and altitude (Figures 21 to 23). Note how the third axis of variation reveals substantial structure coherent with our experimental design.

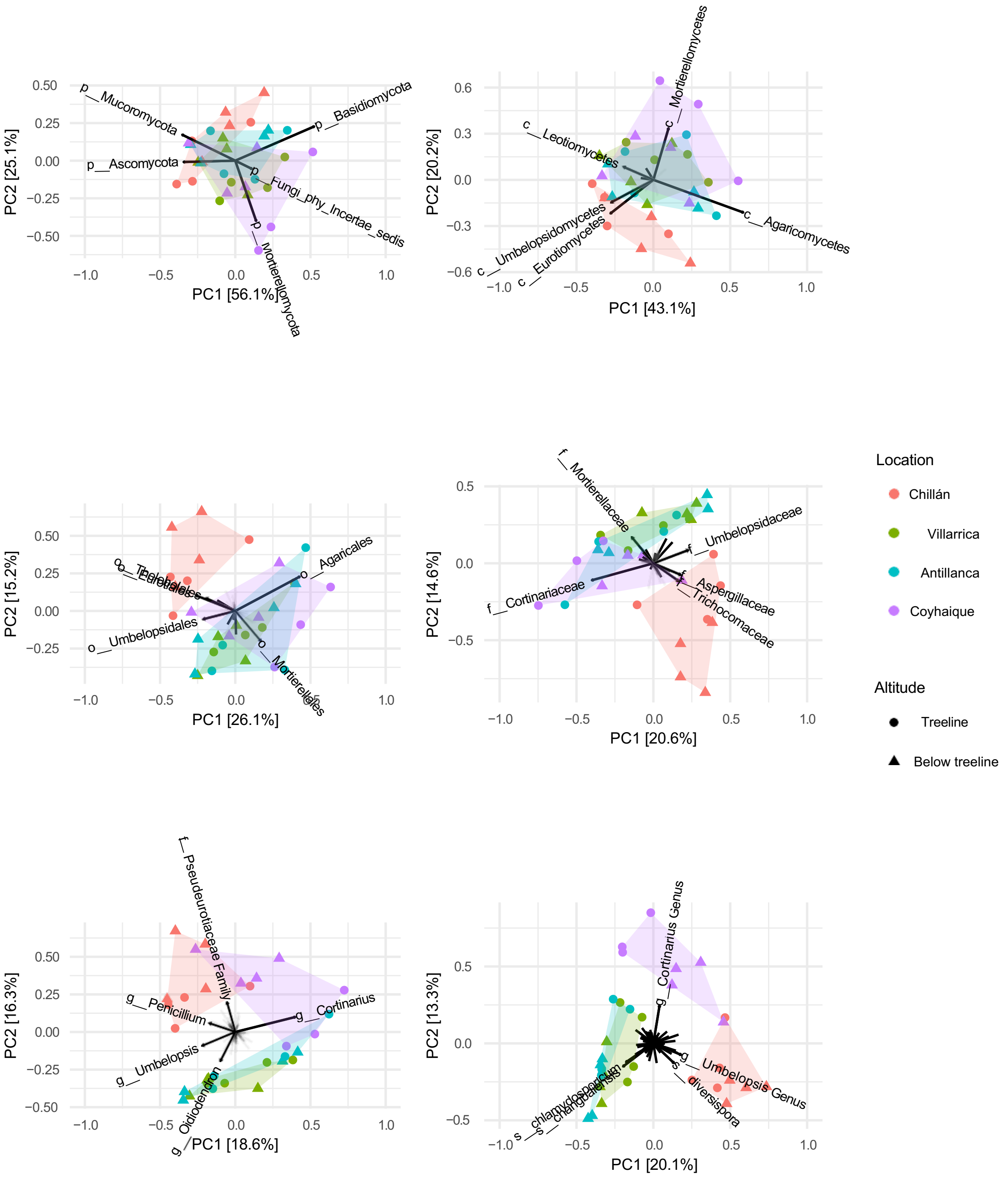

Figure 21: Hellinger transformation-based principal component analysis (tb-PCA) on the pseudoreplicates-aggragated data set. Each panel displays the tb-PCA with data further aggregated by taxonomic rank, from Phylum (top-left) to Species (bottom-right). For clarity, only the top-five taxa with higher loadings were labeled.

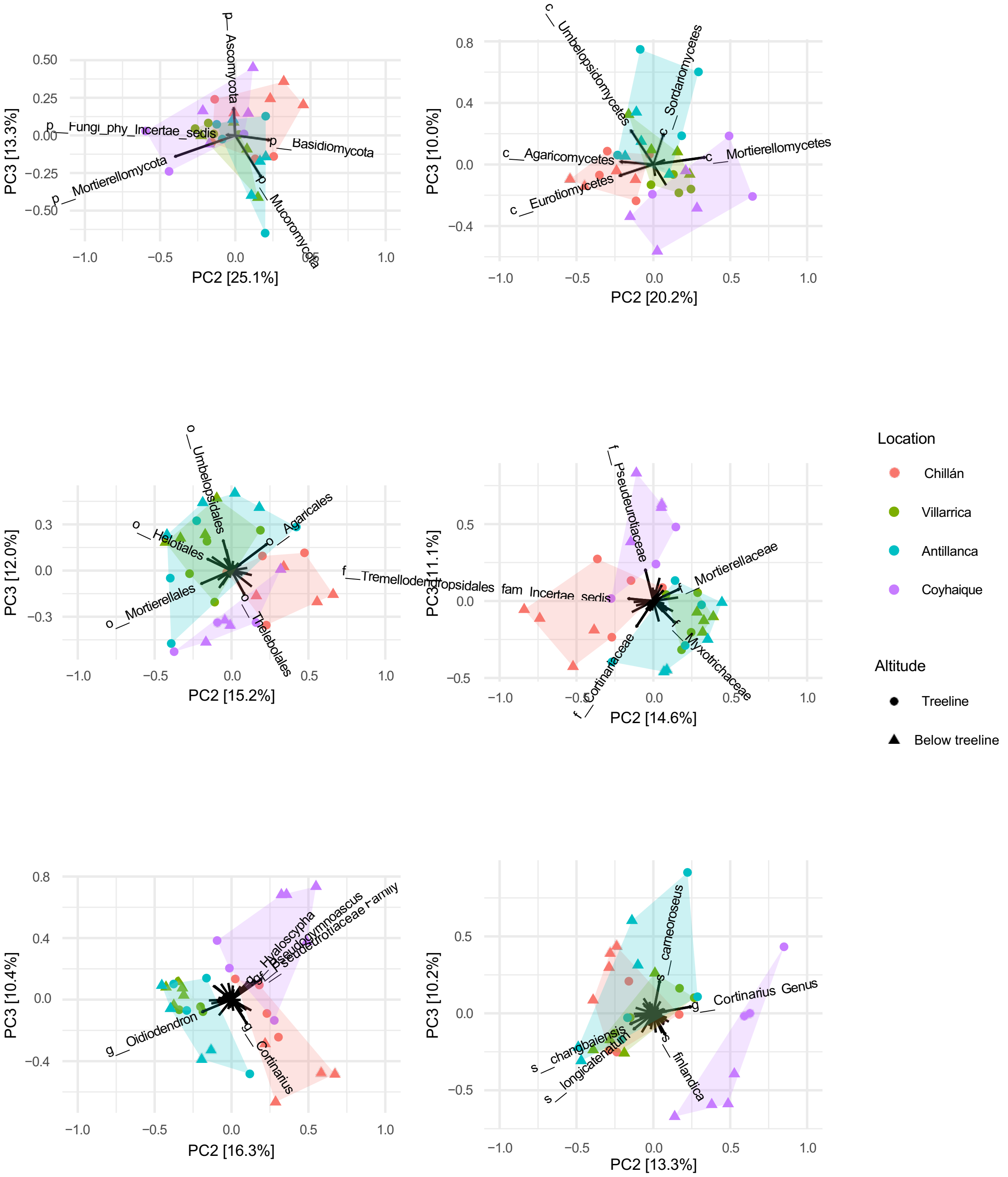

Figure 22: Similar to Figure 21 but plotting canonical axes 2 and 3.

8.2. Transformation-based PCA (tb-PCA) of fungi guilds

Because so many ASVs could not be associated to a guild, guild ordinations or direct gradient analyses were conducted with a filtered data set where ASVs with no associated guild were removed (see Figure 8)). The resulting filtered data set was then aggregated by pooling (adding reads) ASVs from the same guild (7 guilds), transformed (Hellinger and analyzed through transformation-based principal component analysis (tb-PCA; Figures 25 and 26).

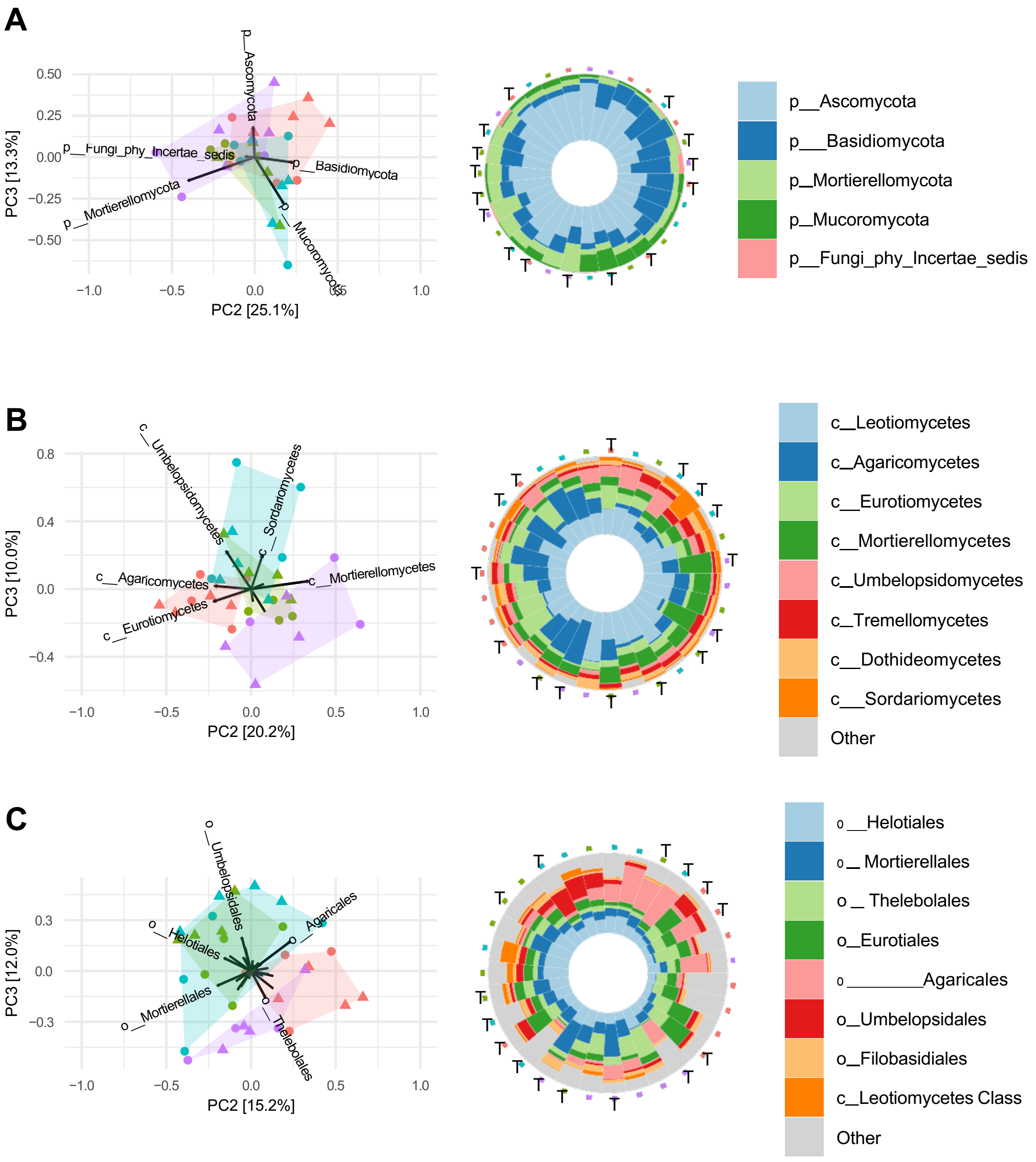

Figure 23: Similar to Figure 22 but adding an iris plot to aid interpretation. Peripheral tick marks on the irisplots indicate whether the sample was taken at the treeline (T) and the location (color-coded as in 22).

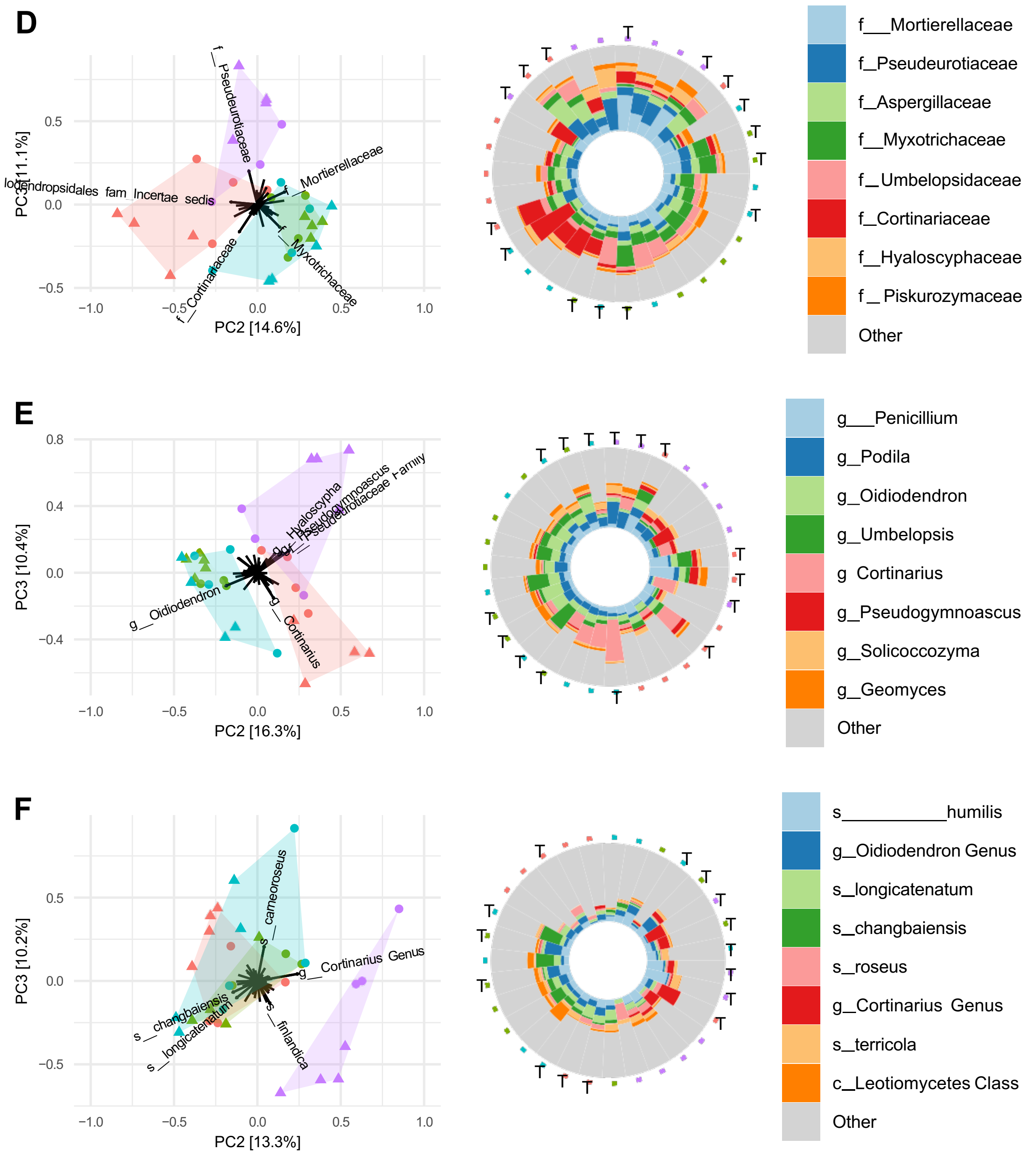

Figure 24: Continuation of Figure 23.

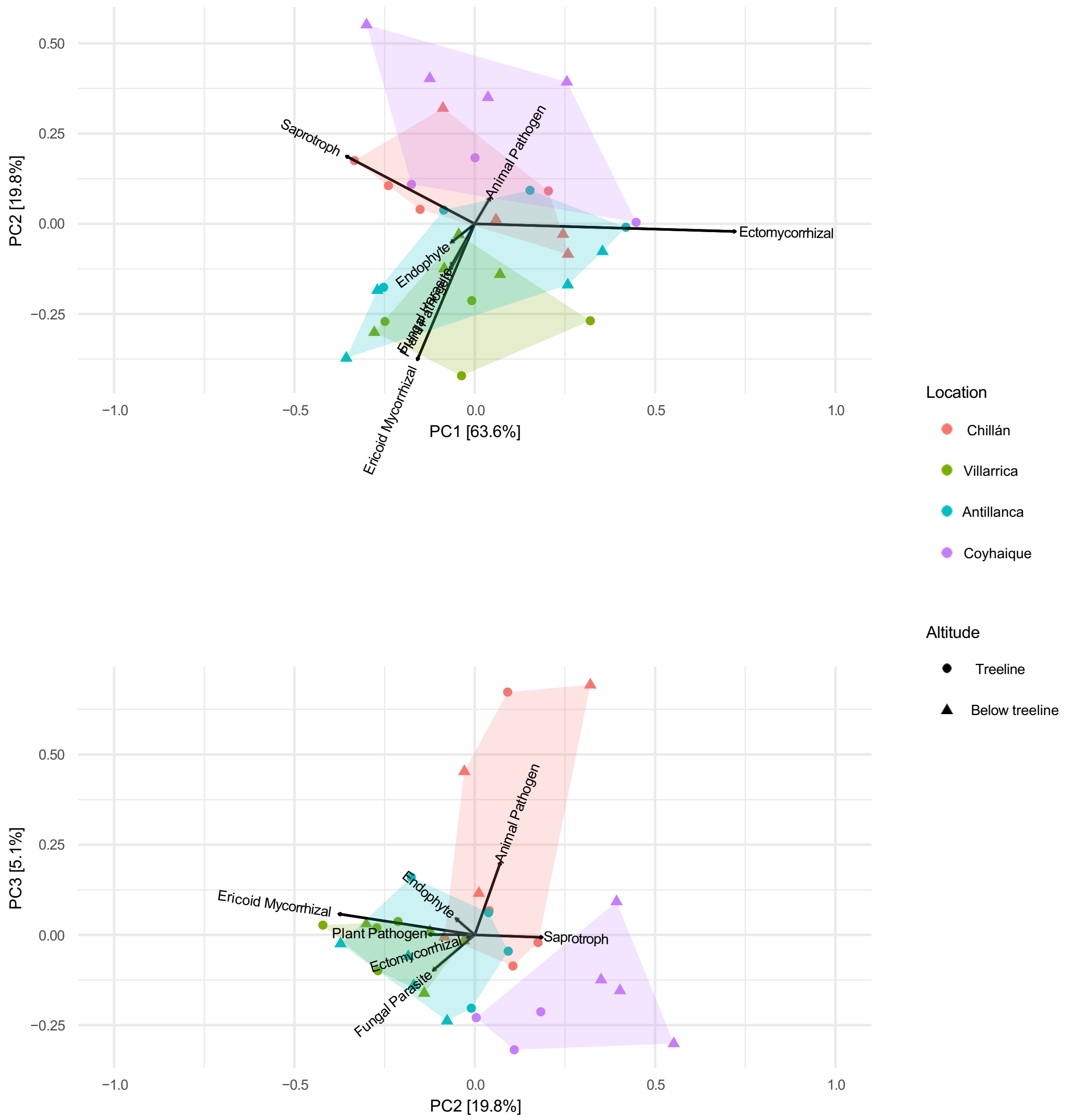

Figure 25: Hellinger transformation-based principal component analysis (tb-PCA) of the Guilds data set. The first panel shows canonical axes 1 and 2, and thecond, 2 and 3.

9. Constrained ordination: transformation-based redundancy analysis (tb-RDA)

A type of direct gradient analysis. Since we are analyzing community composition data along a presumably short ecological gradient, an appropriate analytic sequence is (i) compute an appropriate distance matrix, (ii) calculate PCoA (metric scaling), and use the resulting scores to, (iii) run an RDA. This is called a db-RDA, and can be done step-by-step (not shown) or through shortcut function cmdscale{vegan}. For longer, unimodal gradient analysis, use Canonical Correspondence Analysis (CCA) since RDA is a linear model. Note: After preliminary analyses, we decided to analyze the speudoreplicates-aggregated dataset. There are a number of reasons for this:

1. Pseudoreplicates involved three samples collected at the same soil parcel, influenced by the same landscape and edaphic variables.
2. Community composition patterns were clearer and more consistent when pseudoreplicates were pooled together.
3. Time series required sample units (sites) observed at different points in time. Pseudoreplicates were haphazard within sites and were no tracked in time individually. Time series were used to conduct model- based imputation of missing data, and for restricted PERMANOVA as an argument to the *how()* function.
4. Variation across pseudoreplicates (small-scale spatial heterogeneity and/or sampling error), which was not the focus of this study, was substantial, and hinders patterns of interest (e.g., see Figure 9).
5. Main gradient length estimation by Detrended Correspondence Analysis was substantially shorter after pooling pseudoreplicates (from about 5 SD to 3 SD).

9.1. Main gradint length estimation by Detrended Correspondence Analysis (DCA)

To decide between linear and unimodal ordination methods for your data, the rule of thumb of Lepš & Šmilauer's (2003) can be used: Run Detrended Correspondence Analysis (DCA) and check the *first axis* length in standard deviation (SD) units. If >4 SD, use unimodal methods for heterogeneous data; if <3 SD, linear methods suit homogeneous data. Between 3 and 4 SD, either method works. While linear methods are ineffective for heterogeneous data, unimodal methods can be used for homogeneous data but linear methods are preferred. For heterogeneous data requiring linear ordination (like PCA, RDA), use Hellinger transformed species composition to calculate ordinations, as suggested by Legendre & Gallagher (2001).

```
library(vegan)
```

```
## Loading required package: permute

## Loading required package: lattice ## This is

vegan 2.6-4
```

```
# Hellinger transform
phylo3.hell <- transform_sample_counts(phylo3, function(x) sqrt(x/sum(x)))

# Check the length of the main gradient.
vegan::decorana(otu_table(phylo3.hell))
```

```
##
## Call:
## vegan::decorana(veg = otu_table(phylo3.hell)) ##
## Detrended correspondence analysis with 26 segments. ## Rescaling
of axes with 4 iterations.
## Total inertia (scaled Chi-square): 2.8279 ##
```

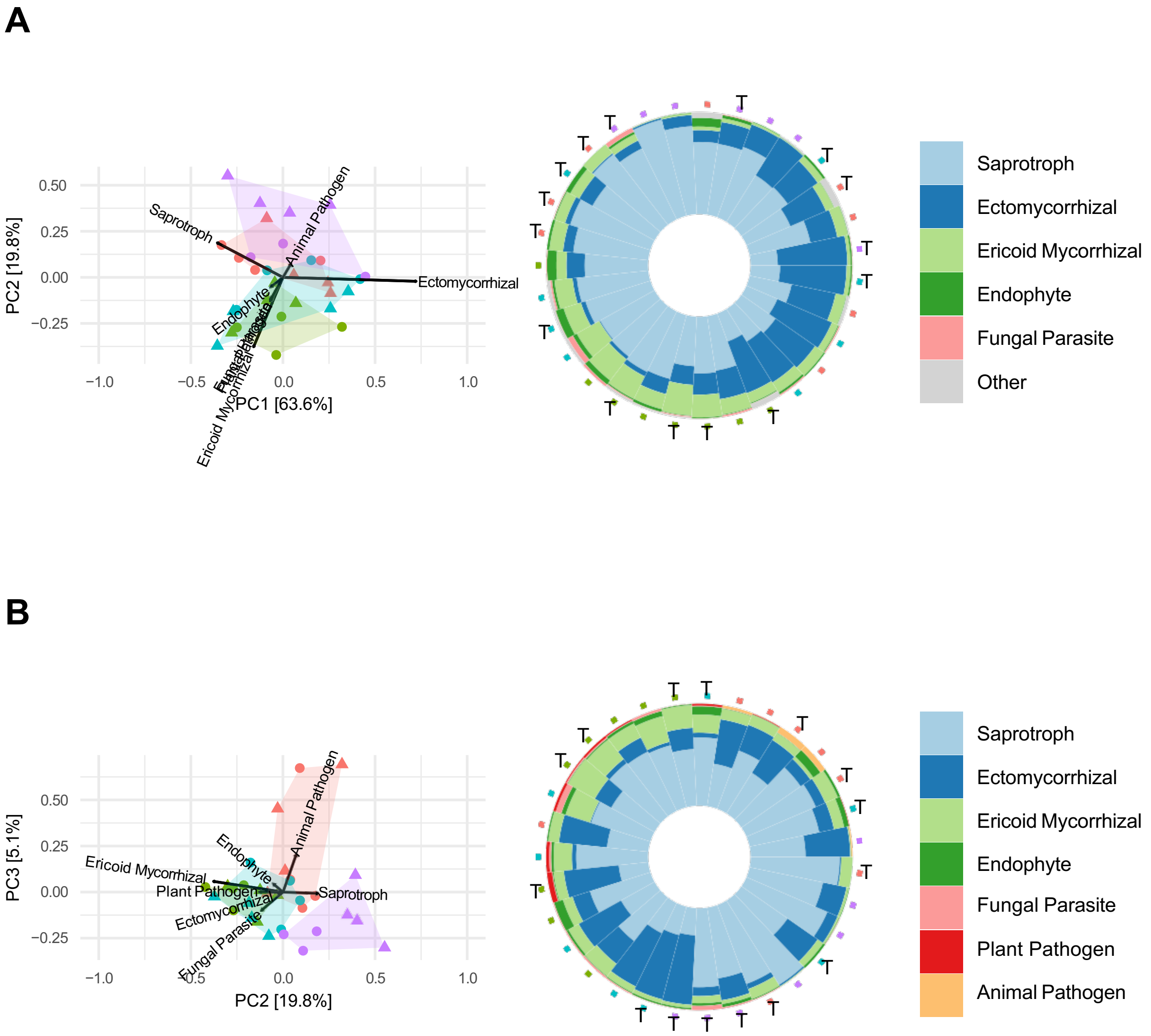

Figure 26: Similar to Figure 25 but adding an iris plot to aid interpretation. Peripheral tick marks on the irisplots indicate whether the sample was taken at the treeline (T) and the location (color-coded as in Figure 25). Panels A and B show different combinations of canonical axes.

```
##                           DCA1    DCA2    DCA3     DCA4
## Eigenvalues              0.4016  0.2615  0.1047  0.14668
## Additive Eigenvalues     0.4016  0.2610  0.1041  0.14699
## Decorana values          0.4118  0.2436  0.1185  0.09062
## Axis lengths             2.9304  2.2883  1.8805  2.05467
```

The length of our *first* DCA axis was 2.93 SD, and we therefore proceeded with linear constrained ordination.

9.2. Data structure and randomization schema

Our study employs a transformation-based redundancy analysis (tb-RDA) to elucidate the composition of fungal communities. This analysis is structured around three primary variables determined by our experimental design: Location, Altitude (nested within Location), and Season (nested within the interaction of Location and Altitude). The nesting of these factors is crucial, as it reflects our study's hierarchical structure. Any reference null model used for hypothesis-testing needs to take into account this hierarchical aspect in the structure of the data. Likewise, the effect of season (time series) need to consider temporal autocorrelation in the structure of the data. Failure to properly incorporate the appropriate data structure in hypothesis testing routines would likely inflate *Type I* error rates, as observed data would render improbable under the wrong (default) completely randomized null model of reference. The above consideration were coded using the how{permute} function.

One nuisance that had to be dealt with first, however, was the unbalance in our data set due to the lack of samples from Coyhaique in the fall. The permutation tests methods used did not allow such unbalance. To balance the data set, we trimmed it by excluding all fall samples. This was a conservative choice because by reducing the data set size (about one forth less observations overall, and three seasons instead of four for testing seasonal effects) the statistical power was expected to decrease (increasing *Type II* error rate). If the effects of Altitude and Season were nonetheless significant, this would indicate a strong signal. Though statistical significance through permutation tests was based on this reduced data set, visualizations for illustration of results used an enhanced data set, where missing data was filled-in with model-based imputed values.

```
# Filtered out fall samples to eliminate imbalance
phylo3.hell.fallout <- subset_samples(phylo3.hell, !(Season %in% "Fall"))

# Created permutation object
set.perm <-
  how(within = Within(type = "series", constant = T, mirror = F), # for time series
      plots = Plots(strata = sample_data(phylo3.hell.fallout)$Altitude, type = "free"),
      blocks = sample_data(phylo3.hell.fallout)$Location,   # for no permutations among blocks
      observed = T,  # include the observed values
      complete = T) # returns all permutations
```

9.3. Full db-RDA model

The following distance-based redundancy analysis (tb-RDA) was fitted to the data. Initially, all relevant variables were included in the model (Latitude, Season, Altitude, T_min, T_mean, T_max), except for Location which was nonetheless included in the permutation scheme (see above, *Data structure and randomization schema*), and all soil variables which were only available for the summer season (analyzed separately, below).

```
full <- vegan::dbrda(otu_table(phylo3.hell.fallout) ~
                Latitude +
                Season +
```

```
                    Altitude + Season:Altitude+
                    T_min+
                    T_mean
                    + T_max,
                    # Organic_matter + Nitrogen + Phosphorus + Sulfur, # Soil variables are for the Summer on
                  data = data.frame(sample_data(phylo3.hell.fallout)), add = "lingoes",
                  # to correct for negative eigenvalues
                  comm = otu_table(phylo3.hell.fallout)) # to produce spp scores

full
```

```
## Call: vegan::dbrda(formula = otu_table(phylo3.hell.fallout) ~ Latitude ## + Season + Altitude
+ Season:Altitude + T_min + T_mean + T_max, data = ##
data.frame(sample_data(phylo3.hell.fallout)), add = "lingoes", comm = ##
otu_table(phylo3.hell.fallout))
##
##                Inertia Proportion Rank
## Total           12.982      1.000
## Constrained      6.881      0.530     9
## Unconstrained    6.101      0.470    14
## Inertia is squared Euclidean distance
##
## Eigenvalues for constrained axes:
## dbRDA1 dbRDA2 dbRDA3 dbRDA4 dbRDA5 dbRDA6 dbRDA7 dbRDA8 dbRDA9
## 2.0660 1.5574 0.9377 0.7185 0.4995 0.3689 0.3121 0.2805 0.1400
##
## Eigenvalues for unconstrained axes:
##   MDS1   MDS2   MDS3   MDS4   MDS5   MDS6   MDS7   MDS8   MDS9  MDS10  MDS11
## 1.4380 1.0204 0.6490 0.5328 0.4625 0.3990 0.3414 0.3295 0.2401 0.1949 0.1449
##  MDS12  MDS13  MDS14
## 0.1401 0.1215 0.0870
```

```
# summary(full) # Additional information and scores full$tot.chi # total
# variance, constrained and unconstrained full$CCA$tot.chi # variance explained # by constraints
full$CA$tot.chi # remaining unconstrained variance
```

Firstly, the results indicate all constraining variables together may explain up to 53 % of the variation in the fungi community data, and the first two constraining axes explain 27.9 %. The remaining 47 % of the variation cannot be explained by the explanatory matrix (unconstrained). Therefore, roughly half of the observed variation was the result of other environmental or stochastic processes, and this needs to be taken into account in all subsequent interpretations and visualizations presented below.

Of course some constraining variables in this full model were expected to be somewhat redundant and correlated with each other (Figure 27).

Correlated variables exacerbated the variance of model coefficients, which is undesirable. This effect was evidenced by variance inflation factors (VIF) that express how much the variance of regression coefficients is inflated by the presence of covariates. VIF increase as variables are correlated with each other.

VIF >= 20 indicate strong collinearity in constraining variables.

VIF >=10 potentially of concern and should be looked at.

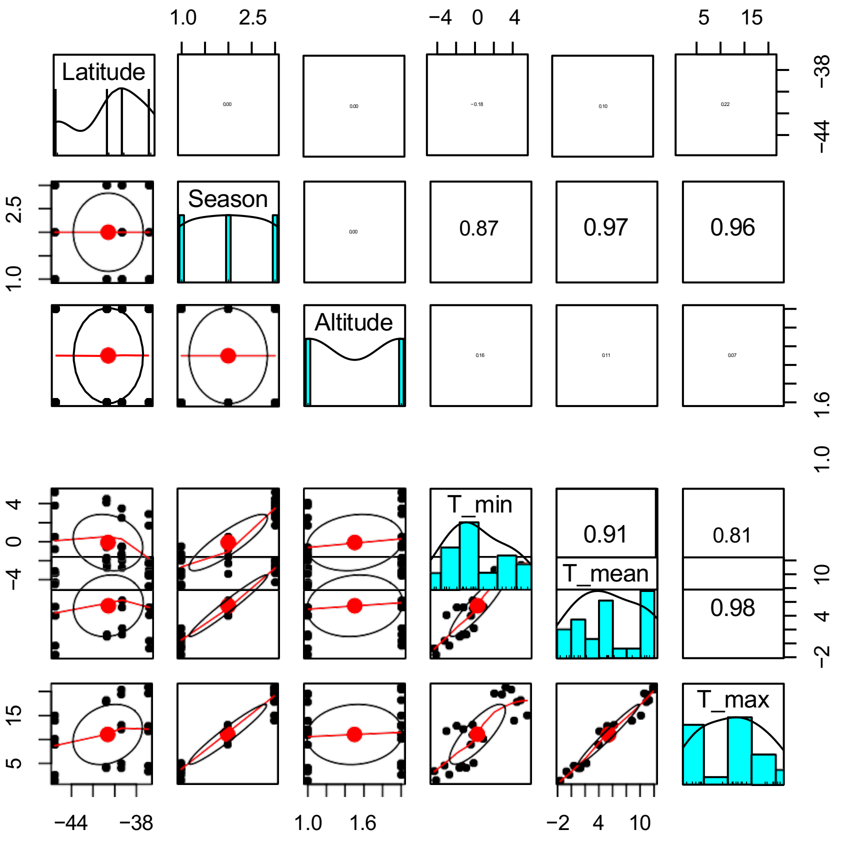

Figure 27: Pairwise correlation plot between constrainig variables before model selection.

```
vif.cca(full)
```

```
##                                  Latitude                          SeasonSpring
##                                  4.442008                             22.126720
##                             SeasonSummer                 AltitudeBelow treeline
##                                 73.672412                              3.568999
##                                     T_min                                T_mean
##                               6141.049584                          53870.935303
##                                     T_max      SeasonSpring:AltitudeBelow  treeline
##                              26921.565610                              4.317169
##       SeasonSummer:AltitudeBelow  treeline
##                                  3.885325
```

Thus, at least the temperature variables are of special concern, and to a lesser degree season. It should be noted that latitude, altitude and season are (functionally) related to temperature and should be correlated with it to different degrees. Latitude, altitude and season also embody other ecological processes and environmental variation so may provide additional insights into the fungi community composition. Model selection (below) will help elucidate which variables best explain the fungi community composition, but first a few additional statistical analyses of the full model.

9.3.1. Tests of significance

The tests of significance were done through Permutational Multivariate Analyses of Variance (PER- MANOVAs), imposing the restrictions to the permutation schema as described earlier.

A few complementary tests revealed different statistical properties of the fitted model. For example, a test of the significance of the constrained axes suggested that only the first axis was statistically significant (P < 0.05).

```
# Test the significance of the axes
perm <- anova.cca(full, by = "axis", permutations = set.perm)
```

```
## Set of permutations < 'minperm'. Generating entire set.
```

```
# Estimated the standardized effect sizes (SES) and confidence intervals
pstat <- permustats(perm)
summary(pstat, interval = 0.95)
```

```
##
##          statistic     SES    mean  lower   median   upper  Pr(perm)
##   dbRDA1    4.7407  2.0371  4.3336          4.3125  4.7018   0.03624 *
##   dbRDA2    3.5735  0.5200  3.3817          3.3702  3.9928   0.30609
##  dbRDA3     2.1517  0.5359  1.9707          1.9487  2.5128   0.32074
##   dbRDA4    1.6488 -0.0252  1.6565          1.6167  2.2830   0.45258
##  dbRDA5     1.1460 -0.7103  1.2960          1.2813  1.6781   0.75867
##   dbRDA6    0.8466 -1.1875  1.0666          1.0442  1.4144   0.91750
##   dbRDA7    0.7162 -1.5121  1.0093          0.9921  1.3632   0.96222
##   dbRDA8    0.6436 -0.9058  0.8380          0.8110  1.2456   0.84503
##___dbRDA9    0.3214 -1.4712  0.6012          0.6091  0.9038   0.94449
## Signif. codes:  0 '***' 0.001 '**' 0.01 '*' 0.05 '.' 0.1 ' ' 1 ##
## (Interval (Upper - Lower) = 0.95)
```

Only the first constraining axis was significant.

The marginal effect of each model term while controlling for the effects of all others:

```
# Significance tests for each marginal term in a model with all other terms # included (order does not matter).
perm <- anova.cca(full, by = "margin", scope = formula(full), permutations = set.perm)
```

Set of permutations < 'minperm'. Generating entire set.

```
perm
```

```
## Permutation test for dbrda under reduced model ##
Marginal effects of terms
## Blocks:  sample_data(phylo3.hell.fallout)$Location
## Plots: sample_data(phylo3.hell.fallout)$Altitude, plot permutation: free ## Permutation: series
constant permutation within each Plot
## Number of permutations: 1296 ##
## Model: vegan::dbrda(formula = otu_table(phylo3.hell.fallout) ~ Latitude + Season + Altitude + Seas ##  Df SumOfSqs      F Pr(>F)
##  Latitude          1    0.6696  1.5364  0.72321
##  T_min             1    0.5238  1.2019  0.17425
##  T_mean            1    0.5128  1.1768  0.18967
##  T_max             1    0.5220  1.1978  0.17887
##  Season:Altitude   2    0.6081  0.6977  0.03392 *
##  Residual         14    6.1012
##  ---
## Signif. codes:  0 '***' 0.001 '**' 0.01 '*' 0.05 '.' 0.1 ' ' 1
```

Only Season and Altitude (Season:Altitude) were significant at explaining the fungi community composi- tion, conditional on the full model, as inferred from PERMANOVA of the marginal effect of each model term while controlling for the effects of all others ($P < 0.05$). Because the high VIF as described above, model selection might reveal other variables as important too.

9.4. Model selection

Model selection was carried out to simplify the full model into a more parsimonious, less redundant model. The function vegan::ordistep() was used, with bidirectional (default) stepwise search of the best set of explanatory variables, and the permutation scheme described above.

```
best <- vegan::ordistep(full, direction = "both", permutations = set.perm, trace = F) best
```

```
## Call: vegan::dbrda(formula = otu_table(phylo3.hell.fallout) ~ Season + ## Altitude + T_max +
Season:Altitude, data =
## data.frame(sample_data(phylo3.hell.fallout)), add = "lingoes", comm = ##
otu_table(phylo3.hell.fallout))
##
##                   Inertia Proportion Rank
##  Total            12.9819     1.0000
##  Constrained       4.2637     0.3284    6
##  Unconstrained     8.7183     0.6716   17
## Inertia is squared Euclidean distance
```

```
##
## Eigenvalues for constrained axes:
## dbRDA1 dbRDA2 dbRDA3 dbRDA4 dbRDA5 dbRDA6
## 1.6916 0.9564 0.7445 0.3805 0.3388 0.1519 ##
## Eigenvalues for unconstrained axes:
##    MDS1   MDS2   MDS3   MDS4   MDS5   MDS6   MDS7
     MDS8 ## 2.4420 1.0544 0.9173 0.7703 0.6077 0.5011 0.4267 0.3454
## (Showing 8 of 17 unconstrained eigenvalues)
```

Following bidirectional stepwise model selection, we concluded that the best model explaining the fungi community composition included Season, Altitude, T_max, and Season:Altitude. Again, even though Location was not included in the model, it was considered in the permutation scheme as a key component of the experimental design.

The best tb-RDA model explained 32.8 % of the variance (R^2), though penalizing for the number of predictors the proportion drops to 9.1 % (i.e., Adj. R^2).

All constraining variables together may explain up to 32.8 % of the variation in the fungi community data, and the first two constraining axes explain 20.4 %. The remaining 67.2 % of the variation cannot be explained by the explanatory matrix (unconstrained). Therefore, most of the observed variation was the result of other environmental or stochastic processes, and this needs to be taken into account in all subsequent interpretations and visualizations presented below.

Variance inflation factors (VIF) were in the range 3.08, 20.67 in the final model indicating some collinearity among explanatory variables.

```
vif.cca(best)
```

```
##                         SeasonSpring                         SeasonSummer
##                             7.777590                            20.673544
##                AltitudeBelow treeline                                T_max
##                             3.082440                            14.624051
## SeasonSpring:AltitudeBelow treeline SeasonSummer:AltitudeBelow treeline ##     3.333634
                             3.333367
```

It might be that SeasonSummer and T_max are interfering with each other.

9.4.1. Tests of significance

The tests of significance were done through Permutational Multivariate Analyses of Variance (PER- MANOVAs), imposing the restrictions to the permutation schema as described earlier.

A few complementary tests revealed different statistical properties of the fitted model. For example, a test of the significance of the constrained axes:

```
# Test the significance of the axes
perm <- anova.cca(best, by = "axis", permutations = set.perm)
```

```
## Set of permutations < 'minperm'. Generating entire set.
```

```
# Estimated the standardized effect sizes (SES) and confidence intervals
pstat <- permustats(perm)
summary(pstat, interval = 0.95)
```

```
##
##              statistic        SES      mean lower      median      upper Pr(perm)
##  dbRDA1     3.2985     2.4366   2.9101              2.9024    3.1890 0.003084 **
##  dbRDA2     1.8649     1.9147   1.4268              1.4289    1.8216 0.036237 *
##  dbRDA3     1.4516     1.3755   1.2003              1.2045    1.5056 0.082498 .
##  dbRDA4     0.7419    -0.0767   0.7526              0.7246    1.0349 0.450270
##  dbRDA5     0.6607     0.2223   0.6315              0.6188    0.8852 0.356978
##  dbRDA6     0.2963    -1.0224   0.4339              0.4391    0.6448 0.847340
##  ---
## Signif. codes:  0 '***' 0.001 '**' 0.01 '*' 0.05 '.' 0.1 ' ' 1 ##
## (Interval (Upper - Lower) = 0.95)
```

The first two constraining axes were statistically significant (P < 0.05).

PERMANOVA of the marginal effect of each model term while controlling for the effects of all others indicated that all model terms of the best model were statistically significant (P < 0.05; Figures 28 and 29).

```
# Significance tests for each marginal term in a model with all other terms # included (order does not matter).
perm <- anova.cca(best, by = "margin", scope = formula(best), permutations = set.perm) perm
```

```
## Permutation test for dbrda under reduced model ##
Marginal effects of terms
## Blocks:  sample_data(phylo3.hell.fallout)$Location
## Plots: sample_data(phylo3.hell.fallout)$Altitude, plot permutation: free ## Permutation: series
constant permutation within each Plot
## Number of permutations: 1296 ##
## Model: vegan::dbrda(formula = otu_table(phylo3.hell.fallout) ~ Season + Altitude + T_max + Season: ##            Df SumOfSqs
                          F                   Pr(>F)
## T_max                   1     1.6040 3.1278 0.013878 *
## Season:Altitude  2           0.5888 0.5740 0.001542 **
## Residual                17      8.7183
## ---
## Signif. codes:  0 '***' 0.001 '**' 0.01 '*' 0.05 '.' 0.1 ' ' 1

##
##                          statistic          SES      mean lower median   upper Pr(perm) ##
T_max                          3.1278 2.1704 2.8373              2.8350 3.0538 0.013878 *
## Season:Altitude             0.5740 3.5030 0.4352              0.4297 0.5044 0.001542 ** ## --
-
## Signif. codes:  0 '***' 0.001 '**' 0.01 '*' 0.05 '.' 0.1 ' ' 1 ##
## (Interval (Upper - Lower) = 0.95)
```

9.5. Missing data model-based imputation

This section is to infer data from Coyhaique in the fall, for enhanced visualizations.

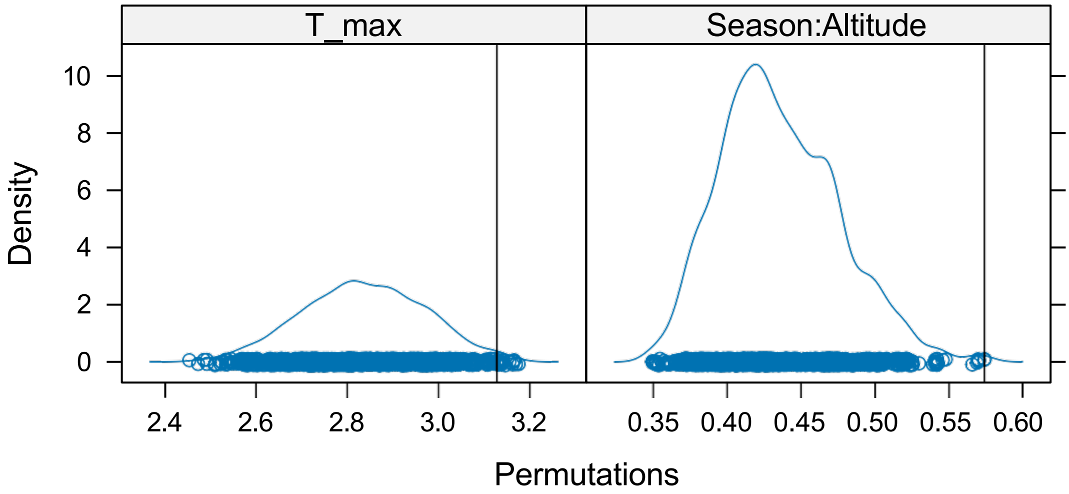

Figure 28: Density distributions of marginal psudo-F values by model term under the null expectations (permuted). Vertical lines indicates the observed values.

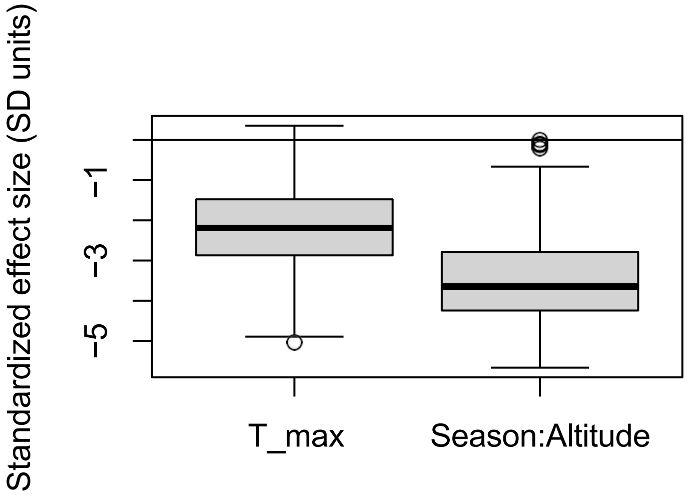

Figure 29: Standardized marginal effect sizes based on psudo-F values of permutated minus the observed values (expressed in SD units).

```
# Add sample data of unobserved samples. This will be used to predict the # unobserved
microbe community (i.e., model-based imputation).
X <- as.matrix(otu_table(phylo3.hell))

sam_data <- get_variable(phylo3.hell, c("Location", "Altitude", "Altitude2", "Season", "spsample", "Latitude",
        "T_min", "T_mean", "T_max", "plot"))

tmp <- data.frame(Location = "Coyhaique", Altitude = "Treeline", Altitude2 = "AT",
        Season = "Fall", spsample = "CT.Fall", Latitude = -45.51626, T_min = -0.2, T_mean = 4.3, T_max = 8.9, plot =
        "Coyhaique.Treeline", row.names = "CT.Fall")

sam_data. <- rbind(sam_data[, colnames(tmp)], tmp)

dummy.X <- model.matrix(~. - 1, data = sam_data.[, c("Location", "Altitude", "Season")])   #, 'Latitud # Add the corresponding row

(filled with NAs) in the community matrix.
tmp <- X[1, ]
tmp[, ] <- NA
row.names(tmp) <- "CT.Fall"
X. <- rbind(X, tmp)

## Model-based imputation ----

# All methodologies implemented in mixOmics can handle missing values.   In # particular,
(s)PLS, (s)PLS-DA, (s)PCA utilize the NIPALS (Non-linear
# Iterative Partial Least Squares) algorithm as part of their dimension # reduction
procedures.   This algorithm is built to handle NAs [1].

# nipals() # function handling data imputation internally If missing values # need to be imputed,
the package contains impute.nipals()

pls1 <- mixOmics::pls(X = dummy.X, Y = X., ncomp = 2, scale = F, mode = "classic") tmp <- predict(pls1,
newdata = pls1$X)
tmp <- as.data.frame(t(tmp$predict[32, , 2]), row.names = "CT.Fall")

# Predictions sometimes produced tiny negative values that were zeroed.
tmp[tmp < 0] <- 0

# Original data + imputed values for Coyhaique-Fall
W <- rbind(X.[-32, ], tmp)

# Consolidate results in a new phyloseq object
phylo3.imputed <- phyloseq(sample_data(sam_data.), otu_table(W, taxa_are_rows = F),
        tax_table(phylo3))
phylo3.imputed <- microViz::phyloseq_validate(phylo3.imputed) phylo3.imputed <-
microViz::tax_fix(phylo3.imputed)

# For the sake of tidiness, reorder samples
phylo3.imputed <- phylo3.imputed %>%
        microViz::ps_arrange(Location, Altitude, Season)
# phylo3.imputed
```

```r
# Convert to psExtra object and add the original count data to the counts slot # (for iris plotting)
sampleid <- data.frame(rowname = rownames(sample_data(phylo3.imputed))) tmp <-
as.data.frame(otu_table(phylo3)) %>%
    rownames_to_column()
tmp <- full_join(sampleid, tmp) %>% select(-
    rowname)
```

```
## Joining with `by = join_by(rowname)`
```

```r
tmp[is.na(tmp)] <- 1


phylo3.imputed <- microViz::tax_transform(phylo3.imputed, trans = "identity", keep_counts = TRUE)
```

```
## Warning: otu_table of counts is NOT available!
## Available otu_table contains 1930 values that are not non-negative integers
```

```r
phylo3.imputed@counts <- otu_table(tmp, taxa_are_rows = F)
phylo3.imputed@info$tax_trans <- "hellinger"   # observed and predicted values were pre-transformed.
rownames(phylo3.imputed@counts) <- sample_names(phylo3.imputed)
```

9.6. Plotting RDA

The interpretation of the tb-RDA plot is straightforward: the first axis (RDA1) is related to latitudinal variation and the second (RDA2) with seasonal and altitudinal variation Figure 30.

Coming-up an enhanced figure and additional visualizations (loading plots, iris plots) to aid the interpre- tation of the RDA.

9.6.1. Plotting alternatives

Figure 30 portrays the results of a tb-RDA fitted with the function capscale{vegan} and then custom- plotted layer by layer. Similar results were obtained by fitting with function ord_calc{microViz} and then plotting with ord_plot{microViz}and additional customization.

Note that ord_calc{microViz} can perform both method = "CAP" and method = "RDA". It turns out that only with method = "RDA" species scores are returned, thus facilitating the plotting of taxonomic vectors. Also, it should be noted that the scales of axes are different yet preserving the same patterns/map. When method = "CAP" is used, it is necessary to calculate the distance matrix beforehand adding one line of code.

9.6.2. tb-RDA plots by taxonomic rank

Follow a series of figures, each portraying a similar analysis applied at each taxonomic rank, from Phylum to Species (Figures 33 to 38).

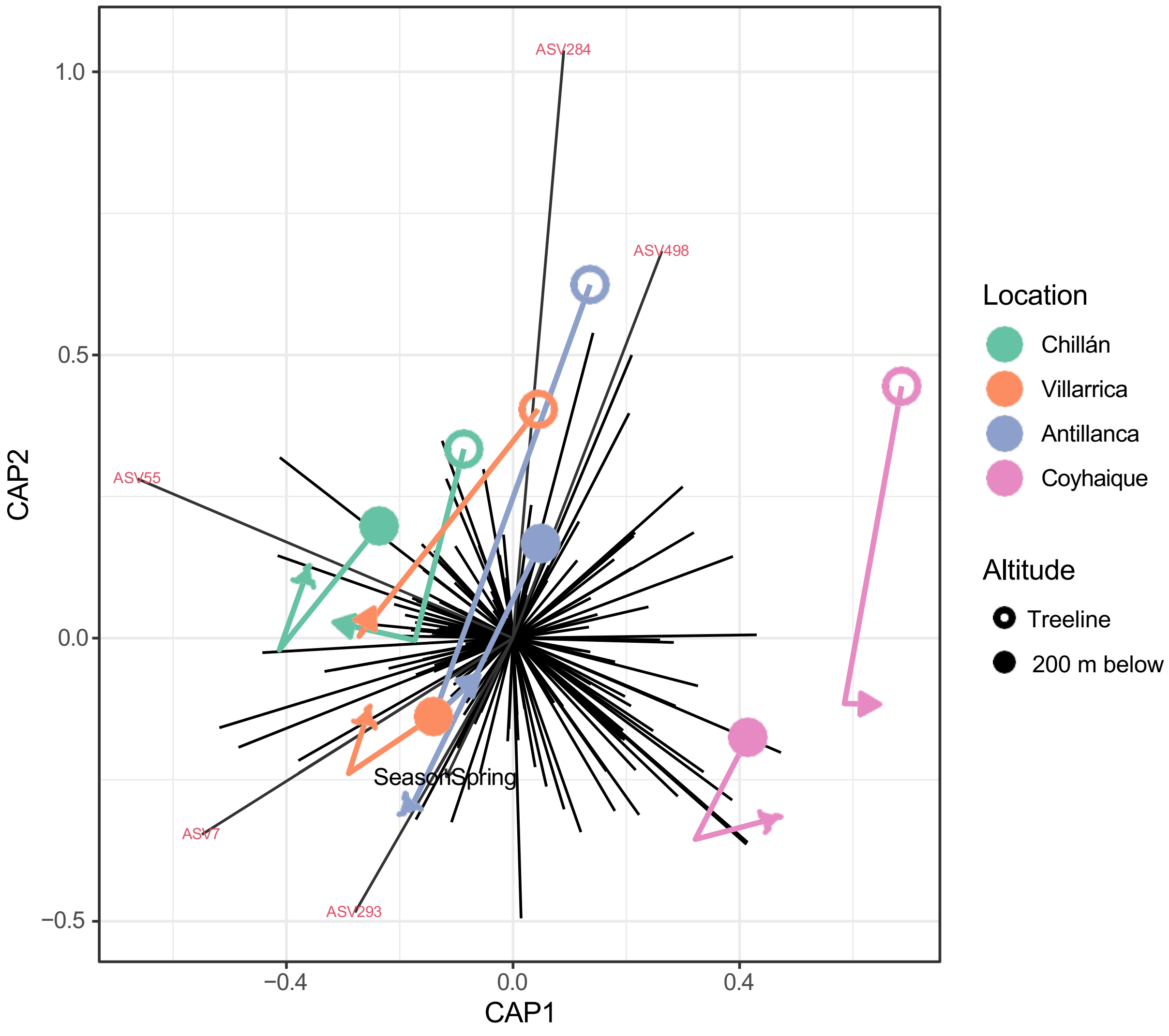

Figure 30: Preliminary visualization of the tbRDA resuls. Samples from the same place were connected with edges beginning from a start circle in winter and ending with arrowheads in summer. Altitude is also distinguished with different edge type and start-circles. Note that all fall samples are missing (for now, but will do model-based imputation).

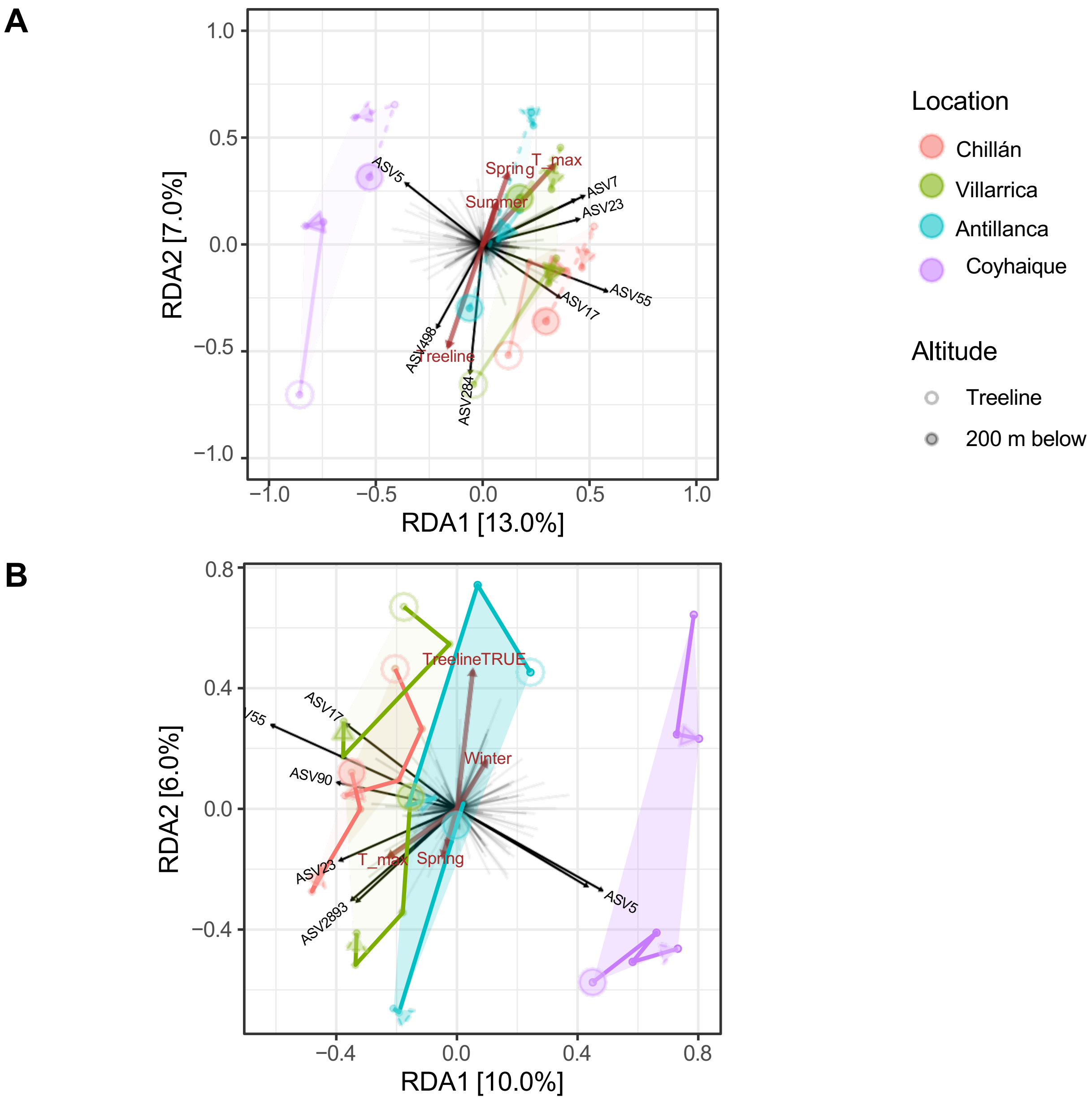

Figure 31: (#fig:tbRDA2,)Transformation-based redundancy analysis (tb-RDA) triplots of the data set excluding (A) or not (B) the fall data. Site scores from samples from the same place were connected with edges beginning in winter (A) or fall (B) (circles) and ending in summer (arrowheads). Altitude is distinguished by edge type (continuous for treeline) and start-circle type (hollow for treeline). Scaling was sample-oriented (i.e., type 2) trying to preserve Euclidean distance in the ordination space.

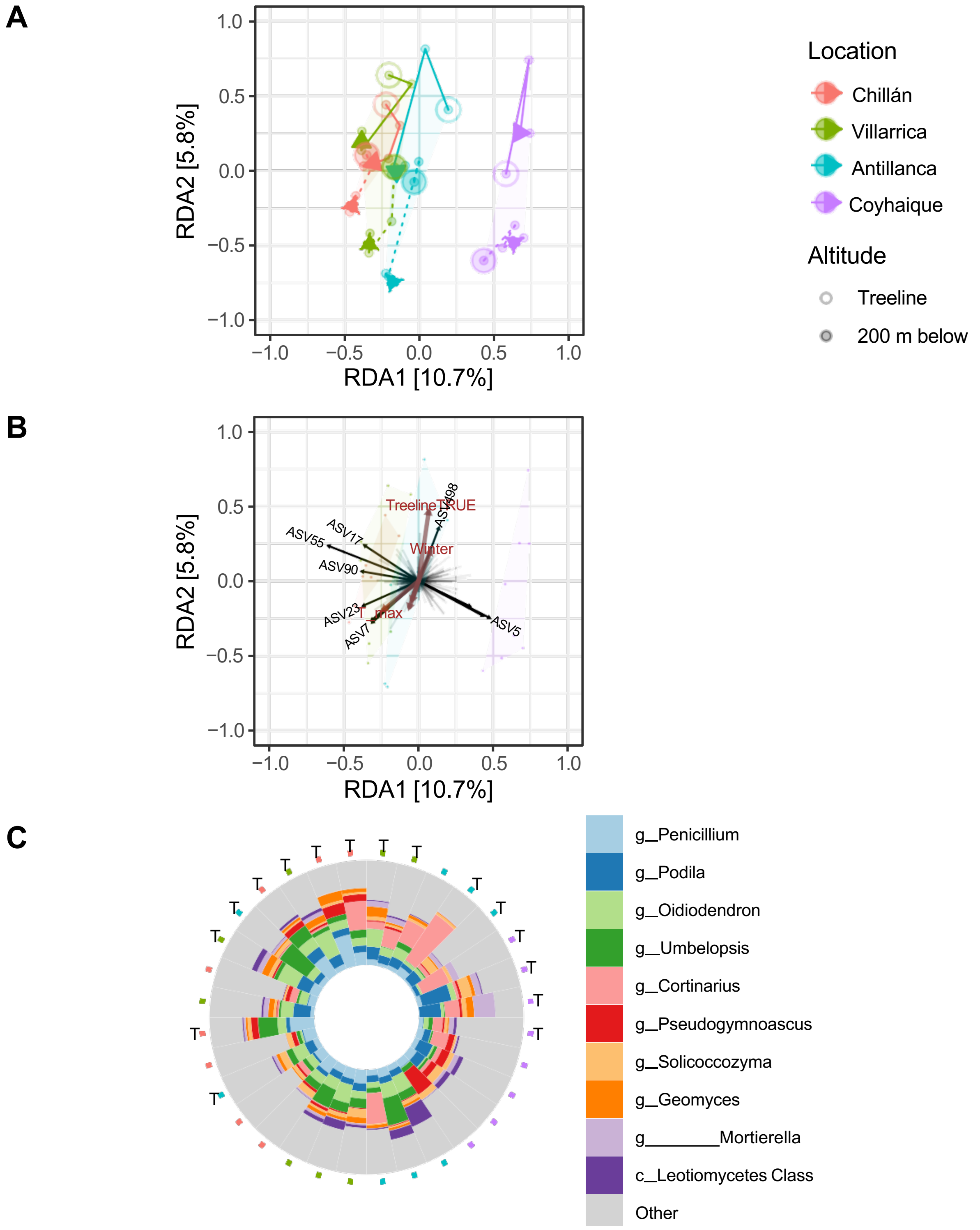

Figure 32: Transformation-based redundancy analysis (tb-RDA) triplot, including model-based imputation of missing data from Coyhaique in the fall. (A) Site scores from samples from the same place were connected with edges beginning in the fall (circles) and ending in the summer (arrowheads). Altitude is distinguished by edge type (continuous for treeline) and start-circle type (hollow for treeline). (B) Species scores as well as constraining variables of the same ordination are shown. (C) Irisplot to aid the interpretation of taxonomic composition of the ordinated samples. Only the top ten taxa were labeled. Triplot scaling was sample-oriented (i.e., type 2) trying to preserve Euclidean distance in the ordination space.

9.6.3. tb-RDA plots by taxonomic rank and including latitude

Follow a series of figures, each portraying a similar analysis applied at each taxonomic rank, from Phylum to Species, but this time Latitude was arbitrarily added to the models (Figures 39 to 44).

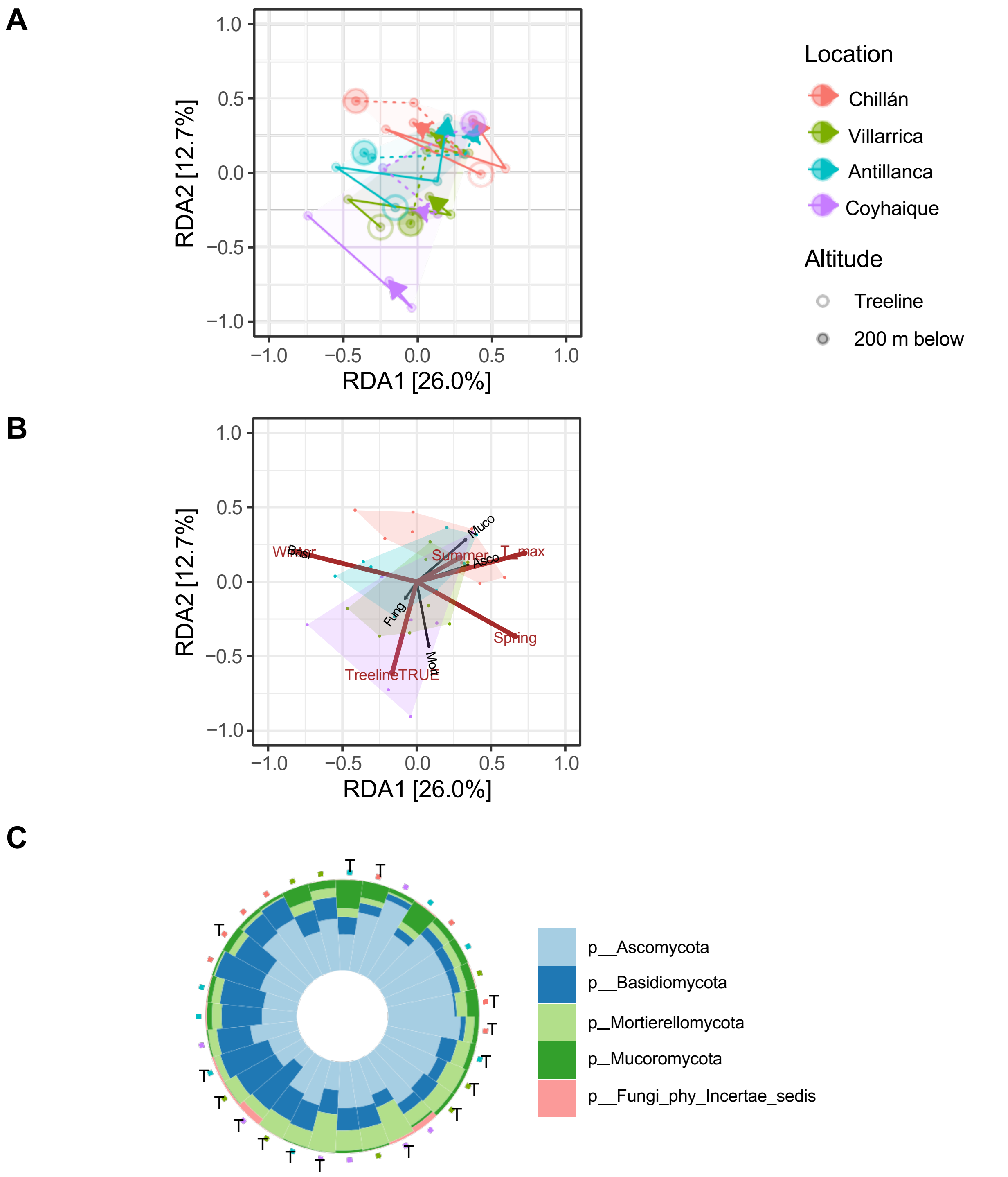

Figure 33: Transformation-based redundancy analysis (tb-RDA) triplot of the Phylum-level aggregated data set. (A) Site scores from time series were connected forming temporal trajectories beginning in the fall (circles) and ending in the summer (arrowheads). Trajectories from the treeline were drawn with continuous lines and hollow circles, whereas those from 200 m below the treeline with broken lines and filled circles. (B) Species scores as well as constraining variables of the same ordination were plotted separately for clarity. (C) An irisplot displaying the relative abundance of each taxon further aids the interpretation of the community composition of the ordinated samples. Only the top ten taxa were labeled. Triplot scaling was sample-oriented (i.e., type 2) trying to preserve Euclidean distance in the ordination space.

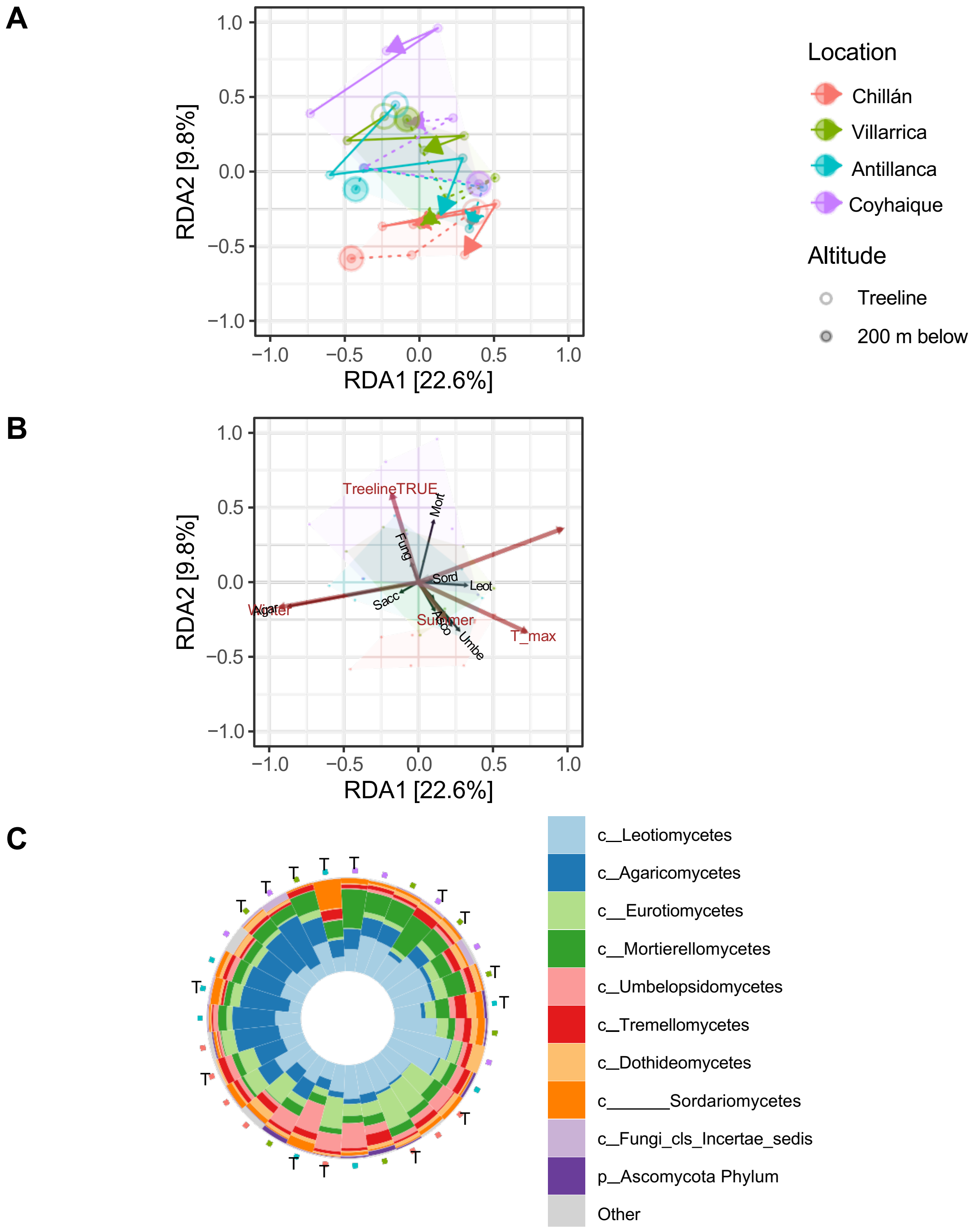

Figure 34: Transformation-based redundancy analysis (tb-RDA) triplot of the Class-level aggregated data set. Additional conventions as in previous figure.

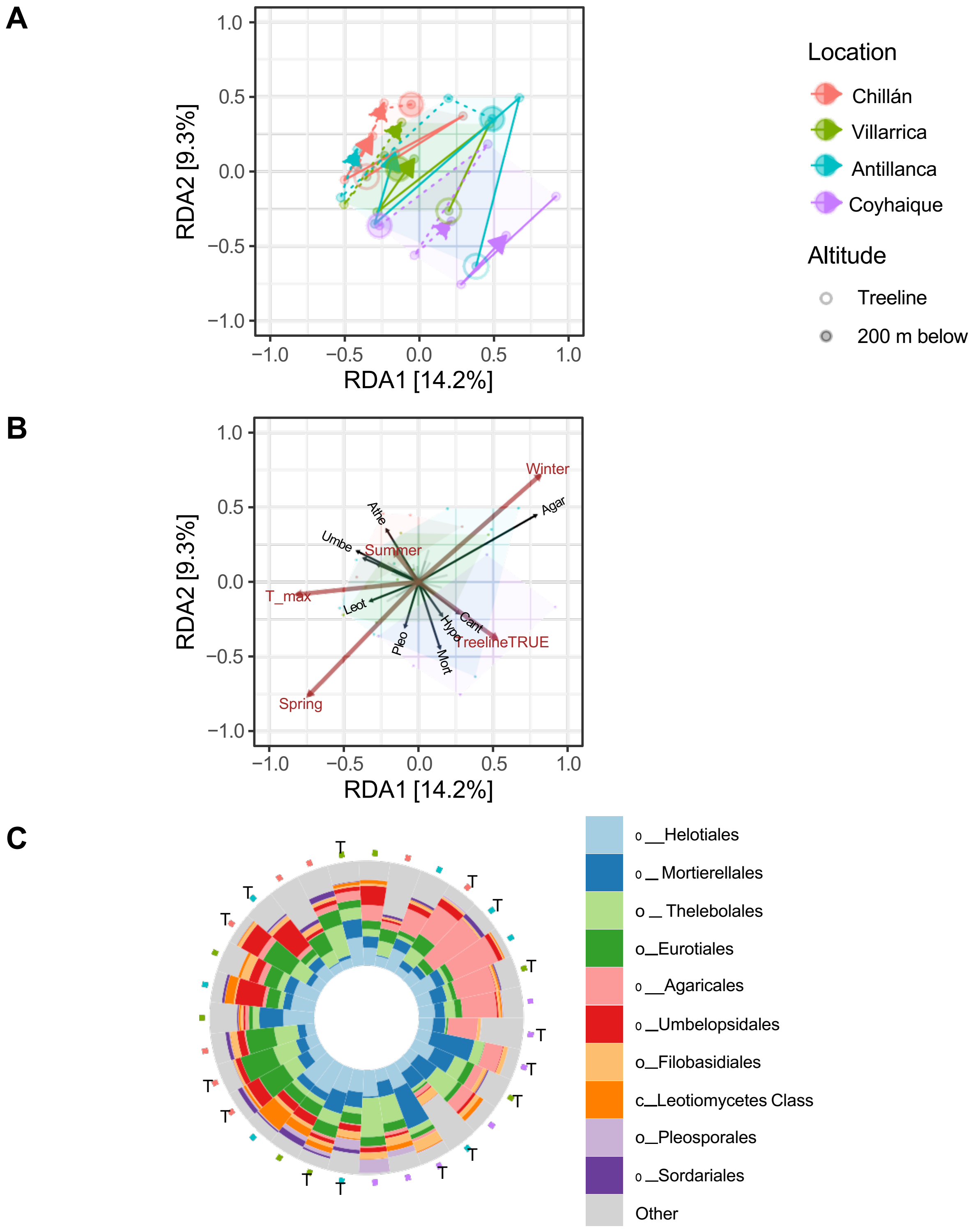

Figure 35: Transformation-based redundancy analysis (tb-RDA) triplot of the Order-level aggregated data set. Additional conventions as in previous figure.

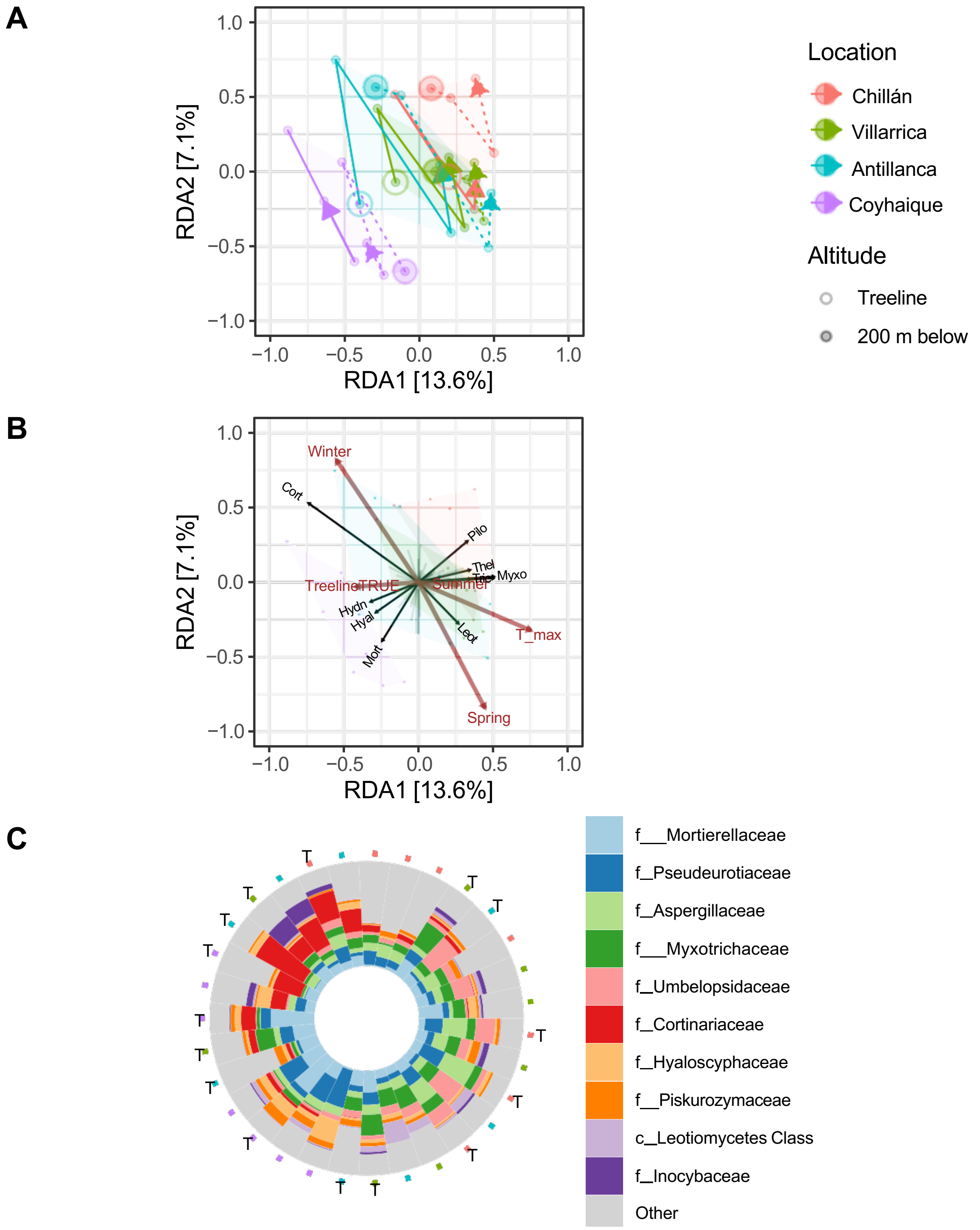

Figure 36: Transformation-based redundancy analysis (tb-RDA) triplot of the Family-level aggregated data set. Additional conventions as in previous figure.

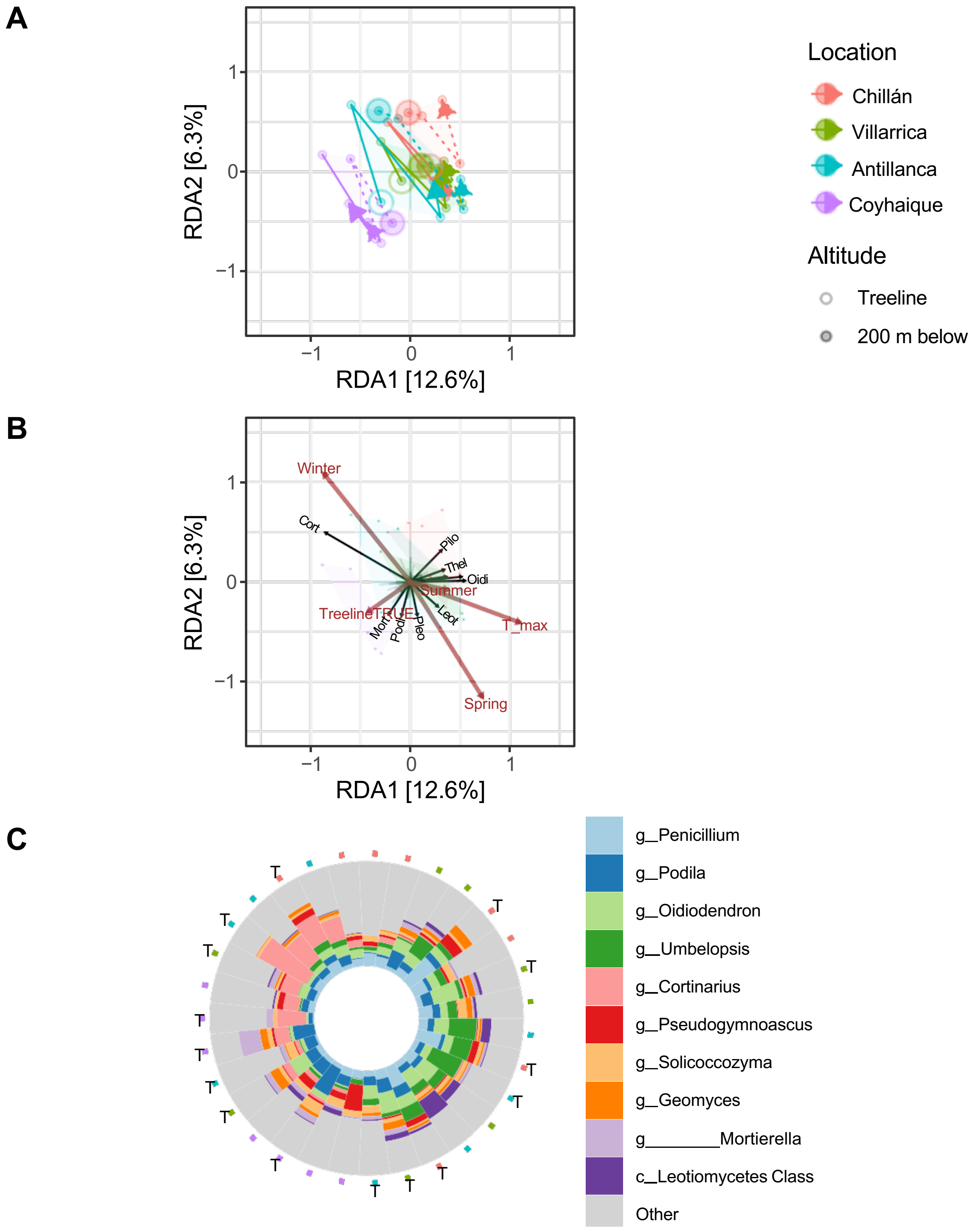

Figure 37: Transformation-based redundancy analysis (tb-RDA) triplot of the Genus-level aggregated data set. Additional conventions as in previous figure.

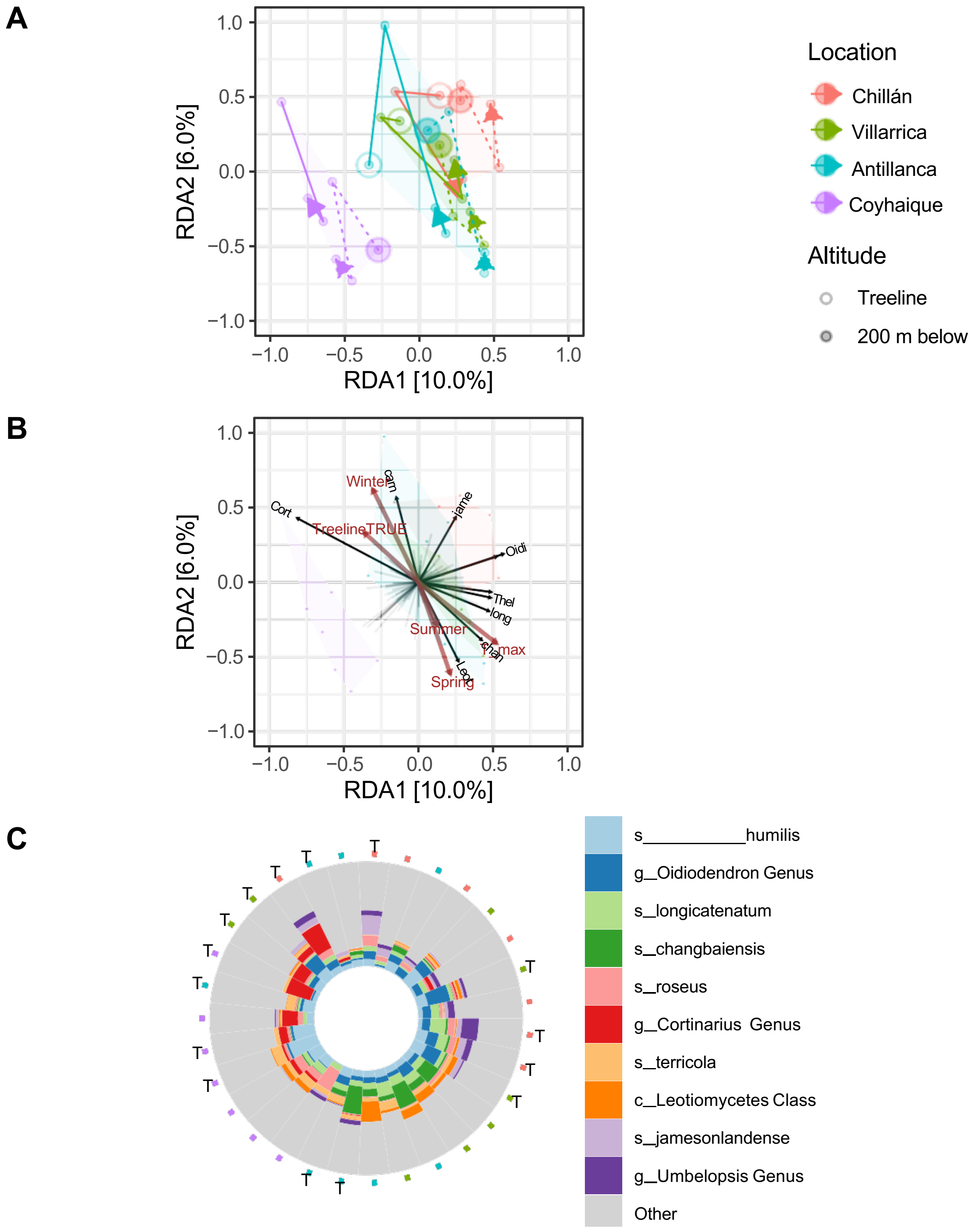

Figure 38: Transformation-based redundancy analysis (tb-RDA) triplot of the Species-level aggregated data set. Additional conventions as in previous figure.

10. Guilds constrained ordination

In this section we used a similar analytic strategy as in the previous section applied to the phylo3_guilded data set described earlier. Much of the code (and R object's names) were recycled. Please see the *Constrained ordination: transformation-based redundancy analysis (tb-RDA)* section above for explanations of the analytic strategy. Here only essential results are presented.

10.1. Main gradint length estimation by Detrended Correspondence Analysis (DCA)

```
# Detrended Correspondence analysis of the Guilds data set library(vegan)

# Group ASVs by guild
phydata <- phylo3_guilded %>%
    microViz::tax_agg(rank = "Guild", force = T)

# Hellinger transform
phylo3.hell <- transform_sample_counts(phydata, function(x) sqrt(x/sum(x)))

phylo3.hell
```

```
## phyloseq-class experiment-level object
## otu_table()       OTU Table:          [ 7 taxa and 31 samples ]
## sample_data() Sample Data:            [ 31 samples by 15 sample variables ] ##
tax_table()         Taxonomy Table:     [ 7 taxa by 8 taxonomic ranks ]
```

```
# Check the length of the main gradient.
vegan::decorana(otu_table(phylo3.hell))
```

```
##
## Call:
## vegan::decorana(veg = otu_table(phylo3.hell)) ##
## Detrended correspondence analysis with 26 segments. ## Rescaling
of axes with 4 iterations.
## Total inertia (scaled Chi-square): 0.2669 ##
##                          DCA1     DCA2     DCA3      DCA4
## Eigenvalues            0.1019  0.04704  0.03343  0.041658
## Additive Eigenvalues   0.1019  0.04747  0.03207  0.030275
## Decorana values        0.1097  0.03852  0.01825  0.008108
## Axis lengths           1.1143  0.96372  0.50536  0.713908
```

The length of our *first* DCA axis was 1.11 SD, and we therefore proceeded with linear constrained ordination.

```
# Filtered out fall samples to eliminate imbalance. # (names of R
objects were recycled)

phylo3.hell.fallout <- subset_samples(phylo3.hell, !(Season %in% "Fall"))

# Created permutation object
```

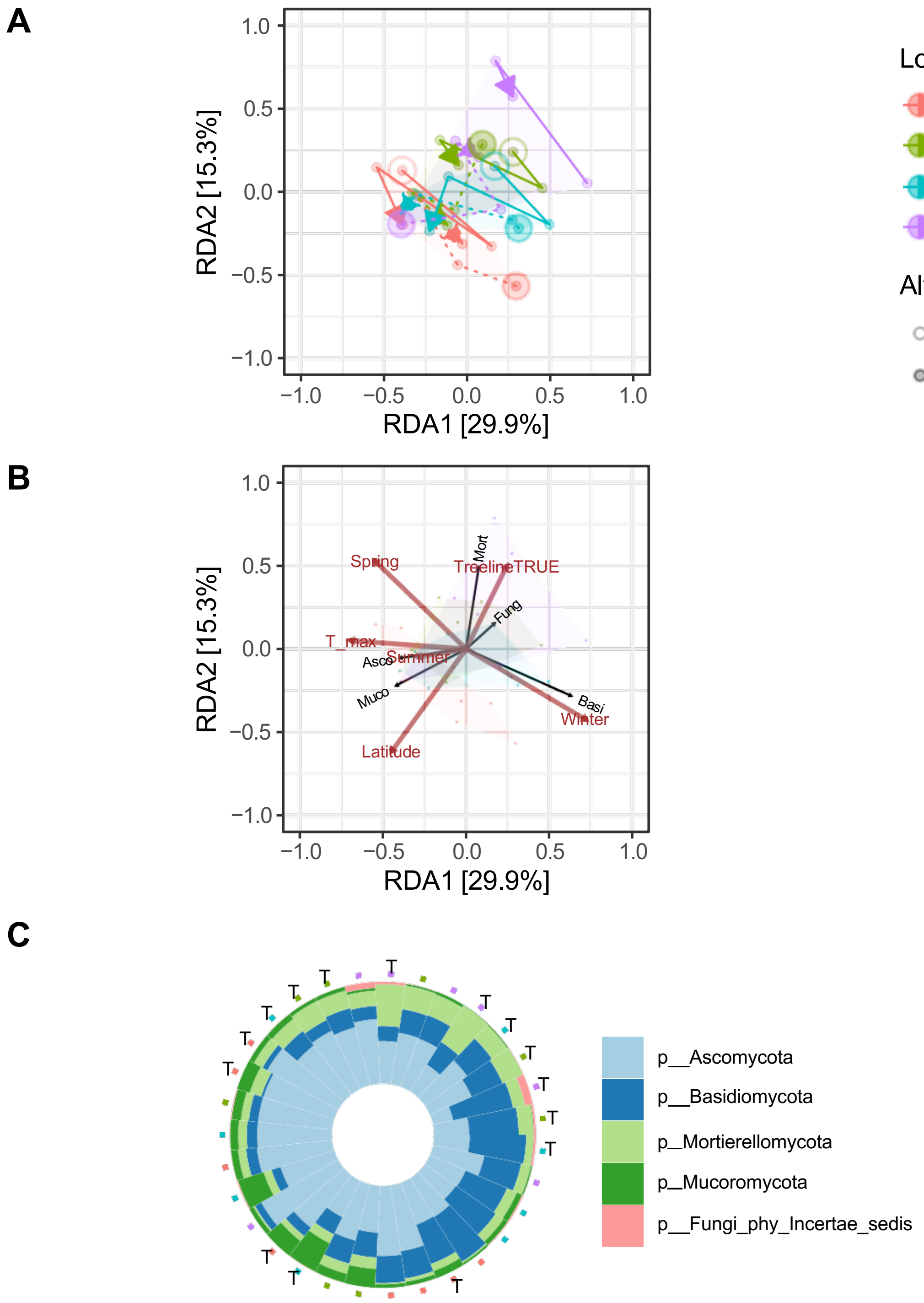

Figure 39: Like Figure 33 but including Latitude in the model.

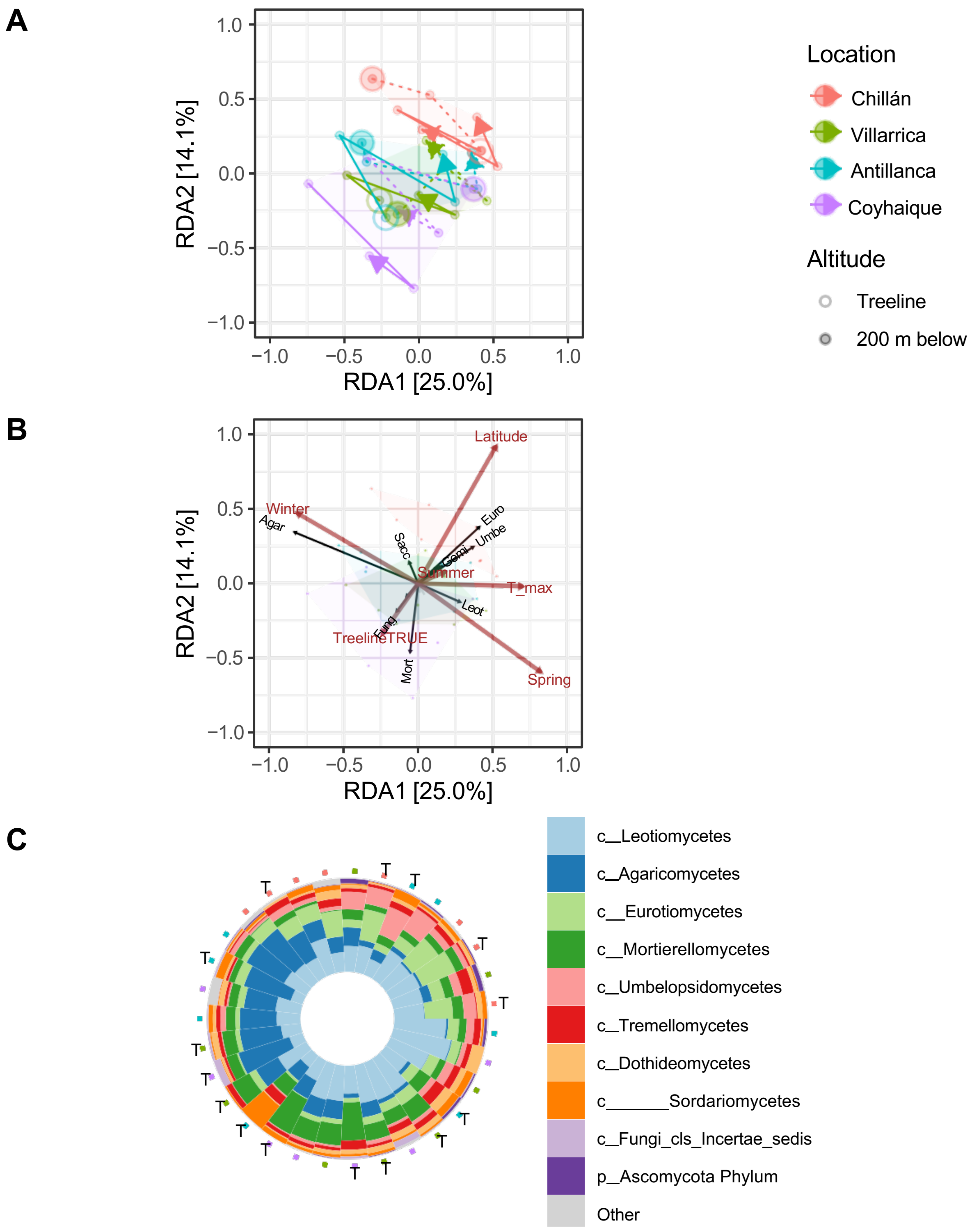

Figure 40: Like Figure 34 but including Latitude in the model.

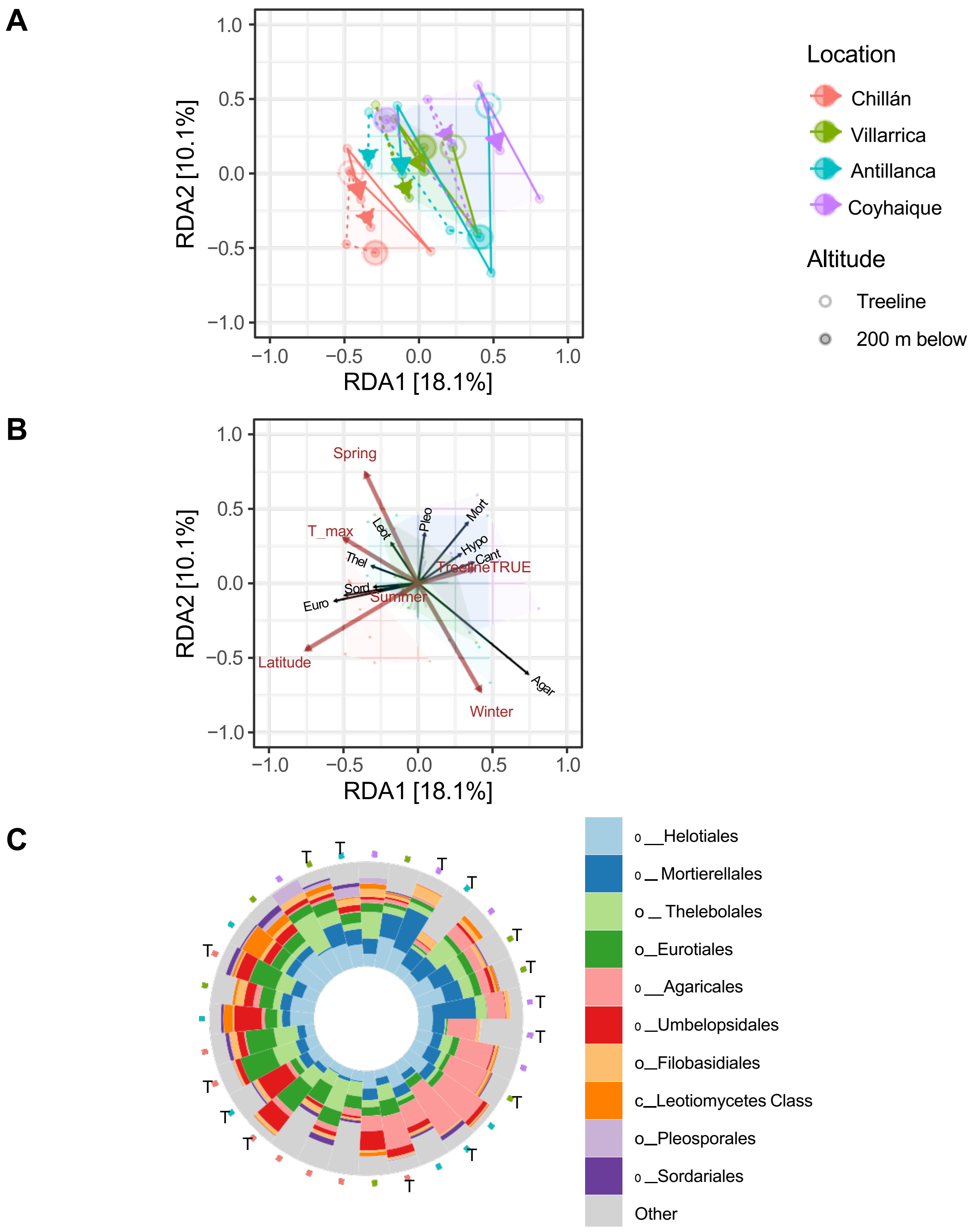

Figure 41: Like Figure 35 but including Latitude in the model.

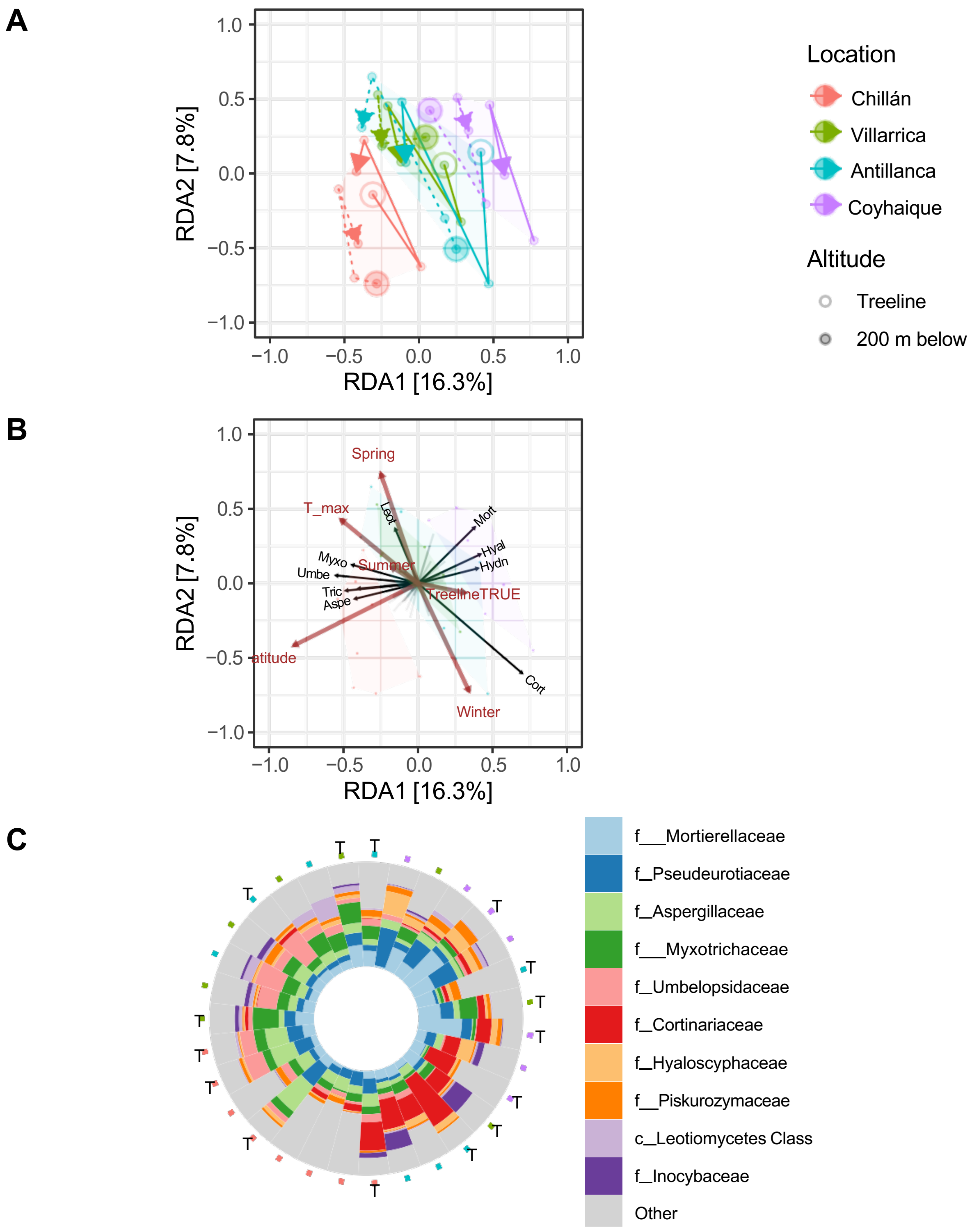

Figure 42: Like Figure 36 but including Latitude in the model.

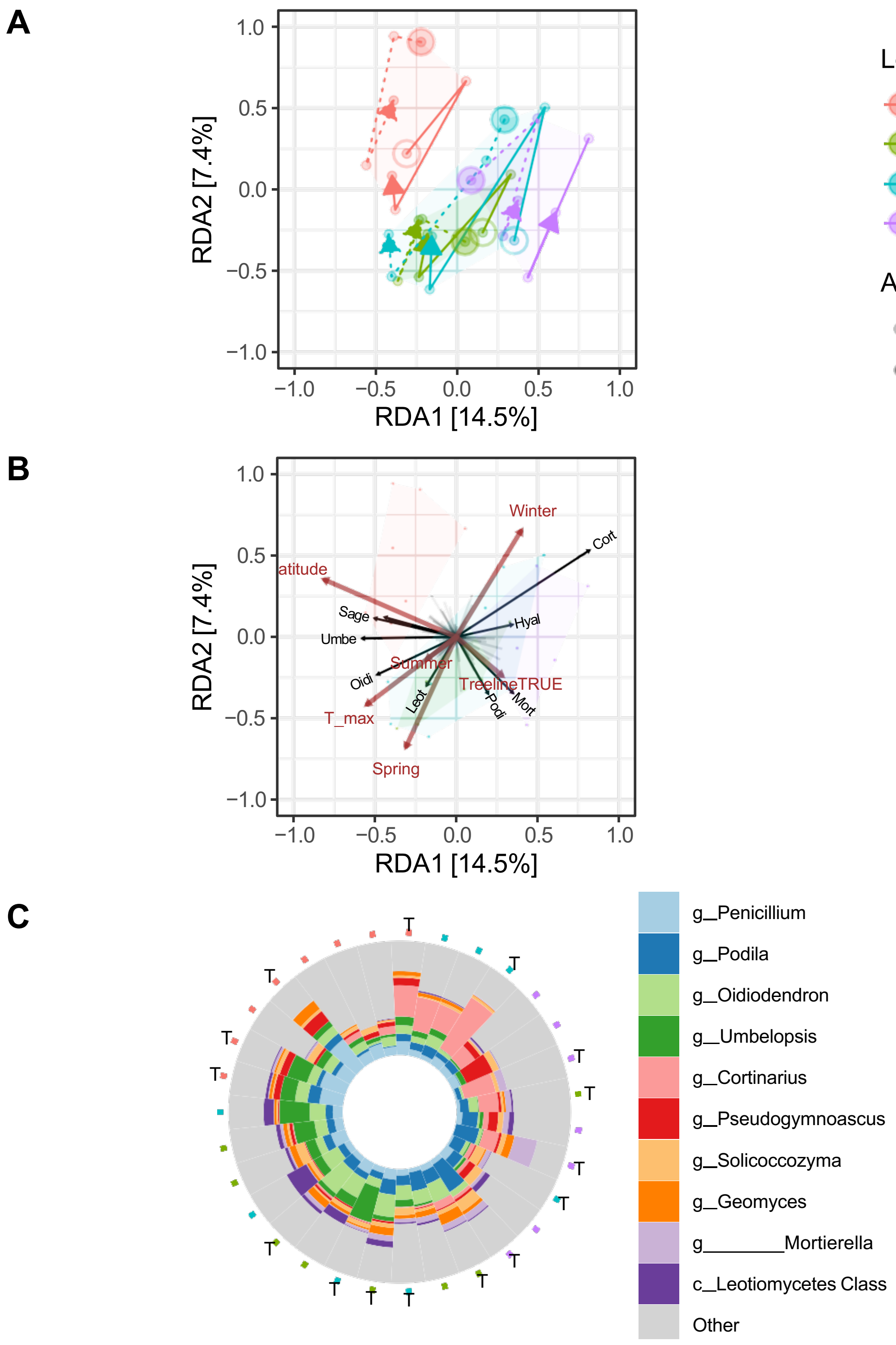

Figure 43: Like Figure 37 but including Latitude in the model.

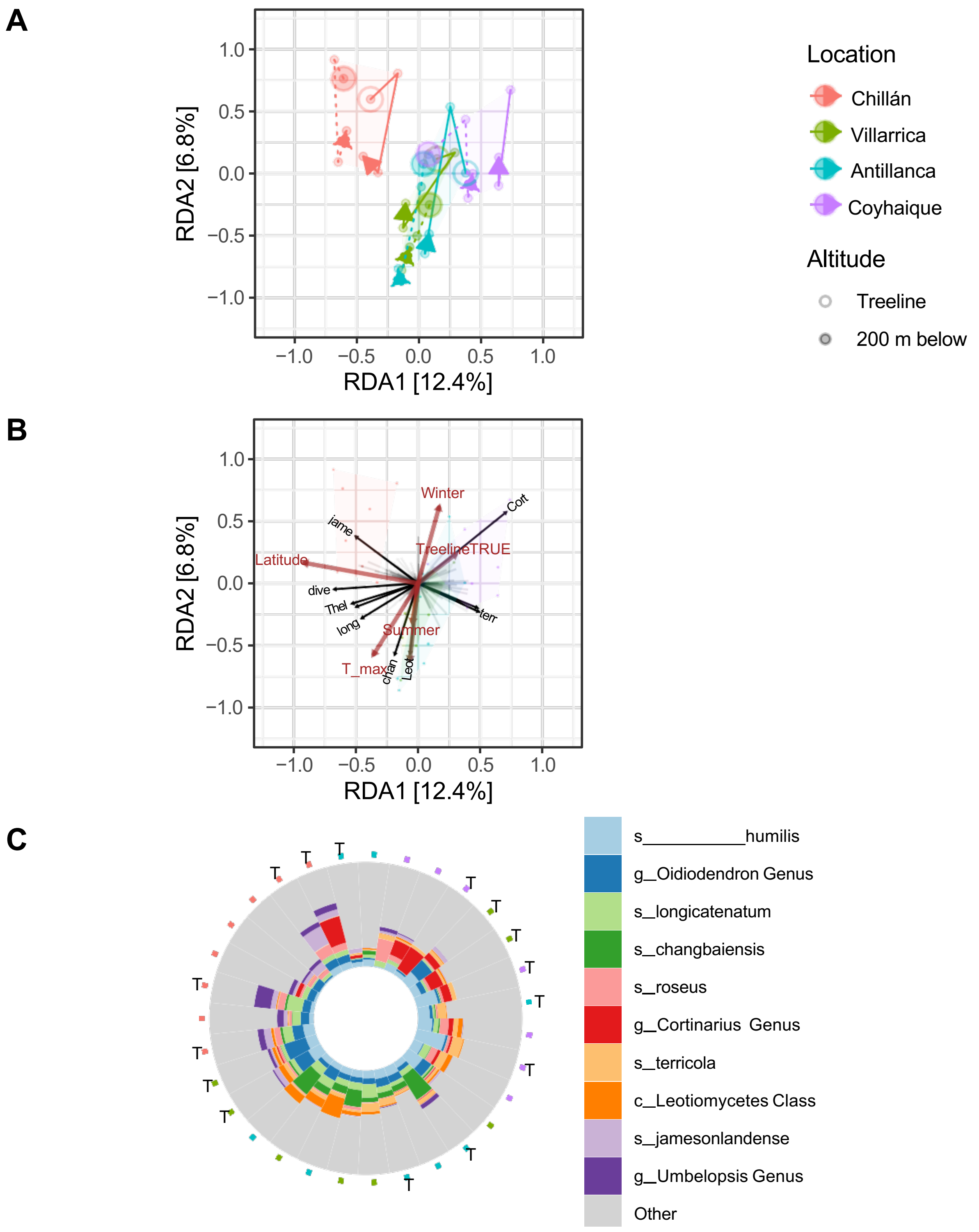

Figure 44: Like Figure 38 but including Latitude in the model.

```
set.perm <-
  how(within = Within(type = "series", constant = T, mirror = F), # for time series
      plots = Plots(strata = sample_data(phylo3.hell.fallout)$Altitude, type = "free"),
      blocks = sample_data(phylo3.hell.fallout)$Location,   # for no permutations among blocks
      observed = T,  # include the observed values
      complete = T) # returns all permutations
```

10.2. Full db-RDA model

The following distance-based redundancy analysis (tb-RDA) was fitted to the data. Initially, all relevant variables were included in the model (Latitude, Season, Altitude, T_min, T_mean, T_max), except for Location which was nonetheless included in the permutation scheme (see above, *Data structure and randomization schema*), and all soil variables which were only available for the summer season (analyzed separately, below).

```
# db-RDA of the Guilds data set. (names of R objects were recycled)

full <- vegan::dbrda(otu_table(phylo3.hell.fallout) ~
                       Latitude + Season +
                       Altitude +
                       Season:Altitude+
                       T_min+
                       T_mean
                       + T_max,
                       # Organic_matter + Nitrogen + Phosphorus + Sulfur, # Soil variables are for the Summer on
                     data = data.frame(sample_data(phylo3.hell.fallout)), add = "lingoes",
                     # to correct for negative eigenvalues
                     comm = otu_table(phylo3.hell.fallout)) # to produce spp scores

full

## Call: vegan::dbrda(formula = otu_table(phylo3.hell.fallout) ~ Latitude ## + Season + Altitude
+ Season:Altitude + T_min + T_mean + T_max, data = ##
data.frame(sample_data(phylo3.hell.fallout)), add = "lingoes", comm = ##
otu_table(phylo3.hell.fallout))
##
##                  Inertia Proportion Rank RealDims
## Total             2.1770     1.0000
## Constrained       1.4110     0.6481    7        7
## Unconstrained     0.7660     0.3519    7        7
## Inertia is squared Euclidean distance ##
## Eigenvalues for constrained axes:
## dbRDA1 dbRDA2 dbRDA3 dbRDA4 dbRDA5 dbRDA6 dbRDA7 ##
1.0423 0.1952 0.0948 0.0447 0.0259 0.0077 0.0005 ##
## Eigenvalues for unconstrained axes:
##    MDS1    MDS2    MDS3    MDS4    MDS5    MDS6    MDS7 ##
0.3886 0.2174 0.0665 0.0489 0.0276 0.0139 0.0029 ##
## Constant added to distances: 2.262361e-16
```

```
# summary(full) # Additional information and scores full$tot.chi # total
# variance, constrained and unconstrained full$CCA$tot.chi # variance explained # by constraints
full$CA$tot.chi # remaining unconstrained variance
```

Firstly, the results indicate all constraining variables together may explain up to 64.8 % of the variation in the fungi community data, and the first two constraining axes explain 56.8 %. The remaining 35.2 % of the variation cannot be explained by the explanatory matrix (unconstrained).

Correlated variables exacerbated the variance of model coefficients.

```
vif.cca(full)
```

```
##                               Latitude                    SeasonSpring
##                               4.442008                       22.126720
##                           SeasonSummer          AltitudeBelow treeline
##                              73.672412                        3.568999
##                                  T_min                          T_mean
##                            6141.049584                    53870.935303
##                                  T_max  SeasonSpring:AltitudeBelow  treeline
##                           26921.565610                        4.317169
##   SeasonSummer:AltitudeBelow  treeline
##                               3.885325
```

10.2.1. Tests of significance

The tests of significance were done through Permutational Multivariate Analyses of Variance (PER- MANOVAs), imposing the restrictions to the permutation schema as described earlier (set.permobject).

A few complementary tests revealed different statistical properties of the fitted model. For example, a test of the significance of the constrained axes suggested that the first was statistically significant ($P < 0.05$).

```
# Test the significance of the axes
perm <- anova.cca(full, by = "axis", permutations = set.perm)
```

```
## Set of permutations < 'minperm'. Generating entire set.
```

```
# Estimated the standardized effect sizes (SES) and confidence intervals
pstat <- permustats(perm)
summary(pstat, interval = 0.95)
```

```
##
##          statistic         SES     mean lower  median      upper Pr(perm) ##
dbRDA1        21.7703  2.6564  9.0929 7.7990 19.2534   0.01542 *
## dbRDA2      4.0776 -1.3446  6.0724                  6.0144  8.6303  0.91673
## dbRDA3      1.9795 -1.5923  4.0511                  3.8654  6.5535  0.97533
## dbRDA4      0.9331 -1.8464  3.6574                  3.5025  6.4072  0.99306
## dbRDA5      0.5411 -1.9473  3.3459                  3.1723  6.0206  0.99769
## dbRDA6      0.1601 -2.0261  3.0476                  2.8191  5.6188  0.99846
## dbRDA7      0.0105 -2.1244  1.1283                  1.0499  2.1024  0.99923 ## --
-
## Signif. codes:  0 '***' 0.001 '**' 0.01 '*' 0.05 '.' 0.1 ' ' 1 ##
## (Interval (Upper - Lower) = 0.95)
```

The marginal effect of each model term while controlling for the effects of all others:

```
# Significance tests for each marginal term in a model with all other terms # included (order does not matter).
perm <- anova.cca(full, by = "margin", scope = formula(full), permutations = set.perm)
```

Set of permutations < 'minperm'. Generating entire set.

```
perm
```

Permutation test for dbrda under reduced model
Marginal effects of terms
Blocks: sample_data(phylo3.hell.fallout)$Location
Plots: sample_data(phylo3.hell.fallout)$Altitude, plot permutation: free ## Permutation: series constant permutation within each Plot
Number of permutations: 1296
Model: vegan::dbrda(formula = otu_table(phylo3.hell.fallout) ~ Latitude + Season + Altitude + Seas ## Df SumOfSqs F Pr(>F)

##	Latitude	1	0.10022	1.8316	0.2552
##	T_min	1	0.05754	1.0516	0.4827
##	T_mean	1	0.05283	0.9656	0.5081
##	T_max	1	0.05171	0.9452	0.5197
##	Season:Altitude	2	0.11372	1.0392	0.1527
##	Residual	14	0.76600		

None of the marginal effects were significant at explaining the fungi guild composition, but recall that coefficient's variances are substantially influenced by covatiates.

10.3. Model selection

Model selection was carried out to simplify the full model into a more parsimonious, less redundant model. The function vegan::ordistep() was used, with bidirectional (default) stepwise search of the best set of explanatory variables, and the permutation scheme described above.

```
best <- vegan::ordistep(full, direction = "both", permutations = set.perm, trace = F) best
```

Call: vegan::dbrda(formula = otu_table(phylo3.hell.fallout) ~ Latitude ## + Season + Altitude + Season:Altitude, data =
data.frame(sample_data(phylo3.hell.fallout)), add = "lingoes", comm = ## otu_table(phylo3.hell.fallout))
##

##		Inertia	Proportion	Rank
##	Total	2.1770	1.0000	
##	Constrained	1.1561	0.5311	6
##	Unconstrained	1.0209	0.4689	7

Inertia is squared Euclidean distance
Eigenvalues for constrained axes:
dbRDA1 dbRDA2 dbRDA3 dbRDA4 dbRDA5 dbRDA6
0.9610 0.1296 0.0467 0.0130 0.0056 0.0001

Eigenvalues for unconstrained axes:
MDS1 MDS2 MDS3 MDS4 MDS5 MDS6 MDS7
0.4830 0.3166 0.0734 0.0660 0.0515 0.0234 0.0070 ##
Constant added to distances: 2.262361e-16

Following bidirectional stepwise model selection, we concluded that the best model explaining the fungi guild composition included Latitude, Season, Altitude, and the interaction Season:Altitude. Again, even though Location was not included in the model, it was considered in the permutation scheme as a key component of the experimental design.

The best tb-RDA model explained 53.1 % of the variance (R^2), though penalizing for the number of predictors the proportion drops to 36.6 % (i.e., Adj. R^2).

All constraining variables together may explain up to 53.1 % of the variation in the fungi community data, and the first two constraining axes explain 50.1 %. The remaining 46.9 % of the variation cannot be explained by the explanatory matrix (unconstrained). Therefore, about half of the observed variation was the result of other environmental or stochastic processes, and this needs to be taken into account in all subsequent interpretations and visualizations presented below.

Variance inflation factors (VIF) were in the range 1, 3.33 in the final model indicating no concern regarding collinearity among explanatory variables.

```
vif.cca(best)
```

```
##                                Latitude                          SeasonSpring
##                                1.000001                              2.666667
##                            SeasonSummer                 AltitudeBelow treeline
##                                2.666667                              3.000001
## SeasonSpring:AltitudeBelow treeline SeasonSummer:AltitudeBelow treeline ##     3.333333
                                3.333333
```

10.3.1. Tests of significance

The tests of significance were done through Permutational Multivariate Analyses of Variance (PER- MANOVAs), imposing the restrictions to the permutation schema as described earlier.

A few complementary tests revealed different statistical properties of the fitted model. For example, a test of the significance of the constrained axes:

```
# Test the significance of the axes
perm <- anova.cca(best, by = "axis", permutations = set.perm)
```

```
## Set of permutations < 'minperm'. Generating entire set.
```

```
# Estimated the standardized effect sizes (SES) and confidence intervals
pstat <- permustats(perm)
summary(pstat, interval = 0.95)
```

```
##
##         statistic     SES    mean lower  median    upper Pr(perm)
## dbRDA1    16.0037  3.7654  4.4970         3.8150 12.1836  0.00771 **
## dbRDA2     2.1580 -1.0478  2.7619         2.6762  3.8035  0.88126
## dbRDA3     0.7781 -1.4239  1.9508         1.8805  3.4346  0.93138
## dbRDA4     0.2167 -1.7395  1.6654         1.5468  3.2075  0.98766
## dbRDA5     0.0932 -1.7826  1.1577         1.0365  2.3435  0.99383
```

dbRDA6 0.0021 -1.2367 0.4374 0.3268 1.1977 0.99614 ## --
-
Signif. codes: 0 '***' 0.001 '**' 0.01 '*' 0.05 '.' 0.1 ' ' 1
(Interval (Upper - Lower) = 0.95)

The first constraining axis was statistically significant (P < 0.05).

PERMANOVA of the marginal effect of each model term while controlling for the effects of all others indicated that all model terms of the best model were statistically significant (P < 0.05; Figures 45 and 46).

```
# Significance tests for each marginal term in a model with all other terms # included (order does
not matter).
perm <- anova.cca(best, by = "margin", scope = formula(best), permutations = set.perm) perm
```

Permutation test for dbrda under reduced model
Marginal effects of terms
Blocks: sample_data(phylo3.hell.fallout)$Location
Plots: sample_data(phylo3.hell.fallout)$Altitude, plot permutation: free ## Permutation: series
constant permutation within each Plot
Number of permutations: 1296
Model: vegan::dbrda(formula = otu_table(phylo3.hell.fallout) ~ Latitude + Season + Altitude + Seas ## Df SumOfSqs F
Pr(>F)
Latitude 1 0.14875 2.4771 0.007710 **
Season:Altitude 2 0.17123 1.4256 0.003084 **
Residual 17 1.02088

Signif. codes: 0 '***' 0.001 '**' 0.01 '*' 0.05 '.' 0.1 ' ' 1

All constraining variables of the stepwise-selected best model were significant (P < 0.05; 45 and 46).

##
statistic SES mean lower median upper Pr(perm)
Latitude 2.4771 3.6576 1.6115 1.5625 2.2263 0.007710 **
Season:Altitude 1.4256 4.0087 0.4589 0.4179 0.8838 0.003084 ** ## --
-
Signif. codes: 0 '***' 0.001 '**' 0.01 '*' 0.05 '.' 0.1 ' ' 1
(Interval (Upper - Lower) = 0.95)

10.4. Guilds tb-RDA triplot

Figure 47 shows the results of the stepwise selected tb-RDA based on a the subset of data with guilds information (i.e., phylo3_guilded dataset).

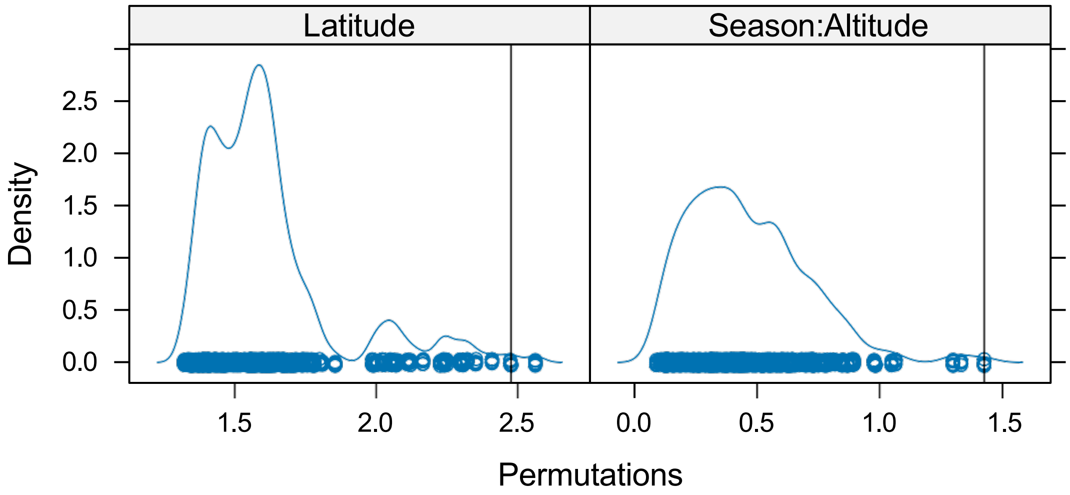

Figure 45: Density distributions of marginal psudo-F values by model term under the null expectations (permuted). Vertical lines indicates the observed values.

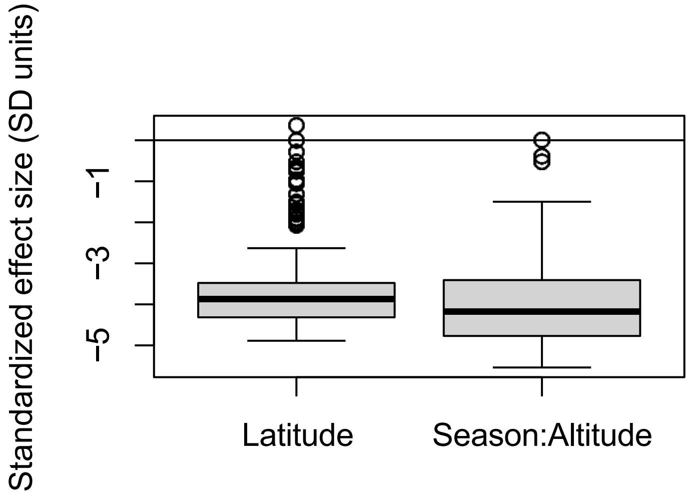

Figure 46: Standardized marginal effect sizes based on psudo-F values of permutated minus the observed values (expressed in SD units).

11. Soil summer variables and fungi taxonomic and functional beta diversity

In this section we used a similar analytic strategy applied to a subset of the phylo3 data set retaining only summer samples which had soil nutrient content variables in addition to all other variables previously analyzed.

Much of the code (and R object's names) were recycled. Please see the *Constrained ordination: transformation-based redundancy analysis (tb-RDA)* section above for explanations of the analytic strategy. Here only essential results are presented.

```
# Hellinger transform
phylo3.hell <- transform_sample_counts(phylo3, function(x) sqrt(x/sum(x)))

# Filtered summer samples
phylo3.hell.summer <- subset_samples(phylo3.hell, (Season %in% "Summer"))
```

11.1. Main gradint length estimation by Detrended Correspondence Analysis (DCA)

```
# Detrended Correspondence analysis of the Summer data set library(vegan)

# Check the length of the main gradient.
vegan::decorana(otu_table(phylo3.hell.summer))
```

```
##
## Call:
## vegan::decorana(veg = otu_table(phylo3.hell.summer)) ##
## Detrended correspondence analysis with 26 segments. ## Rescaling
of axes with 4 iterations.
## Total inertia (scaled Chi-square): NaN ##
##                          DCA1   DCA2   DCA3    DCA4
## Eigenvalues             0.4030 0.3276 0.1606 0.18866
## Additive Eigenvalues    0.0000 0.0000 0.0000 0.00000
## Decorana values         0.4319 0.2024 0.1258 0.01062
## Axis lengths            2.5679 2.0051 1.5427 1.56666
```

The length of our *first* DCA axis was 2.57 SD, and we therefore proceeded with linear constrained ordination.

11.2. Full db-RDA model

The following distance-based redundancy analysis (tb-RDA) was fitted to the data. Initially, all nutrient content variables were included in the model (log10(P), log10(N), log10(S), and log10(OM)).

```
full <- vegan::dbrda(otu_table(phylo3.hell.summer) ~
            log10(Organic_matter)+ log10(Nitrogen)+ log10(Phosphorus)+ log10(Sulfur), data =
            data.frame(sample_data(phylo3.hell.summer)),
            add = "lingoes", # to correct for negative eigenvalues
            comm = otu_table(phylo3.hell.summer)) # to produce spp scores

full
```

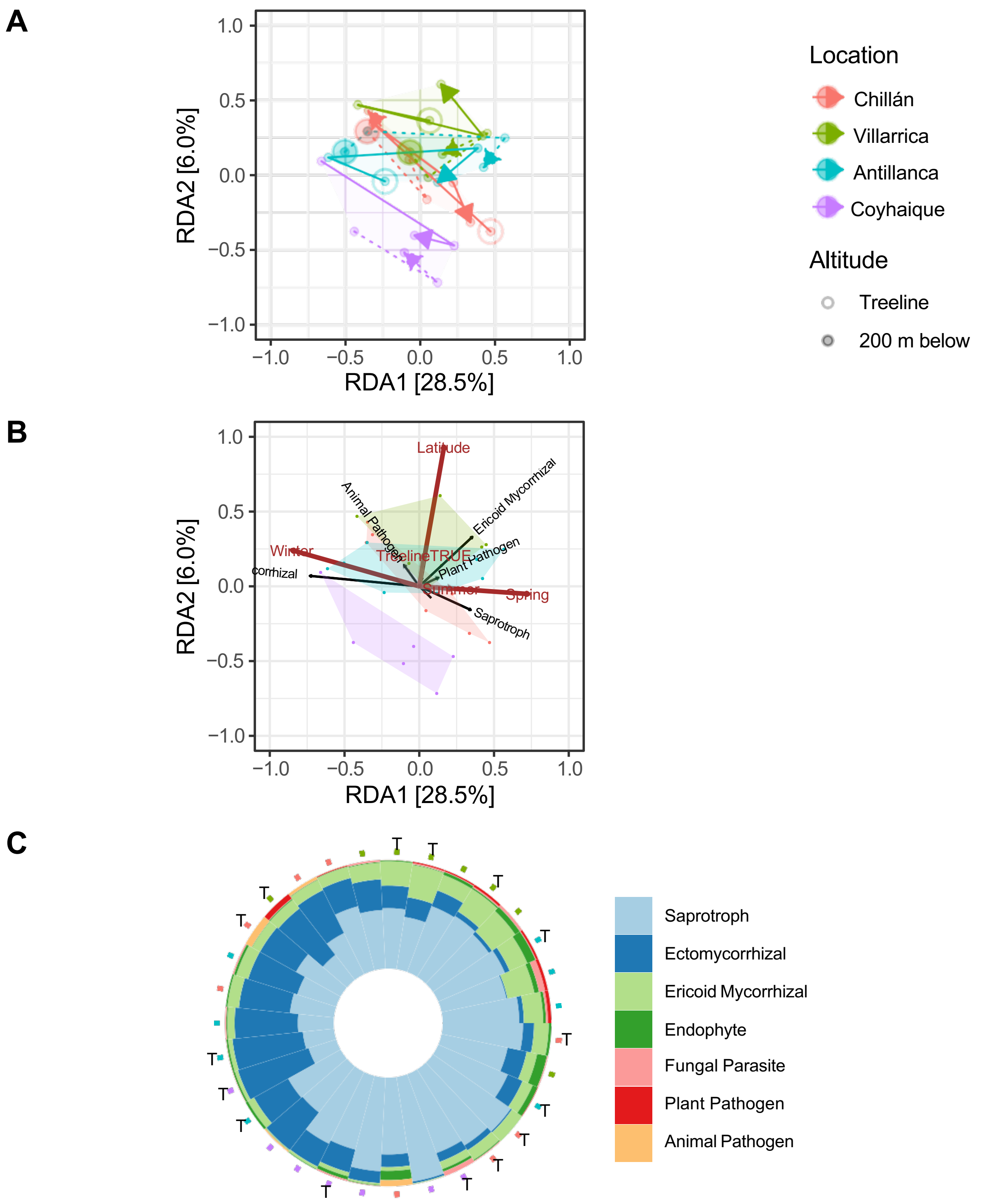

Figure 47: Transformation-based redundancy analysis (tb-RDA) triplot of the data set aggregated by guild. (A) Site scores from time series were connected forming temporal trajectories beginning in the fall (circles) and ending in the summer (arrowheads). Trajectories from the treeline were drawn with continuous lines and hollow circles, whereas those from 200 m below the treeline with broken lines and filled circles. (B) Guild scores as well as constraining variables of the same ordination were plotted separately for clarity. (C) An irisplot displaying the relative abundance of each guild further aids the interpretation of the guild composition of the ordinated samples. Triplot scaling was sample-oriented (i.e., type 2) trying to preserve Euclidean distance in the ordination space.

```
## Call: vegan::dbrda(formula = otu_table(phylo3.hell.summer) ~ ##
log10(Organic_matter) + log10(Nitrogen) + log10(Phosphorus) +
## log10(Sulfur), data = data.frame(sample_data(phylo3.hell.summer)), add ## = "lingoes",
comm = otu_table(phylo3.hell.summer))
##
##                    Inertia Proportion Rank
##  Total              3.795      1.000
##  Constrained        2.619      0.690     4
## Unconstrained       1.177      0.310     3
## Inertia is squared Euclidean distance ##
## Eigenvalues for constrained axes: ##
dbRDA1 dbRDA2 dbRDA3 dbRDA4
## 0.9877 0.8414 0.4339 0.3558 ##
## Eigenvalues for unconstrained axes: ## MDS1
      MDS2    MDS3
## 0.4522 0.3884 0.3359 ##
## Constant added to distances: 4.83226e-17
```

```
# summary(full) # Additional information and scores full$tot.chi # total
# variance, constrained and unconstrained full$CCA$tot.chi # variance explained # by constraints
full$CA$tot.chi # remaining unconstrained variance
```

Firstly, the results indicate all constraining variables together may explain up to 69 % of the variation in the fungi community data, and the first two constraining axes explain 48.2 %. The remaining 31 % of the variation cannot be explained by the explanatory matrix (unconstrained). Therefore, roughly half of the observed variation was the result of other environmental or stochastic processes, and this needs to be taken into account in all subsequent interpretations and visualizations presented below.

Of course some constraining variables in this full model were expected to be somewhat redundant and correlated with each other (Figure 48).

Correlated variables exacerbated the variance of model coefficients, which is undesirable. This effect was evidenced by variance inflation factors (VIF) that express how much the variance of regression coefficients is inflated by the presence of covariates. VIF increase as variables are correlated with each other.

VIF >= 20 indicate strong collinearity in constraining variables.

VIF >=10 potentially of concern and should be looked at.

```
vif.cca(full)
```

```
##   log10(Organic_matter)      log10(Nitrogen)     log10(Phosphorus)
##               14.008445             7.903064              3.986518
##           log10(Sulfur)
##                2.157976
```

11.2.1. *Tests of significance*

The permutation scheme was set to free permutations because there is no longer need to model time series and there is no replication at each sampling site.

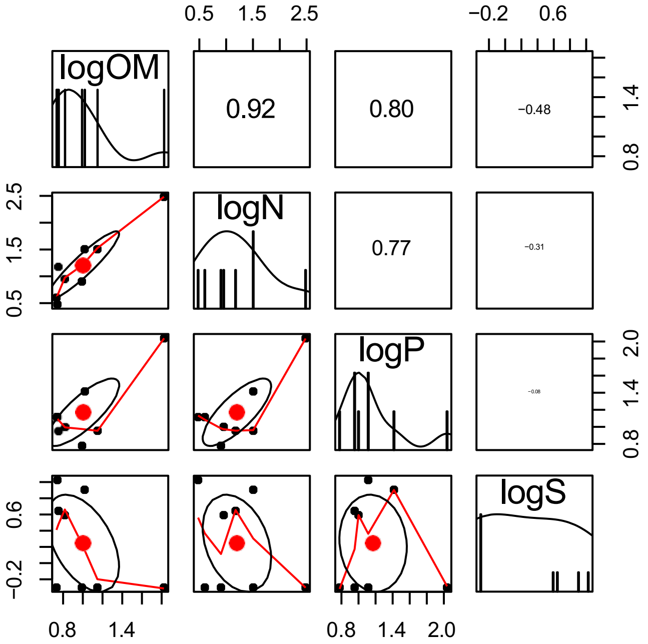

Figure 48: Pairwise correlation plot between constrainig variables before model selection.

```
# Test the significance of the axes
perm <- anova.cca(full, by = "axis")

# Estimated the standardized effect sizes (SES) and confidence intervals
pstat <- permustats(perm)

summary(pstat, interval = 0.95)
```

```
##
##          statistic     SES    mean lower  median   upper Pr(perm)
## dbRDA1      2.5185  2.5649  1.6213        1.6131  2.2082    0.004 **
## dbRDA2      2.1454  1.1778  1.5596        1.4495  2.4387    0.137
## dbRDA3      1.1064 -0.6772  1.3494        1.3273  1.9373    0.757
## dbRDA4      0.9072 -0.3004  1.0766        1.0265  2.0586    0.578
## ---
## Signif. codes:  0 '***' 0.001 '**' 0.01 '*' 0.05 '.' 0.1 ' ' 1 ##
## (Interval (Upper - Lower) = 0.95)
```

Only the first constraining axis was significant.
The marginal effect of each model term while controlling for the effects of all others:

```
# Significance tests for each marginal term in a model with all other terms # included (order does
not matter).
perm <- anova.cca(full, by = "margin", scope = formula(full))

perm
```

```
## Permutation test for dbrda under reduced model
```

```
## Marginal effects of terms ##
Permutation: free
## Number of permutations: 999 ##
## Model: vegan::dbrda(formula = otu_table(phylo3.hell.summer) ~ log10(Organic_matter) + log10(Nitrog ##       Df SumOfSqs
                                             F Pr(>F)
## log10(Organic_matter)  1  0.37095 0.9459  0.499
## log10(Nitrogen)                  1  0.58045 1.4801  0.104
## log10(Phosphorus)                1  0.65254 1.6639  0.049 *
## log10(Sulfur)                    1  0.38293 0.9764  0.525
## Residual                         3  1.17653
## ---
## Signif. codes:  0 '***' 0.001 '**' 0.01 '*' 0.05 '.' 0.1 ' ' 1
```

Only log10(Phosphorus) was significant at explaining the fungi community composition, as inferred from PERMANOVA of the marginal effect of each model term while controlling for the effects of all others (P < 0.05).

11.3. Model selection

Model selection was carried out to simplify the full model into a more parsimonious, less redundant model. The function vegan::ordistep() was used, with bidirectional (default) stepwise search of the best set of explanatory variables, and the permutation scheme described above.

```
best <- vegan::ordistep(full, direction = "both", trace = F) best
```

```
## Call: vegan::dbrda(formula = otu_table(phylo3.hell.summer) ~ ##
log10(Nitrogen) + log10(Phosphorus), data =
## data.frame(sample_data(phylo3.hell.summer)), add = "lingoes", comm = ##
otu_table(phylo3.hell.summer))
##
##                      Inertia Proportion Rank
##  Total                 3.7953       1.0000
##  Constrained           1.8099       0.4769      2
##  Unconstrained         1.9854       0.5231      5
## Inertia is squared Euclidean distance ##
## Eigenvalues for constrained axes:
## dbRDA1 dbRDA2
## 0.9801 0.8298 ##
## Eigenvalues for unconstrained axes:
##   MDS1   MDS2   MDS3   MDS4   MDS5  ##
0.5170 0.4369 0.4001 0.3829 0.2485 ##
## Constant added to distances: 4.83226e-17
```

Following bidirectional stepwise model selection, we concluded that the best model explaining the fungi community composition included log10(Nitrogen) and log10(Phosphorus).

The best tb-RDA model explained 47.7 % of the variance (R^2), though penalizing for the number of predictors the proportion drops to 26.8 % (i.e., Adj. R^2).

All constraining variables together may explain up to 47.7 % of the variation in the fungi community data, and the first two constraining axes explain 47.7 %. The remaining 52.3 % of the variation cannot be explained by the explanatory matrix (unconstrained). Therefore, about half of the observed variation was the result of other environmental or stochastic processes, and this needs to be taken into account in all subsequent interpretations and visualizations presented below.

Variance inflation factors (VIF) were in the range 2.44, 2.44 in the final model indicating no concern of collinearity between explanatory variables.

```
vif.cca(best)
```

```
##     log10(Nitrogen) log10(Phosphorus)
##             2.43659           2.43659
```

11.3.1. Tests of significance

The tests of significance were done through Permutational Multivariate Analyses of Variance (PER- MANOVAs), imposing no restriction to the permutation schema (default settings).

A few complementary tests revealed different statistical properties of the fitted model. For example, a test of the significance of the constrained axes:

```
# Test the significance of the axes
perm <- anova.cca(best, by = "axis")

# Estimated the standardized effect sizes (SES) and confidence intervals
pstat <- permustats(perm)
summary(pstat, interval = 0.95)
```

```
##
##          statistic    SES   mean lower median  upper Pr(perm)
## dbRDA1      2.4682 4.0200 1.2250        1.2065 1.7970    0.001 ***
## dbRDA2      2.0898 2.7503 1.0172        0.9283 1.7232    0.028 *
## ---
## Signif. codes:  0 '***' 0.001 '**' 0.01 '*' 0.05 '.' 0.1 ' ' 1 ##
## (Interval (Upper - Lower) = 0.95)
```

The two constraining axes were statistically significant (P < 0.05).

PERMANOVA of the marginal effect of each model term while controlling for the effects of all others indicated that all model terms of the best model were statistically significant (P < 0.05; Figures 49 and 50).

```
# Significance tests for each marginal term in a model with all other terms # included (order does
not matter).
perm <- anova.cca(best, by = "margin", scope = formula(best)) perm
```

```
## Permutation test for dbrda under reduced model ##
Marginal effects of terms
## Permutation: free
## Number of permutations: 999 ##
## Model: vegan::dbrda(formula = otu_table(phylo3.hell.summer) ~ log10(Nitrogen) + log10(Phosphorus), ##       Df SumOfSqs
                                  F Pr(>F)
## log10(Nitrogen)                1  0.97897 2.4654  0.001 ***
```

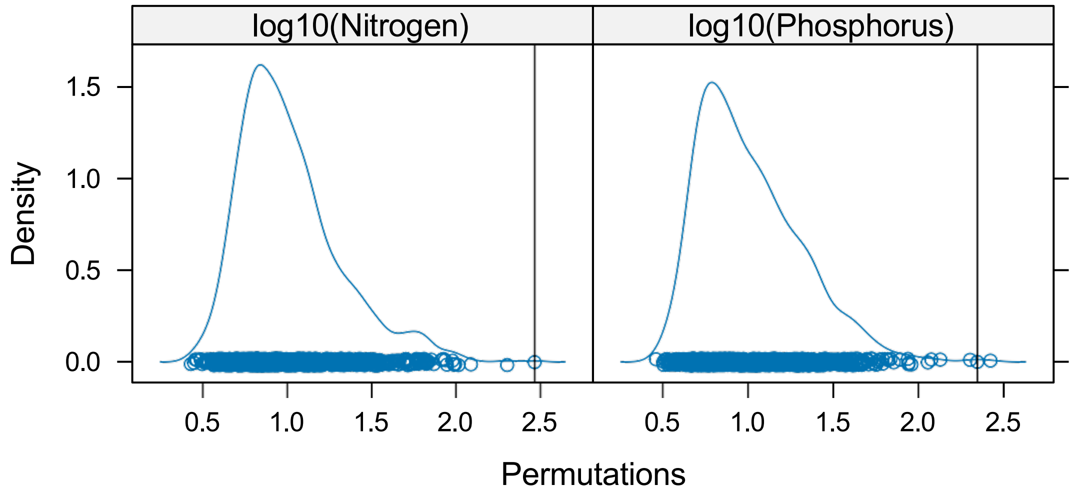

Figure 49: Density distributions of marginal psudo-F values by model term under the null expectations (permuted). Vertical lines indicates the observed values.

```
## log10(Phosphorus)  1  0.93088 2.3443  0.002 **
## Residual                    5  1.98541
## ---
## Signif. codes:  0 '***' 0.001 '**' 0.01 '*' 0.05 '.' 0.1 ' ' 1

##
##                              statistic     SES   mean lower median  upper Pr(perm)
## log10(Nitrogen)                 2.4654 4.8841 1.0131         0.9513 1.6237    0.001 ***
## log10(Phosphorus)               2.3443 4.4467 1.0115         0.9527 1.5859    0.002 **
## ---
## Signif. codes:  0 '***' 0.001 '**' 0.01 '*' 0.05 '.' 0.1 ' ' 1
##
## (Interval (Upper - Lower) = 0.95)
```

11.3.2. Soil tb-RDA plots by taxonomic rank

The analyses just presented were conducted at the finest taxonomic scale that corresponds to ASVs. The corresponding plot of the selected model is presented in Figure 51.

Follow a series of figures, each portraying a similar analysis applied at each taxonomic rank, from Phylum to Species (Figures 52 to 57). Note, however, that tests of significance have not been carried out at these levels of taxonomic resolution (tests were conducted at the ASV-level).

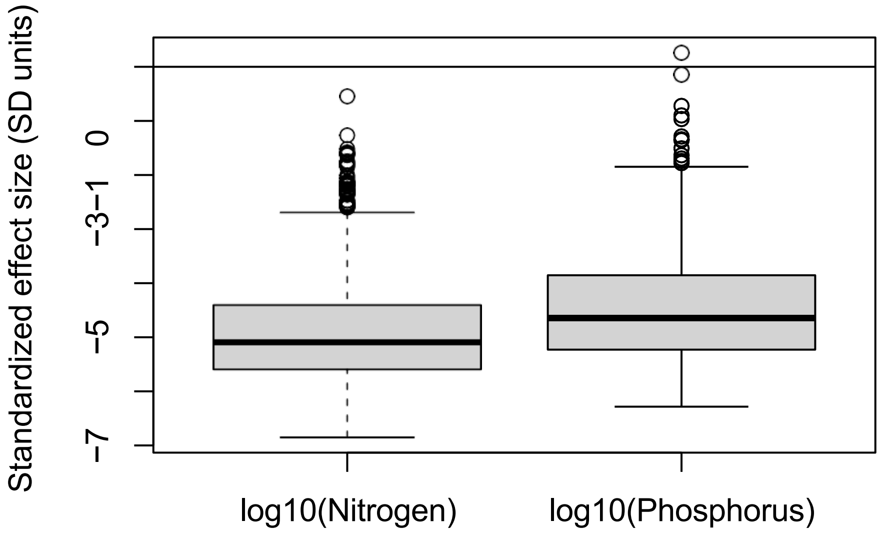

Figure 50: Standardized marginal effect sizes based on psudo-F values of permutated minus the observed values (expressed in SD units).

12. Effects of soil nutrient content on guilds composition

In this section we used a similar analytic strategy applied to a subset of the phylo3_guilded data set retaining only Summer samples, which had soil nutrient content variables.

Much of the code (and R object's names) were recycled. Please see the *Constrained ordination: transformation-based redundancy analysis (tb-RDA)* section above for explanations of the analytic strategy. Here only essential results are presented.

```
phydata <- phylo3_guilded %>%
      microViz::tax_agg(rank = "Guild", force = T)

# Hellinger transform
phylo3.hell <- transform_sample_counts(phydata, function(x) sqrt(x/sum(x)))

# Filtered summer samples
phylo3.hell.summer <- subset_samples(phylo3.hell, (Season %in% "Summer"))
```

12.1. Main gradint length estimation by Detrended Correspondence Analysis (DCA)

```
# Detrended Correspondence analysis of the Summer data set library(vegan)

# Check the length of the main gradient.
vegan::decorana(otu_table(phylo3.hell.summer))
```

```
##
## Call:
## vegan::decorana(veg = otu_table(phylo3.hell.summer)) ##
## Detrended correspondence analysis with 26 segments. ## Rescaling
of axes with 4 iterations.
```

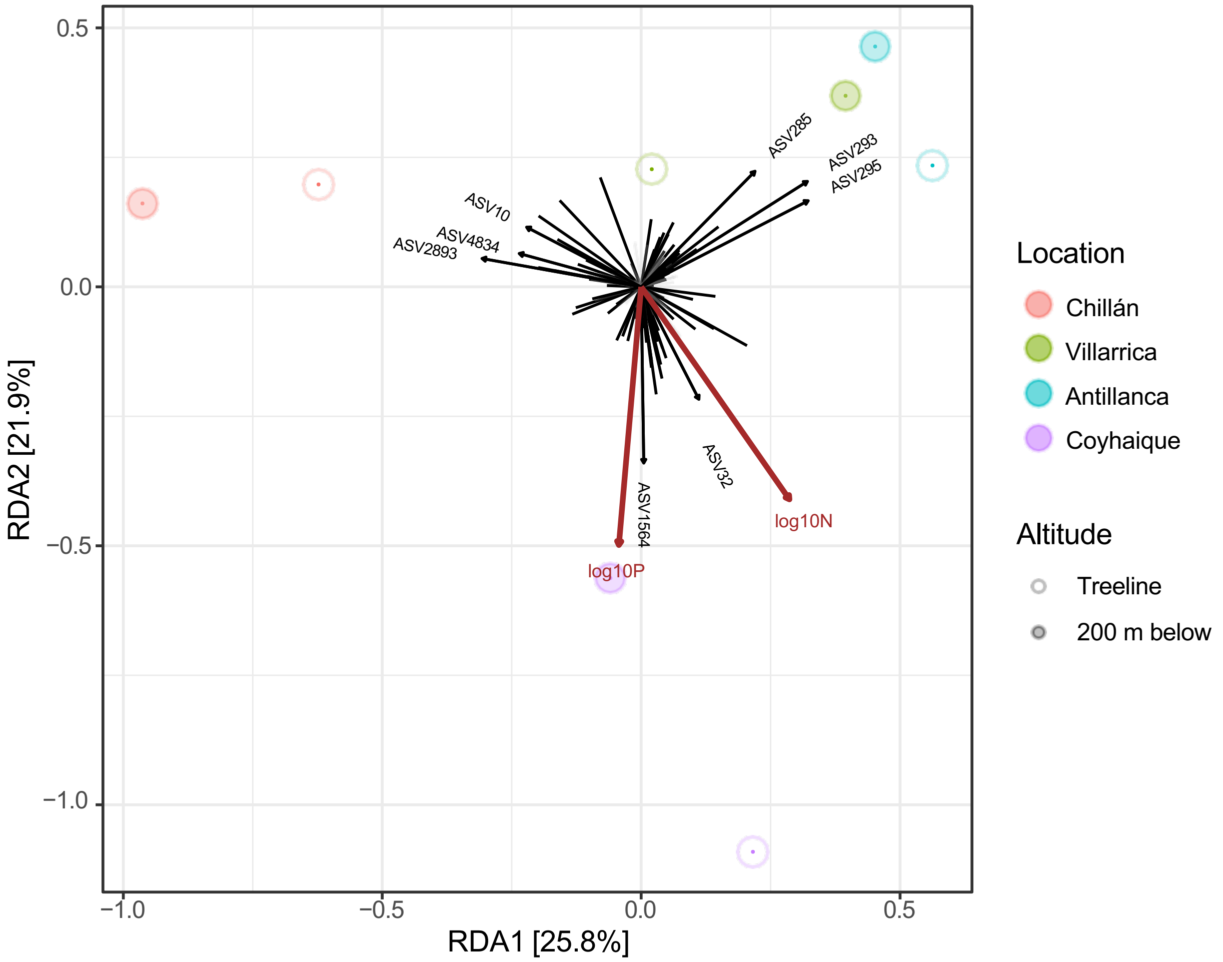

Figure 51: Transformation-based redundancy analysis (tb-RDA) triplots of summer data with edaphic variables. Nutrient content variables (OM, N, P, S,) were screened through a stepwise selection procedure, and the selected model with two variables (N nd P) was portrayed here.

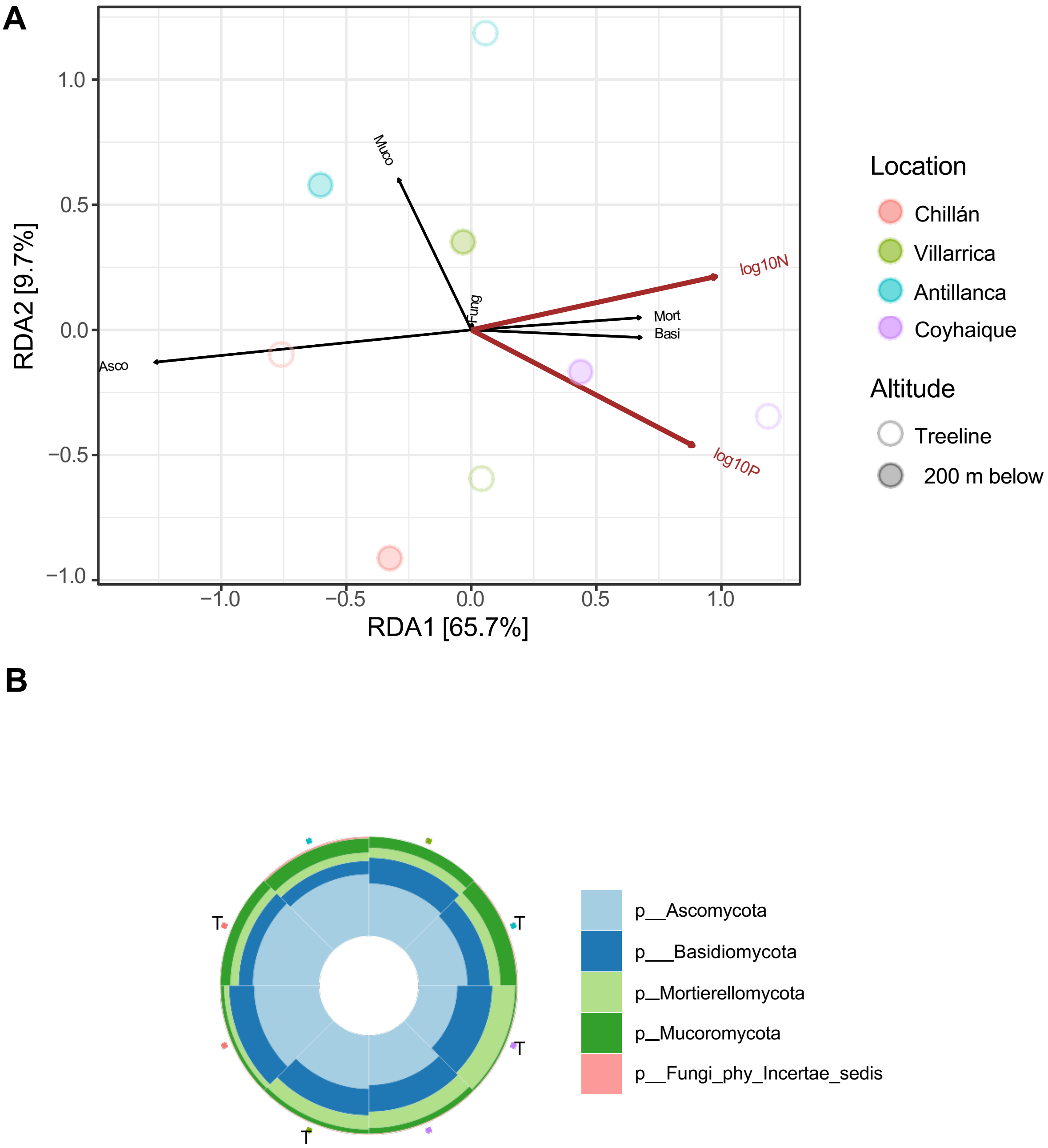

Figure 52: Phylum-level analysis of the microbiom of summer samples as a function of selected soil nutrient content variables.
(A) Transformation-based redundancy analysis (tb-RDA) triplot showing site scores (circles), taxon scores (black vectors), and constraining variables (red vectors). (B) An irisplot displaying the relative abundance of each taxon further aids the interpretation of the community composition of the ordinated samples. Triplot scaling was sample-oriented (i.e., type 2) trying to preserve Euclidean distance in the ordination space.

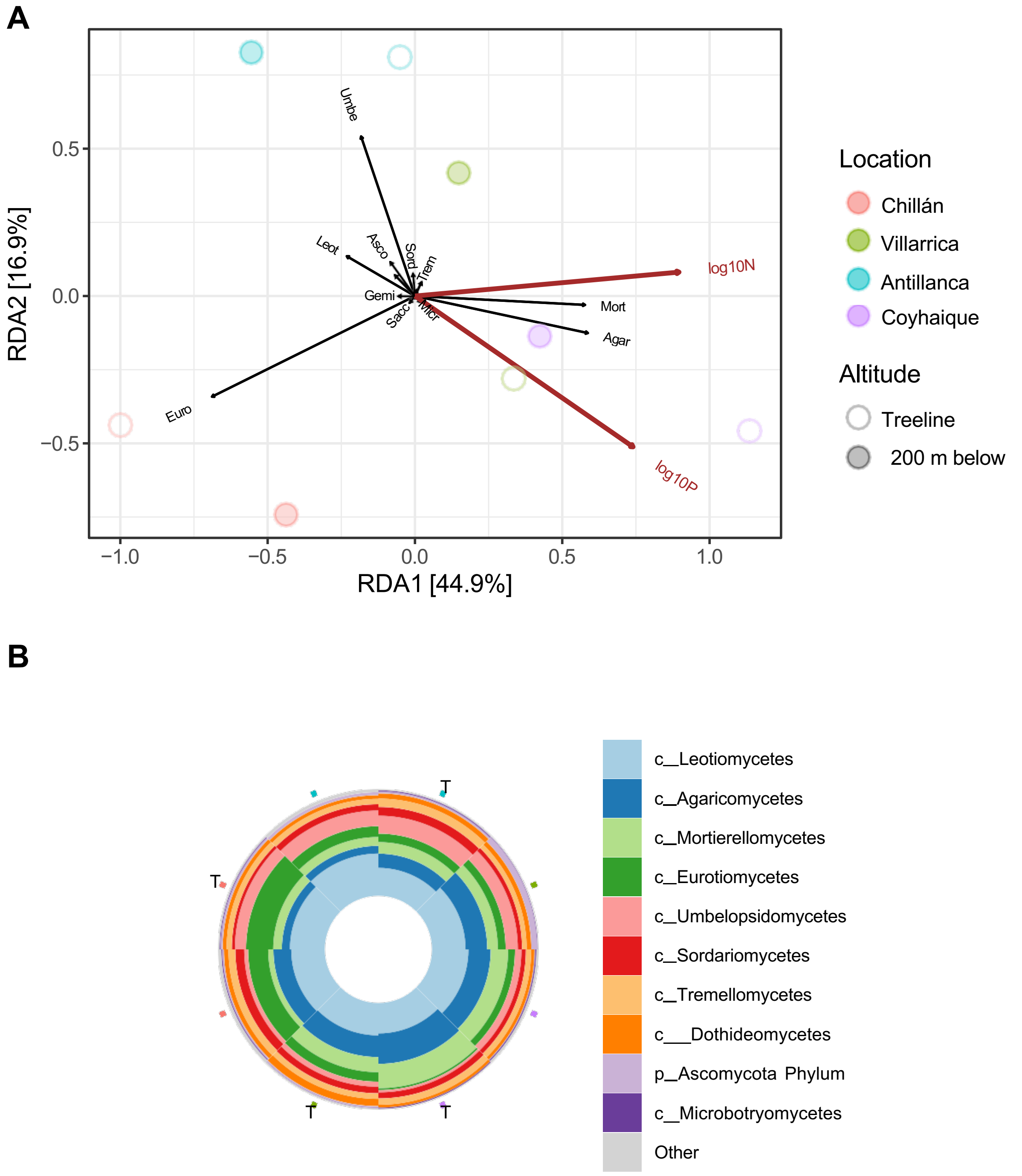

Figure 53: Class-level analysis of the microbiom of summer samples as a function of selected soil nutrient content variables. Other conventions as in Figure 52.

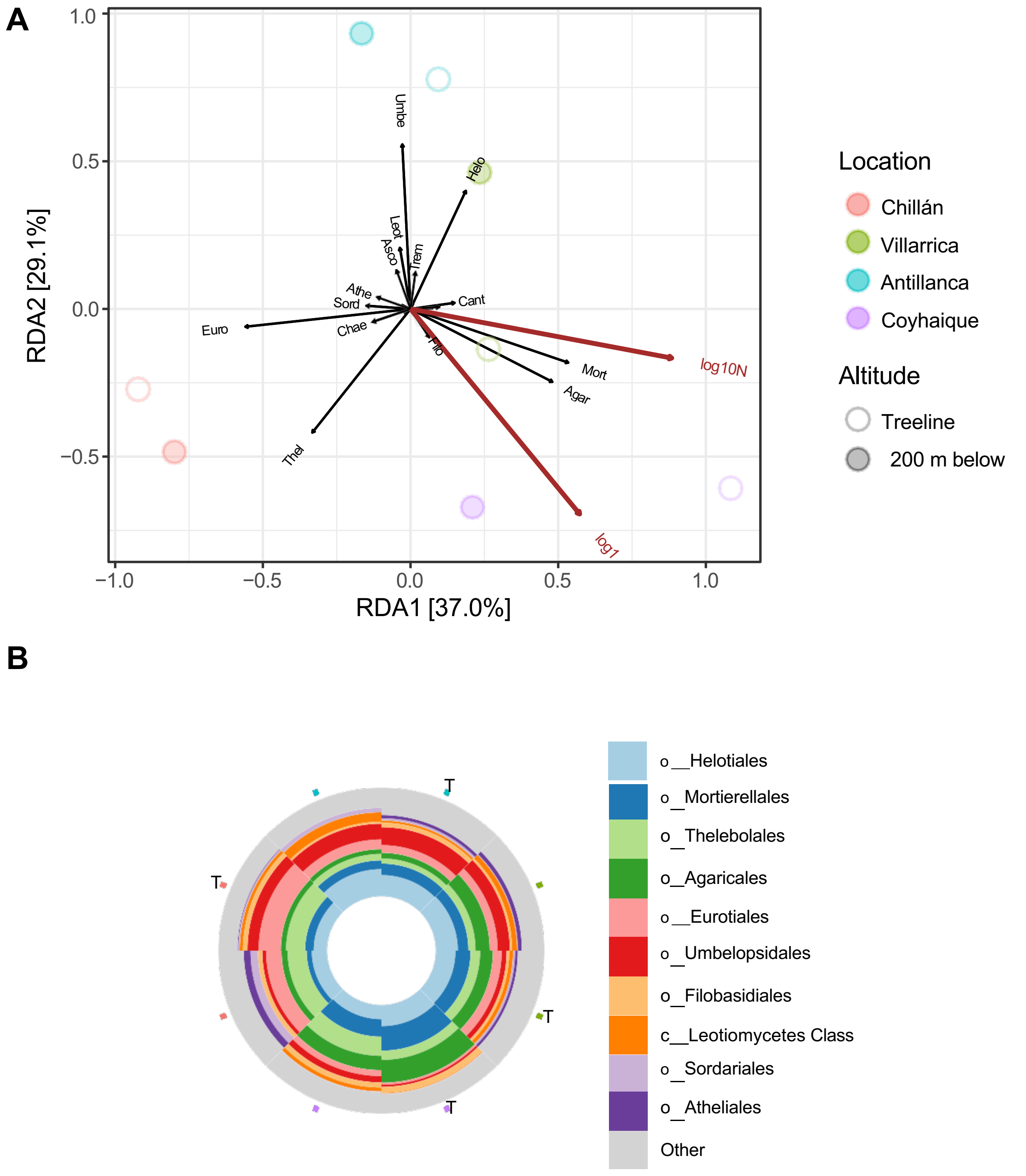

Figure 54: Order-level analysis of the microbiom of summer samples as a function of selected soil nutrient content variables. Other conventions as in Figure 52.

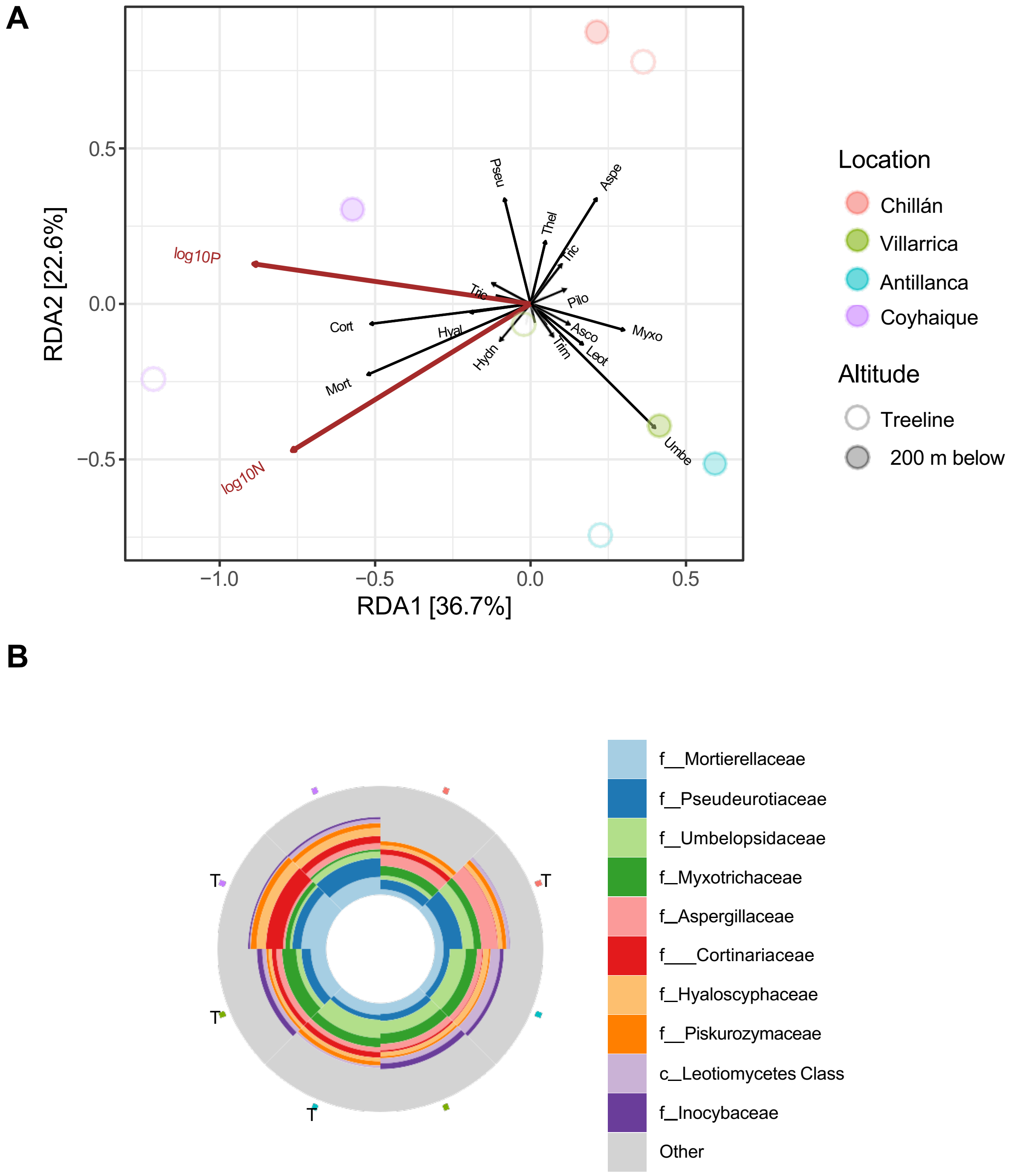

Figure 55: Family-level analysis of the microbiom of summer samples as a function of selected soil nutrient content variables. Other conventions as in Figure 52.

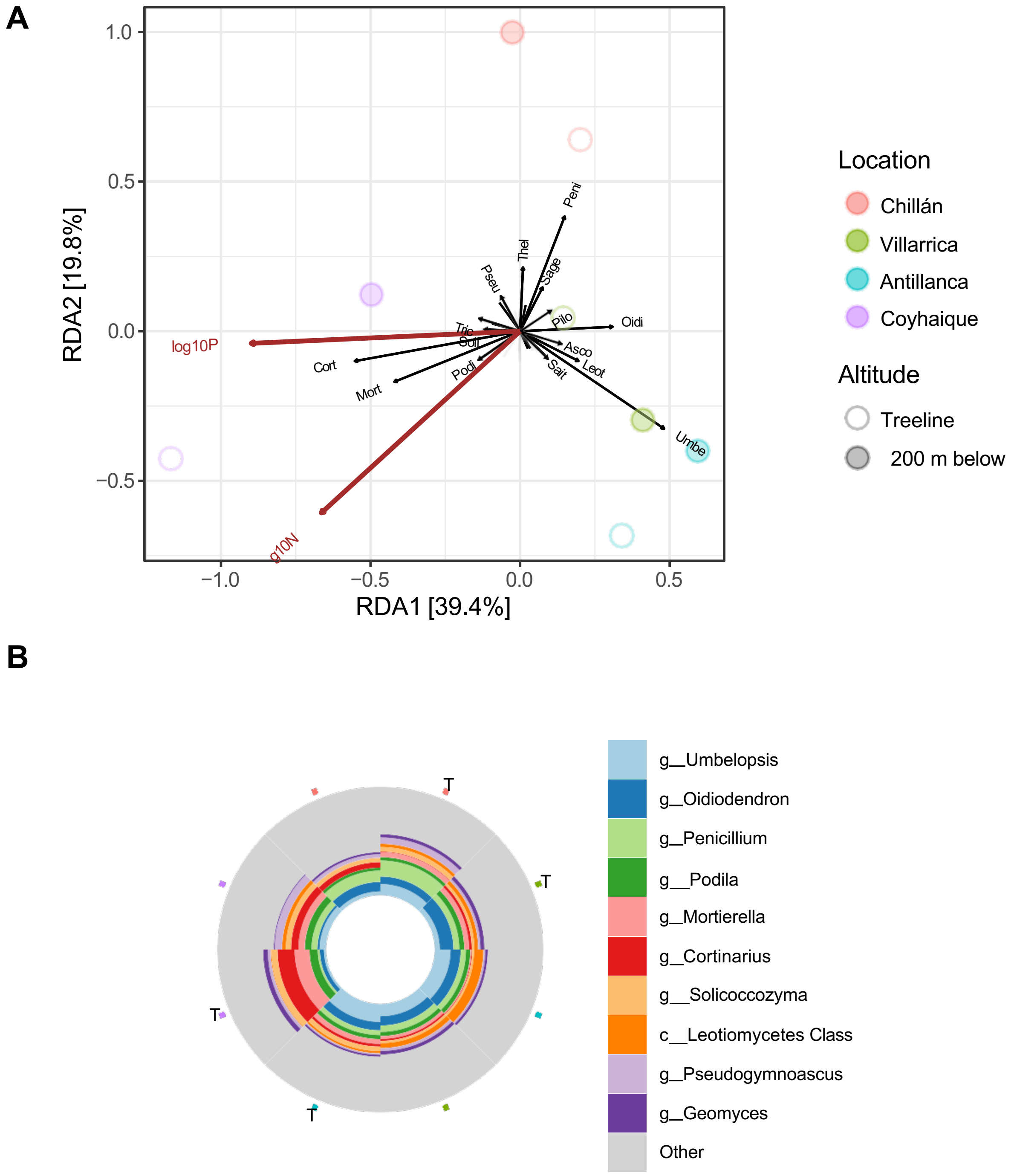

Figure 56: Genus-level analysis of the microbiom of summer samples as a function of selected soil nutrient content variables. Other conventions as in Figure 52.

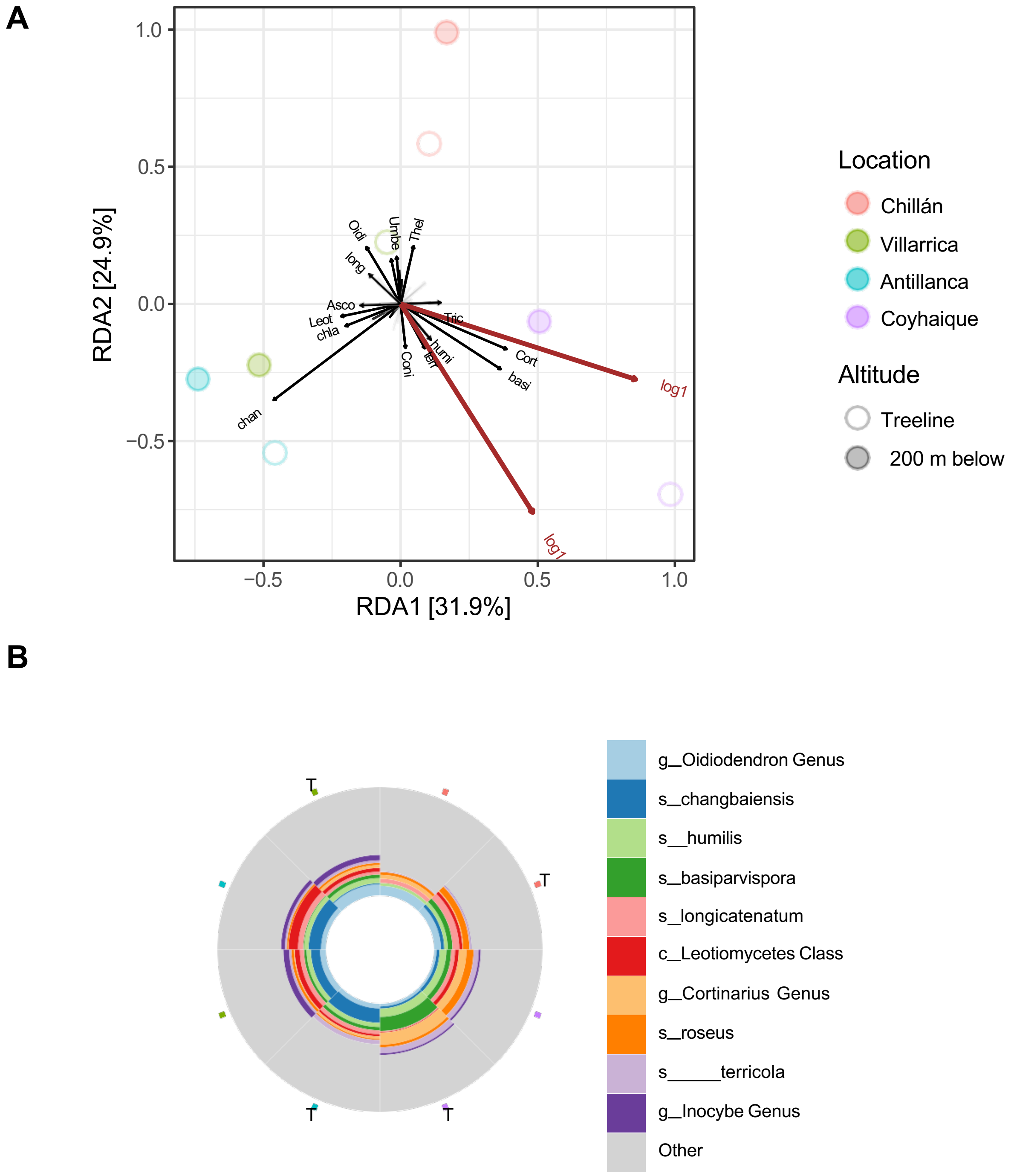

Figure 57: Species-level analysis of the microbiom of summer samples as a function of selected soil nutrient content variables. Other conventions as in Figure 52.

```
## Total inertia (scaled Chi-square): 0.1635 ##
##                          DCA1      DCA2      DCA3      DCA4
## Eigenvalues          0.08363  0.010595  0.010344  0.016266
## Additive Eigenvalues 0.08363  0.010363  0.010659  0.020472
## Decorana values      0.09746  0.007956  0.002992  0.000585
## Axis lengths         0.89775  0.300738  0.281513  0.407111
```

The length of our *first* DCA axis was 0.90 SD, and we therefore proceeded with linear constrained ordination.

12.2. Full db-RDA model

The following distance-based redundancy analysis (tb-RDA) was fitted to the data. Initially, all nutrient content variables were included in the model (log10(P), log10(N), log10(S), and log10(OM)).

```
full <- vegan::dbrda(otu_table(phylo3.hell.summer) ~
            log10(Organic_matter)+ log10(Nitrogen)+ log10(Phosphorus)+ log10(Sulfur), data =
            data.frame(sample_data(phylo3.hell.summer)),
            add = "lingoes", # to correct for negative eigenvalues
            comm = otu_table(phylo3.hell.summer)) # to produce spp scores

full
```

```
## Call: vegan::dbrda(formula = otu_table(phylo3.hell.summer) ~ ##
log10(Organic_matter) + log10(Nitrogen) + log10(Phosphorus) +
## log10(Sulfur), data = data.frame(sample_data(phylo3.hell.summer)), add ## = "lingoes",
comm = otu_table(phylo3.hell.summer))
##
##                Inertia Proportion Rank
## Total           0.4004     1.0000
## Constrained     0.2711     0.6772    4
## Unconstrained   0.1292     0.3228    3
## Inertia is squared Euclidean distance ##
## Eigenvalues for constrained axes: ##
dbRDA1  dbRDA2  dbRDA3  dbRDA4  ##
0.17567 0.07193 0.01807 0.00547 ##
## Eigenvalues for unconstrained axes: ## MDS1
      MDS2      MDS3
## 0.08195 0.04172 0.00554 ##
## Constant added to distances: 9.311863e-18
```

```
# summary(full) # Additional information and scores full$tot.chi # total
# variance, constrained and unconstrained full$CCA$tot.chi # variance explained # by constraints
full$CA$tot.chi # remaining unconstrained variance
```

Firstly, the results indicate all constraining variables together may explain up to 67.7 % of the variation in the fungi community data, and the first two constraining axes explain 61.8 %. The remaining 32.3 % of the variation cannot be explained by the explanatory matrix (unconstrained).

Correlated variables exacerbated the variance of model coefficients, which is undesirable. This effect was evidenced by variance inflation factors (VIF) that express how much the variance of regression coefficients is inflated by the presence of covariates. VIF increase as variables are correlated with each other.

VIF >= 20 indicate strong collinearity in constraining variables.

VIF >=10 potentially of concern and should be looked at.

```
vif.cca(full)
```

```
##      log10(Organic_matter)       log10(Nitrogen)       log10(Phosphorus)
##                  14.008445              7.903064                3.986518
##              log10(Sulfur)
##                   2.157976
```

12.2.1. Tests of significance

The permutation scheme was set to free permutations because there is no longer need to model time series and there is no replication at each sampling site.

```
# Test the significance of the axes
perm <- anova.cca(full, by = "axis")

# Estimated the standardized effect sizes (SES) and confidence intervals
pstat <- permustats(perm)

summary(pstat, interval = 0.95)
```

```
##
##          statistic      SES    mean lower  median    upper Pr(perm)
## dbRDA1      4.0785  0.4669  3.1117          2.6008  6.5042    0.223
## dbRDA2      1.6698 -0.5468  2.8038          2.2081  6.8310    0.650
## dbRDA3      0.4196 -1.1330  1.9101          1.5634  4.4274    0.962
## dbRDA4      0.1270 -0.8412  1.3250          0.9056  3.9902    0.954
##
## (Interval (Upper - Lower) = 0.95)
```

No constraining axis was significant.

The marginal effect of each model term while controlling for the effects of all others:

```
# Significance tests for each marginal term in a model with all other terms # included (order does
not matter).
perm <- anova.cca(full, by = "margin", scope = formula(full))

perm
```

```
## Permutation test for dbrda under reduced model ##
Marginal effects of terms
## Permutation: free
## Number of permutations: 999 ##
## Model: vegan::dbrda(formula = otu_table(phylo3.hell.summer) ~ log10(Organic_matter) + log10(Nitrog ##       Df SumOfSqs
                                  F Pr(>F)
## log10(Organic_matter)  1 0.039933 0.9271  0.468
```

```
## log10(Nitrogen)          1  0.037415 0.8686  0.515
## log10(Phosphorus)        1  0.037401 0.8683  0.503
## log10(Sulfur)            1  0.123139 2.8588  0.092 .
## Residual                 3  0.129219
## ---
## Signif. codes:  0 '***' 0.001 '**' 0.01 '*' 0.05 '.' 0.1 ' ' 1
```

No soil nutrient content variable was significant at explaining the fungi guild composition, as inferred from PERMANOVA of the marginal effect of each model term while controlling for the effects of all others (P > 0.05).

12.3. Model selection

Model selection was carried out to simplify the full model into a more parsimonious, less redundant model. The function vegan::ordistep() was used, with bidirectional (default) stepwise search of the best set of explanatory variables, and the permutation scheme described above.

```
best <- vegan::ordistep(full, direction = "both", trace = F) best
```

```
## Call: vegan::dbrda(formula = otu_table(phylo3.hell.summer) ~
## log10(Sulfur), data = data.frame(sample_data(phylo3.hell.summer)), add ## = "lingoes",
comm = otu_table(phylo3.hell.summer))
##
##                   Inertia Proportion Rank
##  Total             0.4004      1.0000
##  Constrained       0.1215      0.3034     1
##  Unconstrained     0.2789      0.6966     6
## Inertia is squared Euclidean distance ##
## Eigenvalues for constrained axes:
##    dbRDA1
##    0.12147
##
## Eigenvalues for unconstrained axes:
##      MDS1      MDS2      MDS3      MDS4      MDS5      MDS6  ##
0.16043 0.07926 0.01989 0.01086 0.00776 0.00070 ##
## Constant added to distances: 9.311863e-18
```

Following bidirectional stepwise model selection, we concluded that the best model explaining the fungi guild composition included only log10(Sulfur) and log10(Phosphorus).

The best tb-RDA model explained 30.3 % of the variance (R^2), though penalizing for the number of predictors the proportion drops to 18.7 % (i.e., Adj. R^2).

All constraining variables together may explain up to 30.3 % of the variation in the fungi community data, and the first two constraining axes explain NA %. The remaining 69.7 % of the variation cannot be explained by the explanatory matrix (unconstrained). Therefore, about half of the observed variation was the result of other environmental or stochastic processes, and this needs to be taken into account in all subsequent interpretations and visualizations presented below.

Variance inflation factors (VIF) were in the range 1, 1 in the final model indicating no concern of collinearity between explanatory variables.

```r
vif.cca(best)
```

```
## log10(Sulfur)
##             1
```

12.3.1. Tests of significance

The tests of significance were done through Permutational Multivariate Analyses of Variance (PER- MANOVAs), imposing no restriction to the permutation schema (default settings).

A few complementary tests revealed different statistical properties of the fitted model. For example, a test of the significance of the constrained axes:

```r
# Test the significance of the axes
perm <- anova.cca(best, by = "axis")

# Estimated the standardized effect sizes (SES) and confidence intervals
pstat <- permustats(perm)
summary(pstat, interval = 0.95)
```

```
##
##          statistic      SES    mean lower median   upper Pr(perm) ##
dbRDA1      2.6133 1.8492 1.0483          0.8151 2.6576       0.054 .
## ---
## Signif. codes:  0 '***' 0.001 '**' 0.01 '*' 0.05 '.' 0.1 ' ' 1 ##
## (Interval (Upper - Lower) = 0.95)
```

The first constraining axes were statistically significant (P < 0.05).

PERMANOVA of the marginal effect of each model term while controlling for the effects of all others indicated that the effect of log10(Sulfur) was statistically significant (P < 0.05; Figures 58 and 59).

```r
# Significance tests for each marginal term in a model with all other terms # included (order does
not matter).
perm <- anova.cca(best, by = "margin", scope = formula(best)) perm
```

```
## Permutation test for dbrda under reduced model
## Marginal effects of terms
## Permutation: free
## Number of permutations: 999
##
## Model: vegan::dbrda(formula = otu_table(phylo3.hell.summer) ~ log10(Sulfur), data = data.frame(sam
##                 Df SumOfSqs       F Pr(>F)
## log10(Sulfur)  1  0.12147 2.6133  0.057 .
## Residual        6  0.27889
## ---
## Signif. codes:  0 '***' 0.001 '**' 0.01 '*' 0.05 '.' 0.1 ' ' 1

##
##                 statistic      SES    mean lower median  upper Pr(perm)
## log10(Sulfur)      2.6133 1.7953 1.0721          0.8488 2.7130       0.057 .
## ---
```

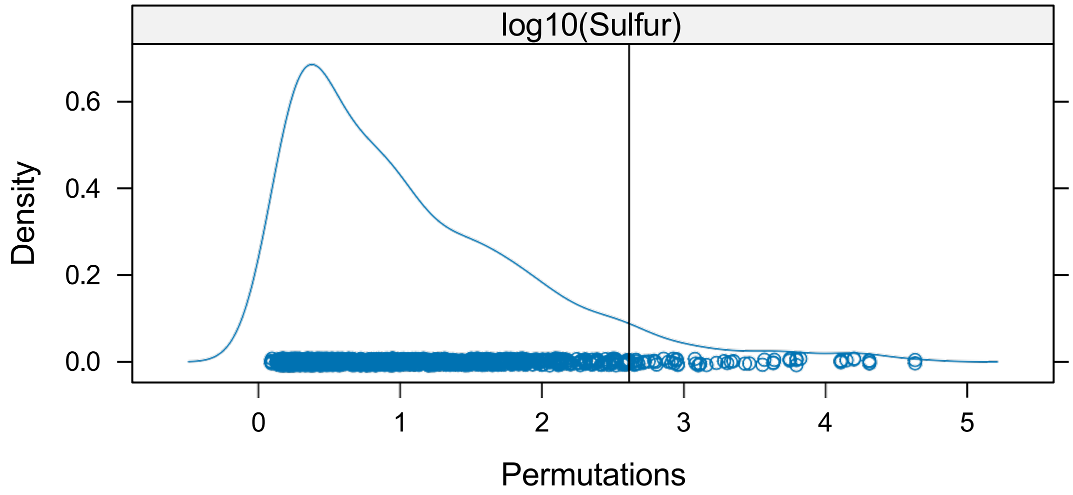

Figure 58: Density distributions of marginal psudo-F values by model term under the null expectations (permuted). Vertical lines indicates the observed values.

Signif. codes: 0 '***' 0.001 '**' 0.01 '*' 0.05 '.' 0.1 ' ' 1
(Interval (Upper - Lower) = 0.95)

12.3.2. Soil tb-RDA plots by taxonomic rank

The analyses just presented were conducted at the finest taxonomic scale that corresponds to ASVs. The corresponding plot of the selected model is presented in Figure 51.

12.4. Edaphic effects on guilds tb-RDA triplot

Figure 60 shows the results of the stepwise selected tb-RDA based on a the subset of data from the summer with edaphic and guilds information (i.e., phylo3_guilded-Summer data set). An additional figure is presented where and additional edaphic variable (N) ware included (Figure 61).

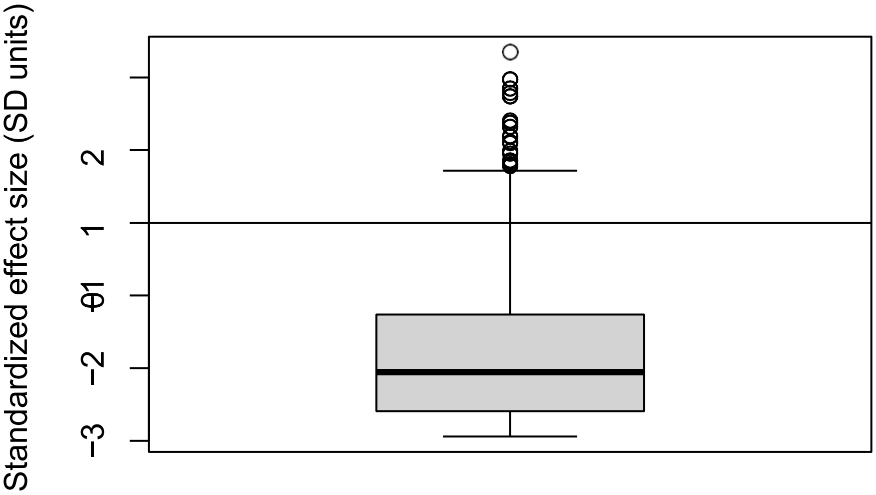

Figure 59: Standardized marginal effect sizes based on psudo-F values of permutated minus the observed values (expressed in SD units).

##	Location	Season	Altitude	Latitude	Longitude	T_min	T_mean ##
CHT.Summer	Chillán	Summer	Treeline	-36.90613	-71.39394	1.7	10.6
## CHBT.Summer	Chillán	Summer	Below treeline	-36.90548	-71.40683	2.9	11.9
## VT.Summer	Villarrica	Summer	Treeline	-39.39359	-71.96410	2.5	10.9
## VBT.Summer	Villarrica	Summer	Below treeline	-39.38123	-71.97063	3.5	11.9
## AT.Summer	Antillanca	Summer	Treeline	-40.78633	-72.19268	4.1	10.9
## ABT.Summer	Antillanca	Summer	Below treeline	-40.77709	-72.19871	4.5	11.3
## CT.Summer	Coyhaique	Summer	Treeline	-45.51626	-72.04409	3.8	8.8
## CBT.Summer	Coyhaique	Summer	Below treeline	-45.52073	-72.05089	5.2	10.1

##	T_max	Organic_matter	Nitrogen	Phosphorus	Sulfur	spsample
## CHT.Summer	19.6	5.40	4	13	0.50	CHT.Summer
## CHBT.Summer	20.9	5.52	3	13	10.60	CHBT.Summer
## VT.Summer	19.4	6.57	9	10	3.91	VT.Summer
## VBT.Summer	20.4	5.67	15	9	4.41	VBT.Summer
## AT.Summer	17.8	14.05	32	9	0.50	AT.Summer
## ABT.Summer	18.2	9.78	8	6	0.50	ABT.Summer
## CT.Summer	13.9	66.53	303	109	0.50	CT.Summer
## CBT.Summer	15.0	10.43	32	26	8.04	CBT.Summer

##	Altitude2	plot	log10N	log10P	log10S
## CHT.Summer	AT	Chillán.Treeline	0.6020600	1.1139434	-0.3010300
## CHBT.Summer	BT	Chillán.Below treeline	0.4771213	1.1139434	1.0253059
## VT.Summer	AT	Villarrica.Treeline	0.9542425	1.0000000	0.5921768
## VBT.Summer	BT	Villarrica.Below treeline	1.1760913	0.9542425	0.6444386
## AT.Summer	AT	Antillanca.Treeline	1.5051500	0.9542425	-0.3010300
## ABT.Summer	BT	Antillanca.Below treeline	0.9030900	0.7781513	-0.3010300
## CT.Summer	AT	Coyhaique.Treeline	2.4814426	2.0374265	-0.3010300
## CBT.Summer	BT	Coyhaique.Below treeline	1.5051500	1.4149733	0.9052560

13. Distance Threshold Network

Below a distance threshold network of the speudoreplica-combined dataset, using Hellinger distance (Figure 62).

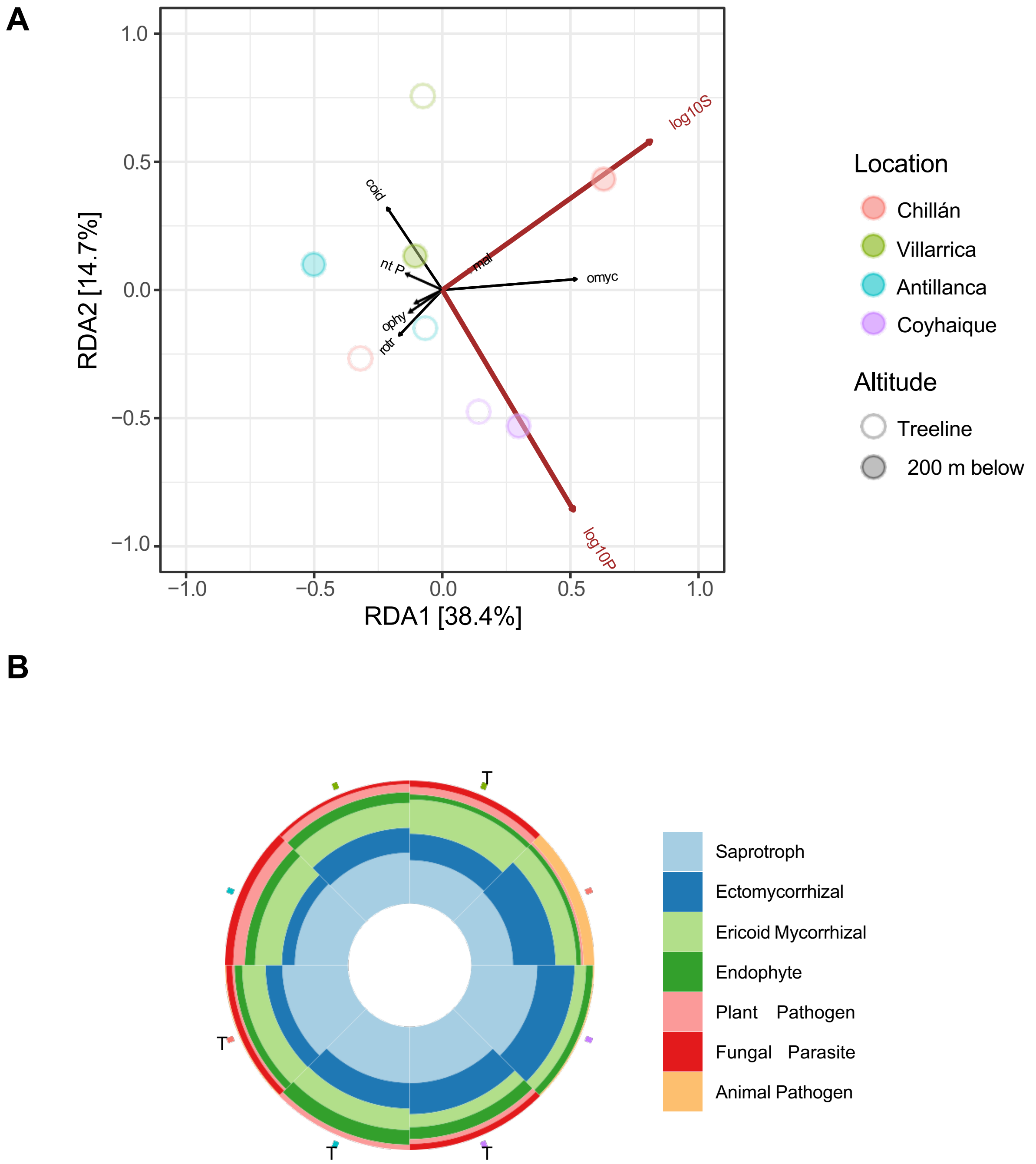

Figure 60: Transformation-based redundancy analysis (tb-RDA) triplots of summer guilds data with edaphic variables. Nutrient content variables (OM, N, P, S,) were screened through a stepwise selection procedure, and the selected model with two variables (S nd P) was portrayed here.

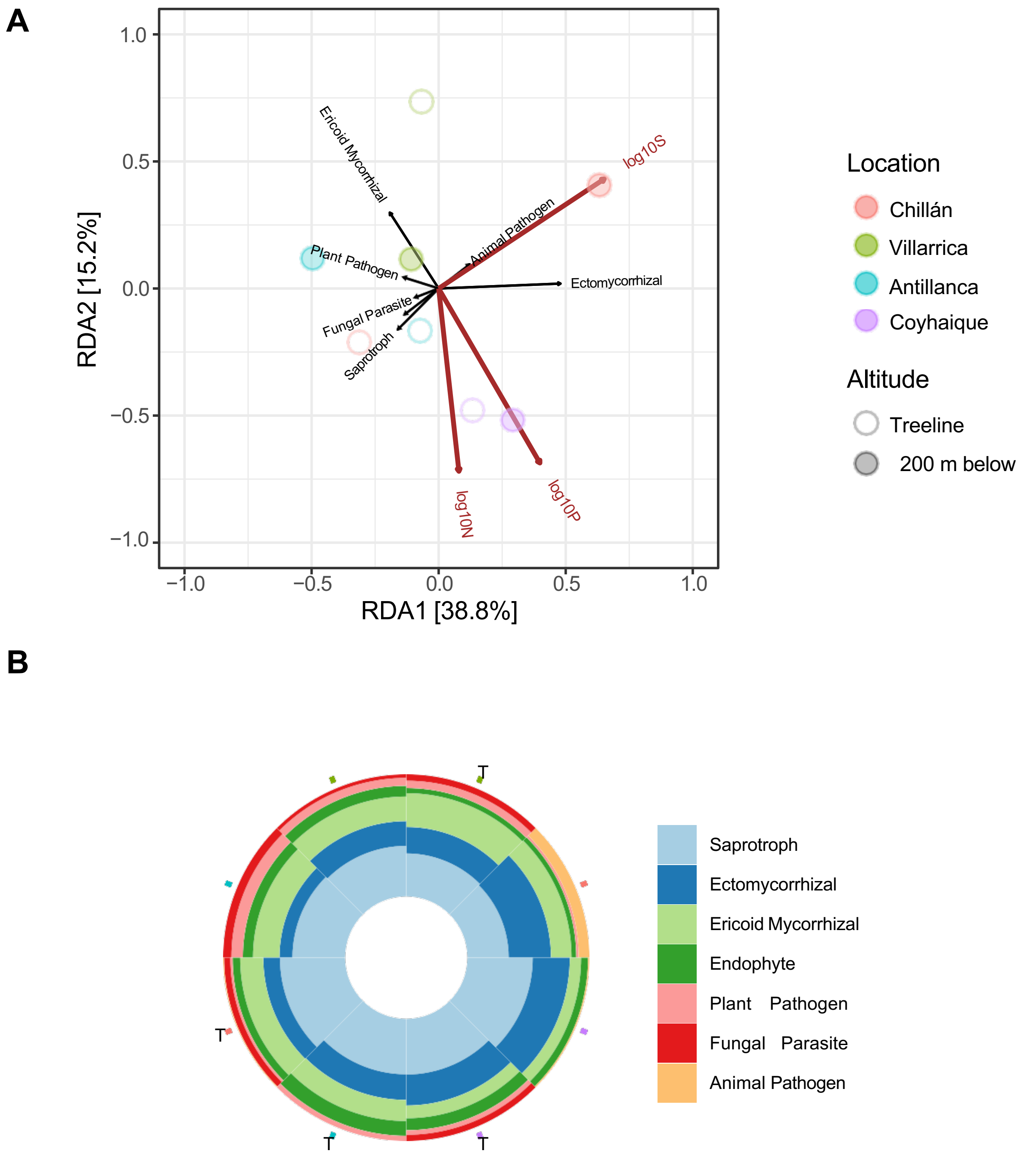

Figure 61: Similar as in Figure 60, but adding additional constraining variables.

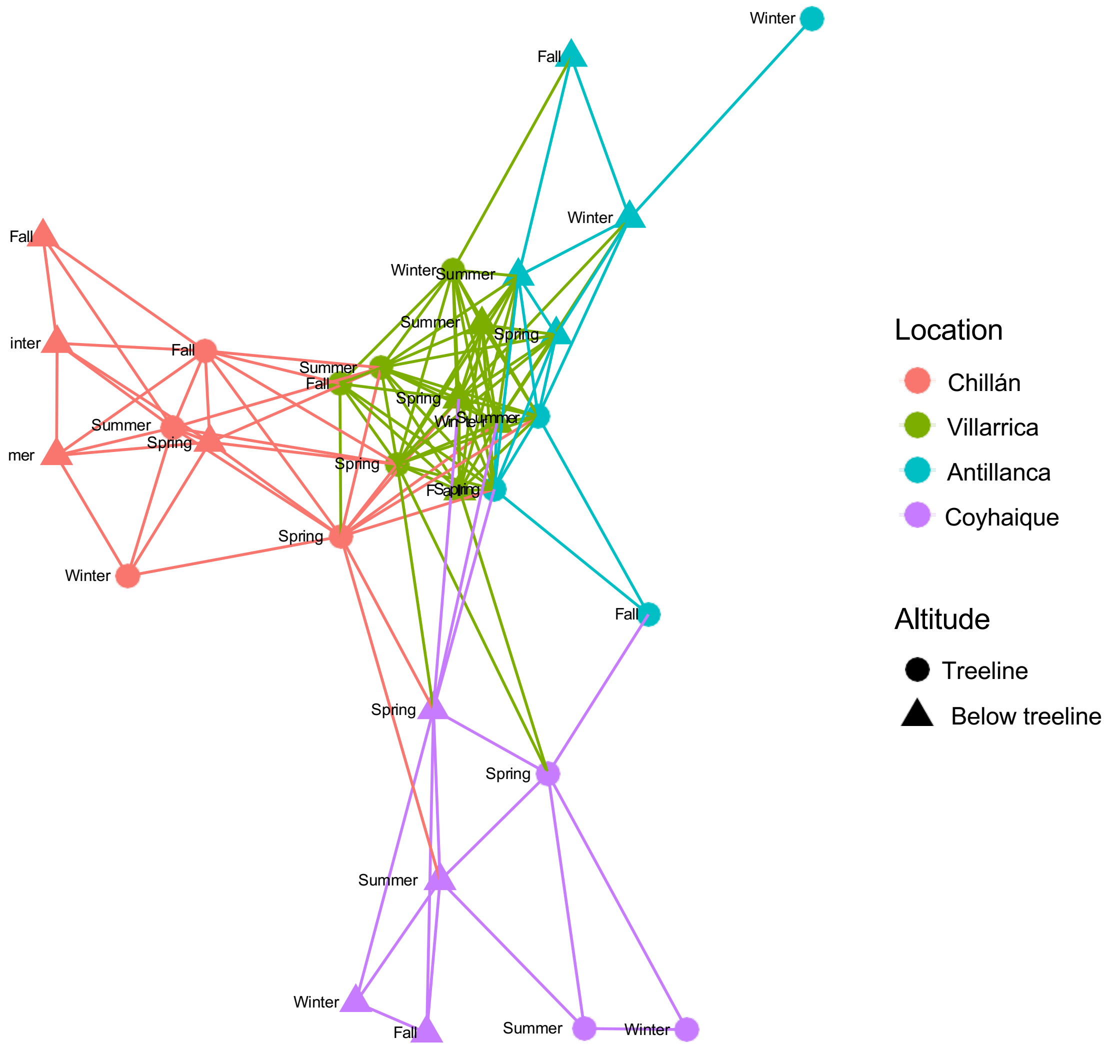

Figure 62: Distance Threshold Network using Hellinger distance (max.dist = 1).

14. Session info

```
devtools::session_info()
```

```
## - Session info ---------------------------------------------------------------
## setting  value
## version  R version 4.3.2 (2023-10-31)
## os       Ubuntu 20.04.6 LTS
## system   x86_64, linux-gnu
## ui       X11
## language (EN)
## collate  C.UTF-8
## ctype    C.UTF-8
## tz       UTC
## date     2024-02-12
## pandoc   3.1.1 @ /usr/lib/rstudio-server/bin/quarto/bin/tools/ (via rmarkdown)
##
## - Packages ---------------------------------------------------------------------
## package       * version  date (UTC) lib source
## ade4            1.7-22   2023-02-06 [1] RSPM (R 4.3.0)
## ape             5.7-1    2023-03-13 [1] RSPM (R 4.3.0)
## Biobase         2.62.0   2023-10-24 [1] Bioconductor
## BiocGenerics    0.48.1   2023-11-01 [1] Bioconductor
## BiocParallel    1.36.0   2023-10-24 [1] Bioconductor
## biomformat      1.30.0   2023-10-24 [1] Bioconductor
## Biostrings      2.70.1   2023-10-25 [1] Bioconductor
## bitops          1.0-7    2021-04-24 [1] RSPM (R 4.3.0)
## bookdown        0.36     2023-10-16 [1] RSPM (R 4.3.0)
## ca              0.71.1   2020-01-24 [1] RSPM (R 4.3.0)
## cachem          1.0.8    2023-05-01 [1] RSPM (R 4.3.0)
## callr           3.7.3    2022-11-02 [1] RSPM (R 4.3.0)
## cli             3.6.1    2023-03-23 [1] RSPM (R 4.3.0)
## cluster         2.1.4    2022-08-22 [2] CRAN (R 4.3.2)
## codetools       0.2-19   2023-02-01 [2] CRAN (R 4.3.2)
## colorspace      2.1-0    2023-01-23 [1] RSPM (R 4.3.0)
## corpcor         1.6.10   2021-09-16 [1] RSPM (R 4.3.0)
## cowplot         1.1.1    2020-12-30 [1] RSPM (R 4.3.0)
## crayon          1.5.2    2022-09-29 [1] RSPM (R 4.3.0)
## data.table      1.14.8   2023-02-17 [1] RSPM (R 4.3.0)
## devtools        2.4.5    2022-10-11 [1] RSPM (R 4.3.0)
## digest          0.6.33   2023-07-07 [1] RSPM (R 4.3.0)
## dplyr         * 1.1.3    2023-09-03 [1] RSPM (R 4.3.0)
## ellipse         0.5.0    2023-07-20 [1] RSPM (R 4.3.0)
## ellipsis        0.3.2    2021-04-29 [1] RSPM (R 4.3.0)
## evaluate        0.23     2023-11-01 [1] RSPM (R 4.3.0)
## fansi           1.0.5    2023-10-08 [1] RSPM (R 4.3.0)
## farver          2.1.1    2022-07-06 [1] RSPM (R 4.3.0)
## fastmap         1.1.1    2023-02-24 [1] RSPM (R 4.3.0)
## foreach         1.5.2    2022-02-02 [1] RSPM (R 4.3.0)
## formatR         1.14     2023-01-17 [1] RSPM (R 4.3.0)
## fs              1.6.3    2023-07-20 [1] RSPM (R 4.3.0)
```

```
## generics 0.1.3 2022-07-05 [1] RSPM (R 4.3.0)
## GenomeInfoDb 1.38.1 2023-11-08 [1] Bioconductor
## GenomeInfoDbData 1.2.11 2023-11-16 [1] Bioconductor
## ggfortify * 0.4.16 2023-03-20 [1] RSPM (R 4.3.0)
## ggmap * 4.0.0 2023-10-19 [1] Github (stadiamaps/ggmap@191159d)
## ggplot2 * 3.4.4 2023-10-12 [1] RSPM (R 4.3.0)
## ggrepel 0.9.4 2023-10-13 [1] RSPM (R 4.3.0)
## ggvegan * 0.1.999 2023-11-29 [1] Github (gavinsimpson/ggvegan@058c08c)
## glue 1.6.2 2022-02-24 [1] RSPM (R 4.3.0)
## gridExtra * 2.3 2017-09-09 [1] RSPM (R 4.3.0)
## gtable 0.3.4 2023-08-21 [1] RSPM (R 4.3.0)
## highr 0.10 2022-12-22 [1] RSPM (R 4.3.0)
## htmltools 0.5.7 2023-11-03 [1] RSPM (R 4.3.0)
## htmlwidgets 1.6.2 2023-03-17 [1] RSPM (R 4.3.0)
## httpuv 1.6.12 2023-10-23 [1] RSPM (R 4.3.0)
## httr 1.4.7 2023-08-15 [1] RSPM (R 4.3.0)
## igraph 1.5.1 2023-08-10 [1] RSPM (R 4.3.0)
## IRanges 2.36.0 2023-10-24 [1] Bioconductor
## iterators 1.0.14 2022-02-05 [1] RSPM (R 4.3.0)
## jpeg 0.1-10 2022-11-29 [1] RSPM (R 4.3.0)
## jsonlite 1.8.7 2023-06-29 [1] RSPM (R 4.3.0)
## kableExtra * 1.3.4.9000 2023-08-29 [1] Github (kupietz/kableExtra@3bf9b21)
## knitr 1.45 2023-10-30 [1] RSPM (R 4.3.0)
## labeling 0.4.3 2023-08-29 [1] RSPM (R 4.3.0)
## later 1.3.1 2023-05-02 [1] RSPM (R 4.3.0)
## lattice * 0.21-9 2023-10-01 [2] CRAN (R 4.3.2)
## lifecycle 1.0.4 2023-11-07 [1] RSPM (R 4.3.0)
## magrittr 2.0.3 2022-03-30 [1] RSPM (R 4.3.0)
## MASS 7.3-60 2023-05-04 [2] CRAN (R 4.3.2)
## Matrix 1.6-3 2023-11-14 [1] RSPM (R 4.3.0)
## matrixStats 1.1.0 2023-11-07 [1] RSPM (R 4.3.0)
## memoise 2.0.1 2021-11-26 [1] RSPM (R 4.3.0)
## metagMisc * 0.5.0 2023-11-14 [1] Github (vmikk/metagMisc@310b1a4)
## mgcv 1.9-0 2023-07-11 [2] CRAN (R 4.3.2)
## microbiome 1.24.0 2023-10-24 [1] Bioconductor
## microViz 0.11.0 2023-11-28 [1] https://david-barnett.r-universe.dev (R 4.3.1)
## mime 0.12 2021-09-28 [1] RSPM (R 4.3.0)
## miniUI 0.1.1.1 2018-05-18 [1] RSPM (R 4.3.0)
## mixOmics 6.26.0 2023-10-24 [1] Bioconductor (R 4.3.1)
## mnormt 2.1.1 2022-09-26 [1] RSPM (R 4.3.0)
## multtest 2.58.0 2023-10-24 [1] Bioconductor
## munsell 0.5.0 2018-06-12 [1] RSPM (R 4.3.0)
## nlme 3.1-163 2023-08-09 [2] CRAN (R 4.3.2)
## permute * 0.9-7 2022-01-27 [1] RSPM (R 4.3.0)
## phyloseq * 1.46.0 2023-10-24 [1] Bioconductor
## pillar 1.9.0 2023-03-22 [1] RSPM (R 4.3.0)
## pkgbuild 1.4.2 2023-06-26 [1] RSPM (R 4.3.0)
## pkgconfig 2.0.3 2019-09-22 [1] RSPM (R 4.3.0)
## pkgload 1.3.3 2023-09-22 [1] RSPM (R 4.3.0)
## plyr 1.8.9 2023-10-02 [1] RSPM (R 4.3.0)
## png 0.1-8 2022-11-29 [1] RSPM (R 4.3.0)
## prettyunits 1.2.0 2023-09-24 [1] RSPM (R 4.3.0)
```

```
## processx        3.8.2     2023-06-30 [1] RSPM (R 4.3.0)
## profvis         0.3.8     2023-05-02 [1] RSPM (R 4.3.0)
## promises        1.2.1     2023-08-10 [1] RSPM (R 4.3.0)
## ps              1.7.5     2023-04-18 [1] RSPM (R 4.3.0)
## psych           2.3.9     2023-09-26 [1] RSPM (R 4.3.0)
## purrr           1.0.2     2023-08-10 [1] RSPM (R 4.3.0)
## R6              2.5.1     2021-08-19 [1] RSPM (R 4.3.0)
## rARPACK         0.11-0    2016-03-10 [1] RSPM (R 4.3.0)
## RColorBrewer    1.1-3     2022-04-03 [1] RSPM (R 4.3.0)
## Rcpp            1.0.11    2023-07-06 [1] RSPM (R 4.3.0)
## RCurl           1.98-1.13 2023-11-02 [1] RSPM (R 4.3.0)
## registry        0.5-1     2019-03-05 [1] RSPM (R 4.3.0)
## remotes         2.4.2.1   2023-07-18 [1] RSPM (R 4.3.0)
## reshape2        1.4.4     2020-04-09 [1] RSPM (R 4.3.0)
## RgoogleMaps     1.5.1     2023-11-06 [1] RSPM (R 4.3.0)
## rhdf5           2.46.0    2023-10-24 [1] Bioconductor
## rhdf5filters    1.14.1    2023-11-06 [1] Bioconductor
## Rhdf5lib        1.24.0    2023-10-24 [1] Bioconductor
## rlang           1.1.2     2023-11-04 [1] RSPM (R 4.3.0)
## rmarkdown       2.25      2023-09-18 [1] RSPM (R 4.3.0)
## RSpectra        0.16-1    2022-04-24 [1] RSPM (R 4.3.0)
## rstudioapi      0.15.0    2023-07-07 [1] RSPM (R 4.3.0)
## rticles         0.25      2023-05-15 [1] RSPM (R 4.3.0)
## Rtsne           0.16      2022-04-17 [1] RSPM (R 4.3.0)
## rvest           1.0.3     2022-08-19 [1] RSPM (R 4.3.0)
## S4Vectors       0.40.1    2023-10-26 [1] Bioconductor
## scales          1.2.1     2022-08-20 [1] RSPM (R 4.3.0)
## seriation       1.5.2     2023-11-26 [1] RSPM (R 4.3.0)
## sessioninfo     1.2.2     2021-12-06 [1] RSPM (R 4.3.0)
## shiny           1.7.5.1   2023-10-14 [1] RSPM (R 4.3.0)
## stringi         1.8.1     2023-11-13 [1] RSPM (R 4.3.0)
## stringr       * 1.5.1     2023-11-14 [1] RSPM (R 4.3.0)
## survival        3.5-7     2023-08-14 [2] CRAN (R 4.3.2)
## svglite         2.1.2     2023-10-11 [1] RSPM (R 4.3.0)
## systemfonts     1.0.5     2023-10-09 [1] RSPM (R 4.3.0)
## tibble        * 3.2.1     2023-03-20 [1] RSPM (R 4.3.0)
## tidyr         * 1.3.0     2023-01-24 [1] RSPM (R 4.3.0)
## tidyselect      1.2.0     2022-10-10 [1] RSPM (R 4.3.0)
## TSP             1.2-4     2023-04-04 [1] RSPM (R 4.3.0)
## urlchecker      1.0.1     2021-11-30 [1] RSPM (R 4.3.0)
## usethis         2.2.2     2023-07-06 [1] RSPM (R 4.3.0)
## utf8            1.2.4     2023-10-22 [1] RSPM (R 4.3.0)
## vctrs           0.6.4     2023-10-12 [1] RSPM (R 4.3.0)
## vegan         * 2.6-4     2022-10-11 [1] RSPM (R 4.3.0)
## viridisLite     0.4.2     2023-05-02 [1] RSPM (R 4.3.0)
## webshot         0.5.5     2023-06-26 [1] RSPM (R 4.3.0)
## withr           2.5.2     2023-10-30 [1] RSPM (R 4.3.0)
## xfun            0.41      2023-11-01 [1] RSPM (R 4.3.0)
## xml2            1.3.5     2023-07-06 [1] RSPM (R 4.3.0)
## xtable          1.8-4     2019-04-21 [1] RSPM (R 4.3.0)
## XVector         0.42.0    2023-10-24 [1] Bioconductor
## yaml            2.3.7     2023-01-23 [1] RSPM (R 4.3.0)
```

```
## zlibbioc            1.48.0    2023-10-24 [1] Bioconductor ##
## [1] /cloud/lib/x86_64-pc-linux-gnu-library/4.3
## [2] /opt/R/4.3.2/lib/R/library ##
## ──────────────────────────────────────────────────────────────────────────────
```
