# Supplementary material for: High ectomycorrhizal relative abundance during winter at the treeline
Source: ISME Commun. 2025 Jan 25;5(1):ycaf010. doi: 10.1093/ismeco/ycaf010 (PMC11815889; doi:10.1093/ismeco/ycaf010)
Supplement: 250122_Supplementary_Legends_ycaf010 [file 250122_supplementary_legends_ycaf010.docx]

**Supplementary Legends**

**Supplementary Figure Legends**

**Figure S1. Rarefaction curves of rhizosphere fungal communities in Nothofagus forests.** Rarefaction curves displaying species richness as a function of sequence sample size for each sample collected across different locations and seasons. The samples are color-coded by location. Each curve represents a different sample, indicating the variation in species richness and sequencing depth among the samples.

**Figure S2. Observed ASVs and Simpson Index residual diversity plots.** (A) Residual diversity plots for the observed number of ASVs illustrating the marginal contribution of location (left panel), altitude (center panel), and season (right panel) to the overall diversity variation. Each panel includes violin plots representing the density function of model predictions. Letters above the violin plots indicate significant differences among groups based on post hoc tests. (B) Residual diversity plots for the Simpson Index, illustrating the same factors: location (left panel), altitude (center panel), and season (right panel). These plots highlight the differences in fungal diversity across various environmental gradients and seasonal changes, providing a detailed understanding of microbial community structure in Patagonian *Nothofagus* forests.

**Figure S3. ASVs relative abundance in rhizosphere fungal communities.** (A) Venn diagram showing the number of shared and unique ASVs across different seasons (Fall, Winter, Spring, Summer). The heatmap to the right displays the relative abundance of the top 10 fungal families among the 323 ASVs shared across all seasons. (B) Venn diagram illustrating the distribution of ASVs across the four study locations (Chillán, Villarrica, Antillanca, Coyhaique). The accompanying heatmap shows the relative abundance of the top 10 fungal families among the 100 ASVs common to all locations. (C) Venn diagram depicting ASVs shared between two altitudes (Treeline and Below Treeline). The heatmap to the right indicates the relative abundance of the top 10 fungal families among the 972 ASVs shared between these altitudinal zones.

**Figure S4. Transformation-based redundancy analysis (tb-RDA) plot of summer fungal guilds with edaphic variables.** Plot displaying the tb-RDA results of fungal guild data from summer, constrained by selected edaphic variables. Nutrient content variables (OM, N, P, S) were included through a stepwise selection procedure. The plot shows the relationship between fungal guilds and the constrained variables, with locations color-coded. Altitude is distinguished by different symbols: Below treeline (filled circles) and Treeline (open circles). The plot illustrates how environmental variables influence the distribution and composition of fungal guilds in the rhizosphere of *Nothofagus* forests during the summer season.

**Supplementary Table Legends**

**Supplementary Table 1.** Environmental characteristics of the sampling sites forests.

**Supplementary Table 2.** Read counts for each sample divided per pool.

**Supplementary Table 3.** Sample metadata of the phyloseq object.

**Supplementary Table 4.** Taxonomic, functional assignment and relative abundance of each ASV identified per sample.

**Supplementary Table 5.** Percentage abundance of fungal phyla across different seasons, locations, and altitudes in Nothofagus pumilio rhizosphere samples.

**Supplementary Table 6.** Chemical analysis of the rhizospheric soil samples from Lenga obtained in summer season.
